# Deep learning empowers genomic selection of pest-resistant grapevine

**DOI:** 10.1093/hr/uhaf128

**Published:** 2025-05-07

**Authors:** Yu Gan, Zhenya Liu, Fan Zhang, Qi Xu, Xu Wang, Hui Xue, Xiangnian Su, Wenqi Ma, Qiming Long, Anqi Ma, Guizhou Huang, Wenwen Liu, Xiaodong Xu, Lei Sun, Yingchun Zhang, Yuting Liu, Xinyue Fang, Chaochao Li, Xuanwen Yang, Pengcheng Wei, Xiucai Fan, Chuan Zhang, Pengpai Zhang, Chonghuai Liu, Lianzhu Zhou, Zhiwu Zhang, Yiwen Wang, Zhongjie Liu, Yongfeng Zhou

**Affiliations:** National Key Laboratory of Tropical Crop Breeding, Tropical Crops Genetic Resources Institute, Chinese Academy of Tropical Agricultural Sciences, Xueyuan Road, Longhua District, Haikou, 571101, China; National Key Laboratory of Tropical Crop Breeding, Shenzhen Branch, Guangdong Laboratory of Lingnan Modern Agriculture, Key Laboratory of Synthetic Biology, Ministry of Agriculture and Rural Affairs, Agricultural Genomics Institute at Shenzhen, Chinese Academy of Agricultural Sciences, Buxin Road, Dapeng New District, Shenzhen, 518000, China; Department of Prenatal Diagnosis Center, Women and Children's Hospital of Chongqing Medical University, Chongqing, 401147, China; National Key Laboratory of Tropical Crop Breeding, Tropical Crops Genetic Resources Institute, Chinese Academy of Tropical Agricultural Sciences, Xueyuan Road, Longhua District, Haikou, 571101, China; National Key Laboratory of Tropical Crop Breeding, Shenzhen Branch, Guangdong Laboratory of Lingnan Modern Agriculture, Key Laboratory of Synthetic Biology, Ministry of Agriculture and Rural Affairs, Agricultural Genomics Institute at Shenzhen, Chinese Academy of Agricultural Sciences, Buxin Road, Dapeng New District, Shenzhen, 518000, China; National Key Laboratory of Tropical Crop Breeding, Tropical Crops Genetic Resources Institute, Chinese Academy of Tropical Agricultural Sciences, Xueyuan Road, Longhua District, Haikou, 571101, China; National Key Laboratory of Tropical Crop Breeding, Shenzhen Branch, Guangdong Laboratory of Lingnan Modern Agriculture, Key Laboratory of Synthetic Biology, Ministry of Agriculture and Rural Affairs, Agricultural Genomics Institute at Shenzhen, Chinese Academy of Agricultural Sciences, Buxin Road, Dapeng New District, Shenzhen, 518000, China; National Key Laboratory of Tropical Crop Breeding, Shenzhen Branch, Guangdong Laboratory of Lingnan Modern Agriculture, Key Laboratory of Synthetic Biology, Ministry of Agriculture and Rural Affairs, Agricultural Genomics Institute at Shenzhen, Chinese Academy of Agricultural Sciences, Buxin Road, Dapeng New District, Shenzhen, 518000, China; National Key Laboratory of Tropical Crop Breeding, Tropical Crops Genetic Resources Institute, Chinese Academy of Tropical Agricultural Sciences, Xueyuan Road, Longhua District, Haikou, 571101, China; National Key Laboratory of Tropical Crop Breeding, Shenzhen Branch, Guangdong Laboratory of Lingnan Modern Agriculture, Key Laboratory of Synthetic Biology, Ministry of Agriculture and Rural Affairs, Agricultural Genomics Institute at Shenzhen, Chinese Academy of Agricultural Sciences, Buxin Road, Dapeng New District, Shenzhen, 518000, China; National Key Laboratory of Tropical Crop Breeding, Tropical Crops Genetic Resources Institute, Chinese Academy of Tropical Agricultural Sciences, Xueyuan Road, Longhua District, Haikou, 571101, China; National Key Laboratory of Tropical Crop Breeding, Shenzhen Branch, Guangdong Laboratory of Lingnan Modern Agriculture, Key Laboratory of Synthetic Biology, Ministry of Agriculture and Rural Affairs, Agricultural Genomics Institute at Shenzhen, Chinese Academy of Agricultural Sciences, Buxin Road, Dapeng New District, Shenzhen, 518000, China; National Key Laboratory of Tropical Crop Breeding, Tropical Crops Genetic Resources Institute, Chinese Academy of Tropical Agricultural Sciences, Xueyuan Road, Longhua District, Haikou, 571101, China; National Key Laboratory of Tropical Crop Breeding, Shenzhen Branch, Guangdong Laboratory of Lingnan Modern Agriculture, Key Laboratory of Synthetic Biology, Ministry of Agriculture and Rural Affairs, Agricultural Genomics Institute at Shenzhen, Chinese Academy of Agricultural Sciences, Buxin Road, Dapeng New District, Shenzhen, 518000, China; National Key Laboratory of Tropical Crop Breeding, Shenzhen Branch, Guangdong Laboratory of Lingnan Modern Agriculture, Key Laboratory of Synthetic Biology, Ministry of Agriculture and Rural Affairs, Agricultural Genomics Institute at Shenzhen, Chinese Academy of Agricultural Sciences, Buxin Road, Dapeng New District, Shenzhen, 518000, China; National Key Laboratory of Tropical Crop Breeding, Shenzhen Branch, Guangdong Laboratory of Lingnan Modern Agriculture, Key Laboratory of Synthetic Biology, Ministry of Agriculture and Rural Affairs, Agricultural Genomics Institute at Shenzhen, Chinese Academy of Agricultural Sciences, Buxin Road, Dapeng New District, Shenzhen, 518000, China; National Key Laboratory of Tropical Crop Breeding, Shenzhen Branch, Guangdong Laboratory of Lingnan Modern Agriculture, Key Laboratory of Synthetic Biology, Ministry of Agriculture and Rural Affairs, Agricultural Genomics Institute at Shenzhen, Chinese Academy of Agricultural Sciences, Buxin Road, Dapeng New District, Shenzhen, 518000, China; National Key Laboratory of Tropical Crop Breeding, Shenzhen Branch, Guangdong Laboratory of Lingnan Modern Agriculture, Key Laboratory of Synthetic Biology, Ministry of Agriculture and Rural Affairs, Agricultural Genomics Institute at Shenzhen, Chinese Academy of Agricultural Sciences, Buxin Road, Dapeng New District, Shenzhen, 518000, China; National Key Laboratory of Tropical Crop Breeding, Shenzhen Branch, Guangdong Laboratory of Lingnan Modern Agriculture, Key Laboratory of Synthetic Biology, Ministry of Agriculture and Rural Affairs, Agricultural Genomics Institute at Shenzhen, Chinese Academy of Agricultural Sciences, Buxin Road, Dapeng New District, Shenzhen, 518000, China; National Key Laboratory of Tropical Crop Breeding, Tropical Crops Genetic Resources Institute, Chinese Academy of Tropical Agricultural Sciences, Xueyuan Road, Longhua District, Haikou, 571101, China; National Key Laboratory of Tropical Crop Breeding, Shenzhen Branch, Guangdong Laboratory of Lingnan Modern Agriculture, Key Laboratory of Synthetic Biology, Ministry of Agriculture and Rural Affairs, Agricultural Genomics Institute at Shenzhen, Chinese Academy of Agricultural Sciences, Buxin Road, Dapeng New District, Shenzhen, 518000, China; Zhengzhou Fruit Research Institute, Chinese Academy of Agricultural Sciences, Southern End of Weilai Road, Guancheng District, Zhengzhou, 450009, China; National Key Laboratory of Tropical Crop Breeding, Shenzhen Branch, Guangdong Laboratory of Lingnan Modern Agriculture, Key Laboratory of Synthetic Biology, Ministry of Agriculture and Rural Affairs, Agricultural Genomics Institute at Shenzhen, Chinese Academy of Agricultural Sciences, Buxin Road, Dapeng New District, Shenzhen, 518000, China; National Key Laboratory of Tropical Crop Breeding, Shenzhen Branch, Guangdong Laboratory of Lingnan Modern Agriculture, Key Laboratory of Synthetic Biology, Ministry of Agriculture and Rural Affairs, Agricultural Genomics Institute at Shenzhen, Chinese Academy of Agricultural Sciences, Buxin Road, Dapeng New District, Shenzhen, 518000, China; National Key Laboratory of Tropical Crop Breeding, Shenzhen Branch, Guangdong Laboratory of Lingnan Modern Agriculture, Key Laboratory of Synthetic Biology, Ministry of Agriculture and Rural Affairs, Agricultural Genomics Institute at Shenzhen, Chinese Academy of Agricultural Sciences, Buxin Road, Dapeng New District, Shenzhen, 518000, China; National Key Laboratory of Tropical Crop Breeding, Tropical Crops Genetic Resources Institute, Chinese Academy of Tropical Agricultural Sciences, Xueyuan Road, Longhua District, Haikou, 571101, China; National Key Laboratory of Tropical Crop Breeding, Shenzhen Branch, Guangdong Laboratory of Lingnan Modern Agriculture, Key Laboratory of Synthetic Biology, Ministry of Agriculture and Rural Affairs, Agricultural Genomics Institute at Shenzhen, Chinese Academy of Agricultural Sciences, Buxin Road, Dapeng New District, Shenzhen, 518000, China; National Key Laboratory of Tropical Crop Breeding, Tropical Crops Genetic Resources Institute, Chinese Academy of Tropical Agricultural Sciences, Xueyuan Road, Longhua District, Haikou, 571101, China; National Key Laboratory of Tropical Crop Breeding, Shenzhen Branch, Guangdong Laboratory of Lingnan Modern Agriculture, Key Laboratory of Synthetic Biology, Ministry of Agriculture and Rural Affairs, Agricultural Genomics Institute at Shenzhen, Chinese Academy of Agricultural Sciences, Buxin Road, Dapeng New District, Shenzhen, 518000, China; National Key Laboratory of Tropical Crop Breeding, Tropical Crops Genetic Resources Institute, Chinese Academy of Tropical Agricultural Sciences, Xueyuan Road, Longhua District, Haikou, 571101, China; Zhengzhou Fruit Research Institute, Chinese Academy of Agricultural Sciences, Southern End of Weilai Road, Guancheng District, Zhengzhou, 450009, China; State Key Laboratory of Genetic Improvement and Germplasm Innovation of Crop Resistance in Arid Desert Regions (Preparation), Key Laboratory of Genome Research and Genetic Improvement of Xinjiang Characteristic Fruits and Vegetables, Institute of Horticultural Crops, Xinjiang Academy of Agricultural Sciences, Nanchang Road, Urumqi, 830091, China; School of Life Sciences, Henan University, Minglun Street, Kaifeng, 475004, China; Zhengzhou Fruit Research Institute, Chinese Academy of Agricultural Sciences, Southern End of Weilai Road, Guancheng District, Zhengzhou, 450009, China; National Key Laboratory of Tropical Crop Breeding, Tropical Crops Genetic Resources Institute, Chinese Academy of Tropical Agricultural Sciences, Xueyuan Road, Longhua District, Haikou, 571101, China; National Key Laboratory of Tropical Crop Breeding, Shenzhen Branch, Guangdong Laboratory of Lingnan Modern Agriculture, Key Laboratory of Synthetic Biology, Ministry of Agriculture and Rural Affairs, Agricultural Genomics Institute at Shenzhen, Chinese Academy of Agricultural Sciences, Buxin Road, Dapeng New District, Shenzhen, 518000, China; Department of Crop and Soil Sciences, Washington State University, Pullman, WA, 646420, USA; National Key Laboratory of Tropical Crop Breeding, Tropical Crops Genetic Resources Institute, Chinese Academy of Tropical Agricultural Sciences, Xueyuan Road, Longhua District, Haikou, 571101, China; National Key Laboratory of Tropical Crop Breeding, Shenzhen Branch, Guangdong Laboratory of Lingnan Modern Agriculture, Key Laboratory of Synthetic Biology, Ministry of Agriculture and Rural Affairs, Agricultural Genomics Institute at Shenzhen, Chinese Academy of Agricultural Sciences, Buxin Road, Dapeng New District, Shenzhen, 518000, China; National Key Laboratory of Tropical Crop Breeding, Tropical Crops Genetic Resources Institute, Chinese Academy of Tropical Agricultural Sciences, Xueyuan Road, Longhua District, Haikou, 571101, China; Institute of Life and Health, China Resources Research Institute of Science and Technology, Pak Shek Kok Road, Sha Tin District, Hong Kong, 999077, China; National Key Laboratory of Tropical Crop Breeding, Tropical Crops Genetic Resources Institute, Chinese Academy of Tropical Agricultural Sciences, Xueyuan Road, Longhua District, Haikou, 571101, China

## Abstract

Crop pests significantly reduce crop yield and threaten global food security. Conventional pest control relies heavily on insecticides, leading to pesticide resistance and ecological concerns. However, crops and their wild relatives exhibit varied levels of pest resistance, suggesting the potential for breeding pest-resistant varieties. This study integrates deep learning (DL)/machine learning (ML) algorithms, plant phenomics, quantitative genetics, and transcriptomics to conduct genomic selection (GS) of pest resistance in grapevine. Building deep convolutional neural networks (DCNNs), we accurately assess pest damage on grape leaves, achieving 95.3% classification accuracy (VGG16) and a 0.94 correlation in regression analysis (DCNN-PDS). The pest damage was phenotyped as binary and continuous traits, and genome resequencing data from 231 grapevine accessions were combined in a Genome-Wide Association Studies, which maps 69 quantitative trait locus (QTLs) and 139 candidate genes involved in pest resistance pathways, including jasmonic acid, salicylic acid, and ethylene. Combining this with transcriptome data, we pinpoint specific pest-resistant genes such as *ACA12* and *CRK3*, which are crucial in herbivore responses. ML-based GS demonstrates a high accuracy (95.7%) and a strong correlation (0.90) in predicting pest resistance as binary and continuous traits in grapevine, respectively. In general, our study highlights the power of DL/ML in plant phenomics and GS, facilitating genomic breeding of pest-resistant grapevine.

## Introduction

The adverse impacts of agricultural pests have long posed challenges for agriculture [1]. Pests, such as aphids, locusts, and moth flies, directly damage crops, resulting in reduced yields and compromised quality [2]. Other pests like scale insects and thrips indirectly harm crops by vectoring plant viruses [3]. The strong reproductive ability of these pests not only decreases crop production but also threatens food security [4]. Traditionally, farmers have primarily relied on copious applications of pesticides to control crop pests. However, the excessive use of pesticides contaminated the environment and jeopardized human and animal health [5]. Moreover, the rapid evolution of pesticide resistance among insect populations has progressively diminished the efficacy of these chemicals [6]. Thus, new monitoring methods and control strategies are needed to precisely control agricultural pests.

Plants and insects have coexisted for over 350 million years [7]. As stationary organisms, plants lack the ability to escape attacks from other organisms, requiring the use of alternative strategies for self-defense. To counter the herbivore attack, plants develop specialized structures or produce secondary metabolites and proteins with toxic, repellent, or antinutritional effects to deter herbivores [8, 9]. Plants defend against herbivores both directly, by altering the preferences, survival, and reproductive success of herbivores, and indirectly, by emitting volatile organic compounds to attract their natural enemies [10]. The defense mechanisms of plants against herbivore attacks involve intricate signal transduction pathways mediated by a network of phytohormones. Plant hormones play a pivotal role in governing plant growth, development, and defense mechanisms [11]. Several plant hormones play roles in both intra- and interplant communication in herbivore-damaged plants. The majority of defense responses against insects are triggered by signal transduction pathways involving jasmonic acid (JA), salicylic acid (SA), and ethylene [10]. Distinct sets of genes associated with defense are activated by these pathways in response to injury or pest feeding. These hormones can work independently, synergistically, or antagonistically, depending on the specific attacker.

One major pest posing prominent threats to grape yield and quality is the tobacco cutworm (*Spodoptera litura*, Fab, SLF) [12]. In grape cultivation, infestations generally occur between July and September, especially in drought conditions, in high humidity, and in proximity to vegetable crops. The larvae of SLF damage the plant mainly by consuming leaf mesophyll tissue, resulting in perforated foliage [13]. Because outbreaks mainly transpire during grape ripening and harvest, extensive feeding can disrupt grape coloring and maturation in the same year. This also impedes nutrient translocation in the following season, leading to poor subsequent bud break and flowering [14, 15]. Although a few grape varieties show resistance to SLF, resistant varieties have not been selected and bred to resist SLF infestations. Therefore, determining more resistance germplasm resources, genetics of plant resistance to SLF and the function of resistance genes have been essential for breeding pest-resistant grapevine.

While the throughput and cost-effectiveness of genotyping have improved considerably [16–18], the measurement of traits of interest remains insufficient in these regards. Most of the resistance traits have been subjected to visual and sensory evaluations by experienced professional breeders, and phenotypic values of the traits are expressed as qualitative categorical scores [19]. For example, drought stress level is categorized as mild, moderate, and severe (three categories) [20], and downy mildew level is categorized into seven categories [21]. An empirical assessment based on the sense of the breeder is not sufficient to evaluate the diverse and continuous variations of the plant resistance. In addition, expertise in visual and sensory evaluation can only be obtained after years of training, and increasing the number of specialized breeders is not practical for large-scale and low-cost phenotyping. To further improve the accuracy of Genome-Wide Association Studies (GWAS) and GS for practical breeding, we need to enhance the data quality of the agronomic traits [22]. Deep learning (DL)-based image analysis offers a promising approach to address the limitations of current qualitative evaluation methods.

DL is a subset of machine learning (ML) that utilizes artificial neural networks to learn from large volumes of data. Deep neural networks contain multiple hidden layers between the input and output layers that enable the model to progressively extract features at multiple levels of abstraction [23]. This hierarchical learning architecture allows DL models to learn complex functions and find intricate patterns in data that most traditional ML techniques may fail to detect. In agriculture, DL has emerged as a promising approach for improving the detection and classification of pests, diseases, and other anomalies. For example, Mohanty *et al.* [24] developed a deep convolutional neural network able to detect 14 crop diseases and 26 crop species with an accuracy exceeding 95%. Sladojevic *et al.* [25] used deep neural networks for automated identification of plant diseases from leaf images, achieving an accuracy of 96.3% compared to 85.9% with a shallow neural network. Overall, DL allows predictive models for agricultural applications to achieve greater complexity and abstraction by learning directly from raw data, enabling more accurate and nuanced detection capabilities than traditional analytical techniques. The ongoing explosion of agricultural big data presents an ideal opportunity for DL techniques to continue advancing in this area.

In this study, we developed DL models that combined object detection and pest severity assessment to generate pest resistance phenotype data from 231 grapevine accessions. Using GWAS, we identified QTL regions associated with resistance and identified pest resistance-related candidate genes within these regions. Furthermore, we integrated GWAS with population genetic analysis, transcriptomic assays, comparisons among different *Vitis* populations, and interspecies comparisons to determine which genomic regions contribute to resistance, as well as to identify potential candidate resistance genes within these regions. Finally, we employed ML-based GS to evaluate different models in breeding programs of pest-resistant grapevine. Overall, our research deepens insight into pest resistance genetics and evolution, promoting advanced genomic breeding strategies for grapevine resistance, and outlines a pathway for potential broader application to other crops.

## Results

### The performances and cross-validations of different binary-classification models

**Fig. 1:**
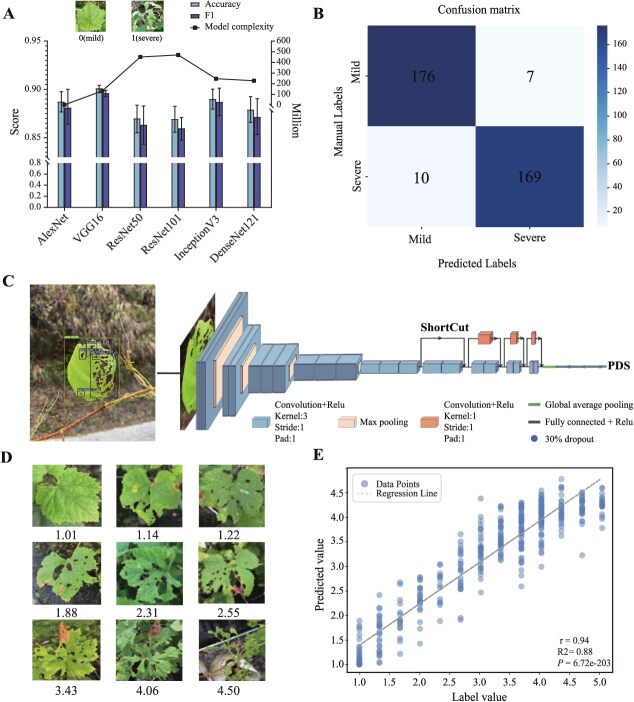
**The DL model (DCNN) for phenotyping pest resistance as binary and continuous traits. A**, Performance of six classic convolutional neural networks in CV. The bars correspond to the left Y-axis (accuracy score), while the line is referenced to the right Y-axis (model complexity). **B**, The number of correct recognitions of different categories by VGG16 in the test set. **C**, The model architecture of DCNN-PDS and the workflow for deriving continuous traits from images. The model uses pretrained VGG16 for initial feature extraction, followed by four residual blocks (each consisting of two convolutional layers) to enhance deep feature learning and prevent degradation. A global average pooling (GAP) layer is then applied to reduce the feature dimensions, and three fully connected layers are used for feature fusion. **D**, Examples of the DCNN-PDS prediction results for the different extent of damages by pest, and the PDS values are shown at the bottom of each plot. **E**, The correlation between the predicted values provided by DCNN-PDS and the manually labeled values in the test set.

To build the binary classification models for the categorization of damage levels caused by insects, we employed a set of deep convolutional neural networks (DCNNs), including AlexNet, VGG16, ResNet50, ResNet101, InceptionV3, and DenseNet121. The model performance was rigorously evaluated through cross-validation (CV) with the aim of identifying the most optimal hyperparameters. After hyperparameter tuning, the best model was then selected based on the results from the test set. The entire CV process utilized a total of 1448 leaf images for training and validation purposes. After choosing the best hyperparameters and training the models for sufficient epochs to ensure convergence, the highest average accuracy was achieved by VGG16 at 90.1%, while the lowest accuracy was observed in ResNet101, scoring 86.9%. Other models fell within the range of 87% to 89% accuracy (Fig. 1A). Correspondingly, VGG16 also exhibited the highest average F1 score, reaching 0.90, while others ranged from 0.86 to 0.89.

Under the condition that all six models are optimized, we further used the test set (362 images) to determine which of the six models is most suitable for our binary classification task. We found that VGG16 continued to perform the best on the test set, achieving an accuracy of 95.3% (Supplementary Table 1). Upon scrutinizing the confusion matrix, it was evident that the model accurately classified 169 positive and 176 negative samples (Fig. 1B). We then performed a correlation analysis between accuracy, F1 score, and model complexity. This revealed a negative correlation trend between the model’s complexity and its performance (Supplementary Fig. 1). VGG16 exhibited the best performance with a relatively low complexity, making it our choice for the binary classification task. The learning curves of VGG16 were shown in Supplementary Fig. 3.

### The performance of DCNN-PDS on continuous traits

To further enhance the granularity of damage assessment caused by pests on leaves, we devised and trained a regression model to assign continuous scores ranging from 0–5, simultaneously providing more suitable data for genetic analysis (Fig. 1C). The pest damage regression model underwent CV and was subsequently evaluated on an independent test set to ensure that there was no overfitting. During CV, a dataset of 2181 images was used to identify the optimal hyperparameters configuration. Through hyperparameter tuning, the model achieved an average mean squared error (MSE) loss of 0.1245 and an average mean absolute error (MAE) loss of 0.2343 in CV (Supplementary Table 2). After the model was configured, formal training was conducted. The model was initially set to train for 300 epochs. Because of early stopping, the training was halted after 218 epochs (Supplementary Fig. 4). After DCNN-PDS (pest damage score) converged, the MSE loss decreased to 0.0241 on the training set and 0.0866 on the validation set, while the MAE dropped to 0.1061 for the training set and 0.1845 for the validation set.

Before applying the model in practical settings, we captured 439 new images for testing to ensure its generalization and robustness (Supplementary Fig. 5). During the testing phase, the model achieved an MSE of 0.1946 and an MAE of 0.3504 on the test set. The DCCN-PDS prediction example showcases the model’s precise quantification of subtle variations in pest damage severity (Fig. 1D). To further analyze the relationship between the predicted values and the manual tag values, a correlation analysis was performed. A significant correlation (*P* < 0.001) between the manual tag values and the predicted values on the test set was observed, as indicated by a Pearson correlation coefficient of 0.94, with a Coefficient of Determination *R^2^* = 0.88 (Fig. 1E).

### Phenotypic assessment of grape accessions using binary classifications and regression models

**Fig. 2:**
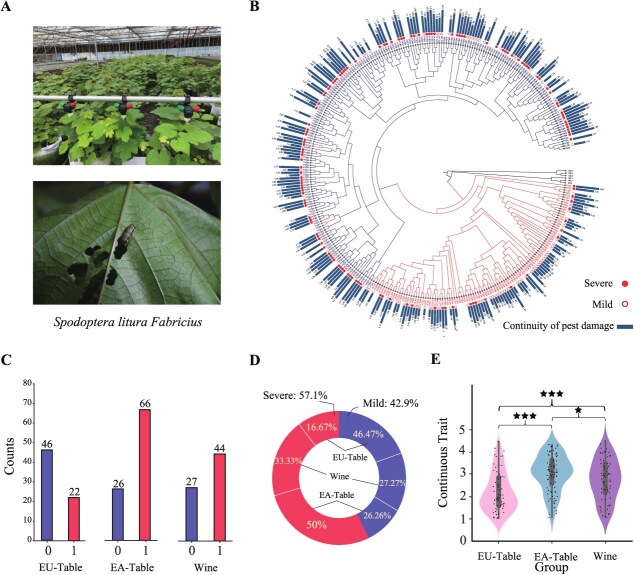
**The overall phenotype of pest resistance in grapevine populations. A**, The greenhouse conditions and the experimental setup for SLF infestation. **B**, The evolutionary tree of 323 individuals is divided into two populations: Europe and America, and Eurasia. The bar chart displays continuous trait values for each accession, with solid dots indicating severity in binary traits. The footrule distance between the species order generated by the phylogenetic tree and the insect resistance order is 19405, whereas the average distance from 1000 randomly generated ordering simulations is 23 102, a significant deviation from the observed result (one-sample *t*-test, *P* < 2.2 × 10^−16^). **C**, The bar chart shows VGG16-classified grape populations, with distict patterns in the legend indicating healthy (x-axis values ‘0’) and severely pest-damaged samles (x-axis value ‘1’). **D**, A pie chart depicting the overall binary classification phenotypes, describing the percentage of their respective binary phenotypes. **E**, Violin plots depicting the distribution of continuous phenotypes for the three categories, with significant differences denoted by an asterisk (★: *P* < 0.05, ★★★: *P* < 0.001).

To assess the pest resistance of grapevine populations, we collected cuttings of 231 accessions from National Grape Germplasm at Zhengzhou in November 2021 and cultivated in greenhouse at the Shenzhen station (Fig. 2A and Supplementary Fig. 6). After the outbreak of SLF in the greenhouse in June–August 2022 (Fig. 2A, see Methods), we randomly took pictures for 6–10 leaves for each accession in September 2022 to represent the pest resistance of the sample. A population structure analysis was performed on a total of 231 grape varieties, including table grape varieties domesticated from *Vitis vinifera* ssp. *sylvestris* (EU-Table), table grape varieties originated from hybridization between *V. vinifera* and *Vitis labrusca* (EA-Table), and wine-making grape varieties domesticated from *V. vinifera* ssp. *sylvestris* (Wine) (Fig. 2 and Supplementary Table 3). The examination utilized both the VGG16 model and our custom-developed model DCNN-PDS, resulting in binary and continuous phenotypes (Fig. 2B). Binary classification results revealed 99 varieties exhibited mild damage by pests and 132 varieties have suffered severe pest damage (Supplementary Fig. 7). The EA-Table seems to be the least resistant to pests, with 66 out of 92 accessions experiencing severe infestation (50% of the total severe grape accessions). In contrast, the EU-Table appears to have stronger pest resistance, with 46 varieties identified as having mild infestation (46.47% of the total instances of mild damage) (Fig. 2C and D).

DCCN-PDS predicted and scored damage levels in 0–5 for all accessions based on their images (Supplementary Fig. 7). Regression analysis of the leaf images revealed the EU-Table group had the lowest mean and median values at 2.22 and 1.94, respectively (Fig. 2E). Normality testing using the Shapiro–Wilk test indicated the EU-wine group followed a normal distribution (*P* = 0.082), while the *P* values for the other two groups were below 0.05 (Supplementary Fig. 8). Homogeneity of variance among the three groups was supported by a Bartlett test (*P* = 0.5542). Given these results, the Wilcoxon rank sum test was used to detect differences between groups and found significant differences between all three. It was evident that, compared to the EA-Table and EA-wine groups, the EU-Table exhibited significant (*P* < 0.001) phenotypic distinctions, indicating a generally heightened pest resistance compared to the aforementioned two populations (Fig. 2E). Phylogenetic regression was performed by mapping regression phenotypes onto the phylogenetic tree (Fig. 2B), and correlation and Spearman’s footrule distance were calculated. The correlation between regression value order and tree order was 0.2747, with a Spearman’s footrule distance of 19 405. In contrast, the average correlation of random sequences was −3.09e−4, with an average distance of 23 102. Single sample *t*-tests showed both analyses were significantly nonrandom (*P* < 2.2e−16), suggesting a significant association between pest resistance and phylogenetic evolution.

### Genome-wide Association Study of binary and continuous pest-resistant traits

**Fig. 3:**
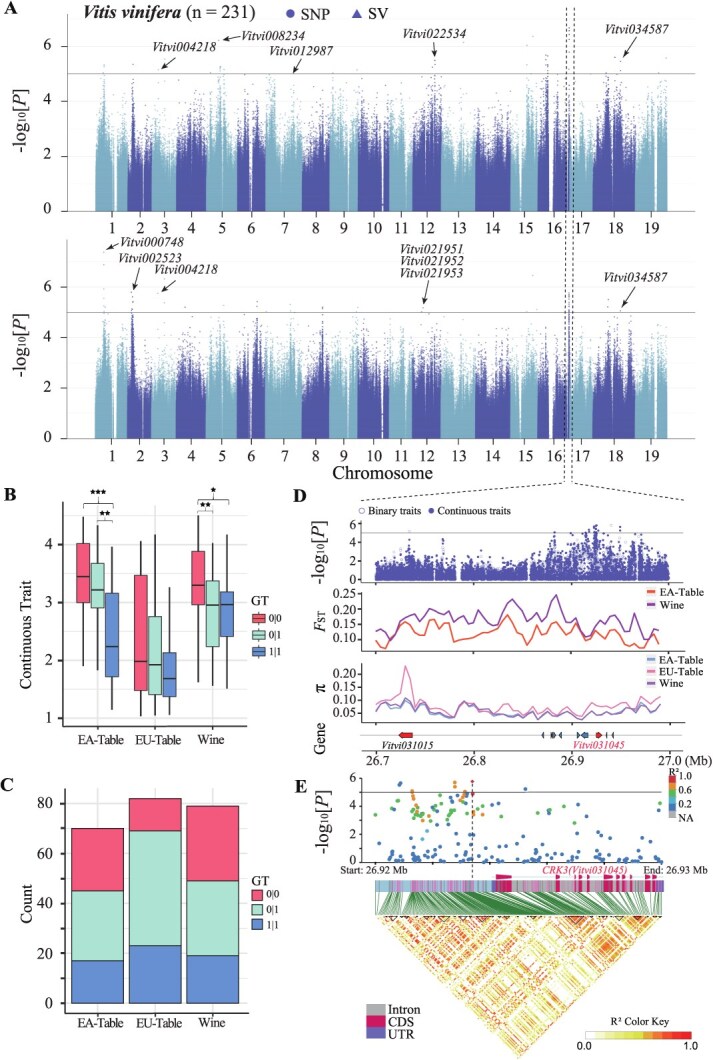
**GWAS mapping for binary and continuous traits of pest resistance in grapevine. A**, The Manhattan plot reveals GWAS results for two phenotypes, with the upper section representing binary phenotype results and the lower section representing continuous phenotype results. **B**, A boxplot depicting the continuous phenotype corresponding to the genotype of snp16_26926513 in three grape populations; the asterisk indicates significant differences between them (★: *P* < 0.05, ★★: *P* < 0.01, ★★★: *P* < 0.001). **C**, The number of individuals corresponding to three genotypes in three populations, where 0/0 represents no mutation, 0/1 represents heterozygous mutation, and 1/1 represents homozygous mutation. **D**, The region on chromosome 16 (chr16: 26.7–27.0 Mb) was zoomed in, sequentially illustrating the corresponding GWAS results, *F*_ST_, π, and the situation of the corresponding genes. **E**, LD analysis was conducted for the gene *Vitvi031045* and an upstream segment (chr16: 26.92–26.93 Mb), with the upper section revealing the *P* values of the GWAS results.

To further map the quantitative genetic basis of pest damage, we conducted GWAS for binary traits derived from VGG16 and for continuous traits obtained from DCNN-PDS, respectively (Fig. 3A and Supplementary Fig. 9). This aimed to investigate their ability to efficiently identify genetic loci associated with pest resistance. The analysis revealed 33 significant QTLs associated with binary traits, involving 67 candidate genes, and 36 QTLs associated with continuous traits, encompassing 85 candidate genes (Supplementary Tables 4 and 5). Genes associated with continuous traits, as identified through GO enrichment analysis, were found to be primarily involved in the calcium ion binding process (*P* < 0.05). In contrast, the genes associated with binary traits did not exhibit significant enrichment in molecular functions, nor did the total 139 nonredundant candidate gene sets (Supplementary Table 6). Notably, all three gene sets detected a cluster related to involvement in protein kinase activity and protein phosphorylation, although the significance level was not sufficient (Supplementary Table 7). We identified *1-aminocyclopropane-1-carboxylate oxidase homolog 1* (*ACO*, *Vitvi008234*) and *sterol 3-beta-glucosyltransferase UGT80B1* (*TT15*, *Vitvi022534*) on chromosomes 5 and 12, respectively, associated with binary phenotype results, as well as *probable protein phosphatase 2C 14* (*PP2C*, *Vitvi000748*) on chromosome 1 linked to continuous phenotype results, all of which have been previously implicated in pest resistance in plants (Fig. 3A). *Vitvi008234* and *Vitvi022534* are involved in the synthesis of ethylene and flavonoids (Supplementary Table 8), respectively. These two compounds play a significant role in influencing the plant’s defense against herbivore feeding [26, 27]. *Vitvi000748* encodes a PP2C-type phosphatase (Supplementary Table 9), PP2C has been identified as a negative regulator of protein kinase cascades activated in response to stress [28], and existing research has indicated that PP2C is involved in the plant’s defense response against herbivores [29]. In addition, we identified a strong signal (Chr16: 26.7–27.0 Mb) for continuous trait (snp_16_26926513, *P* = 1.71e−06). Plants possessing both pure and mutated loci demonstrate lower scores and diminished pest damage (Fig. 3B). The predominant locus among the three populations is characterized by heterozygous haplotypes, with a higher prevalence of homozygous mutant haplotypes in the EU-Table and an increased frequency of homozygous mutant haplotypes in the EA-Table (Fig. 3C). Additionally, the genome-wide analysis of genetic differentiation (*F*_ST_) and genetic diversity (π) indicates an absence of selection events in this region (Fig. 3D). The linkage disequilibrium (LD) analysis highlights a significant linkage in the promoter region of *CDPK-RELATED KINASE 3* (*CRK3*, *Vitvi031045*) (Fig. 3E). The gene *Vitvi031045* encodes a protein kinase related to Ca^2+^/calmodulin-dependent kinases and functions in calmodulin-mediated signaling pathways [30] (Supplementary Table 7). Previous studies have demonstrated that CRK overexpression and knockout mutants exhibit increased and decreased defense traits against SLF [31]. Furthermore, it was found that the transcription of *CRK3* is positively regulated by JA and abscisic acid (ABA) signals, particularly under herbivory-induced stimulation [31]. It plays a pivotal role in modulating plant defense responses by phosphorylating and activating *WRKY14*, and enhancing the defense response through the upregulation of *PDF1.2* [31].

### Integration of GWAS and RNA-seq identified pest-resistant candidate genes

**Fig. 4:**
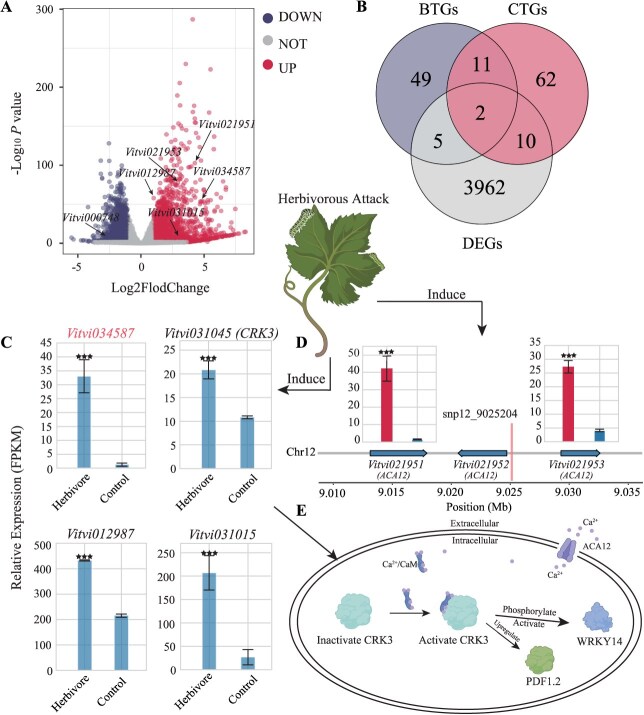
**Transcriptomic analyses in grapevine under herbivorous and normal conditions. A**, Volcano plot showing differential gene expression, with upregulated genes on the right and downregulated genes on the left, and GWAS-identified genes marked by arrows. **B**, Venn diagram illustrating the overlap between genes associated with binary and continuous phenotypes, as well as DEGs. **C**, Gene expression patterns of the four pest-defense genes that were derived from the intersection of GWAS and RNA-seq results. **D**, Three genes in the region of chromosome 12 (9.010–9.035 Mb) encode the same protein and were detected by GWAS (snp12_9025204). Bar charts showing gene expression levels, with herbivory conditions on the left and controls on the right, and significant differences marked by asterisks(★: *P* < 0.05, ★★★: *P* < 0.001). **E**, The functions performed by two insect-resistant genes within the cell are elucidated, along with their potential interactions.

In order to verify the accuracy of the DL model predictions and GWAS analysis results, we conducted transcriptomic analyses. Under assault by the two-spotted spider mite, *Tetranychus urticae*, another herbivorous insect, we identified a total of 3979 differentially expressed genes (DEGs) with *P* ≤ 0.05 and a fold change greater than 2. Subsequently, a total of 2010 upregulated genes and 1969 downregulated genes were identified (Fig. 4A and Supplementary Table 10). GO enrichment analysis revealed these genes are significantly involved in signal transduction, defense responses, and metabolic processes, consistent with previous studies [32] (Supplementary Table 11). It is noteworthy that DEGs were also found to be enriched in a cluster with the highest significance, specifically in protein phosphorylation and protein kinase activity (protein serine/threonine/tyrosine kinase activity) (*P* < 0.05), in line with our GWAS analyses. We observed a convergence between the DEGs uncovered through RNA-seq and the genes associated with binary traits (BTGs) and continuous traits (CTGs). There were 13 genes common to both traits, with 7 genes shared between BTGs and DEGs, and 12 genes shared between CTGs and DEGs, respectively (Fig. 4B). Notably, all three analyses identified the presence of two genes (*Vitvi034587* and *Vitvi031046*). *Vitvi031046 (SAUR32)* encodes an auxin-responsive protein and *Vitvi034587 (ERF110),* known as *ethylene-responsive transcription factor* (*ERF110, Vitvi034587*), which was significantly induced under conditions of herbivore attack (Fig. 4C). *Vitvi012987* (*ATHB-6*) and *Vitvi031015* (*DMR6*) constituted the intersection of BTGs and DEGs, playing roles in the regulation of the ABA signaling pathway and the synthesis of defensive oxygenase, respectively [33, 34]. They were also significantly induced in expression, indicating their relevance to pest defense (Fig. 4C). Within the intersection of CTGs and DEGs, two key genes have been identified (*Vitvi021951* and *Vitvi021953*). These genes are associated with continuous traits (snp_12_9025204) in GWAS and are strongly induced under herbivore attack. Additionally, it is noteworthy that these three genes are tandemly linked (Chr12: 9.01–9.04 Mb) and are encoded by the same gene, *calcium-transporting ATPase 12, ACA12* (Fig. 4D). *ACA12* encodes a calcium ion pump localized on the plasma membrane of plant cells (Fig. 4E). Mutants of *ACA12* have been demonstrated to exhibit extreme sensitivity to herbivorous attack by *Spodoptera littoralis*. This sensitivity manifests as a significant increase in leaf damage, accompanied by a decrease in intracellular calcium ion concentrations [35]. Simultaneously, *Vitvi031045* (previously mentioned in the GWAS results) was also found to be significantly induced (Fig. 4C and E). Analysis of integrated transcriptome data demonstrated the effectiveness of the identified pest resistance genes across both traits through the application of DL models.

### Improving genomic selection with the optimal ML model

**Fig. 5:**
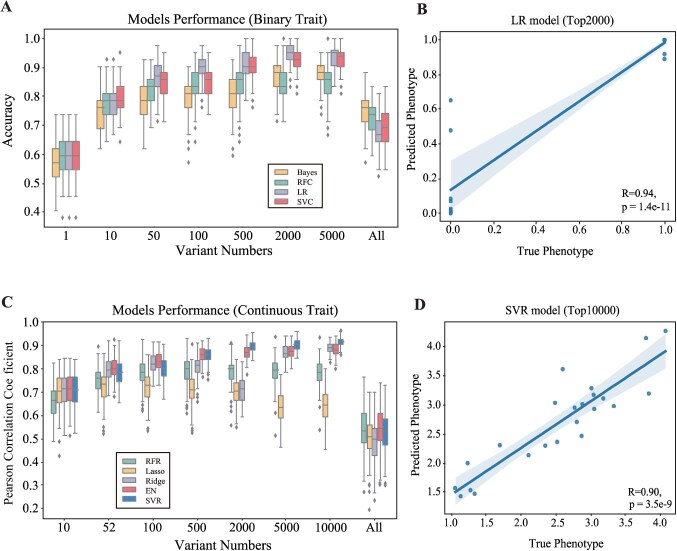
**ML-based GS of pest resistance in grapevine. A**, Cross-validation of the accuracy of the four classification models, using the average accuracy value as the accuracy indicator. **B**, Using the optimized model and variable count, predictions were made on the test set samples, revealing the correlation between the predicted binary phenotype and the true binary phenotype. **C**, Cross-validation of the accuracy of the five regression models, using the average correlation coefficient value as the accuracy indicator. **D**, Using the optimized model and variable count, predictions were made on the test set samples, revealing the correlation between the predicted continuous phenotype and the true continuous phenotype.

The outcomes from GWAS with binary and continuous traits were utilized to construct genomic selection (GS) models based on ML methodologies, respectively. Among four evaluated models, including Logistic Regression, Support Vector Classifier (SVC), Random Forest Classifier (RFC), and Naïve Bayes Classifier models for the binary trait prediction (Supplementary Table 12). The performance of the RFC model with different variations consistently fell short, achieving an accuracy that did not exceed 85%. Naive Bayes outperformed RFC but fell behind the other two models, achieving a maximum average accuracy of 87.6%. As the number of variations increased, the performance of the SVC model consistently improved. Beyond 500 variations, the average accuracy showed a gradual plateauing, reaching its peak at 93% when the number of variations reached 5000. The logistic regression model was deemed more suitable for this modeling task. In contrast to the SVC model, logistic regression reached its peak accuracy at 2000 variations (94.5%) and exhibited a declining trend in accuracy with 5000 variations (Fig. 5A). We performed the final validation of the test set by combining the logistic regression model with the appropriate number of variations. The accuracy achieved was 95.7%, and a significant correlation of 0.94 (*P* = 1.4e−11) was observed between the predicted values and the actual phenotypes (Fig. 5B). In the prediction of continuous traits, we compared various linear models (Lasso, Ridge, ElasticNet) and ML models, including Random Forest Regression (RFR) and Support Vector Regression (SVR) (Supplementary Table 13). Among these models, the Lasso model exhibited a poor performance, with the highest average correlation coefficient not exceeding 0.72. The accuracy of the RFR showed negligible variation, reaching a maximum of only 0.79. ElasticNet performed well, achieving a high accuracy of 0.88, and demonstrated stable performance beyond 500 variations. According to the results, the Ridge model exhibited instability, with a sudden increase in accuracy after finding an appropriate number of variations, reaching a peak of 0.89 at 10000 variations. SVR outperformed all other models, showing a steady increase in performance. Beyond 2000 variations, its performance gradually stabilized, reaching a peak accuracy of 0.92 (Fig. 5C). Based on the results of CV, we selected SVR and applied the identified 10 000 single-nucleotide polymorphisms (SNPs) to predict the continuous phenotype in the test set. Ultimately, we obtained a significant correlation coefficient of 0.90 (*P* = 3.5e−9) between the predicted and actual phenotypes, demonstrating the accuracy of the predictions (Fig. 5D). Additionally, all Support Vector Machine models (SVC, SVR) undergoes CV to select the optimal kernel function that represents the corresponding optimal results (Supplementary Fig. 10).

## Discussion

Our study implemented cutting-edge breeding methodologies to develop grapevine cultivars with enhanced resistance to pests, leveraging recent breakthroughs in DL-based plant phenomics and ML-based GS. Specifically, we employed GWAS to map the genetic basis of pest resistance in grapes [36, 37]. Subsequently, GS is utilized to selectively breed individuals harboring pest-resistant alleles. Although binary phenotypes are commonly deployed in pest resistance analyses, they overlook subtle-effect genes in GWAS. Conversely, the continuous phenotype generated by DCCN-PDS enables the detection of minor-effect genes, thereby offering a more comprehensive understanding of pest resistance mechanisms. We integrated DL-based binary and continuous pest damage traits, leading to the identification of 139 candidate genes endowing pest resistance, facilitating ML-based genomic prediction of pest damage traits in grapevine breeding.

### The genetic basis of pest resistance in grapevine

This study aimed to identify genes associated with resistance to *S. litura* (Fabricius) (SLF) in grape. Since there was no prior knowledge about the impact of identified genes on SLF resistance, both binary and continuous damage scores were used as phenotypes in GWAS. Our findings revealed 139 candidate genes related to insect resistance that participate in various signaling pathways, including JA, SA, ethylene, and others. Notably, the gene *CRK3* showed a significant association with both types of phenotypes, indicating its crucial role in SLF resistance. Previous research has also suggested *CRK3*’s involvement in plant defense responses, as overexpressing *CRK3* in Arabidopsis enhances defense properties, while knockout mutants exhibited reduced expression levels of the defense gene *PDF1.2* [31]. Additionally, *CRK3* has been demonstrated to phosphorylate tyrosine residues of herbivory-responsive regulators like *WRKY14*, highlighting its participation in the defense response against SLF [38]. It is worth mentioning that *ACA12*, another gene associated with continuous damage scores, encodes a calcium ion pump protein localized to the plasma membrane of plant cells [39]. Research has shown that *ACA12* mutants are more susceptible to SLF attacks compared to wild-type plants, suggesting that *ACA12* may play a vital role in providing a calcium ion-enriched environment for *CRK3* [35]. This interaction could potentially activate *CRK3* or other defense proteins reliant on calcium ion signaling, initiating subsequent defense responses [31]. Nonetheless, further investigation is necessary to validate this hypothesis.

It is interesting to note that genes conferring resistance to diseases tend to display higher levels of polymorphism compared to other genomic regions, likely due to the selective pressure imposed by rapidly evolving pathogen populations [39]. Unfortunately, only a minuscule proportion (approximately 0.1%) of the available biodiversity in resistance loci controlling pests and pathogens has been leveraged in commercial crop varieties. To develop more pest-resistant crops, integrating diverse genetic resources from local varieties, wild ancestors, and wild relatives into breeding programs is essential [40]. These resources possess distinct combinations of resistance traits shaped by evolutionary pressures, rendering them valuable sources of novel genetic material [41]. By capitalizing on the innate resistance of host plants through these resources, breed selection becomes more effective, and germplasm utilization is significantly improved, ultimately facilitating crop improvement.

### DL/ML-based methods for grapevine genomic breeding

Compared to traditional methods, DL enables more efficient, objective, and accurate assessment of phenotypic variation in plants [42, 43]. Commonly, the extent of leaf damage is manually assessed by visual inspection; therefore, it is slow, labor consuming, and subjective to the judgment of individual assessors. Our DL models can objectively assess one leaf image within 0.03 to 0.04 seconds and measure its damage condition with high accuracy, up to 0.95 for binary phenotype and with a high correlation, up to 0.94 for continuous scores, compared to the manual labeling. Therefore, the sample size can be easily scaled up if required. Our DL models improve the feasibility to establish large-scale phenomics and empower the molecular breeding of grapes [44]. Noteworthily, in insect pest research, DL is mostly applied to identify the pest types based on the morphological features of the pest itself [45], hence not designed to evaluate the pest resistance of the plants. To precisely determine the resistance feature of plants themselves, it is required to measure the damage conditions in various infestation parts of the different plant samples, for example damage to air potato tubers [46], leaf damage [47], dead heart tillers and whitehead-panicles [48], root damage [49] and so on. In our study, the major pest in grape cultivation is SLF. Its larvae mainly consume grape leaves and can cause complete defoliation [13].

Generally, the damage to plants caused by the disease and pest are often considered together in most studies using popular DL models designed for image recognition [50]. It is difficult to disengage the disease and pest resistant capabilities from the same plant. To avoid this, our study is deliberately based on disease-free plants and our model is then capable of measuring the damage (mild vs. severe) caused by the pest only. We selected VGG16, which outperformed the other widely used image recognition models, including AlexNet, ResNet50, ResNet101, InceptionV3, and DenseNet121, as our DL model backbone. We added four residual networks based on the backbone to generate both binary and continuous damage scores. For GS of pest-resistant grapevines, we compared multiple ML methods including random forest, naive Bayes, support vector machines, elastic net and other models, to avoid empirical bias in model selection. We finally selected logistic regression (95.7% in accuracy) and SVR (0.90 in correlation coefficient) as models for precisely predicting binary and continuous phenotypes, respectively.

Pest resistance is a polygenic complex trait, with many small-effect variants contributing jointly to phenotypic variation. Moreover, the predictive ability of significant SNPs may be limited by population-specific differences and genomic landscapes (e.g., genomic linkage, 3D structure and epigenomic modifications). In summary, filtering markers through the GWAS approach can enhance predictive performance. Future studies could further explore how combining functional annotations or biological prior knowledge could optimize marker selection to improve prediction stability and transferability. Furthermore, although this study demonstrates the effectiveness of ML methods in predicting pest resistance traits, we acknowledge that traditional genome-wide selection methods, such as genomic best linear unbiased prediction (GBLUP), remain widely adopted benchmark methods in plant breeding. As highlighted in the review by Tong and Nikoloski [51], GBLUP and related mixed linear models have shown predictive ability for various agronomic traits across multiple crop species. The focus on alternative ML methods is due to their ability to capture nonadditive genetic effects and complex genotype–phenotype relationships, which may be particularly important for pest resistance traits. Future research could involve direct comparisons with GBLUP and more recent DL-based genome selection methods, such as deep neural network for genomic prediction (DNNGP), to comprehensively assess the relative advantages of different modeling frameworks.

In conclusion, our results indicate that DL is an effective technique for phenotypic evaluation with high speed and low biases. It lays the foundation for large-scale phenomics data establishment. By integrating genomics, phenomics, and transcriptomics with a high-accuracy, optimally selected machine learning model, we identified the polygenic architecture of grapevine pest resistance, thus advancing efficient breeding strategies. The phenomics evaluation model and GS model would be integrated to automated breeding platform. The breeding strategy will be greatly advanced by the integration of mass breeding data and artificial intelligence algorithms.

## Materials and methods

### Viticulture conditions and pest infestations outbreak

A total of 231 grape varieties were introduced from the Grape Germplasm Repository of the Zhengzhou Fruit Research Institute of the Chinese Academy of Agricultural Sciences and planted in the Grape Germplasm Resource Nursery of the Shenzhen Institute of Agricultural Genomics (AGIS) of the Chinese Academy of Agricultural Sciences in November 2021. All experimental varieties were 2-year-old cuttings from standard vines and were cultivated in pots under greenhouse conditions, with each pot including at least three branches to represent given accession. All plants were subject to standard management practices prior to the experiment, including cultivation, irrigation, fertilization, pruning, and disease control (Supplementary Fig. 6). To examine the impact of different grape varieties on pest damage, we randomly arranged different grape varieties in a greenhouse. We performed a ~20-day experiment, when a natural outbreak of tobacco cutworm occurred in our greenhouse during July–August 2022, with 9–10 larvae per plant. The pesticide was sprayed after the experiment ended (Fig. 2). We randomly took pictures for 6 to 10 leaves for each accession in early September 2022 to represent the pest damage of the sample.

### Collection and preprocessing of image datasets for model training

The images were captured with the high-quality built-in camera of a Sony ZV-1 II, which features advanced sensor technology and image processing. We captured thousands of grape leaf samples from different locations, including wild and farm environments (https://github.com/zhouyflab/Pest-Resistance), showcasing a range of colors, backgrounds, and pest damage levels. To focus on the main part of the leaves and eliminate excess pixels, we trained a YOLO object detection model for leaf detection [52]. This allowed us to accurately and automatically crop leaf bounding boxes from thousands of images (Fig. 1C). Although the model has the capability to detect wormholes as well, they were not considered in this study, and the weights file for this YOLO model can be available for download (https://github.com/zhouyflab/Pest-Resistance). The object detection model achieved an impressive mAP of 0.91 after 300 epochs of training (Supplementary Fig. 11). Leave images were resized to 224 × 224 pixels using the Python Imaging Library (Version: 9.4.0), and pixel values were normalized to the 0–1 range for consistent process and model input. To increase data diversity and generalization, we used the ImageDataGenerator class from Keras (Version: 2.4.0) to apply random transformations and augmentations, including random rotation up to 45 degrees, shifts of 15% in width and height, horizontal flipping, and random zooming with a range of 50% [53].

### Image labeling and dataset splitting process

To train the binary classification model, a total of 1810 images were labeled using one-hot encoding: leaves with over 25% pest damage were labeled as 1, while those with 25% or less damage were labeled as 0. The dataset was randomly split into a 4:1 ratio, with 80% of the data (1448 images) used for CV (training and validation), and the remaining 20% (362 images) used as the test set. For the regression model, a total of 2620 images were used: 439 images for testing, while the remaining 2181 images for training and CV process, split in a 4:1 ratio. All the images are labeled from 1 to 5 based on damage severity: 1 for up to 20% damage area, increasing by 1 for each additional 20%, with 5 for damage exceeding 80%. Note that all labels, including binary classification labels and regression labels, are determined among three raters to reduce subjective bias (Supplementary Fig. 12).

### Modeling process of six binary task models

All DL models for grape leaf recognition were built using the Keras library with TensorFlow (Version: 2.4.0) backend [54]. Modeling was performed on a Linux platform with a GeForce RTX 3090 GPU, equipped with 24 GB of memory.

For the binary-classification modeling procedures, a comparative analysis of the performance of the AlexNet [55], VGG16 [56], ResNet50, ResNet101 [57], InceptionV3 [58], and DenseNet121 [59] models was conducted for the classification of pest damage into two categories: mild (0) and severe (1). The network architecture was modified with a focus on the fully connected and output layer. Each model featured two fully connected layers with 4096 neurons and ReLu activation, and a dropout rate of 0.5 was applied to enhance robustness [60]. The output layer used a sigmoid activation function to match the binary cross-entropy loss. To prevent overfitting, early stopping was implemented to terminate the training process when it no longer resulted in meaningful improvements. Models varied in complexity, resulting in different patience values. For example, AlexNet was tolerated for 20 epochs, while DenseNet121 required 40 epochs. We evaluated these models using 5-fold CV and calculated accuracy and F1-score metrics with scikit-learn (Version 1.0.2). Random seeds were applied to ensure consistent data splitting during the CV process across all models. All the six models share the same hyperparameter range and settings. We only tuned the batch size (32, 64, 128) and learning rate (0.0001, 0.001, 0.01), while the number of epochs (200) was not specifically adjusted due to early stopping. Then the Adam optimizer was used for all models [61, 62]. Transfer learning is applied to enhance model performance [63], with the weights automatically downloaded through the Keras library.

### DCNN-PDS modeling procedures

The regression model generates a continuous value to assess the pest-damage score (PDS) of grape leaves. The model was built using the Keras library [64] (Version: 2.4.0) and was executed on a Linux platform. The network architecture is constructed based on a combination of VGG16 [56] and residual networks [57]. VGG16 is renowned for its strong feature extraction capabilities. However, merely stacking networks to boost performance can lead to the loss of crucial image information and degrade the overall model [57]. To address this, we integrated residual networks. By adding four residual blocks after the VGG16 convolutional layer, we could stack deeper CNNs without worrying about network degradation. Each residual block contains two convolutional layers with 512, 256, 128, and 64 filters, respectively. A global average pooling (GAP) layer was then added after the last convolutional layer to reduce feature dimensions. Three fully connected (FC) layers with ReLU activations were appended behind the GAP layer for feature extraction and fusion, each with 1024 neurons. A dropout layer with a 0.3 rate followed each FC layer to reduce overfitting. Unlike the original VGG16, which used softmax for multiclassification, our model aims to solve a numerical regression problem for pest-damage scores [56]. Therefore, we used sigmoid activation in the final FC regression layer [65], with the output X5 serving as the pest damage score, corresponding to the manual label value (Fig. 1C).

In this study, the parameters of the convolutional layers of DCNN-PDS have been pretrained on ImageNet [66] and are frozen during the training process [67]. For this regression model, MSE was chosen as the loss function, and MAE was used as the performance metric to evaluate accuracy. Similarly, we fine-tuned the learning rate (0.0001,0.001,0.01) and batch size (32,64 128) through 5-fold CV with early stopping set to a tolerance of 35 epochs. The overall training process consisted of 300 epochs, after CV, we proceeded with formal model training and evaluated it on a new test set, keeping the optimal hyperparameters unchanged. To further assess the performance of the model, we utilized the Numpy library (Version: 1.19.5) to calculate the Pearson correlation coefficient [68] between the predicted values and the labels, aiming to determine their linear relationship.

### Grape phenotype calculation

Six to 10 images are available for each grape accession to determine its phenotype (https://github.com/zhouyflab/Pest-Resistance). To calculate the binary phenotype, indicating the severity or mild of pest damage, the model computes a probability value of the 0–1 classification for each image. Images with an average probability value below 0.5 are classified as mild pest damage, while those with an average probability value greater than 0.5 are classified as severe pest damage. For a more detailed evaluation of pest damage, the DCCN-PDS provides direct damage scores for multiple images. These scores are then averaged to obtain the final continuity phenotype value. The accurate scores provided by DCCN-PDS quantify the severity of pest damage on a continuous scale from 0–5, without dimensions. This approach offers a more objective alternative to manual measurement and provides data suitable for genetic analysis.

### Distribution statistics of pest phenotypes

The Shapiro–Wilk test was employed to assess the normality of the sample data. Specifically, the Shapiro test was utilized in *R* to compute the *W*-statistic and associated *P* value for various samples. A *P* value inferior to 0.05 signifies that the sample’s phenotypic distribution conforms to a normal distribution. In order to evaluate variance homogeneity on normally distributed data, the Bartlett test was performed. The test function in R was leveraged to determine the corresponding *P* value for each sample. If the *P* value surpasses 0.05, it may be inferred that the variance of the data exhibits homogeneity. We employed R’s Spearman and rcorr functions to calculate Spearman’s footrule distance and correlation, respectively. Spearman’s footrule is a nonparametric statistical method used to quantify the difference between two ranked sequences. It is defined as the sum of the absolute differences in positions for each element in the two lists, and its values range from 0 to n [2]/2 (where n represents the sample size). List A was based on the order of the phylogenetic tree, while List B was based on the degree of pest damage to different varieties. Furthermore, we generated 100 random orders for conducting a single-sample *t*-test.

### The GWAS analyses

In this study, paired-end resequencing reads were mapped to the *V. vinifera* reference genome PNT2T [69] using BWA-mem2 (Version: 2.2.1) with default parameters. Mapping results were converted into the BAM format and filtered for unmapped and nonunique reads using SAMtools (Version: 1.17). BWA alignment was conducted, and two functions ‘vc and joint’ of GTX (Version: 2.2.1) were applied to obtain SNP VCF files. To ensure the accuracy of the results, SNPs with a missing genotype frequency greater than 0.05 or a minor allele frequency less than 0.05 were excluded from analysis. Imputation was not performed. Populations were considered for their structure and cryptic relationships, and GEMMA [70] (Version: 0.98.3) was used to implement the GWAS of the standard linear mixed model:


\begin{align*}y=W\alpha + X\beta + \epsilon.\end{align*}

The whole-genome significance cutoff was defined using the adjusted Bonferroni test threshold, which was set as *P* < 1e−5.

### Transcriptomic analyses under herbivorous conditions

Our transcriptome data sets originated from the Sequence Read Archive accession SRP067967, which was sequenced for its transcriptome data on grape leaves under herbivorous exposure to two-spotted spider mite (*T. urticae*) [32]. The raw transcriptomic data was processed using fastq-dump [71] (Version: 2.11.0), converting it into paired-end fastq files. Quality control was performed using FastQC [72] (Version: 0.11.9) to assess data quality. Subsequently, trim-galore [73] software (Version: 0.6.7) was utilized to filter out adapter sequences and low-quality reads (–Stringency 3–phred33–q 25). The data was then aligned to the grape reference genome using STAR [74] software (Version: 2.7.10b), resulting in aligned BAM files. Quantification of gene expression was performed using featureCounts (Version: 2.0.1). For the final analysis, we employed DESeq2 [75] package (Version: 1.38.3) in RStudio to conduct differential gene expression analysis. Genes with a fold change greater than two and a *P* value less than 0.05 would be considered as DEGs. All GO enrichment analyses were conducted on the DAVID website (https://david.ncifcrf.gov/tools.jsp). Additionally, we employed materials from BioRender (https://app.biorender.com/) to construct mode pattern.

### The comparison of GS models based on the cross-validation

Regarding the severity of pest damage, two phenotypic descriptors were estimated. One was binary qualitative shape with the phenotype divided into two classes (‘mild’ vs. ‘severe’). The other one represented a quantitative measurement of the damage with the phenotype quantified into a continuous scale ranging from 0 to 5. For binary phenotype, Logistic Regression, SVC, RFC and Naive Bayes Classifier models were selected to enable comparisons of predictive accuracy. The prediction accuracy between different models was determined by the average accuracy. For continuous phenotype prediction, two ML models, SVR and RFR, and three regularization models, Lasso (L1 regularization), Ridge regression (L2 regularization), and ElasticNet (combining L1 and L2 regularization), were compared with each other. L1 and L2 regularization represent two types of marker effect estimation. The former, L1 regularization, is well suited for identifying and adapting to a few major QTLs that have significant effects on the phenotype; L2 regularization, also known as ridge regression, is effective in capturing the effects of many minor-effect QTLs. Pearson’s correlation coefficient between the predicted and the observed phenotypes was calculated as the prediction accuracy. The support vector machine (SVM) models were applied to predict both binary and continuous phenotypes. Additionally, SVM offers various kernel functions, including the linear kernel, polynomial kernel, and radial basis function kernel. These three kernel functions were also compared by the CV to select the optimal one that represented the best results for this model. All models utilized in GS were implemented using the scikit-learn Python library (Version: 1.0.2). Different models in two types of pest phenotypes were compared based on accuracy, and the optimal model was chosen.

### Stepwise variation selection based on GWAS results for optimal prediction

Regarding the results from GWAS, the sorting based on their *P* values and a stepwise selection of variations were performed to identify the optimal number of variations to construct a GS model. Before modeling, Plink (Version: 1.90b7) was used to filter variations with the parameters including window size set to 100, step size set to 50, and LD threshold set to 0.2 to reduce redundancy and minimize the impact of LD in the GS process. After filtering, a total of 7 359 942 variations were reduced to 313 961 variations, representing all the variations that participated in the modeling. Different numbers of variations in models with binary and continuous phenotypes were evaluated with cross-validation. With the binary phenotype, a total of 50 significant SNPs (*P* < 1e−5) were identified through GWAS. The variations with the numbers including 1, 10, 50 (all significant SNPs), 100, 500, 2000, 5000, and 313 961 (all variations) according to their sorted *P* values were selected for GS. Regarding the continuous phenotypes, a total of 52 significant SNPs (*P* < 1e−5) were identified. The numbers of variations including 10, 52 (all significant SNPs), 100, 500, 2000, 5000, 10 000, and 313 961 (all variations) were considered for prediction modeling. Ten percent of the samples’ phenotype data were reserved for testing, while the remaining 90% of the samples were split in a 4:1 ratio for modeling and validation. The optimal number of variations was evaluated based on the final modeling accuracy averaged over 100 iterations. Random seeds were set to ensure the training data consistency across these 100 modeling and evaluation iterations.

## Supplementary Material

Web_Material_uhaf128

## Data Availability

All resequencing data generated have been deposited in the NCBI database under the BioProject: PRJNA994294.

## Code availability

All scripts performed in this study are available on Github: https://github.com/zhouyflab/Pest-Resistance.
