# Supplementary material for: Deep learning empowers genomic selection of pest-resistant grapevine
Source: Hortic Res. 2025 May 7;12(8):uhaf128. doi: 10.1093/hr/uhaf128 (PMC12265469; doi:10.1093/hr/uhaf128)
Supplement: Web_Material_uhaf128 [file web_material_uhaf128.zip › 1_Supplementary Table1-13.pdf]

Supplementary Tables. Gan et al. (2024). Deep learning based genomic breeding of pest-resistant grapevine.

List of Tables	Title
**Table S1**	Performance of Six Classical Neural Networks in Cross validation
**Table S2**	Performance of DCNN-PDS in Cross validation
**Table S3**	Phenotypes and grouping of 231 grape varieties cultivated in greenhouses
**Table S4**	Significant regional loci discovered by binary traits in GWAS results
**Table S5**	Significant regional loci discovered by continuous traits in GWAS results
**Table S6**	GO functional enrichment analysis conducted for three gene sets
**Table S7**	GO enrichment clustering analysis results based on three gene sets
**Table S8**	Candidate gene of binary traits in GWAS results
**Table S9**	Candidate gene of continuous traits in GWAS results
**Table S10**	Upregulated and downregulated genes by Tetranyhus urticae
**Table S11**	GO enrichment of differentially expressed gene sets in transcriptome analysis
**Table S12**	The performance of genomic selection models with different SNPs in cross validation (binary phenotype)
**Table S13**	The performance of genomic selection models with different SNPs in cross validation (continuous phenotype)

Table. S.1. Performance of Six Classical Neural Networks

Model Name	Accuracy								F1								Model complexity
	cv1	cv2	cv3	cv4	cv5	Mean	sd	P	cv1	cv2	cv3	cv4	cv5	Mean	sd	P	
AlexNet	0.90	0.91	0.87	0.90	0.86	0.89	0.02	ab	0.90	0.90	0.87	0.90	0.84	0.88	0.02	ab	37315329
VGG16	0.89	0.91	0.90	0.90	0.91	0.90	0.00	a	0.90	0.89	0.90	0.90	0.90	0.90	0.00	a	134264641
ResNet50	0.87	0.87	0.87	0.90	0.85	0.87	0.01	b	0.87	0.83	0.87	0.90	0.85	0.86	0.02	b	451365889
ResNet101	0.85	0.89	0.88	0.88	0.85	0.87	0.01	b	0.85	0.85	0.87	0.88	0.85	0.86	0.01	b	470384129
InceptionV3	0.88	0.90	0.88	0.91	0.88	0.89	0.01	ab	0.89	0.88	0.88	0.92	0.88	0.89	0.01	ab	248273057
DenseNet121	0.88	0.89	0.86	0.90	0.87	0.88	0.01	ab	0.88	0.85	0.86	0.90	0.86	0.87	0.02	ab	229264257

Model Name	Test Sets	
	**Accuracy**	**F1**
AlexNet	0.876	0.868
VGG16	0.953	0.952
ResNet50	0.939	0.94
ResNet101	0.950	0.949
InceptionV3	0.920	0.916
DenseNet121	0.925	0.921

Table. S.2. Performance of DCNN-PDS in Cross validation

Cross-Validation	**MAE**	**MSE**
CV1	0.206493499	0.103421228
CV2	0.222956283	0.112112086
CV3	0.245080608	0.13402079
CV4	0.219771671	0.121531521
CV5	0.277494478	0.151570749
Mean	0.2343	0.1245

Table. S.3. Phenotypes and grouping of 231 grape varieties cultivated in greenhouses

ID	usage(table/wine)	Europe and America/Europe and Asia	binary phenotype	continuous phenotype
VV_116	table	Europe and Asia	severe(1)	3.326081912
VV_117	table	Europe and Asia	mild(0)	1.237140656
VV_118	table	Europe and Asia	mild(0)	2.396071196
VV_119	table	Europe and Asia	severe(1)	2.972805341
VV_120	table	Europe and America	severe(1)	4.484113216
VV_123	table	Europe and Asia	severe(1)	3.152928273
VV_124	table	Europe and America	mild(0)	1.034006119
VV_126	table	Europe and America	mild(0)	1.367179553
VV_128	table	Europe and Asia	mild(0)	1.827744683
VV_129	table	Europe and America	mild(0)	2.208755314
VV_130	wine	Europe and Asia	severe(1)	2.912342548
VV_131	table	Europe and America	mild(0)	1.065222144
VV_133	table	Europe and Asia	severe(1)	2.786969066
VV_136	table	Europe and Asia	severe(1)	3.832064311
VV_137	table	Europe and Asia	mild(0)	2.108248909
VV_258	table	Europe and America	severe(1)	2.640814567
VV_259	table	Europe and America	mild(0)	2.082942406
VV_260	table	Europe and America	mild(0)	1.915759166
VV_261	table	Europe and America	severe(1)	3.014800469
VV_262	table	Europe and America	mild(0)	1.827455163
VV_264	table	Europe and America	mild(0)	1.88957417
VV_266	table	Europe and America	mild(0)	1.047026396
VV_269	table	Europe and America	severe(1)	2.883549611
VV_271	table	Europe and America	severe(1)	3.406123161
VV_272	table	Europe and America	severe(1)	3.183132966
VV_273	table	Europe and America	mild(0)	1.477995912
VV_274	table	Europe and America	severe(1)	3.318817774
VV_275	table	Europe and America	mild(0)	2.078773816
VV_276	table	Europe and America	mild(0)	1.859123031
VV_277	table	Europe and America	mild(0)	1.06127739
VV_278	table	Europe and America	mild(0)	1.238183141
VV_280	table	Europe and America	severe(1)	4.173624635
VV_282	table	Europe and America	mild(0)	2.311575532
VV_283	table	Europe and America	mild(0)	2.431916396
VV_284	table	Europe and America	mild(0)	1.33568272
VV_285	table	Europe and America	mild(0)	2.235961676
VV_287	table	Europe and America	mild(0)	1.670177281
VV_288	table	Europe and America	mild(0)	1.579439104
VV_289	table	Europe and America	severe(1)	3.818575541
VV_290	table	Europe and America	mild(0)	1.70907263
VV_291	table	Europe and America	mild(0)	1.981978655
VV_292	table	Europe and America	mild(0)	1.92841359

ID	usage(table/wine)	Europe and America/Europe and Asia	binary phenotype	continuous phenotype
VV_293	table	Europe and America	severe(1)	3.419659853
VV_294	table	Europe and America	severe(1)	2.448877414
VV_296	table	Europe and America	mild(0)	1.205862085
VV_297	table	Europe and America	mild(0)	1.683758855
VV_298	table	Europe and America	severe(1)	2.878824234
VV_299	table	Europe and America	mild(0)	2.067542712
VV_300	table	Europe and America	mild(0)	1.459111651
VV_301	table	Europe and America	mild(0)	1.056115707
VV_303	table	Europe and America	mild(0)	1.286811352
VV_304	table	Europe and America	mild(0)	1.240658919
VV_305	table	Europe and America	severe(1)	2.798814495
VV_310	table	Europe and America	mild(0)	1.934646964
VV_311	table	Europe and America	mild(0)	1.283754309
VV_312	table	Europe and America	mild(0)	2.537267764
VV_313	table	Europe and America	severe(1)	3.120071769
VV_314	table	Europe and America	mild(0)	1.662545125
VV_315	table	Europe and America	mild(0)	1.080940048
VV_318	wine	Europe and Asia	severe(1)	4.061426004
VV_319	table	Europe and Asia	severe(1)	3.468955755
VV_321	table	Europe and America	mild(0)	2.856053511
VV_322	table	Europe and America	mild(0)	1.203740398
VV_323	table	Europe and America	mild(0)	1.607494354
VV_324	table	Europe and America	severe(1)	2.5441281
VV_326	table	Europe and America	mild(0)	1.674870571
VV_328	table	Europe and America	mild(0)	1.533480485
VV_329	table	Europe and America	mild(0)	1.773929159
VV_332	table	Europe and America	severe(1)	2.793280602
VV_334	table	Europe and America	mild(0)	2.349544088
VV_337	table	Europe and America	mild(0)	1.410556793
VV_339	table	Europe and America	mild(0)	1.953410824
VV_340	table	Europe and America	mild(0)	1.602388501
VV_341	table	Europe and America	severe(1)	3.884764671
VV_342	table	Europe and America	mild(0)	1.062199632
VV_344	table	Europe and Asia	severe(1)	3.801187992
VV_345	table	Europe and America	mild(0)	1.091579636
VV_346	table	Europe and America	severe(1)	3.264424404
VV_347	table	Europe and America	mild(0)	2.177103519
VV_350	table	Europe and America	mild(0)	1.85264643
VV_352	table	Europe and America	severe(1)	2.385908286
VV_354	table	Europe and America	mild(0)	1.395202676
VV_356	table	Europe and America	mild(0)	1.084275723
VV_357	table	Europe and America	mild(0)	1.439319571
VV_358	table	Europe and America	severe(1)	2.317218661

ID	usage(table/wine)	Europe and America/Europe and Asia	binary phenotype	continuous phenotype
VV_360	table	Europe and America	mild(0)	1.214596748
VV_361	table	Europe and America	mild(0)	1.316882173
VV_363	table	Europe and America	mild(0)	2.18894879
VV_364	table	Europe and America	mild(0)	2.193342487
VV_365	table	Europe and America	mild(0)	1.203295231
VV_366	table	Europe and America	mild(0)	2.801041524
VV_368	table	Europe and America	mild(0)	1.615747929
VV_369	table	Europe and America	severe(1)	2.945287466
VV_376	wine	Europe and Asia	severe(1)	2.426189343
VV_377	wine	Europe and Asia	severe(1)	3.786609491
VV_378	wine	Europe and Asia	severe(1)	3.37234354
VV_384	wine	Europe and Asia	mild(0)	1.861323595
VV_386	wine	Europe and Asia	severe(1)	3.65570116
VV_387	wine	Europe and Asia	severe(1)	2.916013002
VV_388	wine	Europe and Asia	severe(1)	2.874456485
VV_389	wine	Europe and Asia	severe(1)	2.084806204
VV_390	wine	Europe and Asia	severe(1)	2.954741955
VV_391	table	Europe and Asia	severe(1)	2.963211139
VV_392	wine	Europe and Asia	severe(1)	3.036816299
VV_393	wine	Europe and Asia	severe(1)	3.89903748
VV_396	wine	Europe and Asia	severe(1)	3.987045288
VV_397	table	Europe and Asia	severe(1)	3.485035499
VV_399	wine	Europe and Asia	severe(1)	4.33368206
VV_400	wine	Europe and Asia	severe(1)	3.153264205
VV_401	wine	Europe and Asia	severe(1)	2.962570985
VV_402	wine	Europe and Asia	mild(0)	3.015776952
VV_404	wine	Europe and Asia	severe(1)	3.037567616
VV_405	wine	Europe and Asia	severe(1)	2.349585931
VV_406	table	Europe and Asia	severe(1)	3.011780421
VV_407	table	Europe and Asia	severe(1)	3.324626684
VV_408	wine	Europe and Asia	severe(1)	3.12104706
VV_409	wine	Europe and Asia	severe(1)	3.625582457
VV_410	table	Europe and Asia	severe(1)	4.174600244
VV_411	wine	Europe and Asia	severe(1)	4.019950867
VV_412	wine	Europe and Asia	severe(1)	3.015728474
VV_413	wine	Europe and Asia	mild(0)	2.199775736
VV_414	wine	Europe and Asia	severe(1)	2.661127329
VV_418	wine	Europe and Asia	severe(1)	3.049901843
VV_419	wine	Europe and Asia	mild(0)	1.586729288
VV_420	table	Europe and Asia	severe(1)	3.567489942
VV_421	wine	Europe and Asia	mild(0)	1.621431669
VV_422	wine	Europe and Asia	mild(0)	2.228954315
VV_425	table	Europe and Asia	mild(0)	2.488489151

ID	usage(table/wine)	Europe and America/Europe and Asia	binary phenotype	continuous phenotype
VV_426	wine	Europe and Asia	severe(1)	3.214734316
VV_427	wine	Europe and Asia	mild(0)	2.420867761
VV_428	wine	Europe and Asia	severe(1)	3.132063309
VV_429	table	Europe and Asia	severe(1)	2.447240472
VV_433	table	Europe and Asia	severe(1)	3.645800591
VV_434	wine	Europe and Asia	severe(1)	3.0953269
VV_435	table	Europe and Asia	severe(1)	3.930037737
VV_436	wine	Europe and Asia	severe(1)	3.354551236
VV_437	wine	Europe and Asia	severe(1)	4.341817697
VV_441	wine	Europe and Asia	severe(1)	3.231540362
VV_442	wine	Europe and Asia	mild(0)	1.798410505
VV_444	wine	Europe and Asia	severe(1)	3.268937767
VV_445	wine	Europe and Asia	mild(0)	2.313761155
VV_446	wine	Europe and Asia	severe(1)	3.992145459
VV_447	table	Europe and Asia	severe(1)	2.92320919
VV_449	wine	Europe and Asia	severe(1)	3.417360067
VV_451	wine	Europe and Asia	mild(0)	2.259010315
VV_452	wine	Europe and Asia	severe(1)	3.125683029
VV_453	wine	Europe and Asia	mild(0)	1.51148659
VV_456	wine	Europe and Asia	severe(1)	2.98145625
VV_457	table	Europe and Asia	severe(1)	3.825743437
VV_458	wine	Europe and Asia	severe(1)	2.615231037
VV_459	table	Europe and Asia	severe(1)	4.019579013
VV_460	wine	Europe and Asia	mild(0)	2.273874919
VV_463	wine	Europe and Asia	mild(0)	2.95369792
VV_464	wine	Europe and Asia	mild(0)	1.914487219
VV_465	table	Europe and Asia	mild(0)	2.236280362
VV_470	table	Europe and Asia	severe(1)	3.085087935
VV_471	table	Europe and Asia	mild(0)	2.007945061
VV_473	table	Europe and America	mild(0)	1.899216215
VV_475	table	Europe and Asia	severe(1)	3.964715004
VV_477	table	Europe and Asia	severe(1)	4.145817439
VV_482	table	Europe and Asia	severe(1)	3.489840229
VV_483	table	Europe and Asia	severe(1)	2.685116649
VV_484	wine	Europe and Asia	severe(1)	3.843798637
VV_490	table	Europe and Asia	mild(0)	1.827373226
VV_492	table	Europe and Asia	severe(1)	3.225362937
VV_493	table	Europe and Asia	mild(0)	2.122792323
VV_494	table	Europe and Asia	severe(1)	4.274713198
VV_495	table	Europe and Asia	mild(0)	1.999107917
VV_496	table	Europe and Asia	mild(0)	2.901817958
VV_498	wine	Europe and Asia	severe(1)	3.47029376
VV_499	wine	Europe and Asia	severe(1)	3.099366665

ID	usage(table/wine)	Europe and America/Europe and Asia	binary phenotype	continuous phenotype
VV_500	wine	Europe and Asia	mild(0)	1.934715331
VV_501	table	Europe and Asia	severe(1)	4.335581938
VV_503	wine	Europe and Asia	mild(0)	2.47855703
VV_504	wine	Europe and Asia	severe(1)	4.174375852
VV_505	table	Europe and Asia	mild(0)	1.70469137
VV_506	table	Europe and Asia	severe(1)	3.464440743
VV_509	table	Europe and Asia	severe(1)	2.883146564
VV_512	table	Europe and Asia	severe(1)	3.908085903
VV_513	wine	Europe and Asia	mild(0)	1.801957846
VV_514	wine	Europe and Asia	severe(1)	3.327371359
VV_515	table	Europe and Asia	mild(0)	1.144932508
VV_516	table	Europe and Asia	mild(0)	1.492141207
VV_517	wine	Europe and Asia	mild(0)	2.52165397
VV_518	wine	Europe and Asia	severe(1)	4.026337147
VV_519	table	Europe and Asia	mild(0)	1.716742913
VV_520	table	Europe and Asia	severe(1)	2.872635603
VV_523	wine	Europe and Asia	severe(1)	3.936837912
VV_524	wine	Europe and Asia	severe(1)	3.85697333
VV_525	table	Europe and Asia	severe(1)	3.80722332
VV_526	table	Europe and Asia	severe(1)	2.604045093
VV_527	table	Europe and Asia	severe(1)	4.085120122
VV_530	table	Europe and Asia	severe(1)	3.845262925
VV_531	table	Europe and Asia	severe(1)	2.765826523
VV_532	table	Europe and Asia	mild(0)	2.659858525
VV_534	table	Europe and Asia	severe(1)	3.098465443
VV_541	wine	Europe and Asia	severe(1)	3.374690384
VV_543	wine	Europe and Asia	severe(1)	3.497093995
VV_544	wine	Europe and Asia	mild(0)	2.953739007
VV_546	wine	Europe and Asia	severe(1)	4.504898071
VV_548	wine	Europe and Asia	severe(1)	2.667685986
VV_550	wine	Europe and Asia	mild(0)	1.858692169
VV_551	wine	Europe and Asia	severe(1)	2.751123945
VV_553	wine	Europe and Asia	mild(0)	2.337804159
VV_554	table	Europe and Asia	severe(1)	4.019770304
VV_556	table	Europe and Asia	severe(1)	3.353704929
VV_557	table	Europe and Asia	mild(0)	1.654237151
VV_558	table	Europe and Asia	severe(1)	4.272048155
VV_559	wine	Europe and Asia	severe(1)	4.362481117
VV_560	table	Europe and Asia	severe(1)	3.198367993
VV_562	table	Europe and Asia	severe(1)	4.071468989
VV_563	table	Europe and Asia	severe(1)	2.893718163
VV_564	table	Europe and Asia	severe(1)	3.650581439
VV_567	table	Europe and Asia	severe(1)	4.058786273

ID	usage(table/wine)	Europe and America/Europe and Asia	binary phenotype	continuous phenotype
VV_568	wine	Europe and Asia	severe(1)	3.970906973
VV_569	table	Europe and Asia	severe(1)	3.235196829
VV_572	wine	Europe and Asia	mild(0)	1.560743093
VV_574	table	Europe and Asia	severe(1)	3.159094731
VV_575	table	Europe and Asia	severe(1)	3.447566191
VV_576	table	Europe and Asia	severe(1)	4.234920263
VV_577	table	Europe and Asia	severe(1)	2.939416091
VV_579	table	Europe and Asia	severe(1)	3.766700745
VV_580	table	Europe and Asia	severe(1)	4.262254715
VV_581	table	Europe and Asia	severe(1)	3.336512208
VV_582	table	Europe and Asia	severe(1)	3.512672126
VV_583	table	Europe and Asia	severe(1)	3.930253088
VV_585	table	Europe and Asia	severe(1)	3.424648126
VV_586	table	Europe and Asia	severe(1)	3.521377961
VV_587	table	Europe and Asia	severe(1)	2.998151143
VV_588	wine	Europe and Asia	severe(1)	3.37744991
VV_589	wine	Europe and Asia	severe(1)	3.607762257

Table. S.4. Significant regional loci discovered by binary traits in GWAS results

Region name	Chromosome	Region start	Region end	significant Marker	marker number	PVE
B1	1	6215878	6215878	1__6215878	1	0.057577349
B2	1	7072630	7085061	1__7085061	2	0.061161798
B3	2	4326566	4326566	2__4326566	1	0.061358753
B4	3	5336131	5336131	3__5336131	1	0.058957448
B5	3	10984780	10984780	3__10984780	1	0.06365185
B6	5	3383716	3383716	5__3383716	1	0.057725885
B7	5	10486946	10486947	5__10486947	2	0.071794341
B8	5	11632691	11632691	5__11632691	1	0.059397323
B9	5	11688349	11688349	5__11688349	1	0.05845695
B10	5	11800584	11800584	5__11800584	1	0.059947948
B11	5	11838995	11838995	5__11838995	1	0.060277386
B12	5	12057369	12057369	5__12057369	1	0.060309299
B13	5	15097679	15097679	5__15097679	1	0.059022325
B14	7	24094299	24094299	7__24094299	1	0.057201399
B15	9	2471518	2471518	9__2471518	1	0.057520763
B16	9	23464586	23464586	9__23464586	1	0.058893899
B17	12	16880863	16880863	12__16880863	1	0.060087385
B18	12	19044874	19044882	12__19044874	3	0.063104049
B19	12	19193680	19193680	12__19193680	1	0.061323298
B20	13	19088940	19088940	13__19088940	1	0.070941686
B21	15	13852232	13854473	15__13854473	2	0.069624844
B22	15	17583207	17583207	15__17583207	1	0.057169826
B23	15	19025588	19025588	15__19025588	1	0.073511126
B24	16	6714717	6714717	16__6714717	1	0.063356168
B25	16	6740526	6749217	16__6740526	2	0.065376771
B26	16	8313004	8363565	16__8337656	6	0.065004223
B27	16	26734387	26734387	16__26734387	1	0.059193238
B28	16	26941200	26950465	16__26950400	5	0.077163192
B29	18	11267451	11267451	18__11267451	1	0.057526982
B30	18	18854294	18854298	18__18854294	2	0.064400813
B31	18	23770517	23770549	18__23770549	2	0.062636854
B32	19	20208670	20208670	19__20208670	1	0.057639252
B33	19	26634049	26634049	19__26634049	1	0.06412175

Table. S.5. Significant regional loci discovered by continuous traits in GWAS results

Region name	Chromosome	Region start	Region end	significant Marker	marker number	PVE
C1	1	7205826	7207198	1__7205876	4	0.085469257
C2	1	7265000	7265000	1__7265000	1	0.060205532
C3	1	7438060	7438060	1__7438060	1	0.05763055
C4	1	8400760	8400760	1__8400760	1	0.059570257
C5	2	3322879	3322879	2__3322879	1	0.066742163
C6	2	3368525	3368525	2__3368525	1	0.058038148
C7	2	3732244	3732244	2__3732244	1	0.058746698
C8	2	4134797	4134797	2__4134797	1	0.058580247
C9	2	4299287	4299287	2__4299287	1	0.064537353
C10	2	4483015	4483015	2__4483015	1	0.061954948
C11	2	4753428	4753428	2__4753428	1	0.062203978
C12	3	5336253	5336253	3__5336253	1	0.06615323
C13	3	10984780	10984824	3__10984780	2	0.073010777
C14	4	7623664	7623664	4__7623664	1	0.059566553
C15	5	15120052	15120052	5__15120052	1	0.060402071
C16	6	17089795	17089795	6__17089795	1	0.059961071
C17	6	17306466	17306466	6__17306466	1	0.062223065
C18	7	567554	567554	7__567554	1	0.057429096
C19	7	21370441	21370441	7__21370441	1	0.060922819
C20	7	21673810	21673810	7__21673810	1	0.057804315
C21	8	17616186	17616186	8__17616186	1	0.058890614
C22	8	17668179	17668179	8__17668179	1	0.059576776
C23	9	23235754	23235754	9__23235754	1	0.057294833
C24	9	24269754	24269754	9__24269754	1	0.059390145
C25	12	7799955	7799955	12__7799955	1	0.05729557
C26	12	9025204	9025204	12__9025204	1	0.059274675
C27	13	19088940	19088940	13__19088940	1	0.08495708
C28	15	13854473	13854473	15__13854473	1	0.057393908
C29	15	19025588	19025588	15__19025588	1	0.074618265
C30	15	22509825	22509825	15__22509825	1	0.058419022
C31	16	26883188	26883188	16__26883188	1	0.057642726
C32	16	26914866	26928314	16__26926513	12	0.066389125
C33	16	26950326	26950360	16__26950360	2	0.064469766
C34	18	12766119	12766119	18__12766119	1	0.059720297
C35	18	13212556	13212556	18__13212556	1	0.06317863
C36	18	23770549	23770549	18__23770549	1	0.06317863

Table. S.6. GO functional enrichment analysis conducted for three gene sets

**BTG(Binary Trait Gene)**												
Category	Term	Count	%	PValue	Genes	List Total	Pop Hits	Pop Total	Fold Enrichmer	Bonferroni	Benjamini	FDR
GOTERM_CC_DIRECT	GO:0099503~secretory vesicle	4	8.333333333	0.001970017	Q9FMH8, Q94	37	180	25998	15.61441441	0.079485313	0.08274072	0.082740716
GOTERM_BP_DIRECT	GO:0006811~ion transport	3	6.25	0.003795809	Q84MB3, Q9S	34	65	23397	31.76063348	0.356704883	0.43651809	0.436518087
GOTERM_BP_DIRECT	GO:0010150~leaf senescence	4	8.333333333	0.007885678	F8S296, Q9FL	34	291	23397	9.459066101	0.600828618	0.4534265	0.453426503
GOTERM_CC_DIRECT	GO:0005737~cytoplasm	14	29.16666667	0.020012715	Q8L5R3, O230	37	5264	25998	1.868746406	0.572182525	0.42026701	0.420267013
GOTERM_CC_DIRECT	GO:0005576~extracellular region	10	20.83333333	0.033723517	Q9FY79, Q6T	37	3321	25998	2.115774311	0.763266775	0.47212924	0.472129239
GOTERM_BP_DIRECT	GO:0009813~flavonoid biosynthetic process	2	4.166666667	0.064246028	Q9FLV0, Q9X	34	47	23397	29.28285357	0.999548399	1	1
GOTERM_BP_DIRECT	GO:0042742~defense response to bacterium	5	10.41666667	0.06588029	F8S296, Q9FL	34	1091	23397	3.153744541	0.999631282	1	1
GOTERM_CC_DIRECT	GO:0000325~plant-type vacuole	4	8.333333333	0.085459997	Q9XIG1, Q94	37	753	25998	3.732529342	0.976529968	0.89732997	0.897329966
GOTERM_MF_DIRECT	GO:0009931~calcium-dependent protein serine/threonine kinase activit	2	4.166666667	0.086749548	Q9ZUZ2	33	55	19438	21.41928375	0.993201012	1	1
GOTERM_MF_DIRECT	GO:0004683~calmodulin-dependent protein kinase activity	2	4.166666667	0.086749548	Q9ZUZ2	33	55	19438	21.41928375	0.993201012	1	1
GOTERM_BP_DIRECT	GO:0009733~response to auxin	3	6.25	0.088708046	Q9ZUZ3, Q9S	34	354	23397	5.831754736	0.999979094	1	1
GOTERM_MF_DIRECT	GO:0005515~protein binding	12	25	0.094448955	Q8S3D2, Q84	33	4451	19438	1.58803946	0.995732127	1	1
**CTG(Continuous Trait Gene)**												
Category	Term	Count	%	PValue	Genes	List Total	Pop Hits	Pop Total	Fold Enrichmer	Bonferroni	Benjamini	FDR
GOTERM_MF_DIRECT	GO:0005509~calcium ion binding	6	10	3.68E-04	Q42529, O818	44	280	19438	9.466558442	0.029023164	0.02981534	0.029815336
GOTERM_BP_DIRECT	GO:0048366~leaf development	5	8.333333333	6.75E-04	O81831, Q9M	47	203	23397	12.26129337	0.10362073	0.10867971	0.108679708
GOTERM_BP_DIRECT	GO:0045740~positive regulation of DNA replication	2	3.333333333	0.005886855	O81831	47	3	23397	331.8723404	0.615760247	0.47389179	0.473891793
GOTERM_MF_DIRECT	GO:0033984~indole-3-glycerol-phosphate lyase activity	2	3.333333333	0.006622155	Q42529	44	3	19438	294.5151515	0.412297436	0.26819728	0.268197278
GOTERM_CC_DIRECT	GO:0016020~membrane	8	13.33333333	0.007052762	O82752, Q7EB	48	1258	25998	3.444356121	0.312793713	0.17958439	0.169419233
GOTERM_CC_DIRECT	GO:0009506~plasmodesma	7	11.66666667	0.009343808	Q94CH6, Q9M	48	1010	25998	3.753836634	0.391981484	0.17958439	0.169419233
GOTERM_CC_DIRECT	GO:0000325~plant-type vacuole	6	10	0.011306302	Q42529, Q9M	48	753	25998	4.315737052	0.452639329	0.17958439	0.169419233
GOTERM_MF_DIRECT	GO:0016298~lipase activity	3	5	0.013866309	Q94CH6, Q9L	44	81	19438	16.36195286	0.672762	0.31154832	0.311548322
GOTERM_MF_DIRECT	GO:0004834~tryptophan synthase activity	2	3.333333333	0.015385102	Q42529	44	7	19438	126.2207792	0.710724132	0.31154832	0.311548322
GOTERM_CC_DIRECT	GO:0005886~plasma membrane	13	21.66666667	0.015797319	O82752, Q7EB	48	3405	25998	2.06787812	0.56998777	0.17958439	0.169419233
GOTERM_CC_DIRECT	GO:0005635~nuclear envelope	3	5	0.016941923	Q42529, Q9FJ	48	110	25998	14.77159091	0.595706917	0.17958439	0.169419233
GOTERM_BP_DIRECT	GO:0045736~negative regulation of cyclin-dependent protein serine/thr	2	3.333333333	0.019491322	O81831	47	10	23397	99.56170213	0.958777571	0.62289902	0.622899023
GOTERM_BP_DIRECT	GO:0010150~leaf senescence	4	6.666666667	0.019499534	F8S296, Q9LY	47	291	23397	6.842728669	0.958833468	0.62289902	0.622899023
GOTERM_MF_DIRECT	GO:0004861~cyclin-dependent protein serine/threonine kinase inhibito	2	3.333333333	0.019738217	O81831	44	9	19438	98.17171717	0.797060903	0.31975911	0.319759108
GOTERM_MF_DIRECT	GO:0005516~calmodulin binding	4	6.666666667	0.025917488	Q9LY77, Q9M	44	289	19438	6.114501416	0.877634294	0.34988608	0.349886084
GOTERM_BP_DIRECT	GO:0014070~response to organic cyclic compound	4	6.666666667	0.028284257	O82752, Q94C	47	336	23397	5.926291793	0.990420194	0.62289902	0.622899023
GOTERM_BP_DIRECT	GO:0009733~response to auxin	4	6.666666667	0.032299126	O81831, Q9ZU	47	354	23397	5.624954922	0.995101495	0.62289902	0.622899023
GOTERM_BP_DIRECT	GO:0052544~defense response by callose deposition in cell wall	2	3.333333333	0.036715415	Q42529	47	19	23397	52.40089586	0.997665215	0.62289902	0.622899023
GOTERM_CC_DIRECT	GO:0005773~vacuole	4	6.666666667	0.039048689	Q9M1S3, Q38	48	415	25998	5.220481928	0.878891861	0.34368186	0.324228171
GOTERM_CC_DIRECT	GO:0009570~chloroplast stroma	5	8.333333333	0.045391944	Q42529, Q9M	48	746	25998	3.63019437	0.914743001	0.34368186	0.324228171
GOTERM_BP_DIRECT	GO:0048367~shoot system development	4	6.666666667	0.053236804	O81831, Q9LI	47	433	23397	4.598692939	0.999858388	0.62289902	0.622899023
GOTERM_BP_DIRECT	GO:0016042~lipid catabolic process	3	5	0.053282765	Q94CH6, Q9L	47	189	23397	7.901722391	0.999859497	0.62289902	0.622899023
GOTERM_BP_DIRECT	GO:0009737~response to abscisic acid	6	10	0.053295919	Q9M1S3, O81	47	1045	23397	2.858230683	0.999859813	0.62289902	0.622899023
GOTERM_BP_DIRECT	GO:0046777~protein autophosphorylation	3	5	0.055296778	Q9ZUZ2, F4H	47	193	23397	7.737956124	0.999900493	0.62289902	0.622899023
GOTERM_BP_DIRECT	GO:0009556~microsporogenesis	2	3.333333333	0.055506232	Q0WWX5, O4	47	29	23397	34.33162142	0.999904004	0.62289902	0.622899023
GOTERM_BP_DIRECT	GO:0000162~tryptophan biosynthetic process	2	3.333333333	0.055506232	Q42529	47	29	23397	34.33162142	0.999904004	0.62289902	0.622899023
GOTERM_BP_DIRECT	GO:0000027~ribosomal large subunit assembly	2	3.333333333	0.057365472	Q9M1S3	47	30	23397	33.18723404	0.999930238	0.62289902	0.622899023
GOTERM_BP_DIRECT	GO:0034613~cellular protein localization	2	3.333333333	0.059221131	Q9FJB8	47	31	23397	32.11667811	0.999949303	0.62289902	0.622899023
GOTERM_BP_DIRECT	GO:0042742~defense response to bacterium	6	10	0.061903009	F8S296, O8275	47	1091	23397	2.737718665	0.999968075	0.62289902	0.622899023
GOTERM_CC_DIRECT	GO:0009505~plant-type cell wall	4	6.666666667	0.070068409	Q94CH6, Q9M	48	528	25998	4.103219697	0.978723359	0.43470979	0.410103575
GOTERM_BP_DIRECT	GO:0009851~auxin biosynthetic process	2	3.333333333	0.072111056	Q42529	47	38	23397	26.20044793	0.999994576	0.68293412	0.682934115
GOTERM_BP_DIRECT	GO:0009790~embryo development	2	3.333333333	0.077582272	Q8L7W5, Q9L	47	41	23397	24.28334198	0.999997919	0.69393032	0.69393032
GOTERM_MF_DIRECT	GO:0004672~protein kinase activity	5	8.333333333	0.079363374	Q9LJY0, Q9M	44	738	19438	2.993040158	0.998660139	0.73991132	0.739911319
GOTERM_MF_DIRECT	GO:0004712~protein serine/threonine/tyrosine kinase activity	5	8.333333333	0.080596557	Q9LJY0, C0LC	44	742	19438	2.97690517	0.998796378	0.73991132	0.739911319
GOTERM_CC_DIRECT	GO:0005788~endoplasmic reticulum lumen	2	3.333333333	0.081598268	Q42529	48	47	25998	23.04787234	0.989016701	0.43470979	0.410103575
GOTERM_CC_DIRECT	GO:0005634~nucleus	26	43.33333333	0.082020715	F8S296, Q4252	48	10940	25998	1.287225777	0.989281286	0.43470979	0.410103575
GOTERM_BP_DIRECT	GO:0050832~defense response to fungus	6	10	0.091756799	F8S296, O8275	47	1227	23397	2.434271446	0.999999831	0.73287737	0.732877371
GOTERM_BP_DIRECT	GO:1990961~drug transmembrane export	2	3.333333333	0.093807252	O82752, Q389	47	50	23397	19.91234043	0.999999883	0.73287737	0.732877371
GOTERM_MF_DIRECT	GO:0005515~protein binding	15	25	0.095371034	Q42529, Q8L7	44	4451	19438	1.488786994	0.999670664	0.73991132	0.739911319

GOTERM_BP_DIRECT	GO:0009630~gravitropism	2	3.333333333	0.095592701	Q42529	47	51	23397	19.52190238	0.999999915	0.73287737	0.732877371
**ALL(Combine Binary and Continuous Traits Genes)**												
Category	Term	Count	%	PValue	Genes	List Total	Pop Hits	Pop Total	Fold Enrichmer	Bonferroni	Benjamini	FDR
GOTERM_CC_DIRECT	GO:0099503~secretory vesicle	6	6.18556701	1.65E-04	Q42529, Q9FM	75	180	25998	11.55466667	0.009997973	0.00858024	0.008158259
GOTERM_CC_DIRECT	GO:0000325~plant-type vacuole	10	10.30927835	2.81E-04	Q42529, Q9M1	75	753	25998	4.603452855	0.017016446	0.00858024	0.008158259
GOTERM_CC_DIRECT	GO:0009506~plasmodesma	10	10.30927835	0.002234495	Q8L5R3, Q94C	75	1010	25998	3.432079208	0.127555887	0.04543472	0.043200228
GOTERM_MF_DIRECT	GO:0005509~calcium ion binding	6	6.18556701	0.002977309	Q42529, O818	69	280	19438	6.036645963	0.273158789	0.32154936	0.321549365
GOTERM_BP_DIRECT	GO:0048366~leaf development	5	5.154639175	0.003396717	O81831, Q9MA	72	203	23397	8.003899836	0.531752049	0.4139771	0.412112338
GOTERM_BP_DIRECT	GO:0009733~response to auxin	6	6.18556701	0.004437333	O81831, Q9ZU	72	354	23397	5.507768362	0.629064562	0.4139771	0.412112338
GOTERM_BP_DIRECT	GO:0042742~defense response to bacterium	10	10.30927835	0.005594285	F8S296, O8275	72	1091	23397	2.978536511	0.713789625	0.4139771	0.412112338
GOTERM_BP_DIRECT	GO:0045740~positive regulation of DNA replication	2	2.06185567	0.00907652	O81831	72	3	23397	216.6388889	0.869097911	0.50374686	0.501477729
GOTERM_MF_DIRECT	GO:0033984~indole-3-glycerol-phosphate lyase activity	2	2.06185567	0.010458772	Q42529	69	3	19438	187.8067633	0.675341633	0.56477366	0.564773663
GOTERM_BP_DIRECT	GO:0010150~leaf senescence	5	5.154639175	0.011867117	F8S296, Q9FL	72	291	23397	5.583476518	0.930205132	0.52689999	0.524526569
GOTERM_BP_DIRECT	GO:0006811~ion transport	3	3.092783505	0.016696685	Q84MB3, Q9S	72	65	23397	14.99807692	0.97659475	0.61777733	0.614994548
GOTERM_CC_DIRECT	GO:0009505~plant-type cell wall	6	6.18556701	0.017351402	Q94CH6, Q6N	75	528	25998	3.939090909	0.656210538	0.26460889	0.251595334
GOTERM_MF_DIRECT	GO:0004834~tryptophan synthase activity	2	2.06185567	0.02423631	Q42529	69	7	19438	80.48861284	0.927576773	0.61589588	0.615895878
GOTERM_CC_DIRECT	GO:0005886~plasma membrane	17	17.5257732	0.028292538	Q7EB72, Q6NI	75	3405	25998	1.730654919	0.826352796	0.31230738	0.296948
GOTERM_BP_DIRECT	GO:0045736~negative regulation of cyclin-dependent protein serine/thr	2	2.06185567	0.029940397	O81831	72	10	23397	64.99166667	0.998862248	0.94953831	0.945261115
GOTERM_CC_DIRECT	GO:0005773~vacuole	5	5.154639175	0.030718759	Q9M1S3, Q9FI	75	415	25998	4.176385542	0.850913514	0.31230738	0.296948
GOTERM_MF_DIRECT	GO:0004861~cyclin-dependent protein serine/threonine kinase inhibitor	2	2.06185567	0.031054028	O81831	69	9	19438	62.60225443	0.965797873	0.61589588	0.615895878
GOTERM_MF_DIRECT	GO:0016298~lipase activity	3	3.092783505	0.032722536	Q94CH6, Q9LI	69	81	19438	10.43370907	0.971557717	0.61589588	0.615895878
GOTERM_MF_DIRECT	GO:0004190~aspartic-type endopeptidase activity	3	3.092783505	0.034216438	Q6NNI8, Q94H	69	83	19438	10.18229439	0.975893198	0.61589588	0.615895878
GOTERM_CC_DIRECT	GO:0005635~nuclear envelope	3	3.092783505	0.039332712	Q42529, Q9FJI	75	110	25998	9.453818182	0.913512538	0.34275649	0.325899617
GOTERM_MF_DIRECT	GO:0005515~protein binding	23	23.71134021	0.047123132	Q42529, Q8L7	69	4451	19438	1.455702838	0.994286057	0.7270426	0.727042605
GOTERM_BP_DIRECT	GO:0052544~defense response by callose deposition in cell wall	2	2.06185567	0.056130045	Q42529	72	19	23397	34.20614035	0.999997457	1	1
GOTERM_CC_DIRECT	GO:0048046~apoplast	5	5.154639175	0.065214398	Q94CH6, Q9FY	75	532	25998	3.257894737	0.983653195	0.45087212	0.42869808
GOTERM_CC_DIRECT	GO:0016020~membrane	8	8.24742268	0.066522116	O82752, Q7EB	75	1258	25998	2.204387917	0.984991198	0.45087212	0.42869808
GOTERM_BP_DIRECT	GO:0050832~defense response to fungus	8	8.24742268	0.078081792	F8S296, O8275	72	1227	23397	2.11871774	0.999999987	1	1
GOTERM_CC_DIRECT	GO:0022625~cytosolic large ribosomal subunit	3	3.092783505	0.078758224	Q8VYF1, Q9M	75	163	25998	6.379877301	0.993288941	0.46579076	0.442883016
GOTERM_MF_DIRECT	GO:0005516~calmodulin binding	4	4.12371134	0.080626293	Q9LY77, Q9M	69	289	19438	3.899102352	0.999875934	1	1
GOTERM_BP_DIRECT	GO:0014070~response to organic cyclic compound	4	4.12371134	0.0823254	O82752, Q94C	72	336	23397	3.868551587	0.999999995	1	1
GOTERM_CC_DIRECT	GO:0005576~extracellular region	15	15.46391753	0.083995055	Q9FY79, Q6NI	75	3321	25998	1.56567299	0.995260125	0.46579076	0.442883016
GOTERM_BP_DIRECT	GO:0009556~microsporogenesis	2	2.06185567	0.084412545	Q0WWX5, O4	72	29	23397	22.41091954	0.999999997	1	1
GOTERM_BP_DIRECT	GO:0000162~tryptophan biosynthetic process	2	2.06185567	0.084412545	Q42529	72	29	23397	22.41091954	0.999999997	1	1
GOTERM_BP_DIRECT	GO:0000027~ribosomal large subunit assembly	2	2.06185567	0.087194414	Q9M1S3	72	30	23397	21.66388889	0.999999999	1	1
GOTERM_BP_DIRECT	GO:0034613~cellular protein localization	2	2.06185567	0.089967949	Q9FJB8	72	31	23397	20.96505376	0.999999999	1	1
GOTERM_CC_DIRECT	GO:0005737~cytoplasm	21	21.64948454	0.098155868	Q8L5R3, O230	75	5264	25998	1.38287234	0.998167599	0.498959	0.47442003

Table. S.7. GO enrichment clustering analysis results based on three gene sets

BTG(Binary Trait Gene)

Annotation Cluster 1 **Enrichment Score: 0.34877944084685825**

Category	Term	Count	%	PValue	Genes	List Total	Pop Hits	Pop Total	Fold Enrichmer	Bonferroni	Benjamini	FDR
GOTERM_CC_DIRECT	GO:0005886~plasma membrane	7	14.58333333	0.330202388	Q6NNI8,	37	3405	25998	1.444505298	0.999999951	1	1
GOTERM_MF_DIRECT	GO:0004672~protein kinase activity	3	6.25	0.344295194	O65924, (	33	738	19438	2.394432126	1	1	1
GOTERM_MF_DIRECT	GO:0004712~protein serine/threonine/tyrosine kinase activity	3	6.25	0.346722608	O65924, (	33	742	19438	2.381524136	1	1	1
GOTERM_BP_DIRECT	GO:0006468~protein phosphorylation	3	6.25	0.362674859	O65924, (	34	897	23397	2.30149518	1	1	1
GOTERM_MF_DIRECT	GO:0004674~protein serine/threonine kinase activity	3	6.25	0.412273272	O65924, (	33	852	19438	2.074050363	1	1	1
GOTERM_MF_DIRECT	GO:0005524~ATP binding	5	10.41666667	0.620743676	Q84WV1	33	2552	19438	1.154056236	1	1	1
GOTERM_CC_DIRECT	GO:0016021~integral component of membrane	5	10.41666667	0.989089629	Q9SA38,	37	6506	25998	0.540000499	1	1	1

CTG(Continuous Trait Gene)

Annotation Cluster 1 **Enrichment Score: 1.1261688986368372**

Category	Term	Count	%	PValue	Genes	List Total	Pop Hits	Pop Total	Fold Enrichmer	Bonferroni	Benjamini	FDR
GOTERM_MF_DIRECT	GO:0016298~lipase activity	3	5	0.013866309	Q94CH6,	44	81	19438	16.36195286	0.672762	0.311548322	0.311548322
GOTERM_BP_DIRECT	GO:0016042~lipid catabolic process	3	5	0.053282765	Q94CH6,	47	189	23397	7.901722391	0.999859497	0.622899023	0.622899023
GOTERM_CC_DIRECT	GO:0005576~extracellular region	7	11.66666667	0.566168464	Q94CH6,	48	3321	25998	1.141636555	1	1	0.961538462

Annotation Cluster 2 Enrichment Score: 0.7729668567517273

Category	Term	Count	%	PValue	Genes	List Total	Pop Hits	Pop Total	Fold Enrichmer	Bonferroni	Benjamini	FDR
GOTERM_MF_DIRECT	GO:0004672~protein kinase activity	5	8.333333333	0.079363374	Q9LJY0,	44	738	19438	2.993040158	0.998660139	0.739911319	0.739911319
GOTERM_MF_DIRECT	GO:0004712~protein serine/threonine/tyrosine kinase activity	5	8.333333333	0.080596557	Q9LJY0,	44	742	19438	2.97690517	0.998796378	0.739911319	0.739911319
GOTERM_MF_DIRECT	GO:0004674~protein serine/threonine kinase activity	5	8.333333333	0.118181828	Q9LJY0,	44	852	19438	2.592562953	0.99995731	0.739911319	0.739911319
GOTERM_MF_DIRECT	GO:0016301~kinase activity	4	6.666666667	0.154897139	Q9LJY0,	44	616	19438	2.868654073	0.999998578	0.89619059	0.89619059
GOTERM_MF_DIRECT	GO:0005524~ATP binding	9	15	0.195796929	Q9LJY0,	44	2552	19438	1.557975919	0.999999973	1	1
GOTERM_BP_DIRECT	GO:0006468~protein phosphorylation	4	6.666666667	0.258285466	Q9LJY0,	47	897	23397	2.219881876	1	1	1
GOTERM_CC_DIRECT	GO:0016021~integral component of membrane	12	20	0.655836295	O82752, (	48	6506	25998	0.999000922	1	1	0.961538462

ALL(Combine Binary and Continuous Traits Genes)

Annotation Cluster 1 **Enrichment Score: 0.6231552483680011**

Category	Term	Count	%	PValue	Genes	List Total	Pop Hits	Pop Total	Fold Enrichmer	Bonferroni	Benjamini	FDR
GOTERM_MF_DIRECT	GO:0004672~protein kinase activity	6	6.18556701	0.115767731	Q9LJY0,	69	738	19438	2.290326382	0.999998083	1	1
GOTERM_MF_DIRECT	GO:0004712~protein serine/threonine/tyrosine kinase activity	6	6.18556701	0.117721575	Q9LJY0,	69	742	19438	2.277979609	0.999998487	1	1
GOTERM_MF_DIRECT	GO:0016301~kinase activity	5	5.154639175	0.168894447	Q9LJY0,	69	616	19438	2.286608319	0.999999997	1	1
GOTERM_MF_DIRECT	GO:0004674~protein serine/threonine kinase activity	6	6.18556701	0.177282304	Q9LJY0,	69	852	19438	1.98387426	0.999999999	1	1
GOTERM_BP_DIRECT	GO:0006468~protein phosphorylation	5	5.154639175	0.289415367	Q9LJY0,	72	897	23397	1.811361947	1	1	1
GOTERM_MF_DIRECT	GO:0005524~ATP binding	11	11.34020619	0.402097843	Q9LJY0,	69	2552	19438	1.214267866	1	1	1
GOTERM_CC_DIRECT	GO:0016021~integral component of membrane	15	15.46391753	0.914819869	Q8L7W5,	75	6506	25998	0.799200738	1	1	0.966666667

Annotation Cluster 2 **Enrichment Score: 0.45841204573535654**

Category	Term	Count	%	PValue	Genes	List Total	Pop Hits	Pop Total	Fold Enrichmer	Bonferroni	Benjamini	FDR
GOTERM_CC_DIRECT	GO:0022625~cytosolic large ribosomal subunit	3	3.092783505	0.078758224	Q8VYF1,	75	163	25998	6.379877301	0.993288941	0.465790758	0.442883016
GOTERM_MF_DIRECT	GO:0003735~structural constituent of ribosome	4	4.12371134	0.18792908	Q8VYF1,	69	427	19438	2.638970913	1	1	1
GOTERM_CC_DIRECT	GO:0005840~ribosome	3	3.092783505	0.265997184	Q8VYF1,	75	353	25998	2.945949008	0.999999994	0.846712509	0.80507091
GOTERM_CC_DIRECT	GO:0005730~nucleolus	3	3.092783505	0.46230554	Q8VYF1,	75	546	25998	1.904615385	1	1	0.966666667
GOTERM_BP_DIRECT	GO:0006412~translation	4	4.12371134	0.61015698	Q8VYF1,	72	1030	23397	1.26197411	1	1	1
GOTERM_MF_DIRECT	GO:0003729~mRNA binding	4	4.12371134	0.730467873	Q8VYF1,	69	1070	19438	1.053122037	1	1	1
GOTERM_MF_DIRECT	GO:0003723~RNA binding	4	4.12371134	0.762038538	Q8VYF1,	69	1126	19438	1.000746518	1	1	1

Annotation Cluster 3 **Enrichment Score: 0.3253493709043792**

Category	Term	Count	%	PValue	Genes	List Total	Pop Hits	Pop Total	Fold Enrichmer	Bonferroni	Benjamini	FDR
GOTERM_MF_DIRECT	GO:0046983~protein dimerization activity	3	3.092783505	0.359737736	Q8S3D2,	69	360	19438	2.347584541	1	1	1
GOTERM_BP_DIRECT	GO:0006355~regulation of transcription, DNA-templated	7	7.216494845	0.536281761	Q8LAU9,	72	1928	23397	1.179827974	1	1	1
GOTERM_MF_DIRECT	GO:0003700~transcription factor activity, sequence-specific DNA binding	7	7.216494845	0.547737989	Q8LAU9,	69	1691	19438	1.166156721	1	1	1

Table. S.8. Candidate gene of binary traits in GWAS results

q	GeneID	Chromosome	Trait	PFAM	PFAM Protein Function	IPR	IPR Protein Function2	Go_ID	Swissport	gene_name
1	Vitvi000648	1	B1	PF00248	Aldo/keto reductase family	IPR023210	NADP-dependent oxidoreductase domain	-	Q9SQ64	COR2
2	Vitvi000649	1	B1	PF02519	Auxin responsive protein	IPR003676	Small auxin-up RNA	GO:0009733	Q9SGU2	SAUR71 At1g56150 F14G9.23 T6H22.5
3	Vitvi000650	1	B1	PF00248	Aldo/keto reductase family	IPR023210	NADP-dependent oxidoreductase domain	-	Q9SQ64	COR2
4	Vitvi000651	1	B1	PF00248	Aldo/keto reductase family	IPR023210	NADP-dependent oxidoreductase domain	-	Q9SQ64	COR2
5	Vitvi000729	1	B2	PF15862	Coilin N-terminus	IPR031722	Coilin, N-terminal domain	-	Q8RWK8	COIL At1g13030 F3F19.5
6	Vitvi000730	1	B2	PF01165	Ribosomal protein S21	IPR001911	Ribosomal protein S21	GO:0003735|GO:	-	-
7	Vitvi000731	1	B2	PF10250	GDP-fucose protein O-fuc	IPR019378	GDP-fucose protein O-fucosyltransferase	-	Q9LIN9	PAGR OFUT26 At3g26370 F20C19.9
8	Vitvi000732	1	B2	PF13966	zinc-binding in reverse tran	IPR026960	Reverse transcriptase zinc-binding domain	-	-	-
9	Vitvi002506	2	B3	PF01758	Sodium Bile acid symporte	IPR002657	Bile acid:sodium symporter/arsenical resistance protein Acr3	GO:0016020	B8BDK4	BASS5 OsI_32074
10	Vitvi002507	2	B3	Coil	Coil	-	-	-	Q55720	sll0608
11	Vitvi002508	2	B3	PF00078	Reverse transcriptase (RN	IPR000477	Reverse transcriptase domain	-	P92555	AtMg01250; At2g07697
12	Vitvi002509	2	B3	Coil	Coil	-	-	-	F4IG60	ZPR1 At2g45450 F4L23.4
13	Vitvi002510	2	B3	PF01428	AN1-like Zinc finger	IPR000058	Zinc finger, AN1-type	GO:0008270	Q6NNI8	SAP1 At1g12440 F5O11.17
14	Vitvi004218	3	B4	Coil	Coil	-	-	-	F8S296	ATG2 APG2 PEUP1 At3g19190 MVI11.10/MVI11.11
15	Vitvi004651	3	B5	-	-	-	-	-	-	-
16	Vitvi004652	3	B5	PF00118	TCP-1/cpn60 chaperonin f	IPR002423	Chaperonin Cpn60/GroEL/TCP-1 family	GO:0005524|GO:	Q84WV1	CCT3 At5g26360 F9D12.18
17	Vitvi007599	5	B6	PF00481	Protein phosphatase 2C	IPR001932	PPM-type phosphatase domain	GO:0016791	Q9LUS8	At3g16560 MDC8.3
18	Vitvi007600	5	B6	PF01411	tRNA synthetases class II (	IPR018164	Alanyl-tRNA synthetase, class IIc, N-terminal	GO:0000166|GO:	O57734	alaXL PH1969
19	Vitvi007601	5	B6	PF01249	Ribosomal protein S21e	IPR001931	Ribosomal protein S21e	GO:0003735|GO:	Q3E902	RPS21C At5g27700 T1G16.30
20	Vitvi007602	5	B6	PF05498	Rapid ALkalinization Facto	IPR008801	Rapid ALkalinization Factor	-	Q8L9P8	RALFL33 At4g15800 dl3940c FCAALL.206
21	Vitvi008234	5	B7	Coil	Coil	-	-	-	Q84MB3	At1g06620 F12K11.24 F12K11.6
22	Vitvi008307	5	B8	PF00320	GATA zinc finger	IPR000679	Zinc finger, GATA-type	GO:0006355|GO:	Q8LAU9	GATA1 At3g24050 F14O13.24
23	Vitvi008310	5	B10	PF05938	Plant self-incompatibility p	IPR010264	Plant self-incompatibility S1	-	-	-
24	Vitvi008311	5	B11	PF14868	Domain of unknown functi	IPR027902	Protein of unknown function DUF4487	-	-	-
25	Vitvi008319	5	B12	PF14008	Iron/zinc purple acid phosp	IPR025733	Iron/zinc purple acid phosphatase-like C-terminal domain	-	Q6TPH1	PAP23 AT3 At4g13700 F18A5.90
26	Vitvi008320	5	B12	PF00248	Aldo/keto reductase family	IPR023210	NADP-dependent oxidoreductase domain	-	O23016	KAB1 KV-BETA1 At1g04690 T1G11.6
27	Vitvi008419	5	B13	PF14111	Domain of unknown functi	IPR025558	Domain of unknown function DUF4283	-	-	-
28	Vitvi008420	5	B13	PF01494	FAD binding domain	IPR002938	FAD-binding domain	GO:0071949	P25535	ubiI visC b2906 JW2874
29	Vitvi012987	7	B14	PF02183	Homeobox associated leuci	IPR003106	Leucine zipper, homeobox-associated	GO:0006355|GO:	P46668	ATHB-6 At2g22430 F14M13.17
30	Vitvi016011	9	B15	Coil	Coil	-	-	-	Q8S3D2	BHLH87 EN121 At3g21330 MHC9.1
31	Vitvi016012	9	B15	PF00069	Protein kinase domain	IPR000719	Protein kinase domain	GO:0004672|GO:	O65924	At2g19210 F27F23.1
32	Vitvi016013	9	B15	PF12819	Malectin-like domain	IPR024788	Malectin-like domain	-	O65924	At2g19210 F27F23.1
33	Vitvi017361	9	B16	PF13249	Squalene-hopene cyclase N	IPR032697	Squalene cyclase, N-terminal	-	O82140	OSCPNY1 bAS beta-AS PNY1
34	Vitvi022404	12	B17	-	-	-	-	-	-	-
35	Vitvi022534	12	B18	-	-	-	-	-	Q9XIG1	UGT80B1 TT15 At1g43620 T10P12.7
36	Vitvi022542	12	B19	PF04842	Plant protein of unknown f	IPR006927	Protein of unknown function DUF639	-	-	-
37	Vitvi022547	12	B19	PF00314	Thaumatin family	IPR001938	Thaumatin family	-	Q5DWG1	
38	Vitvi022548	12	B19	PF01151	GNS1/SUR4 family	IPR002076	ELO family	GO:0009922|GO:	Q86JM5	DDB_G0272012
39	Vitvi024368	13	B20	PF00078	Reverse transcriptase (RN	IPR000477	Reverse transcriptase domain	-	P92555	AtMg01250; At2g07697
40	Vitvi024369	13	B20	PF10453	Nuclear fragile X mental re	IPR019496	Nuclear fragile X mental retardation-interacting protein 1, conserved doma	-	-	-
41	Vitvi028358	15	B21	PF04570	zinc-finger of the FCS-type	IPR007650	Zf-FLZ domain	-	O80506	FLZ3 DUF581-9 At2g44670 F16B22.16
42	Vitvi028613	15	B22	-	-	-	-	-	-	-
43	Vitvi028614	15	B22	Coil	Coil	-	-	-	F4KGE8	GIL1 At5g58960 K19M22.15
44	Vitvi028615	15	B22	PF04043	Plant invertase/pectin meth	IPR006501	Pectinesterase inhibitor domain	GO:0004857	P17407	
45	Vitvi028616	15	B22	PF12612	Tubulin folding cofactor D	IPR022577	Tubulin-specific chaperone D, C-terminal	-	Q8L5R3	TFCD CHO EMB133 TBCD TTN1 At3g60740 T4C21.150
46	Vitvi028769	15	B23	PF00847	AP2 domain	IPR001471	AP2/ERF domain	GO:0003700|GO:	P92966	RS41 RSP41 At5g52040 MSG15.12
47	Vitvi028770	15	B23	Coil	Coil	-	-	-	-	-
48	Vitvi028771	15	B23	PF05648	Peroxisomal biogenesis fac	IPR008733	Peroxisomal biogenesis factor 11	GO:0005779|GO:	Q9LQ73	PEX11C PEX11-1 At1g01820 T1N6.24
49	Vitvi029743	16	B24	Coil	Coil	-	-	-	P93604	LRK10
50	Vitvi029746	16	B25	PF05057	Putative serine esterase (DI	IPR007751	Domain of unknown function DUF676, lipase-like	-	P53118	ROG1 YGL144C
51	Vitvi029837	16	B26	PF04506	Rft protein	IPR007594	RFT1	GO:0006488|GO:	Q54IV7	rft1 DDB_G0288491
52	Vitvi029838	16	B26	PF14111	Domain of unknown functi	IPR025558	Domain of unknown function DUF4283	-	-	-
53	Vitvi029839	16	B26	PF13966	zinc-binding in reverse tran	IPR026960	Reverse transcriptase zinc-binding domain	-	-	-
54	Vitvi029840	16	B26	PF00078	Reverse transcriptase (RN	IPR000477	Reverse transcriptase domain	-	P92555	AtMg01250; At2g07697
55	Vitvi029841	16	B26	PF00827	Ribosomal L15	IPR000439	Ribosomal protein L15e	GO:0003735|GO:	Q8VYF1	RPL15B At4g17390 dl4730c FCAALL.32
56	Vitvi031015	16	B27	PF14226	non-haem dioxygenase in n	IPR026992	Non-haem dioxygenase N-terminal domain	-	Q9FLV0	DMR6 At5g24530 K18P6.6
57	Vitvi031016	16	B27	Coil	Coil	-	-	-	Q9XGN1	TTG1 At5g24520 K18P6_4 T31K7.8
58	Vitvi031045	16	B28	PF00069	Protein kinase domain	IPR000719	Protein kinase domain	GO:0004672|GO:	Q9ZUZ2	CRK3 CaMK4 CK At2g46700 T3A4.8
59	Vitvi031046	16	B28	PF02519	Auxin responsive protein	IPR003676	Small auxin-up RNA	GO:0009733	Q9ZUZ3	SAUR32 AAM1 At2g46690
60	Vitvi031047	16	B28	-	-	-	-	-	-	-
61	Vitvi033812	18	B29	PF08246	Cathepsin propeptide inhib	IPR013201	Cathepsin propeptide inhibitor domain (I29)	-	Q9FMH8	RD21B At5g43060 MMG4.7
62	Vitvi033813	18	B29	PF10607	CTLH/CRA C-terminal to	IPR024964	CTLH/CRA C-terminal to LisH motif domain	-	-	-
63	Vitvi034331	18	B30	PF07731	Multicopper oxidase	IPR011706	Multicopper oxidase, C-terminal	GO:0005507|GO:	Q9FY79	LAC14 At5g09360 T5E8.160
64	Vitvi034332	18	B30	Coil	Coil	-	-	-	-	-
65	Vitvi034587	18	B31	PF00847	AP2 domain	IPR001471	AP2/ERF domain	GO:0003700|GO:	Q70II3	ERF110 At5g50080 MPF21.9
66	Vitvi036990	19	B32	PF00646	F-box domain	IPR001810	F-box domain	GO:0005515	Q94B46	At4g14096 dl3085w
67	Vitvi037434	19	B33	PF00083	Sugar (and other) transport	IPR005828	Major facilitator, sugar transporter-like	GO:0016021|GO:	Q9SA38	OCT3 3-Oct At1g16390 F3O9.19

Table. S.9. Candidate gene of continuous traits in GWAS results

q	GeneID	Chromosome	Trait	PFAM	PFAM Protein Function	IPR	IPR Protein Function2	Go_ID	Swissport	gene_name
1	Vitvi000746	1	C1	PF00657	GDSL-like Lipase/Acylhydrolase	IPR001087	GDSL lipase/esterase	GO:0016788	Q9LIN2	At3g26430 F20C19.19
2	Vitvi000747	1	C1	PF05910	Plant protein of unknown function (DUF868	IPR008586	Protein of unknown function DUF868, plant	-	-	-
3	Vitvi000748	1	C1	PF00481	Protein phosphatase 2C	IPR001932	PPM-type phosphatase domain	GO:0016791	Q9FXE4	At1g67820 F12A21.5
4	Vitvi000749	1	C1	Coil	Coil	-	-	-	F4HVG8	CSK At1g67840 F12A21.3
5	Vitvi000767	1	C3	PF01554	MatE	IPR002528	Multi antimicrobial extrusion protein	GO:0015297|G	Q38956	DTX29 At3g26590 MFE16.12
6	Vitvi000768	1	C3	PF01554	MatE	IPR002528	Multi antimicrobial extrusion protein	GO:0015297|G	Q38956	DTX29 At3g26590 MFE16.12
7	Vitvi000861	1	C4	PF01398	JAB1/Mov34/MPN/PAD-1 ubiquitin protea	IPR000555	JAB1/MPN/MOV34 metalloenzyme domain	GO:0005515|G	Q6NKP9	AMSH2 At1g10600 F20B24.2 T10O24.25
8	Vitvi000862	1	C4	PF00484	Carbonic anhydrase	IPR001765	Carbonic anhydrase	GO:0004089|G	P27141	-
9	Vitvi002384	2	C5	PF00575	S1 RNA binding domain	IPR003029	S1 domain	GO:0003676	-	-
10	Vitvi002385	2	C5	PF00412	LIM domain	IPR001781	Zinc finger, LIM-type	-	O04193	WLIN2A WLIM2 At2g39900 T28M21.6
11	Vitvi002392	2	C6	PF01429	Methyl-CpG binding domain	IPR001739	Methyl-CpG DNA binding	GO:0003677	Q9LTJ8	MBD13 At5g52230 F17P19.13
12	Vitvi002393	2	C6	-	-	-	-	-	P10978	-
13	Vitvi002441	2	C7	PF01554	MatE	IPR002528	Multi antimicrobial extrusion protein	GO:0015297|G	O82752	DTX49 NIC1 At4g23030 F7H19.220
14	Vitvi002442	2	C7	PF00847	AP2 domain	IPR001471	AP2/ERF domain	GO:0003700|G	Q9CAN9	ERF026 At1g63040 F16M19.1 F16P17.21
15	Vitvi002475	2	C8	Coil	Coil	-	-	-	Q9FLI1	BHLH36 EN6 At5g51780 MIO24.9
16	Vitvi002476	2	C8	PF04146	YT521-B-like domain	IPR007275	YTH domain	GO:0003723	Q0DA50	Os06g0677700 LOC_Os06g46400 B1153E06.24 P0710B08.18
17	Vitvi002477	2	C8	PF13966	zinc-binding in reverse transcriptase	IPR026960	Reverse transcriptase zinc-binding domain	-	-	-
18	Vitvi002478	2	C8	PF00078	Reverse transcriptase (RNA-dependent DN	IPR000477	Reverse transcriptase domain	-	P92555	AtMg01250; At2g07697
19	Vitvi002479	2	C8	PF00078	Reverse transcriptase (RNA-dependent DN	IPR000477	Reverse transcriptase domain	-	P14381	-
20	Vitvi002480	2	C8	-	-	-	-	-	-	-
21	Vitvi002481	2	C8	PF14111	Domain of unknown function (DUF4283)	IPR025558	Domain of unknown function DUF4283	-	-	-
22	Vitvi002503	2	C9	PF00155	Aminotransferase class I and II	IPR004839	Aminotransferase, class I/classII	GO:0009058|G	Q9LQ10	ACS10 At1g62960 F16P17.11
23	Vitvi002504	2	C9	PF13855	Leucine rich repeat	IPR001611	Leucine-rich repeat	GO:0005515	C0LGE4	At1g12460 F5O11.21 T12C24.1
24	Vitvi002505	2	C9	PF09335	SNARE associated Golgi protein	IPR032816	SNARE associated Golgi protein	-	-	-
25	Vitvi002522	2	C10	PF04116	Fatty acid hydroxylase superfamily	IPR006694	Fatty acid hydroxylase	GO:0005506|G	Q8L7W5	SMO1-1 At4g12110 F16J13.180
26	Vitvi002523	2	C10	-	-	-	-	-	-	-
27	Vitvi002560	2	C11	PF14368	Probable lipid transfer	IPR016140	Bifunctional inhibitor/plant lipid transfer protein/seed storage helical domain	-	Q7EB72	LTPG15 XYLP11 XYP7 At2g48130 F11L15.3
28	Vitvi002561	2	C11	-	-	-	-	-	-	-
29	Vitvi002562	2	C11	PF04043	Plant invertase/pectin methylesterase inhibi	IPR006501	Pectinesterase inhibitor domain	GO:0004857	Q9SI72	PMEI9 At1g62770 F23N19.14
30	Vitvi002563	2	C11	PF04043	Plant invertase/pectin methylesterase inhibi	IPR006501	Pectinesterase inhibitor domain	GO:0004857	P17407	-
31	Vitvi004218	3	C12	PF09333	Autophagy-related protein C terminal doma	IPR015412	Autophagy-related, C-terminal	-	F8S296	ATG2 APG2 PEUP1 At3g19190 MVI11.10/MVI11.11
32	Vitvi004651	3	C13	-	-	-	-	-	-	-
33	Vitvi004652	3	C13	PF00118	TCP-1/cpn60 chaperonin family	IPR002423	Chaperonin Cpn60/GroEL/TCP-1 family	GO:0005524|G	Q84WV1	CCT3 At5g26360 F9D12.18
34	Vitvi008421	5	C15	PF03109	ABC1 atypical kinase-like domain	IPR004147	ABC1 atypical kinase-like domain	-	Q55680	sll0005
35	Vitvi010601	6	C16	PF03732	Retrotransposon gag protein	IPR005162	Retrotransposon gag domain	-	-	-
36	Vitvi010602	6	C16	PF14111	Domain of unknown function (DUF4283)	IPR025558	Domain of unknown function DUF4283	-	-	-
37	Vitvi010603	6	C16	Coil	Coil	-	-	-	P14381	-
38	Vitvi010614	6	C17	PF12776	Myb/SANT-like DNA-binding domain	IPR024752	Myb/SANT-like domain	-	-	-
39	Vitvi010615	6	C17	PF03953	Tubulin C-terminal domain	IPR018316	Tubulin/FtsZ, 2-layer sandwich domain	-	P45960	TUBB4 OSTB-16 R1623 RTUB-1 TUB4 Os01g0805900 LOC_Os01g59150 P0034E02.62
40	Vitvi011215	7	C18	PF01257	Thioredoxin-like [2Fe-2S] ferredoxin	-	-	-	O22769	At4g02580 T10P11.14
41	Vitvi011217	7	C18	PF00010	Helix-loop-helix DNA-binding domain	IPR011598	Myc-type, basic helix-loop-helix (bHLH) domain	GO:0046983	O22768	UNE12 BHLH59 EN93 At4g02590 T10P11.13
42	Vitvi011218	7	C18	PF00290	Tryptophan synthase alpha chain	IPR002028	Tryptophan synthase, alpha chain	GO:0004834|G	Q42529	TSA1 TRP3 TSA2 At3g54640 T14E10.210
43	Vitvi012725	7	C19	PF05739	SNARE domain	IPR000727	Target SNARE coiled-coil homology domain	-	Q9SA23	SYP51 At1g16240 F3O9.4
44	Vitvi012726	7	C19	PF13966	zinc-binding in reverse transcriptase	IPR026960	Reverse transcriptase zinc-binding domain	-	-	-
45	Vitvi012749	7	C20	Coil	Coil	-	-	-	-	-
46	Vitvi015004	8	C21	-	-	-	-	-	-	-
47	Vitvi015005	8	C21	-	-	-	-	-	-	-
48	Vitvi015008	8	C22	-	-	-	-	-	-	-
49	Vitvi017348	9	C23	PF08263	Leucine rich repeat N-terminal domain	IPR013210	Leucine-rich repeat-containing N-terminal, plant-type	-	Q9LJY0	PRK4 PRKA At3g20190 MAL21.21
50	Vitvi017349	9	C23	PF12708	Pectate lyase superfamily protein	IPR024535	Pectate lyase superfamily protein	-	O49432	QRT3 At4g20050 F18F4.150
51	Vitvi017350	9	C23	PF13249	Squalene-hopene cyclase N-terminal domai	IPR032697	Squalene cyclase, N-terminal	-	Q8W3Z4	CASBPX1
52	Vitvi017414	9	C24	PF17921	Integrase zinc binding domain	IPR041588	Integrase zinc-binding domain	-	P0CT42	Tf2-7 SPAC13D1.01c
53	Vitvi017415	9	C24	-	-	-	-	-	-	-
54	Vitvi017416	9	C24	-	-	-	-	-	-	-
55	Vitvi021811	12	C25	PF08387	FBD	IPR006566	FBD domain	-	Q9ZR09	At4g03220 F4C21.15
56	Vitvi021812	12	C25	PF03321	GH3 auxin-responsive promoter	IPR004993	GH3 family	-	Q9LSQ4	GH3.6 DFL1 At5g54510 F24B18.13
57	Vitvi021813	12	C25	PF13833	EF-hand domain pair	IPR002048	EF-hand domain	GO:0005509	O81831	KRP1 At4g27280 M4I22.90
58	Vitvi021951	12	C26	PF00689	Cation transporting ATPase, C-terminus	IPR006068	Cation-transporting P-type ATPase, C-terminal	-	Q9LY77	ACA12 At3g63380 MAA21_10
59	Vitvi021952	12	C26	PF00689	Cation transporting ATPase, C-terminus	IPR006068	Cation-transporting P-type ATPase, C-terminal	-	Q9LY77	ACA12 At3g63380 MAA21_10
60	Vitvi021953	12	C26	PF00689	Cation transporting ATPase, C-terminus	IPR006068	Cation-transporting P-type ATPase, C-terminal	-	Q9LY77	ACA12 At3g63380 MAA21_10
61	Vitvi021954	12	C26	PF00560	Leucine Rich Repeat	IPR001611	Leucine-rich repeat	GO:0005515	Q9C637	RLP6 At1g45616 F2G19.6
62	Vitvi021955	12	C26	PF14223	gag-polypeptide of LTR copia-type	-	-	-	Q94HW2	RE1 RF12 RF28 At1g58889 R18I; RE1 At1g59265 T4M14.18
63	Vitvi024368	13	C27	PF00078	Reverse transcriptase (RNA-dependent DN	IPR000477	Reverse transcriptase domain	-	P92555	AtMg01250; At2g07697
64	Vitvi024369	13	C27	PF10453	Nuclear fragile X mental retardation-interac	IPR019496	Nuclear fragile X mental retardation-interacting protein 1, conserved domain	-	-	-
65	Vitvi028358	15	C28	PF04570	zinc-finger of the FCS-type, C2-C2	IPR007650	Zf-FLZ domain	-	O80506	FLZ3 DUF581-9 At2g44670 F16B22.16
66	Vitvi028769	15	C29	PF00847	AP2 domain	IPR001471	AP2/ERF domain	GO:0003700|G	P92966	RS41 RSP41 At5g52040 MSG15.12
67	Vitvi028770	15	C29	Coil	Coil	-	-	-	-	-
68	Vitvi028771	15	C29	PF05648	Peroxisomal biogenesis factor 11 (PEX11)	IPR008733	Peroxisomal biogenesis factor 11	GO:0005779|G	Q9LQ73	PEX11C PEX11-1 At1g01820 T1N6.24
69	Vitvi029126	15	C30	Coil	Coil	-	-	-	Q0WWX5	PRD3 At1g01690 T1N6.6
70	Vitvi029127	15	C30	Coil	Coil	-	-	-	F4HSD5	TRM32 At1g07620 F24B9.32
71	Vitvi031037	16	C31	PF00076	RNA recognition motif. (a.k.a. RRM, RBD	IPR000504	RNA recognition motif domain	GO:0003723	Q9M1S3	ARP1 At3g54770 T5N23.130
72	Vitvi031038	16	C31	PF14009	PADRE domain	IPR025322	PADRE domain	-	-	-
73	Vitvi031039	16	C31	PF00011	Hsp20/alpha crystallin family	IPR002068	Alpha crystallin/Hsp20 domain	-	Q95661	HSP21 TOM111
74	Vitvi031040	16	C31	PF13178	Protein of unknown function (DUF4005)	IPR025064	Domain of unknown function DUF4005	-	Q9MAM4	IQD18 At1g01110 T25K16.10
75	Vitvi031041	16	C31	PF08879	WRC	IPR014977	WRC domain	-	Q9FJB8	GRF7 At5g53660 MNC6.20
76	Vitvi031043	16	C32	PF05030	SSXT protein (N-terminal region)	IPR007726	SS18, N-terminal	-	Q9MAL9	GIF2 At1g01160 F6F3_1 T25K16.15
77	Vitvi031044	16	C32	Coil	Coil	-	-	-	-	-
78	Vitvi031045	16	C32	PF00069	Protein kinase domain	IPR000719	Protein kinase domain	GO:0004672|G	Q9ZUZ2	CRK3 CaMK4 CK At2g46700 T3A4.8
79	Vitvi031046	16	C32	PF02519	Auxin responsive protein	IPR003676	Small auxin-up RNA	GO:0009733	Q9ZUZ3	SAUR32 AAM1 At2g46690
80	Vitvi031047	16	C33	-	-	-	-	-	-	-
81	Vitvi033967	18	C34	PF00657	GDSL-like Lipase/Acylhydrolase	IPR001087	GDSL lipase/esterase	GO:0016788	Q94CH6	EXL3 At1g75900 T4O12.13
82	Vitvi034002	18	C35	PF00438	S-adenosylmethionine synthetase, N-termin	IPR022628	S-adenosylmethionine synthetase, N-terminal	GO:0004478|G	Q9FPL6	METK2
83	Vitvi034003	18	C35	PF00438	S-adenosylmethionine synthetase, N-termin	IPR022628	S-adenosylmethionine synthetase, N-terminal	GO:0004478|G	A7Q0V4	METK5 GSVIVT00028192001 LOC100243560
84	Vitvi034004	18	C35	Coil	Coil	-	-	-	-	-
85	Vitvi034587	18	C36	PF00847	AP2 domain	IPR001471	AP2/ERF domain	GO:0003700|G	Q70II3	ERF110 At5g50080 MPF21.9

Table. S.10. Upregulated and downregulated genes by *Tetranyhus urticae*

ID	log2FoldChange	pvalue	padj	change	ID	log2FoldChange	pvalue	padj	change
Vitvi022065	8.293335842	2.45E-15	2.72E-14	UP	Vitvi033727	-1.000004106	0.000236521	0.000822923	DOWN
Vitvi016210	8.195813573	4.27E-15	4.66E-14	UP	Vitvi011538	-1.000305351	2.49E-19	3.74E-18	DOWN
Vitvi014209	7.777335687	4.6E-13	4.27E-12	UP	Vitvi035725	-1.000580726	0.009453434	0.023968271	DOWN
Vitvi007988	7.656688142	6.43E-13	5.95E-12	UP	Vitvi011674	-1.002793498	0.00000989	0.0000423	DOWN
Vitvi007290	7.476377577	1.12E-44	6.49E-43	UP	Vitvi005779	-1.003415594	0.006958303	0.018143396	DOWN
Vitvi024267	7.431833793	1.64E-12	1.46E-11	UP	Vitvi008158	-1.004158701	0.000026	0.000104935	DOWN
Vitvi030451	7.39126251	3.54E-12	3.07E-11	UP	Vitvi010507	-1.005012567	4.73E-19	6.95E-18	DOWN
Vitvi018092	7.3040844	1.08E-17	1.44E-16	UP	Vitvi024985	-1.005480543	0.020356312	0.047023475	DOWN
Vitvi021779	7.276645424	1.2E-11	9.85E-11	UP	Vitvi005798	-1.006349071	1.5E-12	3.13E-10	DOWN
Vitvi028954	7.25621315	4.1E-11	3.18E-10	UP	Vitvi014388	-1.006474265	0.000148975	0.003817682	DOWN
Vitvi003290	7.17212637	2.01E-11	1.61E-10	UP	Vitvi015322	-1.00694751	0.000000326	0.0000017	DOWN
Vitvi031534	7.167894838	2.21E-27	5.43E-26	UP	Vitvi010054	-1.007120883	0.000000231	0.00000122	DOWN
Vitvi027444	7.107772221	6.41E-21	1.08E-19	UP	Vitvi020334	-1.007276908	4.36E-22	7.88E-21	DOWN
Vitvi010686	7.057084379	4.77E-11	3.68E-10	UP	Vitvi019970	-1.007514289	5.74E-22	1.03E-20	DOWN
Vitvi024216	7.011465123	1.48E-10	1.09E-09	UP	Vitvi011659	-1.007619369	0.002506864	0.007201161	DOWN
Vitvi005152	6.999187045	1.24E-10	9.2E-10	UP	Vitvi010626	-1.007707276	0.001769782	0.027251531	DOWN
Vitvi018091	6.880875215	2.14E-10	1.56E-09	UP	Vitvi003445	-1.007770274	8.73E-09	5.42E-08	DOWN
Vitvi004117	6.77727911	6.5E-11	4.95E-10	UP	Vitvi006416	-1.00799603	0.0000508	0.000195403	DOWN
Vitvi019580	6.759283761	2.09E-10	1.52E-09	UP	Vitvi025234	-1.008048377	1.17E-10	0.000000017	DOWN
Vitvi003289	6.743917978	5.65E-10	3.96E-09	UP	Vitvi032017	-1.00809909	1.02E-31	3.24E-30	DOWN
Vitvi007275	6.736953968	2.41E-72	4.44E-70	UP	Vitvi033666	-1.008435042	1.61E-27	3.99E-26	DOWN
Vitvi018093	6.727165992	2.25E-69	3.68E-67	UP	Vitvi032121	-1.008974286	1.12E-11	9.22E-11	DOWN
Vitvi011331	6.723193687	9.2E-11	6.91E-10	UP	Vitvi034759	-1.009328871	0.000000143	0.000000781	DOWN
Vitvi033641	6.593301448	2.97E-10	2.13E-09	UP	Vitvi033904	-1.009589315	3.12E-18	4.3E-17	DOWN
Vitvi003307	6.5582426	3.07E-14	3.15E-13	UP	Vitvi033330	-1.009876542	1.9E-11	1.53E-10	DOWN
Vitvi027201	6.520722352	2.33E-09	1.54E-08	UP	Vitvi021457	-1.010585385	8.71E-14	8.68E-13	DOWN
Vitvi007285	6.511863831	6.1E-80	1.39E-77	UP	Vitvi016251	-1.010685399	0.00000408	0.0000184	DOWN
Vitvi010098	6.44429577	5.56E-09	3.54E-08	UP	Vitvi021132	-1.011081315	0.012771398	0.031253546	DOWN
Vitvi019289	6.433368544	6.45E-09	4.07E-08	UP	Vitvi012728	-1.011528793	5.71E-15	6.18E-14	DOWN
Vitvi004536	6.427306667	5.86E-09	3.72E-08	UP	Vitvi025022	-1.011601424	1.45E-10	1.07E-09	DOWN
Vitvi018423	6.424309461	2.04E-09	1.36E-08	UP	Vitvi015021	-1.011638155	3.22E-16	3.85E-15	DOWN
Vitvi006990	6.418252532	9.38E-14	9.32E-13	UP	Vitvi000383	-1.012142546	1.2E-16	1.48E-15	DOWN
Vitvi008055	6.353546205	3.57E-42	1.82E-40	UP	Vitvi000049	-1.012578127	1.19E-18	1.7E-17	DOWN
Vitvi030443	6.333190175	3.44E-59	3.95E-57	UP	Vitvi022539	-1.012673553	6.71E-15	7.22E-14	DOWN
Vitvi030470	6.29892166	1.57E-09	1.06E-08	UP	Vitvi032744	-1.012987254	4.23E-10	2.99E-09	DOWN
Vitvi017349	6.265703641	3.35E-13	3.15E-12	UP	Vitvi001689	-1.013366654	0.0000418	0.000163041	DOWN
Vitvi029300	6.176522711	2.06E-23	4.01E-22	UP	Vitvi033545	-1.013618764	0.003667931	0.046963434	DOWN
Vitvi006669	6.14879656	3.65E-09	2.36E-08	UP	Vitvi019234	-1.01376969	0.000000628	0.00000315	DOWN
Vitvi034545	6.133139173	3.93E-12	3.38E-11	UP	Vitvi025429	-1.014367079	0.000823529	0.002614085	DOWN
Vitvi016622	6.129531583	4.24E-08	0.000000244	UP	Vitvi029276	-1.014416178	5.86E-08	0.000000332	DOWN
Vitvi030466	6.061983705	2.97E-47	1.99E-45	UP	Vitvi033489	-1.015921712	0.0000319	0.000126726	DOWN
Vitvi030444	6.01060842	9.71E-08	0.000000537	UP	Vitvi005692	-1.016811086	0.000133434	0.000481233	DOWN
Vitvi028953	5.99619534	0.000000118	0.000000649	UP	Vitvi014915	-1.017157652	0.019676433	0.045728571	DOWN
Vitvi030469	5.994895128	8.88E-64	1.16E-61	UP	Vitvi006653	-1.017387318	3.12E-18	4.3E-17	DOWN
Vitvi008266	5.98561114	0.000000144	0.000000782	UP	Vitvi011374	-1.017500652	2.25E-21	3.89E-20	DOWN
Vitvi021416	5.963773726	1.66E-11	1.34E-10	UP	Vitvi033915	-1.018631456	0.000000434	0.00000223	DOWN
Vitvi003981	5.959559449	4.44E-08	0.000000255	UP	Vitvi007657	-1.018934961	2.79E-08	0.000000164	DOWN
Vitvi002473	5.946993505	1.18E-18	1.68E-17	UP	Vitvi036685	-1.019049997	1.28E-22	2.38E-21	DOWN
Vitvi032234	5.943434806	2.38E-11	1.89E-10	UP	Vitvi035956	-1.019519913	6.36E-21	1.07E-19	DOWN
Vitvi022403	5.899628202	0.000000196	0.00000104	UP	Vitvi025305	-1.019937838	2.56E-17	3.33E-16	DOWN
Vitvi015485	5.873079939	6.53E-08	0.000000367	UP	Vitvi025837	-1.020329605	0.000835522	0.002646776	DOWN
Vitvi018642	5.867905171	6.14E-15	6.63E-14	UP	Vitvi013543	-1.020340947	0.006045914	0.01598394	DOWN
Vitvi008136	5.857224771	0.000000158	0.000000853	UP	Vitvi019670	-1.020687361	0.00000124	0.00000599	DOWN
Vitvi029308	5.85559821	2.49E-08	0.000000147	UP	Vitvi031207	-1.021846198	6.39E-20	1.01E-18	DOWN
Vitvi030472	5.842161254	9.15E-43	4.77E-41	UP	Vitvi013621	-1.021945188	0.000125928	0.00045619	DOWN
Vitvi028478	5.833986356	2.4E-41	1.17E-39	UP	Vitvi025746	-1.022634187	0.002403941	0.006929027	DOWN

ID	log2FoldChange	pvalue	padj	change	ID	log2FoldChange	pvalue	padj	change
Vitvi008767	5.823441712	6.61E-08	0.000000372	UP	Vitvi017663	-1.022844784	0.000937273	0.002940214	DOWN
Vitvi030479	5.804921585	0.000000681	0.00000341	UP	Vitvi011694	-1.023491373	0.0193459	0.045053189	DOWN
Vitvi007679	5.80265881	1.91E-137	1.94E-134	UP	Vitvi015253	-1.023803669	0.0000039	0.0000177	DOWN
Vitvi034939	5.784616727	5.61E-11	4.31E-10	UP	Vitvi018112	-1.024307602	0.002996303	0.008467514	DOWN
Vitvi030446	5.771939438	2.85E-121	1.75E-118	UP	Vitvi010463	-1.024408512	1.02E-13	1.01E-12	DOWN
Vitvi010287	5.688687183	0.000000364	0.00000189	UP	Vitvi016460	-1.024587278	0.000519354	0.001705872	DOWN
Vitvi036376	5.647673078	0.00000106	0.00000516	UP	Vitvi004332	-1.025273206	6.77E-13	6.26E-12	DOWN
Vitvi032481	5.632155403	2.35E-13	2.24E-12	UP	Vitvi029468	-1.025397112	0.000000208	0.00000111	DOWN
Vitvi035161	5.620342303	1.37E-13	1.34E-12	UP	Vitvi036587	-1.025565333	1.78E-15	1.99E-14	DOWN
Vitvi008056	5.605941533	2.87E-30	8.35E-29	UP	Vitvi023739	-1.025795775	2.26E-08	0.000000134	DOWN
Vitvi018002	5.574963222	0.000000544	0.00000276	UP	Vitvi003337	-1.025987148	2.34E-09	1.54E-08	DOWN
Vitvi006046	5.574514383	0.000000144	0.000000782	UP	Vitvi007840	-1.026268617	2.67E-08	0.000000158	DOWN
Vitvi029303	5.562503805	4.78E-19	7.01E-18	UP	Vitvi030632	-1.02648515	0.0000356	0.000140207	DOWN
Vitvi030457	5.542288345	8.05E-16	9.27E-15	UP	Vitvi036697	-1.026523117	0.000000431	0.00000222	DOWN
Vitvi000212	5.539811042	0.000000162	0.000000873	UP	Vitvi028788	-1.027252985	1.61E-13	1.56E-12	DOWN
Vitvi006045	5.530057429	5.91E-56	5.8E-54	UP	Vitvi033391	-1.027294807	0.00000286	0.0000132	DOWN
Vitvi007332	5.515516521	1.58E-223	1.07E-219	UP	Vitvi023040	-1.027366427	1.94E-12	1.72E-11	DOWN
Vitvi030462	5.51202772	0.00000405	0.0000183	UP	Vitvi013527	-1.027607269	4.87E-14	4.95E-13	DOWN
Vitvi012696	5.495266874	7.92E-15	8.48E-14	UP	Vitvi031284	-1.028369267	7.63E-11	5.76E-10	DOWN
Vitvi032486	5.47317986	6.02E-09	3.81E-08	UP	Vitvi003142	-1.028691619	8.02E-29	2.13E-27	DOWN
Vitvi029301	5.471067954	4.04E-18	5.54E-17	UP	Vitvi011470	-1.029009788	0.00072988	0.002340963	DOWN
Vitvi029103	5.469138596	3.85E-30	1.11E-28	UP	Vitvi018614	-1.02905244	2.01E-27	4.96E-26	DOWN
Vitvi034410	5.466807777	0.00000106	0.00000516	UP	Vitvi006652	-1.029231385	1.38E-10	1.02E-09	DOWN
Vitvi034888	5.463926291	6.95E-10	4.84E-09	UP	Vitvi023415	-1.029406025	4.43E-09	2.85E-08	DOWN
Vitvi004182	5.463799406	0.00000408	0.0000184	UP	Vitvi023755	-1.029998265	0.000000134	0.000000731	DOWN
Vitvi030572	5.458309118	0.000000818	0.00000405	UP	Vitvi031274	-1.030623875	0.000179794	0.000636343	DOWN
Vitvi031685	5.451993267	0.000000887	0.00000437	UP	Vitvi029587	-1.031058336	6.03E-09	3.82E-08	DOWN
Vitvi024415	5.446236177	0.00000111	0.00000538	UP	Vitvi016472	-1.031207448	3.81E-14	3.91E-13	DOWN
Vitvi029042	5.421857764	0.0000108	0.0000459	UP	Vitvi005713	-1.031756268	0.000987019	0.003084357	DOWN
Vitvi009457	5.404777595	3.5E-168	7.9E-165	UP	Vitvi020468	-1.031915949	0.00355553	0.009902636	DOWN
Vitvi002854	5.395916426	2.87E-31	8.81E-30	UP	Vitvi000377	-1.032211393	0.004601551	0.012513952	DOWN
Vitvi009653	5.387413394	0.00000358	0.0000163	UP	Vitvi003823	-1.032324718	0.000000634	0.00000318	DOWN
Vitvi037006	5.383788567	5.71E-17	7.21E-16	UP	Vitvi002470	-1.032589999	3.82E-29	1.03E-27	DOWN
Vitvi005066	5.380658651	0.00000809	0.0000351	UP	Vitvi011463	-1.032908795	0.00010699	0.000392519	DOWN
Vitvi011227	5.375429555	0.00000512	0.0000228	UP	Vitvi033908	-1.033273353	1.13E-12	1.02E-11	DOWN
Vitvi031834	5.373108159	0.00000469	0.000021	UP	Vitvi010969	-1.033368464	0.000173959	0.000617323	DOWN
Vitvi010187	5.37043758	0.00000449	0.0000202	UP	Vitvi036408	-1.033479387	3E-15	3.31E-14	DOWN
Vitvi020050	5.368807568	3.26E-12	2.83E-11	UP	Vitvi010963	-1.033889995	0.0000209	0.0000853	DOWN
Vitvi013368	5.367607323	0.0000167	0.0000692	UP	Vitvi001035	-1.034022785	1.64E-11	1.33E-10	DOWN
Vitvi015868	5.349553621	7.01E-19	1.01E-17	UP	Vitvi012640	-1.034353698	1.58E-11	1.28E-10	DOWN
Vitvi007288	5.318754249	0.00000752	0.0000327	UP	Vitvi011500	-1.034538288	0.0000444	0.000172498	DOWN
Vitvi002858	5.316672308	6.27E-29	1.67E-27	UP	Vitvi005382	-1.034548362	8.32E-08	0.000000463	DOWN
Vitvi030456	5.308494761	4.93E-25	1.08E-23	UP	Vitvi021716	-1.034743531	0.00000106	0.00000515	DOWN
Vitvi019677	5.302975087	3.36E-53	2.97E-51	UP	Vitvi015566	-1.034965464	5.51E-08	0.000000313	DOWN
Vitvi005154	5.300296733	0.0000232	0.0000941	UP	Vitvi020511	-1.036061078	0.00000709	0.000031	DOWN
Vitvi030452	5.288376132	6.09E-56	5.95E-54	UP	Vitvi030781	-1.036562829	0.0000041	0.0000185	DOWN
Vitvi008038	5.288010958	0.00000891	0.0000384	UP	Vitvi019355	-1.036787651	0.000000147	0.000000796	DOWN
Vitvi028684	5.279651219	0.00000879	0.0000379	UP	Vitvi018133	-1.037994007	0.000000701	0.0000035	DOWN
Vitvi027091	5.272429197	2.67E-14	2.76E-13	UP	Vitvi022411	-1.038457281	0.003118357	0.008776509	DOWN
Vitvi033175	5.267246026	0.01015665	0.02556405	UP	Vitvi036181	-1.038614194	5.71E-10	0.000000004	DOWN
Vitvi007286	5.252851403	0.00000425	0.0000191	UP	Vitvi020226	-1.038941521	1.95E-27	4.81E-26	DOWN
Vitvi025774	5.25261764	0.0000153	0.0000638	UP	Vitvi026689	-1.03925695	4.03E-12	3.46E-11	DOWN
Vitvi030475	5.23884227	2.37E-40	1.11E-38	UP	Vitvi024646	-1.040146974	3.42E-12	2.97E-11	DOWN
Vitvi021375	5.180225083	0.0000217	0.0000882	UP	Vitvi023201	-1.040508412	0.000569463	0.001856947	DOWN
Vitvi029312	5.177738567	0.0000138	0.0000578	UP	Vitvi030533	-1.040694432	1.15E-18	1.65E-17	DOWN
Vitvi029309	5.170810791	0.00000449	0.0000202	UP	Vitvi000264	-1.040792353	0.000206185	0.000722467	DOWN
Vitvi020111	5.162356607	1.19E-37	4.94E-36	UP	Vitvi028868	-1.041258685	2.46E-08	0.000000145	DOWN

ID	log2FoldChange	pvalue	padj	change	ID	log2FoldChange	pvalue	padj	change
Vitvi021271	5.149922434	1.14E-31	3.58E-30	UP	Vitvi031332	-1.041511011	3.01E-08	0.000000177	DOWN
Vitvi013553	5.146044469	8.57E-09	5.32E-08	UP	Vitvi008784	-1.042078038	6.48E-17	8.15E-16	DOWN
Vitvi014874	5.136583324	6.22E-11	4.75E-10	UP	Vitvi018170	-1.042196096	5.16E-27	1.25E-25	DOWN
Vitvi013124	5.130234986	1.11E-45	6.74E-44	UP	Vitvi006971	-1.042604909	0.000000918	0.00000451	DOWN
Vitvi010688	5.129605718	1.25E-10	9.29E-10	UP	Vitvi001945	-1.043097997	4.19E-11	3.25E-10	DOWN
Vitvi008766	5.114784432	9.67E-09	5.96E-08	UP	Vitvi018356	-1.043313958	1.14E-11	9.34E-11	DOWN
Vitvi034895	5.111841584	5.22E-43	2.75E-41	UP	Vitvi001158	-1.043392886	1.17E-09	7.96E-09	DOWN
Vitvi007790	5.11003367	4.13E-11	3.21E-10	UP	Vitvi013062	-1.043435949	0.000249809	0.005768306	DOWN
Vitvi013849	5.091387598	2.33E-13	2.22E-12	UP	Vitvi004272	-1.043801653	1.21E-18	1.73E-17	DOWN
Vitvi030450	5.086406498	8.3E-52	6.83E-50	UP	Vitvi030416	-1.043856338	4.69E-15	5.12E-14	DOWN
Vitvi018094	5.080137268	0.0000315	0.000125062	UP	Vitvi030269	-1.043938897	0.0000527	0.00020236	DOWN
Vitvi029314	5.073720011	0.000000015	9.06E-08	UP	Vitvi024987	-1.043963891	0.010553034	0.026433991	DOWN
Vitvi033497	5.072809117	5.99E-66	8.33E-64	UP	Vitvi025175	-1.044245406	0.000188674	0.000665106	DOWN
Vitvi009204	5.071548206	0.00000791	0.0000343	UP	Vitvi008005	-1.044398729	0.000980402	0.003066272	DOWN
Vitvi011271	5.060401685	4.44E-13	4.14E-12	UP	Vitvi007370	-1.044402342	1.23E-15	1.4E-14	DOWN
Vitvi002428	5.057280873	1.29E-39	5.82E-38	UP	Vitvi002531	-1.046212419	2.8E-09	1.84E-08	DOWN
Vitvi008032	5.034886962	0.00000361	0.0000164	UP	Vitvi015638	-1.046930613	9.71E-13	8.86E-12	DOWN
Vitvi028289	5.029211026	9.5E-11	7.11E-10	UP	Vitvi002991	-1.047108862	1.89E-14	1.97E-13	DOWN
Vitvi020112	5.013216885	0.000105624	0.000388105	UP	Vitvi017603	-1.047188162	0.0000112	0.0000474	DOWN
Vitvi011177	5.011094213	3.48E-17	4.47E-16	UP	Vitvi002898	-1.047896671	0.000329521	0.00111796	DOWN
Vitvi002863	5.007263364	0.000036	0.000141502	UP	Vitvi017660	-1.047995731	4.44E-16	5.22E-15	DOWN
Vitvi035756	4.997430591	0.0000199	0.0000817	UP	Vitvi009876	-1.048073174	2.21E-14	2.3E-13	DOWN
Vitvi009963	4.993057941	1.29E-10	9.54E-10	UP	Vitvi001320	-1.048555373	2.08E-08	0.000000124	DOWN
Vitvi006667	4.987665366	1.04E-69	1.72E-67	UP	Vitvi031815	-1.048861211	3.84E-10	2.73E-09	DOWN
Vitvi018100	4.975888188	6.84E-69	1.09E-66	UP	Vitvi013741	-1.049390558	0.000135326	0.000487451	DOWN
Vitvi017505	4.968346248	3.44E-54	3.12E-52	UP	Vitvi007246	-1.049520167	0.001371723	0.004179158	DOWN
Vitvi013252	4.95845388	6.03E-21	1.02E-19	UP	Vitvi025932	-1.049571113	8.15E-16	9.38E-15	DOWN
Vitvi000457	4.927598805	8.89E-17	1.11E-15	UP	Vitvi020773	-1.04970464	1.79E-10	1.31E-09	DOWN
Vitvi010825	4.872736996	2.75E-40	1.27E-38	UP	Vitvi027287	-1.049729789	1.81E-21	3.14E-20	DOWN
Vitvi035641	4.862183723	0.0000229	0.0000929	UP	Vitvi023380	-1.049811696	0.0000702	0.000264109	DOWN
Vitvi001694	4.85727945	0.00000756	0.0000329	UP	Vitvi000695	-1.050404836	1.35E-21	2.38E-20	DOWN
Vitvi031787	4.843023276	6.85E-10	4.77E-09	UP	Vitvi021571	-1.050409811	3.13E-15	3.45E-14	DOWN
Vitvi019960	4.840858758	4.55E-13	4.23E-12	UP	Vitvi030771	-1.050891253	5.55E-30	1.59E-28	DOWN
Vitvi015441	4.833770026	0.0000922	0.000341086	UP	Vitvi001906	-1.051523095	1.37E-14	1.44E-13	DOWN
Vitvi008057	4.814517319	9.8E-55	9.17E-53	UP	Vitvi033300	-1.05157519	5.05E-30	1.45E-28	DOWN
Vitvi035265	4.797232978	1.04E-09	7.12E-09	UP	Vitvi008351	-1.051667794	0.000150583	0.000538494	DOWN
Vitvi024466	4.795599144	0.000288319	0.000986913	UP	Vitvi013026	-1.052171198	0.000372209	0.001252535	DOWN
Vitvi030449	4.793878475	2.11E-37	8.59E-36	UP	Vitvi012919	-1.052174972	1.95E-15	2.18E-14	DOWN
Vitvi029302	4.778366588	1.19E-09	8.11E-09	UP	Vitvi033288	-1.052564233	3.44E-16	4.1E-15	DOWN
Vitvi036119	4.770686891	0.000133434	0.000481233	UP	Vitvi031234	-1.0525666	0.017447222	0.041150098	DOWN
Vitvi004208	4.759853022	0.000131851	0.000475948	UP	Vitvi004023	-1.052902759	0.003306266	0.00926174	DOWN
Vitvi021951	4.756270344	1.08E-106	5.25E-104	UP	Vitvi023806	-1.053343968	3.1E-20	5.01E-19	DOWN
Vitvi026040	4.751086753	4.97E-25	1.08E-23	UP	Vitvi019845	-1.053532787	0.00000173	0.00000819	DOWN
Vitvi019681	4.747879307	0.0000147	0.0000615	UP	Vitvi011450	-1.053728061	5.87E-33	1.97E-31	DOWN
Vitvi020580	4.743031577	4.77E-53	4.19E-51	UP	Vitvi008164	-1.053821004	3.52E-24	7.28E-23	DOWN
Vitvi035159	4.73554439	0.0003222	0.001094767	UP	Vitvi025671	-1.053839585	1.58E-14	1.65E-13	DOWN
Vitvi034587	4.719667359	2.57E-54	2.35E-52	UP	Vitvi018018	-1.054211426	0.00000013	0.000000713	DOWN
Vitvi016835	4.716686637	6.74E-23	1.28E-21	UP	Vitvi024028	-1.054498326	0.000126216	0.000456987	DOWN
Vitvi011592	4.709891975	4.55E-82	1.14E-79	UP	Vitvi030140	-1.054661921	1.29E-14	1.36E-13	DOWN
Vitvi010905	4.681177915	0.001112192	0.003442676	UP	Vitvi016143	-1.054664942	0.0000192	0.0000788	DOWN
Vitvi006668	4.678150691	1.06E-50	8.25E-49	UP	Vitvi011609	-1.054853613	0.000000118	0.000000648	DOWN
Vitvi001829	4.66279811	6.12E-124	4.44E-121	UP	Vitvi004283	-1.054913031	1.02E-12	9.28E-12	DOWN
Vitvi009460	4.644940094	3.85E-22	6.99E-21	UP	Vitvi029069	-1.055322807	0.000663208	0.002139285	DOWN
Vitvi005417	4.625099312	0.000494801	0.001632167	UP	Vitvi033095	-1.055401596	8.29E-11	6.24E-10	DOWN
Vitvi019296	4.561992905	0.000143308	0.000514015	UP	Vitvi001434	-1.056169793	1.08E-09	7.37E-09	DOWN
Vitvi018869	4.560291725	0.000047	0.000182071	UP	Vitvi030305	-1.058679212	5.18E-14	5.25E-13	DOWN
Vitvi018857	4.549488067	4.76E-136	4.6E-133	UP	Vitvi020925	-1.059591077	0.000000263	0.00000139	DOWN

ID	log2FoldChange	pvalue	padj	change	ID	log2FoldChange	pvalue	padj	change
Vitvi012369	4.546836366	0.00053003	0.00173841	UP	Vitvi017781	-1.059892706	0.001010072	0.003152516	DOWN
Vitvi006095	4.545354754	0.0000657	0.000248474	UP	Vitvi019944	-1.060066451	0.012492683	0.030679649	DOWN
Vitvi036980	4.516507791	0.0000831	0.000309697	UP	Vitvi032300	-1.060114777	2.41E-20	3.93E-19	DOWN
Vitvi004090	4.513060172	0.000655372	0.002116363	UP	Vitvi016678	-1.060409012	0.008420395	0.021598064	DOWN
Vitvi000094	4.512521395	0.000201915	0.000708462	UP	Vitvi033298	-1.060511442	5.18E-32	1.67E-30	DOWN
Vitvi014382	4.502582303	2.88E-10	2.06E-09	UP	Vitvi012192	-1.06059234	0.00000955	0.0000409	DOWN
Vitvi016834	4.493116176	0.000092	0.00034052	UP	Vitvi009470	-1.060763708	0.000148033	0.000530028	DOWN
Vitvi029306	4.486521858	7.96E-29	2.12E-27	UP	Vitvi021071	-1.060960946	0.000153987	0.00054989	DOWN
Vitvi028908	4.486147139	2.02E-13	1.93E-12	UP	Vitvi015881	-1.061049912	0.000000004	2.58E-08	DOWN
Vitvi021576	4.467642758	0.000584777	0.00190277	UP	Vitvi037429	-1.061303967	1.19E-33	4.1E-32	DOWN
Vitvi018422	4.464995828	0.000273003	0.000939392	UP	Vitvi015377	-1.061951444	0.000267582	0.00092199	DOWN
Vitvi020477	4.464660714	6.71E-10	4.67E-09	UP	Vitvi027704	-1.062242113	2.01E-15	2.24E-14	DOWN
Vitvi017532	4.464297701	0.000166735	0.000593109	UP	Vitvi011726	-1.062358336	0.001385839	0.004218367	DOWN
Vitvi004181	4.460343656	0.000888894	0.002802299	UP	Vitvi025328	-1.06240282	1.11E-16	1.38E-15	DOWN
Vitvi000287	4.449152436	3.63E-22	6.61E-21	UP	Vitvi023164	-1.062656831	5.63E-23	1.08E-21	DOWN
Vitvi007280	4.448609025	0.000638269	0.002064741	UP	Vitvi002110	-1.063323136	1.09E-36	4.34E-35	DOWN
Vitvi021673	4.40966452	0.000310082	0.00105642	UP	Vitvi010243	-1.063580074	1.62E-14	1.7E-13	DOWN
Vitvi014371	4.406486542	0.000218783	0.000763955	UP	Vitvi012099	-1.064879875	7.26E-08	0.000000407	DOWN
Vitvi021617	4.405680286	1.49E-14	1.57E-13	UP	Vitvi004690	-1.064981435	0.002886331	0.008185862	DOWN
Vitvi028389	4.397505996	2.25E-31	6.95E-30	UP	Vitvi001949	-1.06524785	6.53E-11	4.97E-10	DOWN
Vitvi003316	4.397224936	0.000239925	0.000833479	UP	Vitvi001133	-1.066164218	0.0010157	0.003168623	DOWN
Vitvi026019	4.38086857	1.11E-21	1.97E-20	UP	Vitvi031663	-1.066358668	0.006894958	0.017996708	DOWN
Vitvi001951	4.380321926	1.74E-67	2.7E-65	UP	Vitvi030602	-1.066478989	0.000000016	9.63E-08	DOWN
Vitvi004705	4.373473923	0.000570079	0.001858063	UP	Vitvi011511	-1.066535301	9.44E-16	1.08E-14	DOWN
Vitvi007518	4.371501568	0.000837081	0.002650474	UP	Vitvi013448	-1.067121505	3.61E-44	2.02E-42	DOWN
Vitvi019696	4.367939622	0.000980476	0.003066272	UP	Vitvi002163	-1.067940707	0.0000441	0.000171206	DOWN
Vitvi006752	4.36505233	5.01E-142	5.66E-139	UP	Vitvi005890	-1.068428957	1.43E-22	2.65E-21	DOWN
Vitvi036203	4.360948127	0.000287897	0.000985632	UP	Vitvi010176	-1.068623891	0.01986351	0.046096954	DOWN
Vitvi020767	4.359564328	0.002671637	0.007631316	UP	Vitvi026057	-1.069090645	2.21E-12	4.36E-10	DOWN
Vitvi032539	4.357324169	0.001002794	0.003130283	UP	Vitvi005331	-1.069911927	1.53E-18	2.17E-17	DOWN
Vitvi002166	4.35416029	0.000298072	0.001018581	UP	Vitvi031149	-1.071114294	5.6E-22	1.01E-20	DOWN
Vitvi018157	4.34940106	3.88E-155	6.56E-152	UP	Vitvi025646	-1.071977633	0.003040408	0.008583288	DOWN
Vitvi034074	4.330671494	3.89E-18	5.33E-17	UP	Vitvi024161	-1.07235515	0.002706707	0.007725887	DOWN
Vitvi019291	4.325812214	0.000485888	0.001605532	UP	Vitvi018476	-1.07475367	0.001473306	0.023578991	DOWN
Vitvi012584	4.32092916	1.02E-34	3.66E-33	UP	Vitvi009474	-1.075474157	1.43E-21	2.52E-20	DOWN
Vitvi017894	4.316136872	1.21E-78	2.68E-76	UP	Vitvi011210	-1.075549566	0.003955804	0.010920126	DOWN
Vitvi007984	4.314474571	0.000000028	0.000000165	UP	Vitvi031661	-1.075912482	8.54E-21	1.43E-19	DOWN
Vitvi006671	4.314178497	0.0000088	0.000038	UP	Vitvi020901	-1.076288996	0.00000863	0.000375137	DOWN
Vitvi014889	4.306649782	1.78E-18	2.5E-17	UP	Vitvi033154	-1.077261642	2.05E-11	1.65E-10	DOWN
Vitvi032894	4.297735627	2.04E-59	2.35E-57	UP	Vitvi001918	-1.077305381	0.00000204	0.00000961	DOWN
Vitvi000375	4.29036501	0.000235031	0.000817879	UP	Vitvi023306	-1.078127093	0.0000311	0.000123868	DOWN
Vitvi023254	4.287082568	8.36E-45	4.91E-43	UP	Vitvi029759	-1.080080179	1.15E-08	7.02E-08	DOWN
Vitvi013420	4.283450337	9.42E-11	7.06E-10	UP	Vitvi010289	-1.080649522	1.77E-08	0.00000172	DOWN
Vitvi004667	4.282255994	0.001265803	0.003879148	UP	Vitvi033934	-1.080731022	9.02E-10	6.23E-09	DOWN
Vitvi030445	4.280997578	0.001939653	0.005708234	UP	Vitvi019555	-1.080918279	0.016625712	0.039437623	DOWN
Vitvi028944	4.277544522	0.001278626	0.00391608	UP	Vitvi009763	-1.080995034	0.0000498	0.000191678	DOWN
Vitvi013749	4.276237307	2.58E-13	2.46E-12	UP	Vitvi032115	-1.082093762	2.49E-12	2.18E-11	DOWN
Vitvi020110	4.274170715	2.49E-155	4.6E-152	UP	Vitvi004349	-1.082685295	0.00000239	0.0000111	DOWN
Vitvi005030	4.272672132	0.00000487	0.0000217	UP	Vitvi018464	-1.082857445	2.62E-31	8.07E-30	DOWN
Vitvi027611	4.268293329	2.01E-103	8.68E-101	UP	Vitvi005307	-1.083273167	4.25E-27	1.03E-25	DOWN
Vitvi004582	4.266894843	1.56E-160	3.17E-157	UP	Vitvi036216	-1.083353914	1.92E-08	0.000000115	DOWN
Vitvi020753	4.259361777	0.001933414	0.005692347	UP	Vitvi014903	-1.083414432	5.12E-09	3.27E-08	DOWN
Vitvi022784	4.252583417	0.000178837	0.000633398	UP	Vitvi021462	-1.083470437	0.000136298	0.000490693	DOWN
Vitvi015586	4.247817268	2.38E-28	6.19E-27	UP	Vitvi013035	-1.083511971	9.67E-35	3.48E-33	DOWN
Vitvi000876	4.244734981	0.002075753	0.006077943	UP	Vitvi010937	-1.083550578	5.1E-19	7.48E-18	DOWN
Vitvi000359	4.238088654	7.82E-28	1.98E-26	UP	Vitvi010720	-1.08374398	0.00000152	0.00000724	DOWN
Vitvi035817	4.23243652	2.44E-20	3.97E-19	UP	Vitvi011198	-1.084277773	6.51E-29	1.73E-27	DOWN

ID	log2FoldChange	pvalue	padj	change	ID	log2FoldChange	pvalue	padj	change
Vitvi011713	4.219225932	2.13E-10	1.55E-09	UP	Vitvi019535	-1.084287235	7.74E-19	1.11E-17	DOWN
Vitvi013636	4.212113626	5.93E-177	1.72E-173	UP	Vitvi032725	-1.084372277	0.003085977	0.00869381	DOWN
Vitvi028826	4.201112882	1.1E-13	1.08E-12	UP	Vitvi023021	-1.08668579	9.77E-22	1.74E-20	DOWN
Vitvi016342	4.186029344	8.51E-62	1.06E-59	UP	Vitvi017714	-1.087441472	0.000000819	0.00000405	DOWN
Vitvi007780	4.183740088	0.001921248	0.005658988	UP	Vitvi027787	-1.087924673	1.77E-26	4.15E-25	DOWN
Vitvi000981	4.181146887	0.001852411	0.00548167	UP	Vitvi031099	-1.088041177	7.42E-25	1.6E-23	DOWN
Vitvi003186	4.180161572	0.000223605	0.000779719	UP	Vitvi014702	-1.088597327	0.001190711	0.003661174	DOWN
Vitvi008054	4.176901251	0.002081782	0.006093841	UP	Vitvi002104	-1.089431452	0.00000414	0.0000186	DOWN
Vitvi001848	4.176788156	0.002496173	0.007174512	UP	Vitvi035091	-1.089600373	1.02E-08	6.26E-08	DOWN
Vitvi024271	4.174548736	0.000266668	0.000919308	UP	Vitvi033099	-1.090847175	2.69E-25	5.93E-24	DOWN
Vitvi014212	4.168571619	0.000000232	0.00000123	UP	Vitvi018095	-1.090877713	0.00000121	0.00000583	DOWN
Vitvi001695	4.166800472	0.00443924	0.012111463	UP	Vitvi031383	-1.092693543	2.62E-13	2.49E-12	DOWN
Vitvi020898	4.165367416	0.00065947	0.002128243	UP	Vitvi007069	-1.092963583	0.00000486	0.0000217	DOWN
Vitvi014364	4.158835953	1.69E-08	0.000000101	UP	Vitvi030116	-1.092980066	5.7E-20	9.04E-19	DOWN
Vitvi007983	4.149138723	1.08E-09	7.4E-09	UP	Vitvi026859	-1.094399388	2E-12	1.77E-11	DOWN
Vitvi023422	4.149055283	1.66E-20	2.73E-19	UP	Vitvi000243	-1.094464615	7.84E-08	0.000000438	DOWN
Vitvi007395	4.134786251	8.77E-37	3.52E-35	UP	Vitvi036004	-1.096200067	1.15E-25	2.58E-24	DOWN
Vitvi015690	4.126521	1.8E-26	4.22E-25	UP	Vitvi005622	-1.097116432	2.79E-09	1.83E-08	DOWN
Vitvi019850	4.118122953	6.62E-169	1.68E-165	UP	Vitvi031891	-1.097159774	0.000000108	0.000000597	DOWN
Vitvi000304	4.110653984	0.000742552	0.002377478	UP	Vitvi018045	-1.098622473	0.000281694	0.000966188	DOWN
Vitvi009205	4.098563671	1.11E-287	2.25E-283	UP	Vitvi002472	-1.099221535	1.95E-11	1.57E-10	DOWN
Vitvi034574	4.086584896	1.02E-28	2.7E-27	UP	Vitvi010127	-1.099344645	1.21E-13	1.19E-12	DOWN
Vitvi028089	4.08168444	0.002954065	0.008361754	UP	Vitvi026986	-1.100058236	0.020272798	0.046862533	DOWN
Vitvi031175	4.072460408	0.002834199	0.008052628	UP	Vitvi029323	-1.100729327	2.39E-14	2.48E-13	DOWN
Vitvi014533	4.071475856	0.003977684	0.010967111	UP	Vitvi035831	-1.100953543	3.49E-19	5.18E-18	DOWN
Vitvi002432	4.06687852	6.89E-38	2.9E-36	UP	Vitvi017509	-1.101457883	0.01167981	0.028914113	DOWN
Vitvi018587	4.066655603	0.003775069	0.010461008	UP	Vitvi011001	-1.101513875	0.000000825	0.00000408	DOWN
Vitvi008071	4.062476249	5.01E-70	8.62E-68	UP	Vitvi010035	-1.101658277	0.00000103	0.00000503	DOWN
Vitvi019576	4.044946766	0.0000157	0.0000653	UP	Vitvi014737	-1.102160043	0.00000167	0.00000795	DOWN
Vitvi007041	4.038536682	0.001023388	0.003190157	UP	Vitvi012187	-1.103295155	1.36E-09	9.23E-09	DOWN
Vitvi031853	4.030205612	6.09E-16	7.08E-15	UP	Vitvi019591	-1.103554481	1.27E-16	1.56E-15	DOWN
Vitvi024612	4.014556993	9.19E-26	2.08E-24	UP	Vitvi031791	-1.103704443	6.81E-13	6.29E-12	DOWN
Vitvi017334	3.992674663	1.23E-29	3.44E-28	UP	Vitvi007400	-1.104202319	1.84E-31	5.73E-30	DOWN
Vitvi013419	3.990557984	3.66E-99	1.46E-96	UP	Vitvi010319	-1.104324979	1.63E-10	1.2E-09	DOWN
Vitvi036425	3.982905758	0.002581248	0.00739601	UP	Vitvi030292	-1.104610519	4.61E-12	3.94E-11	DOWN
Vitvi022334	3.977546801	0.006998706	0.018234701	UP	Vitvi023957	-1.10461918	2.43E-08	0.000000144	DOWN
Vitvi008115	3.974708929	5.32E-56	5.27E-54	UP	Vitvi023224	-1.106409971	1.81E-24	3.8E-23	DOWN
Vitvi005763	3.969277251	0.005487001	0.014630081	UP	Vitvi005764	-1.106554054	0.00000387	0.0000175	DOWN
Vitvi016195	3.959144264	4.42E-67	6.8E-65	UP	Vitvi037221	-1.107308153	0.00000044	0.00000226	DOWN
Vitvi034342	3.948818663	0.00494256	0.01332713	UP	Vitvi009540	-1.107521081	5.11E-18	6.95E-17	DOWN
Vitvi034069	3.94782189	0.005508519	0.014679747	UP	Vitvi029792	-1.108072534	0.005433782	0.014514858	DOWN
Vitvi003671	3.946931507	0.004685514	0.012708123	UP	Vitvi027310	-1.108416156	0.004605704	0.012523568	DOWN
Vitvi018777	3.94029064	0.006133517	0.016173439	UP	Vitvi031996	-1.108806999	8.73E-15	9.31E-14	DOWN
Vitvi004665	3.93629332	0.012657887	0.031014114	UP	Vitvi033774	-1.109069599	1.66E-17	2.17E-16	DOWN
Vitvi017303	3.930624564	0.000000162	0.000000876	UP	Vitvi005272	-1.109249462	0.001108278	0.003431085	DOWN
Vitvi029311	3.928339648	0.0000328	0.000129893	UP	Vitvi021351	-1.109434968	2.69E-21	4.63E-20	DOWN
Vitvi013099	3.926141477	0.010195056	0.025635302	UP	Vitvi012506	-1.109639566	0.001910062	0.005630125	DOWN
Vitvi001031	3.91738113	1.34E-08	8.11E-08	UP	Vitvi015574	-1.110680432	1.66E-13	1.61E-12	DOWN
Vitvi015373	3.915883073	1.61E-15	1.8E-14	UP	Vitvi022389	-1.110906789	0.0000231	0.0000938	DOWN
Vitvi028776	3.911500983	1.34E-20	2.21E-19	UP	Vitvi025642	-1.111241149	1.33E-19	2.04E-18	DOWN
Vitvi027623	3.910381525	0.002093256	0.006124969	UP	Vitvi021047	-1.111523317	0.00000344	0.0000157	DOWN
Vitvi026096	3.907452835	0.0000376	0.000147546	UP	Vitvi037275	-1.111851332	0.000799111	0.002541737	DOWN
Vitvi023178	3.907081563	0.002034794	0.005964893	UP	Vitvi002953	-1.112565553	5.04E-09	3.22E-08	DOWN
Vitvi028199	3.906666152	0.002260364	0.006558931	UP	Vitvi011937	-1.112568796	0.000890848	0.002808024	DOWN
Vitvi036614	3.887891128	7.18E-43	3.75E-41	UP	Vitvi005701	-1.11311196	1.32E-42	6.81E-41	DOWN
Vitvi011209	3.887708782	5.1E-28	1.3E-26	UP	Vitvi014510	-1.113142244	0.000000566	0.00000286	DOWN
Vitvi011415	3.882009468	3.01E-41	1.45E-39	UP	Vitvi021225	-1.113536036	1.91E-13	1.83E-12	DOWN

ID	log2FoldChange	pvalue	padj	change	ID	log2FoldChange	pvalue	padj	change
Vitvi035471	3.88142304	0.000000825	0.00000408	UP	Vitvi016910	-1.113817083	4.96E-10	3.49E-09	DOWN
Vitvi036118	3.881115752	0.000000703	0.0000035	UP	Vitvi005370	-1.113837737	3.95E-09	2.55E-08	DOWN
Vitvi034461	3.863693639	0.015797978	0.03767624	UP	Vitvi036534	-1.115103081	9.43E-11	7.07E-10	DOWN
Vitvi033546	3.862569123	0.000049	0.000188898	UP	Vitvi027392	-1.11516189	9.16E-20	1.42E-18	DOWN
Vitvi002429	3.862415807	6.5E-44	3.58E-42	UP	Vitvi013871	-1.115488133	0.0000194	0.0000797	DOWN
Vitvi031493	3.860898668	4.03E-98	1.54E-95	UP	Vitvi007632	-1.115840668	1.58E-17	2.07E-16	DOWN
Vitvi034411	3.852453349	1.28E-09	8.72E-09	UP	Vitvi033153	-1.11586996	0.000000583	0.00000294	DOWN
Vitvi013176	3.85236682	0.018850649	0.044020969	UP	Vitvi023338	-1.115955869	1.18E-08	0.000000072	DOWN
Vitvi010775	3.848015988	6.96E-22	1.25E-20	UP	Vitvi031381	-1.116089748	0.0000679	0.0002561	DOWN
Vitvi032834	3.841203792	4.4E-130	3.72E-127	UP	Vitvi014850	-1.116456651	6.36E-43	3.33E-41	DOWN
Vitvi017810	3.840641396	3.48E-29	9.43E-28	UP	Vitvi029012	-1.116871065	2.34E-08	0.000000139	DOWN
Vitvi002277	3.83903232	3.65E-36	1.42E-34	UP	Vitvi003708	-1.116888486	0.00000025	0.00000132	DOWN
Vitvi023188	3.8300277	0.007374613	0.019125784	UP	Vitvi007837	-1.117056087	1.1E-09	7.5E-09	DOWN
Vitvi027620	3.829848537	9.84E-16	1.12E-14	UP	Vitvi020116	-1.117499677	4.17E-17	5.31E-16	DOWN
Vitvi005010	3.828801997	0.00000632	0.0000278	UP	Vitvi011907	-1.117618742	1.33E-10	9.82E-10	DOWN
Vitvi016784	3.82209609	0.010452845	0.026218596	UP	Vitvi027304	-1.11763778	0.00000107	0.00000519	DOWN
Vitvi017849	3.819659973	1.51E-36	5.97E-35	UP	Vitvi002085	-1.11800445	1.77E-19	2.68E-18	DOWN
Vitvi021190	3.817698305	0.000000024	0.000000142	UP	Vitvi015489	-1.119201061	0.007094261	0.018462348	DOWN
Vitvi009189	3.814115371	4.04E-08	0.000000233	UP	Vitvi023727	-1.119575865	1.8E-19	2.72E-18	DOWN
Vitvi033169	3.808820814	0.000000429	0.00000221	UP	Vitvi030557	-1.119856022	1.03E-24	2.21E-23	DOWN
Vitvi003948	3.806320901	0.009337082	0.023706851	UP	Vitvi026071	-1.11993708	0.00000261	0.0000121	DOWN
Vitvi033674	3.792145729	5.89E-08	0.000000333	UP	Vitvi028377	-1.120374947	5.07E-10	3.57E-09	DOWN
Vitvi005541	3.786395683	1.23E-64	1.63E-62	UP	Vitvi014623	-1.122295422	0.0000321	0.000127512	DOWN
Vitvi017811	3.77760981	4.31E-98	1.62E-95	UP	Vitvi029962	-1.122454837	5.3E-10	3.72E-09	DOWN
Vitvi029658	3.776896715	2.22E-44	1.27E-42	UP	Vitvi013482	-1.12344866	0.000166761	0.000593109	DOWN
Vitvi033637	3.77000016	0.00000876	0.0000378	UP	Vitvi021158	-1.124308859	0.000677913	0.002182213	DOWN
Vitvi025631	3.769866801	1.37E-27	3.42E-26	UP	Vitvi032036	-1.124357665	1.44E-15	1.62E-14	DOWN
Vitvi028624	3.767312891	3.24E-09	2.11E-08	UP	Vitvi030614	-1.126173006	0.000000331	0.00000172	DOWN
Vitvi031732	3.751234204	0.003787869	0.010490754	UP	Vitvi027458	-1.126478477	0.000000374	0.00000194	DOWN
Vitvi009463	3.718407908	9.17E-10	6.33E-09	UP	Vitvi017880	-1.126678745	1.02E-41	5.07E-40	DOWN
Vitvi036105	3.714277219	6.94E-15	7.45E-14	UP	Vitvi027760	-1.12777662	1.44E-26	3.4E-25	DOWN
Vitvi016307	3.71329966	0.014155714	0.034233939	UP	Vitvi036391	-1.127777048	0.00000238	0.0000111	DOWN
Vitvi011854	3.707990835	0.012473497	0.030639941	UP	Vitvi005038	-1.127778206	2.61E-10	1.88E-09	DOWN
Vitvi001875	3.706907443	0.012503366	0.030702174	UP	Vitvi025683	-1.128493855	6.76E-30	1.92E-28	DOWN
Vitvi022618	3.706364795	2.38E-122	1.61E-119	UP	Vitvi026874	-1.128632946	3.47E-15	3.82E-14	DOWN
Vitvi027487	3.70368516	2.11E-18	2.94E-17	UP	Vitvi014589	-1.129147669	2.49E-16	3E-15	DOWN
Vitvi009962	3.700568506	0.012674845	0.031048174	UP	Vitvi035926	-1.1292342	0.000124843	0.00045242	DOWN
Vitvi034330	3.69968713	0.012699452	0.031097199	UP	Vitvi004578	-1.129595291	9.74E-08	0.000000539	DOWN
Vitvi021371	3.696923827	0.011100859	0.027615511	UP	Vitvi017590	-1.129915729	1.54E-13	1.5E-12	DOWN
Vitvi005778	3.695082423	8.95E-09	5.54E-08	UP	Vitvi003707	-1.129969857	0.00000103	0.00000503	DOWN
Vitvi025768	3.691243257	0.020504684	0.047309774	UP	Vitvi032675	-1.130272945	0.00000115	0.00000555	DOWN
Vitvi014276	3.688053501	1.89E-25	4.2E-24	UP	Vitvi013842	-1.131436885	0.000191633	0.0006746	DOWN
Vitvi027635	3.685977621	0.013087235	0.031919778	UP	Vitvi015905	-1.131459157	0.000733844	0.002352192	DOWN
Vitvi009392	3.685879451	0.002186013	0.006367745	UP	Vitvi028450	-1.131740386	0.00000203	0.00000956	DOWN
Vitvi012312	3.67657641	0.012185896	0.03002425	UP	Vitvi018540	-1.132010646	4.92E-09	3.15E-08	DOWN
Vitvi009292	3.673337001	0.000227872	0.000794188	UP	Vitvi018737	-1.132151112	6.01E-18	8.12E-17	DOWN
Vitvi011596	3.667705602	0.001997575	0.00586425	UP	Vitvi035721	-1.133031823	1.01E-24	2.17E-23	DOWN
Vitvi034075	3.663630742	0.0000335	0.000132436	UP	Vitvi013422	-1.133140075	2.15E-09	1.43E-08	DOWN
Vitvi030493	3.66202045	0.021779731	0.04989735	UP	Vitvi029162	-1.13328912	2.08E-20	3.4E-19	DOWN
Vitvi035424	3.658733261	0.011948042	0.029499044	UP	Vitvi017687	-1.134288398	0.003651448	0.010141968	DOWN
Vitvi012940	3.653728131	0.017974656	0.042213113	UP	Vitvi014997	-1.13449098	2.21E-20	3.62E-19	DOWN
Vitvi029098	3.646893534	6.22E-24	1.25E-22	UP	Vitvi008144	-1.134604825	0.00000293	0.0000135	DOWN
Vitvi012516	3.645797218	0.005836131	0.015475651	UP	Vitvi025660	-1.134693774	4.11E-10	2.91E-09	DOWN
Vitvi000034	3.645139986	2.71E-52	2.28E-50	UP	Vitvi030111	-1.135280049	3.86E-08	0.000000224	DOWN
Vitvi006547	3.639240044	9.58E-57	9.87E-55	UP	Vitvi027591	-1.135744883	2.9E-11	2.29E-10	DOWN
Vitvi022834	3.623724051	2.42E-12	2.13E-11	UP	Vitvi014953	-1.135759127	4.27E-10	3.02E-09	DOWN
Vitvi011869	3.621837499	7.06E-13	6.52E-12	UP	Vitvi018914	-1.13642432	1.04E-20	1.72E-19	DOWN

ID	log2FoldChange	pvalue	padj	change	ID	log2FoldChange	pvalue	padj	change
Vitvi027447	3.60614428	5.67E-12	4.81E-11	UP	Vitvi021229	-1.137070758	0.000000574	0.0000029	DOWN
Vitvi031141	3.60479521	1.68E-66	2.5E-64	UP	Vitvi024342	-1.137208483	0.005460081	0.014575526	DOWN
Vitvi033601	3.604523911	2.18E-81	5.21E-79	UP	Vitvi005951	-1.137384684	0.0000316	0.000125746	DOWN
Vitvi029203	3.5881571	7.91E-38	3.33E-36	UP	Vitvi009381	-1.1379007	0.001134238	0.003504508	DOWN
Vitvi007283	3.586103958	1.06E-145	1.27E-142	UP	Vitvi023170	-1.13909696	0.0000394	0.000153895	DOWN
Vitvi019286	3.574453635	0.002398691	0.006916838	UP	Vitvi021138	-1.13913641	0.0000618	0.000234727	DOWN
Vitvi000224	3.571618306	0.00033863	0.001146566	UP	Vitvi002184	-1.139186568	4.35E-19	6.43E-18	DOWN
Vitvi015128	3.562995508	0.006785687	0.017745702	UP	Vitvi032163	-1.139353024	6.08E-25	1.32E-23	DOWN
Vitvi014978	3.5581118	0.000000126	0.00000069	UP	Vitvi019773	-1.139523044	0.007348994	0.019066645	DOWN
Vitvi004698	3.556979956	0.006863841	0.017922399	UP	Vitvi032404	-1.140078335	3.03E-12	2.64E-11	DOWN
Vitvi007773	3.553438536	1.68E-230	1.71E-226	UP	Vitvi035391	-1.140267679	0.000488249	0.001612571	DOWN
Vitvi031463	3.551785794	8.77E-121	5.24E-118	UP	Vitvi031512	-1.140466194	0.006061832	0.016013516	DOWN
Vitvi001576	3.547867122	0.019992285	0.046351249	UP	Vitvi018503	-1.141016063	4.18E-35	1.56E-33	DOWN
Vitvi002719	3.546364773	0.019236143	0.044823281	UP	Vitvi001459	-1.141663715	0.000435462	0.001449305	DOWN
Vitvi007794	3.53951463	1.71E-57	1.84E-55	UP	Vitvi005600	-1.143089718	1.89E-12	1.68E-11	DOWN
Vitvi019997	3.538585901	2.16E-29	5.93E-28	UP	Vitvi035879	-1.143113584	7.03E-11	5.33E-10	DOWN
Vitvi003291	3.512825346	0.00000905	0.0000389	UP	Vitvi010718	-1.143252686	5.8E-11	4.45E-10	DOWN
Vitvi023059	3.504063941	1.03E-53	9.26E-52	UP	Vitvi035815	-1.143428233	3.62E-16	4.29E-15	DOWN
Vitvi000352	3.503085715	9.9E-14	9.79E-13	UP	Vitvi015249	-1.14429958	0.001114847	0.00345037	DOWN
Vitvi007675	3.48213396	0.000441782	0.001468174	UP	Vitvi000687	-1.144472269	1.11E-45	6.74E-44	DOWN
Vitvi011873	3.48147934	0.012826363	0.031373927	UP	Vitvi017805	-1.14461327	6.79E-20	1.07E-18	DOWN
Vitvi029088	3.476790771	0.0098659	0.02488466	UP	Vitvi000899	-1.145236567	0.000000968	0.00000473	DOWN
Vitvi007176	3.475070027	0.009758674	0.024644808	UP	Vitvi002226	-1.145327848	0.000161579	0.000575788	DOWN
Vitvi032553	3.472836014	0.000000022	0.000000131	UP	Vitvi009502	-1.145549976	9.82E-12	8.12E-11	DOWN
Vitvi005745	3.459223861	2.39E-70	4.14E-68	UP	Vitvi030999	-1.14678094	1.21E-10	9.02E-10	DOWN
Vitvi032886	3.456451313	1.17E-08	7.17E-08	UP	Vitvi008886	-1.146931973	0.00000063	0.00000316	DOWN
Vitvi018302	3.451461066	0.008965199	0.022865534	UP	Vitvi015952	-1.147982733	0.00000735	0.000032	DOWN
Vitvi002763	3.44800782	1.95E-54	1.8E-52	UP	Vitvi035664	-1.148295736	5.82E-18	7.87E-17	DOWN
Vitvi011881	3.4466495	2.66E-55	2.55E-53	UP	Vitvi013536	-1.149077143	0.008178095	0.02101904	DOWN
Vitvi033170	3.446410078	0.009268508	0.023550406	UP	Vitvi015118	-1.149225971	1.08E-12	9.8E-12	DOWN
Vitvi011575	3.441862263	0.00000602	0.0000266	UP	Vitvi001874	-1.149480205	5.15E-08	0.000000294	DOWN
Vitvi011486	3.441348022	2.32E-10	1.68E-09	UP	Vitvi017324	-1.149486338	0.004881107	0.013185935	DOWN
Vitvi031225	3.440843265	0.000269544	0.000928119	UP	Vitvi025720	-1.151848663	0.008003819	0.020610241	DOWN
Vitvi023364	3.43637936	7.73E-09	4.82E-08	UP	Vitvi014433	-1.152472861	4.93E-21	8.38E-20	DOWN
Vitvi008172	3.431556578	4.72E-78	1.03E-75	UP	Vitvi019759	-1.153406986	1.8E-19	2.73E-18	DOWN
Vitvi007828	3.425725631	1.57E-20	2.59E-19	UP	Vitvi031164	-1.153586619	0.0000022	0.0000103	DOWN
Vitvi025781	3.414103378	1.64E-13	1.59E-12	UP	Vitvi001007	-1.153984765	0.000000185	0.000000992	DOWN
Vitvi027121	3.410405308	1.51E-67	2.37E-65	UP	Vitvi004263	-1.154388822	7.92E-08	0.000000442	DOWN
Vitvi000231	3.4088121	2.38E-63	3.07E-61	UP	Vitvi028914	-1.154649496	0.00000015	0.000000814	DOWN
Vitvi031532	3.404059976	0.005480245	0.014617826	UP	Vitvi005159	-1.155678744	0.004686089	0.012708123	DOWN
Vitvi002589	3.402428411	6.96E-16	8.03E-15	UP	Vitvi021936	-1.155686448	1.33E-08	8.07E-08	DOWN
Vitvi009197	3.399739647	3.34E-08	0.000000194	UP	Vitvi007918	-1.155882382	1.98E-50	1.52E-48	DOWN
Vitvi020302	3.391867576	1.82E-47	1.24E-45	UP	Vitvi014962	-1.15626642	0.000000006	0.000000038	DOWN
Vitvi002027	3.387119283	3.02E-08	0.000000177	UP	Vitvi017579	-1.15709887	6.19E-35	2.27E-33	DOWN
Vitvi035859	3.38230652	0.0000252	0.000101833	UP	Vitvi016495	-1.159680617	1.44E-21	2.52E-20	DOWN
Vitvi026619	3.380794958	0.018815093	0.043963208	UP	Vitvi022528	-1.159687257	1.34E-12	1.21E-11	DOWN
Vitvi018347	3.378880913	3.38E-20	5.45E-19	UP	Vitvi004426	-1.160418293	0.00000205	0.00000964	DOWN
Vitvi021060	3.378531801	5.99E-59	6.84E-57	UP	Vitvi035276	-1.161219111	7.66E-18	1.03E-16	DOWN
Vitvi033361	3.378429864	2.18E-125	1.64E-122	UP	Vitvi016028	-1.161240097	0.001500476	0.023901416	DOWN
Vitvi002427	3.365200573	0.005910939	0.015655625	UP	Vitvi012834	-1.161614228	2.21E-16	2.68E-15	DOWN
Vitvi020986	3.357120568	2.14E-10	1.55E-09	UP	Vitvi032713	-1.162613497	1.45E-31	4.54E-30	DOWN
Vitvi019320	3.350997375	8.97E-13	8.21E-12	UP	Vitvi032158	-1.163064134	2.33E-17	3.03E-16	DOWN
Vitvi031542	3.350934362	2.33E-09	1.54E-08	UP	Vitvi035898	-1.163486095	0.001845157	0.005465776	DOWN
Vitvi020988	3.349692837	0.000126721	0.000458734	UP	Vitvi002305	-1.164359023	0.00000119	0.00000573	DOWN
Vitvi022621	3.343708929	0.013345608	0.032510913	UP	Vitvi015675	-1.164653354	0.001413312	0.004294912	DOWN
Vitvi005151	3.33925585	2.07E-27	5.09E-26	UP	Vitvi029211	-1.164782892	1.85E-32	6.1E-31	DOWN
Vitvi018643	3.332289407	0.0000375	0.00014727	UP	Vitvi000322	-1.165206371	0.000000841	0.00000415	DOWN

ID	log2FoldChange	pvalue	padj	change	ID	log2FoldChange	pvalue	padj	change
Vitvi006050	3.331889372	3.84E-26	8.85E-25	UP	Vitvi018346	-1.165638855	4.92E-29	1.32E-27	DOWN
Vitvi000222	3.329835311	6.14E-60	7.25E-58	UP	Vitvi021674	-1.166326729	0.002250932	0.006538746	DOWN
Vitvi029430	3.327556653	0.000000112	0.000000615	UP	Vitvi033974	-1.166445208	2.6E-29	7.14E-28	DOWN
Vitvi017867	3.327500542	0.014312229	0.034567182	UP	Vitvi029485	-1.167005423	0.000000174	0.000000934	DOWN
Vitvi002787	3.327284211	2.2E-29	6.04E-28	UP	Vitvi006767	-1.167034361	4.92E-21	8.38E-20	DOWN
Vitvi013149	3.326996535	5.83E-12	4.94E-11	UP	Vitvi023405	-1.167101421	0.0000454	0.000175983	DOWN
Vitvi036125	3.317802057	2.41E-75	4.8E-73	UP	Vitvi028720	-1.168683276	7.54E-08	0.000000422	DOWN
Vitvi036781	3.310465282	0.000194283	0.000683339	UP	Vitvi000862	-1.169342172	4.93E-27	1.19E-25	DOWN
Vitvi032817	3.304184349	3.19E-19	4.75E-18	UP	Vitvi030507	-1.170032162	0.018735495	0.0438125	DOWN
Vitvi006099	3.301847829	0.001314861	0.004017971	UP	Vitvi002126	-1.170149368	9.24E-30	2.6E-28	DOWN
Vitvi030898	3.296317588	0.000000608	0.00000306	UP	Vitvi017735	-1.170220927	1.08E-09	7.38E-09	DOWN
Vitvi027020	3.289637892	1.57E-18	2.22E-17	UP	Vitvi007747	-1.170590158	1.41E-23	2.77E-22	DOWN
Vitvi019433	3.280856176	0.0000301	0.000120068	UP	Vitvi035642	-1.171869827	1.96E-36	7.73E-35	DOWN
Vitvi025410	3.278728954	8.27E-08	0.000000461	UP	Vitvi013299	-1.172295825	1.9E-11	1.53E-10	DOWN
Vitvi023321	3.268892368	1.84E-13	1.78E-12	UP	Vitvi027736	-1.172428926	7.61E-44	4.16E-42	DOWN
Vitvi010828	3.26049801	1.6E-50	1.24E-48	UP	Vitvi021113	-1.172826597	6.16E-12	5.2E-11	DOWN
Vitvi013477	3.259889406	1.09E-147	1.38E-144	UP	Vitvi032874	-1.173098244	6.19E-15	6.68E-14	DOWN
Vitvi001185	3.258774622	0.000000778	0.00000386	UP	Vitvi036919	-1.173363979	1.92E-19	2.9E-18	DOWN
Vitvi026811	3.252645211	3.07E-86	9.04E-84	UP	Vitvi017801	-1.173505646	0.000129835	0.000469088	DOWN
Vitvi018137	3.249387318	6.91E-12	5.8E-11	UP	Vitvi036573	-1.173774889	2.81E-17	3.63E-16	DOWN
Vitvi030845	3.247365853	0.001679787	0.005024272	UP	Vitvi031860	-1.174219634	0.001360044	0.004146064	DOWN
Vitvi012694	3.235344947	0.01709454	0.040440751	UP	Vitvi000085	-1.17445791	2.83E-18	3.91E-17	DOWN
Vitvi001898	3.234601597	3.68E-16	4.36E-15	UP	Vitvi011112	-1.174919443	7.25E-36	2.76E-34	DOWN
Vitvi033859	3.22751332	0.000263711	0.000909733	UP	Vitvi036967	-1.175125884	0.0000837	0.000311646	DOWN
Vitvi024457	3.226772265	0.017200609	0.040663264	UP	Vitvi033319	-1.175706261	2.37E-56	2.41E-54	DOWN
Vitvi008040	3.224489757	4.25E-17	5.41E-16	UP	Vitvi027420	-1.175796839	4.95E-24	1.01E-22	DOWN
Vitvi018260	3.224325303	1.08E-44	6.32E-43	UP	Vitvi028271	-1.175830682	0.004118648	0.011314305	DOWN
Vitvi007241	3.224021277	0.017945714	0.042159767	UP	Vitvi035515	-1.176001924	0.000000421	0.00000217	DOWN
Vitvi033216	3.222266433	2.47E-48	1.72E-46	UP	Vitvi020230	-1.176185103	0.000000253	0.00000133	DOWN
Vitvi035301	3.21825963	8.97E-57	9.3E-55	UP	Vitvi018639	-1.176830142	0.00000046	0.00000236	DOWN
Vitvi013414	3.218062868	1.73E-21	3.02E-20	UP	Vitvi001791	-1.17697149	1.31E-45	7.91E-44	DOWN
Vitvi035967	3.214600753	0.002136895	0.006240779	UP	Vitvi027686	-1.177021548	5.27E-20	8.37E-19	DOWN
Vitvi026106	3.213469259	0.000267406	0.00092154	UP	Vitvi000519	-1.177311837	0.001447938	0.023275733	DOWN
Vitvi000335	3.206529881	0.0000564	0.000215556	UP	Vitvi036366	-1.177555386	0.018216645	0.042707352	DOWN
Vitvi019860	3.204063487	0.00000667	0.0000292	UP	Vitvi028805	-1.178184626	6.64E-12	5.58E-11	DOWN
Vitvi002430	3.202699515	2.32E-128	1.81E-125	UP	Vitvi032969	-1.179726039	0.00000274	0.0000127	DOWN
Vitvi011413	3.190252333	1.6E-96	5.91E-94	UP	Vitvi012800	-1.179770247	7.67E-46	4.68E-44	DOWN
Vitvi018214	3.181991132	1.69E-43	9.11E-42	UP	Vitvi031094	-1.179975859	0.00947289	0.024001695	DOWN
Vitvi019120	3.180725161	1.63E-08	9.77E-08	UP	Vitvi020469	-1.180363678	0.000335429	0.001136485	DOWN
Vitvi035974	3.17956267	2.57E-11	2.04E-10	UP	Vitvi033482	-1.180379344	0.011069847	0.02755186	DOWN
Vitvi031193	3.169820038	0.009902742	0.024971384	UP	Vitvi036220	-1.180478028	2.05E-09	1.37E-08	DOWN
Vitvi022619	3.163891796	0.0000101	0.0000433	UP	Vitvi012508	-1.180568645	9.95E-12	8.21E-11	DOWN
Vitvi033040	3.162046533	2.3E-38	9.88E-37	UP	Vitvi019298	-1.180659151	0.004143392	0.011371513	DOWN
Vitvi028787	3.160455867	2.24E-30	6.54E-29	UP	Vitvi023634	-1.180774743	6.66E-25	1.44E-23	DOWN
Vitvi028718	3.156498514	2.19E-72	4.08E-70	UP	Vitvi017602	-1.181692327	0.000000302	0.0000216	DOWN
Vitvi011244	3.153073189	2.08E-151	3.02E-148	UP	Vitvi020257	-1.181788247	2E-19	3.01E-18	DOWN
Vitvi031238	3.151597656	5.03E-32	1.62E-30	UP	Vitvi009672	-1.182784956	2.7E-13	2.56E-12	DOWN
Vitvi036204	3.148077562	6.69E-11	5.09E-10	UP	Vitvi032804	-1.182809226	0.00000931	0.0000399	DOWN
Vitvi009885	3.14683522	2.12E-09	1.41E-08	UP	Vitvi026450	-1.182992516	0.0000119	0.0000501	DOWN
Vitvi000916	3.142063994	1.14E-39	5.14E-38	UP	Vitvi012188	-1.183473336	0.000289606	0.000990983	DOWN
Vitvi001173	3.134382002	5.22E-27	1.26E-25	UP	Vitvi015087	-1.184016005	0.000000265	0.0000014	DOWN
Vitvi000152	3.132709611	7.03E-88	2.16E-85	UP	Vitvi032735	-1.184369416	1.16E-33	4.02E-32	DOWN
Vitvi006984	3.127021908	6.67E-83	1.74E-80	UP	Vitvi030559	-1.184831482	4.14E-13	3.87E-12	DOWN
Vitvi028390	3.124800162	0.0000217	0.0000883	UP	Vitvi017875	-1.185175163	0.000112181	0.000410047	DOWN
Vitvi035759	3.121008422	5.78E-40	2.64E-38	UP	Vitvi010135	-1.185266882	6.75E-15	7.26E-14	DOWN
Vitvi011192	3.115694099	2.63E-16	3.17E-15	UP	Vitvi022804	-1.186719082	0.003302354	0.009252057	DOWN
Vitvi006640	3.113679658	0.012233048	0.030115426	UP	Vitvi025743	-1.186906908	5.82E-10	4.07E-09	DOWN

ID	log2FoldChange	pvalue	padj	change	ID	log2FoldChange	pvalue	padj	change
Vitvi030563	3.112324037	0.000000295	0.00000154	UP	Vitvi002257	-1.18713642	0.0000152	0.0000636	DOWN
Vitvi025192	3.110378849	0.011955	0.029512634	UP	Vitvi012908	-1.188285698	1.29E-10	9.52E-10	DOWN
Vitvi004321	3.107716146	0.013059662	0.031860179	UP	Vitvi018616	-1.188627656	0.0000554	0.000211854	DOWN
Vitvi019698	3.107624571	1.78E-21	3.1E-20	UP	Vitvi015094	-1.189286702	2.42E-24	5.06E-23	DOWN
Vitvi008030	3.105416375	8.5E-65	1.14E-62	UP	Vitvi018922	-1.190203434	0.000448054	0.001487072	DOWN
Vitvi002359	3.104689217	0.002895648	0.008207702	UP	Vitvi030236	-1.191372082	0.000000702	0.0000035	DOWN
Vitvi009206	3.103240051	1.88E-177	6.36E-174	UP	Vitvi011559	-1.191595649	1.19E-29	3.34E-28	DOWN
Vitvi026815	3.102475029	8.93E-35	3.21E-33	UP	Vitvi001657	-1.191629939	0.007256939	0.018839846	DOWN
Vitvi012948	3.096098057	0.0000993	0.000366214	UP	Vitvi035962	-1.192273056	8.78E-17	1.09E-15	DOWN
Vitvi032406	3.092076827	4.98E-29	1.33E-27	UP	Vitvi013087	-1.192326602	0.00000619	0.0000273	DOWN
Vitvi007786	3.083731763	0.017235134	0.040732752	UP	Vitvi031417	-1.192376062	8.78E-08	0.000000488	DOWN
Vitvi013362	3.081984576	4.99E-18	6.79E-17	UP	Vitvi030616	-1.192880311	2.2E-11	1.76E-10	DOWN
Vitvi004538	3.079019779	5.19E-17	6.56E-16	UP	Vitvi030617	-1.193515169	0.000230147	0.000801844	DOWN
Vitvi011483	3.078183093	0.0000319	0.000126754	UP	Vitvi022951	-1.193756932	3.81E-10	2.71E-09	DOWN
Vitvi027229	3.075512787	4.39E-206	2.23E-202	UP	Vitvi022947	-1.194519556	1.85E-18	2.6E-17	DOWN
Vitvi000814	3.067883182	7.36E-12	6.17E-11	UP	Vitvi020656	-1.196098881	3.35E-09	2.18E-08	DOWN
Vitvi005028	3.06720365	1.63E-82	4.19E-80	UP	Vitvi006537	-1.196242648	3.69E-12	3.19E-11	DOWN
Vitvi015432	3.05862791	5.01E-90	1.64E-87	UP	Vitvi021115	-1.196377478	0.017640768	0.041550603	DOWN
Vitvi025613	3.05594402	3.2E-105	1.41E-102	UP	Vitvi005767	-1.196680379	3.86E-20	6.18E-19	DOWN
Vitvi036983	3.052219555	0.00000591	0.0000261	UP	Vitvi024899	-1.197629695	0.002786893	0.007934872	DOWN
Vitvi033653	3.046581111	1.37E-121	8.71E-119	UP	Vitvi032277	-1.197682484	2.89E-25	6.38E-24	DOWN
Vitvi036127	3.045920541	1.16E-10	8.61E-10	UP	Vitvi025857	-1.197998923	0.006938409	0.018100817	DOWN
Vitvi031464	3.045417804	2.67E-57	2.84E-55	UP	Vitvi017523	-1.198010499	4.95E-20	7.88E-19	DOWN
Vitvi018155	3.041287776	1.31E-20	2.17E-19	UP	Vitvi003800	-1.199199528	9.39E-14	9.32E-13	DOWN
Vitvi002466	3.040449925	0.000794911	0.002529567	UP	Vitvi032787	-1.199368173	1.13E-24	2.41E-23	DOWN
Vitvi007276	3.039025324	0.0000232	0.0000941	UP	Vitvi029062	-1.200677425	0.000026	0.000104712	DOWN
Vitvi009345	3.027514257	0.021305666	0.048927155	UP	Vitvi028244	-1.200889939	0.002250961	0.006538746	DOWN
Vitvi032020	3.02698889	3.72E-08	0.000000216	UP	Vitvi004211	-1.201696565	0.000706668	0.0022701	DOWN
Vitvi033296	3.025110789	0.005326895	0.01425982	UP	Vitvi010710	-1.201770775	1.53E-12	1.37E-11	DOWN
Vitvi000666	3.022943501	1.69E-83	4.51E-81	UP	Vitvi019722	-1.201824388	1.29E-32	4.32E-31	DOWN
Vitvi003436	3.017004474	0.000000891	0.00000438	UP	Vitvi036012	-1.201891728	1.23E-08	7.48E-08	DOWN
Vitvi022623	3.016742802	0.000000023	0.000000137	UP	Vitvi028408	-1.201964555	3.98E-10	2.82E-09	DOWN
Vitvi002528	3.016290631	1.49E-21	2.61E-20	UP	Vitvi033608	-1.202061441	0.006851198	0.017898589	DOWN
Vitvi033138	3.010151873	6.17E-35	2.26E-33	UP	Vitvi031999	-1.202147732	9.45E-13	8.63E-12	DOWN
Vitvi012812	3.00576867	4.83E-14	4.91E-13	UP	Vitvi000792	-1.203213591	0.002020558	0.005928293	DOWN
Vitvi012513	3.001637899	7.25E-09	4.54E-08	UP	Vitvi014299	-1.203992116	0.000000948	0.00000465	DOWN
Vitvi021760	2.986841504	2.25E-12	1.98E-11	UP	Vitvi002413	-1.204061521	3.27E-09	2.13E-08	DOWN
Vitvi003549	2.977613312	0.000263877	0.000910148	UP	Vitvi015872	-1.204977669	0.000188345	0.000664188	DOWN
Vitvi003542	2.974464037	4.2E-154	6.56E-151	UP	Vitvi023210	-1.205217305	0.00855953	0.021918957	DOWN
Vitvi031604	2.973906558	0.00000261	0.0000121	UP	Vitvi034319	-1.20543171	0.0000277	0.000110932	DOWN
Vitvi011094	2.971957061	1.19E-17	1.58E-16	UP	Vitvi003551	-1.20652364	1.08E-33	3.76E-32	DOWN
Vitvi000915	2.970525618	2.52E-09	1.66E-08	UP	Vitvi023463	-1.206883045	2.62E-11	2.08E-10	DOWN
Vitvi020983	2.969772653	0.00000465	0.0000208	UP	Vitvi011780	-1.2073874	5.59E-30	1.6E-28	DOWN
Vitvi019818	2.96762917	1.1E-24	2.35E-23	UP	Vitvi006514	-1.207600645	6.02E-23	1.15E-21	DOWN
Vitvi031015	2.965625239	8.54E-14	8.53E-13	UP	Vitvi024544	-1.208206742	0.003944518	0.010891933	DOWN
Vitvi012019	2.964873778	1.31E-28	3.44E-27	UP	Vitvi003547	-1.208314473	6.85E-08	0.000000384	DOWN
Vitvi028672	2.963008065	5.17E-18	7.02E-17	UP	Vitvi025522	-1.208683382	0.000000665	0.00000333	DOWN
Vitvi017540	2.961380734	2.66E-109	1.38E-106	UP	Vitvi010529	-1.209386781	1.15E-15	1.31E-14	DOWN
Vitvi004385	2.95972056	7.85E-19	1.13E-17	UP	Vitvi020102	-1.210463411	9.95E-23	1.86E-21	DOWN
Vitvi028859	2.957415789	0.000376616	0.001266527	UP	Vitvi027410	-1.210504363	1.07E-09	7.31E-09	DOWN
Vitvi021451	2.955247764	7.78E-18	1.04E-16	UP	Vitvi002263	-1.21057216	5.48E-26	1.25E-24	DOWN
Vitvi007036	2.9494788	0.000000354	0.00000184	UP	Vitvi000400	-1.210755508	1.67E-08	0.0000001	DOWN
Vitvi019588	2.948393034	0.00010658	0.00039126	UP	Vitvi010725	-1.211044334	0.012460277	0.030614871	DOWN
Vitvi005118	2.947079949	4.54E-66	6.36E-64	UP	Vitvi011385	-1.211119909	5.54E-40	2.54E-38	DOWN
Vitvi017857	2.943832961	1.42E-08	0.000000086	UP	Vitvi007210	-1.211168005	4.62E-24	9.42E-23	DOWN
Vitvi001892	2.941986843	3.69E-62	4.62E-60	UP	Vitvi009746	-1.211980548	1.43E-27	3.56E-26	DOWN
Vitvi004706	2.941721243	9.54E-28	2.41E-26	UP	Vitvi001903	-1.212071955	4.56E-19	6.72E-18	DOWN

ID	log2FoldChange	pvalue	padj	change	ID	log2FoldChange	pvalue	padj	change
Vitvi018382	2.941103213	0.000281417	0.000965564	UP	Vitvi000175	-1.212245111	6.95E-09	4.35E-08	DOWN
Vitvi028931	2.937495262	1.67E-26	3.93E-25	UP	Vitvi008226	-1.212301104	2.69E-17	3.48E-16	DOWN
Vitvi020984	2.93636773	0.005341531	0.014292562	UP	Vitvi021358	-1.213888405	1.56E-09	1.05E-08	DOWN
Vitvi018078	2.934213103	2.01E-30	5.87E-29	UP	Vitvi013571	-1.215458825	4.75E-20	7.56E-19	DOWN
Vitvi007393	2.933844755	1.08E-08	6.61E-08	UP	Vitvi031614	-1.216209896	2.49E-46	1.57E-44	DOWN
Vitvi023222	2.932311873	2.9E-26	6.73E-25	UP	Vitvi009556	-1.216425838	0.00000259	0.000012	DOWN
Vitvi023839	2.926205669	1.55E-46	9.8E-45	UP	Vitvi016393	-1.216743356	6.63E-14	6.67E-13	DOWN
Vitvi010182	2.922493931	0.00140706	0.004277834	UP	Vitvi005298	-1.218363067	0.012326852	0.030334749	DOWN
Vitvi002509	2.922347026	2.21E-10	1.6E-09	UP	Vitvi014419	-1.218519978	0.021393552	0.049107353	DOWN
Vitvi001923	2.919500568	5.14E-78	1.11E-75	UP	Vitvi002111	-1.219072535	0.002053855	0.006017295	DOWN
Vitvi018892	2.917720504	0.001341377	0.004092841	UP	Vitvi007758	-1.219327853	1.94E-08	0.000000116	DOWN
Vitvi021286	2.912020152	7.82E-15	8.38E-14	UP	Vitvi021532	-1.220397297	7.25E-15	7.78E-14	DOWN
Vitvi028137	2.911806051	0.005151501	0.013833593	UP	Vitvi032178	-1.220664974	0.001078981	0.003350598	DOWN
Vitvi009163	2.910536548	1.33E-11	1.09E-10	UP	Vitvi002339	-1.222103201	1.17E-16	1.44E-15	DOWN
Vitvi029294	2.906195601	2.37E-115	1.3E-112	UP	Vitvi025401	-1.222466037	4.85E-39	2.14E-37	DOWN
Vitvi014586	2.905746037	3.51E-10	2.51E-09	UP	Vitvi018285	-1.222631217	7.52E-09	4.69E-08	DOWN
Vitvi013072	2.903344619	0.00685826	0.017912432	UP	Vitvi030639	-1.22263548	2.86E-32	9.3E-31	DOWN
Vitvi021237	2.900867569	5.98E-14	6.05E-13	UP	Vitvi019863	-1.222677936	0.000000225	0.00000119	DOWN
Vitvi023166	2.891533268	5.64E-58	6.12E-56	UP	Vitvi015185	-1.223121202	0.002886875	0.008186263	DOWN
Vitvi015970	2.885399277	3.91E-190	1.59E-186	UP	Vitvi007708	-1.223253789	1.18E-36	4.71E-35	DOWN
Vitvi012181	2.877136926	0.00000226	0.0000106	UP	Vitvi025485	-1.223901865	0.000147475	0.000528309	DOWN
Vitvi014901	2.876432138	3.49E-50	2.63E-48	UP	Vitvi029836	-1.223954499	2.07E-20	3.38E-19	DOWN
Vitvi009203	2.868246373	0.000000324	0.00000169	UP	Vitvi009794	-1.224688478	2.73E-29	7.46E-28	DOWN
Vitvi009840	2.864255779	1.28E-23	2.53E-22	UP	Vitvi002355	-1.225209678	5E-24	1.02E-22	DOWN
Vitvi034054	2.863051906	5.73E-75	1.13E-72	UP	Vitvi007814	-1.225344023	0.00000453	0.0000203	DOWN
Vitvi021595	2.857598632	1.14E-100	4.81E-98	UP	Vitvi027691	-1.226071061	0.0000159	0.000066	DOWN
Vitvi027660	2.856075266	0.0000029	0.0000134	UP	Vitvi025109	-1.226770038	1.64E-16	2E-15	DOWN
Vitvi005592	2.856068695	0.0000261	0.000105151	UP	Vitvi004019	-1.227874187	4.15E-08	0.00000024	DOWN
Vitvi021352	2.854776927	1.71E-31	5.33E-30	UP	Vitvi002255	-1.228047426	0.015378715	0.036788655	DOWN
Vitvi027399	2.851094715	1.41E-67	2.22E-65	UP	Vitvi009378	-1.228452531	1.06E-11	8.74E-11	DOWN
Vitvi031657	2.850235439	0.000665605	0.002145653	UP	Vitvi001379	-1.228590305	1.59E-08	9.58E-08	DOWN
Vitvi011122	2.847076151	4.18E-09	2.69E-08	UP	Vitvi003222	-1.228592201	4.86E-09	3.11E-08	DOWN
Vitvi033550	2.842065705	0.000000362	0.00000188	UP	Vitvi000819	-1.229370033	0.0000026	0.0000121	DOWN
Vitvi000848	2.839182585	5.82E-24	1.18E-22	UP	Vitvi010989	-1.230037183	0.000574069	0.001870167	DOWN
Vitvi031059	2.833865672	2.33E-50	1.78E-48	UP	Vitvi002524	-1.230582906	5.51E-08	0.000000313	DOWN
Vitvi002696	2.833566136	2.67E-18	3.71E-17	UP	Vitvi010571	-1.231256246	9.57E-16	1.09E-14	DOWN
Vitvi014925	2.830816792	4.74E-32	1.53E-30	UP	Vitvi001115	-1.231473725	1.85E-10	1.35E-09	DOWN
Vitvi002424	2.826616965	1.02E-13	1.01E-12	UP	Vitvi036020	-1.231588769	8.08E-21	1.35E-19	DOWN
Vitvi025516	2.826580469	1.38E-45	8.3E-44	UP	Vitvi000954	-1.231834306	0.009430347	0.023916746	DOWN
Vitvi035819	2.823398891	5.94E-15	6.42E-14	UP	Vitvi005316	-1.23193857	6.55E-44	3.59E-42	DOWN
Vitvi001417	2.822323639	0.0000191	0.0000784	UP	Vitvi008467	-1.232277107	0.004504187	0.012267237	DOWN
Vitvi032736	2.815701998	4.29E-22	7.76E-21	UP	Vitvi014679	-1.232776669	0.000490396	0.001619397	DOWN
Vitvi014269	2.814421684	0.000317852	0.001081081	UP	Vitvi002880	-1.232922934	8.41E-12	7.01E-11	DOWN
Vitvi030981	2.811663947	0.007067331	0.018396978	UP	Vitvi006639	-1.235205746	1.51E-15	1.7E-14	DOWN
Vitvi017842	2.802470599	6.36E-49	4.57E-47	UP	Vitvi031568	-1.235591451	3.98E-12	3.42E-11	DOWN
Vitvi002838	2.801214052	4.4E-114	2.35E-111	UP	Vitvi012411	-1.23597359	9.28E-10	6.39E-09	DOWN
Vitvi008092	2.800089846	0.000000611	0.00000307	UP	Vitvi023737	-1.237816747	0.000584826	0.00190277	DOWN
Vitvi006022	2.799217104	2.68E-35	1.01E-33	UP	Vitvi011395	-1.237820823	0.006585095	0.017261124	DOWN
Vitvi034060	2.796775817	4.15E-23	7.97E-22	UP	Vitvi012868	-1.237961908	1.25E-23	2.47E-22	DOWN
Vitvi019675	2.794657341	2.99E-12	2.61E-11	UP	Vitvi013475	-1.238370626	0.005008817	0.013489671	DOWN
Vitvi019732	2.79253649	1.91E-27	4.72E-26	UP	Vitvi017878	-1.238481378	1.36E-09	9.23E-09	DOWN
Vitvi019630	2.787820109	8.13E-08	0.000000454	UP	Vitvi010274	-1.238654919	2.02E-12	1.79E-11	DOWN
Vitvi012856	2.7871714	0.002286423	0.006626973	UP	Vitvi009078	-1.238747747	1.05E-08	6.42E-08	DOWN
Vitvi019676	2.786412051	2.94E-26	6.83E-25	UP	Vitvi010958	-1.239092512	1.04E-29	2.92E-28	DOWN
Vitvi019701	2.77573079	6.5E-20	1.03E-18	UP	Vitvi022923	-1.239809239	8.09E-08	0.000000451	DOWN
Vitvi015480	2.771085734	9.56E-12	7.92E-11	UP	Vitvi028606	-1.240027202	2.66E-46	1.66E-44	DOWN
Vitvi009413	2.77096732	6.18E-18	8.34E-17	UP	Vitvi014620	-1.241291743	3.19E-19	4.75E-18	DOWN

ID	log2FoldChange	pvalue	padj	change	ID	log2FoldChange	pvalue	padj	change
Vitvi007851	2.769922777	3.31E-20	5.34E-19	UP	Vitvi014413	-1.241383077	0.011507369	0.028535914	DOWN
Vitvi026282	2.755015765	0.002894518	0.008205646	UP	Vitvi023502	-1.241750328	3.19E-16	3.81E-15	DOWN
Vitvi030562	2.754071046	1.63E-24	3.43E-23	UP	Vitvi031869	-1.242570065	1.51E-55	1.45E-53	DOWN
Vitvi012980	2.753718947	0.003608468	0.01003769	UP	Vitvi000718	-1.244229006	5.95E-09	3.77E-08	DOWN
Vitvi011794	2.751657514	2.68E-92	9.09E-90	UP	Vitvi028524	-1.244424281	0.00000111	0.00000539	DOWN
Vitvi025189	2.751147701	2.51E-15	2.78E-14	UP	Vitvi028898	-1.244917682	4.37E-16	5.13E-15	DOWN
Vitvi021953	2.750766967	8.89E-75	1.74E-72	UP	Vitvi036064	-1.245906576	1.63E-40	7.62E-39	DOWN
Vitvi015832	2.749859415	0.0000213	0.0000869	UP	Vitvi033722	-1.246297483	0.000391132	0.001310568	DOWN
Vitvi013579	2.749295473	1.18E-75	2.36E-73	UP	Vitvi013444	-1.246984854	0.000000018	0.000000108	DOWN
Vitvi008063	2.745730882	2.81E-76	5.82E-74	UP	Vitvi007520	-1.247291466	2.51E-08	0.000000148	DOWN
Vitvi032945	2.734963532	1.89E-61	2.33E-59	UP	Vitvi004066	-1.248887591	8.81E-25	1.89E-23	DOWN
Vitvi025190	2.731098274	5.46E-23	1.05E-21	UP	Vitvi033922	-1.249056164	0.000251813	0.000871053	DOWN
Vitvi011537	2.727832432	6.21E-83	1.64E-80	UP	Vitvi031764	-1.249270241	2.35E-35	8.82E-34	DOWN
Vitvi025999	2.727195761	9.79E-122	6.41E-119	UP	Vitvi018230	-1.249337206	0.000349533	0.001180728	DOWN
Vitvi018215	2.725948414	7.39E-27	1.77E-25	UP	Vitvi017153	-1.249693496	6.95E-23	1.32E-21	DOWN
Vitvi030716	2.723716897	5.07E-133	4.68E-130	UP	Vitvi004003	-1.249781857	2.71E-33	9.2E-32	DOWN
Vitvi019742	2.722543942	3.42E-20	5.49E-19	UP	Vitvi013824	-1.250378578	2.26E-08	0.000000134	DOWN
Vitvi010792	2.720463891	0.000000105	0.000000578	UP	Vitvi003574	-1.25089295	1.71E-28	4.47E-27	DOWN
Vitvi030654	2.7166723	0.0000412	0.000160968	UP	Vitvi033625	-1.25105386	1.18E-28	3.11E-27	DOWN
Vitvi013073	2.709905857	0.000682453	0.002195437	UP	Vitvi001102	-1.25124017	9.79E-26	2.21E-24	DOWN
Vitvi022824	2.707975944	0.000523981	0.00171968	UP	Vitvi012598	-1.251451171	3.17E-23	6.11E-22	DOWN
Vitvi007136	2.705833437	2.35E-25	5.2E-24	UP	Vitvi017038	-1.253107188	2.08E-09	1.38E-08	DOWN
Vitvi022320	2.697449492	0.019010593	0.044338405	UP	Vitvi001908	-1.253174527	6.26E-09	3.95E-08	DOWN
Vitvi004207	2.694703033	0.000000165	0.000000892	UP	Vitvi010321	-1.253361696	1.13E-37	4.71E-36	DOWN
Vitvi022322	2.690342118	1.02E-08	6.25E-08	UP	Vitvi023171	-1.256094814	2.64E-24	5.49E-23	DOWN
Vitvi018493	2.687652291	4.74E-44	2.63E-42	UP	Vitvi036399	-1.257040539	0.007262231	0.018851174	DOWN
Vitvi009393	2.686320616	0.0000237	0.0000961	UP	Vitvi014844	-1.257229286	2.69E-16	3.23E-15	DOWN
Vitvi009284	2.686145706	4.94E-38	2.09E-36	UP	Vitvi003552	-1.25758206	6.54E-17	8.22E-16	DOWN
Vitvi033132	2.68542967	1.61E-16	1.97E-15	UP	Vitvi025038	-1.258095995	3.17E-11	2.49E-10	DOWN
Vitvi010006	2.682927405	0.000102057	0.000375744	UP	Vitvi015093	-1.258890092	2.49E-10	1.79E-09	DOWN
Vitvi030675	2.681097925	0.004675029	0.012681515	UP	Vitvi000834	-1.259978315	7.47E-27	1.79E-25	DOWN
Vitvi002819	2.675253773	4.93E-15	5.37E-14	UP	Vitvi016133	-1.260574389	4.89E-11	3.77E-10	DOWN
Vitvi011876	2.673698383	0.013242387	0.032274943	UP	Vitvi020458	-1.260795923	6.61E-27	1.59E-25	DOWN
Vitvi020894	2.672785747	0.000139095	0.00050023	UP	Vitvi001219	-1.261162555	2.72E-21	4.69E-20	DOWN
Vitvi033521	2.67193369	1.22E-24	2.59E-23	UP	Vitvi035939	-1.261571085	0.0000371	0.000145737	DOWN
Vitvi000987	2.666149568	9.76E-12	8.07E-11	UP	Vitvi029059	-1.261607729	4.67E-29	1.25E-27	DOWN
Vitvi005440	2.665637184	2.74E-119	1.59E-116	UP	Vitvi021198	-1.261879077	2.3E-39	1.03E-37	DOWN
Vitvi031607	2.660472612	0.006017737	0.015915661	UP	Vitvi028836	-1.26319578	2.9E-23	5.62E-22	DOWN
Vitvi026679	2.660217732	2.73E-10	1.96E-09	UP	Vitvi030706	-1.263372517	8.09E-18	1.08E-16	DOWN
Vitvi013861	2.659905407	1.22E-08	7.41E-08	UP	Vitvi015804	-1.263659594	4.99E-34	1.76E-32	DOWN
Vitvi000134	2.656871035	6.86E-17	8.61E-16	UP	Vitvi006129	-1.263843936	0.010097102	0.025423619	DOWN
Vitvi031533	2.656359379	0.000000924	0.00000454	UP	Vitvi019893	-1.264296818	1.38E-21	2.42E-20	DOWN
Vitvi017834	2.655038284	4.59E-123	3.21E-120	UP	Vitvi020464	-1.264753075	0.012536833	0.030769473	DOWN
Vitvi001644	2.643513582	8.11E-22	1.45E-20	UP	Vitvi011387	-1.264923198	1.99E-25	4.41E-24	DOWN
Vitvi020987	2.635673771	8.47E-13	7.77E-12	UP	Vitvi031510	-1.264947251	1.3E-36	5.15E-35	DOWN
Vitvi002718	2.635372625	0.00000854	0.0000369	UP	Vitvi007777	-1.264989087	7.32E-50	5.48E-48	DOWN
Vitvi009368	2.627987554	0.00000146	0.000007	UP	Vitvi012400	-1.265204062	4.79E-22	8.65E-21	DOWN
Vitvi023383	2.624962275	0.0000728	0.000273206	UP	Vitvi008159	-1.265595701	4.47E-09	2.87E-08	DOWN
Vitvi003852	2.624463311	1.62E-24	3.4E-23	UP	Vitvi010034	-1.265781159	9.24E-10	6.37E-09	DOWN
Vitvi010136	2.62131962	0.00012245	0.00044438	UP	Vitvi012730	-1.266314518	8.87E-20	1.38E-18	DOWN
Vitvi032930	2.61240756	8.26E-23	1.56E-21	UP	Vitvi003956	-1.26663702	4.89E-36	1.89E-34	DOWN
Vitvi019699	2.611423093	6.21E-26	1.41E-24	UP	Vitvi021564	-1.267073138	1.97E-27	4.85E-26	DOWN
Vitvi013379	2.610081422	1.35E-100	5.6E-98	UP	Vitvi020402	-1.267280815	1.75E-18	2.47E-17	DOWN
Vitvi002269	2.605256591	2.89E-24	5.98E-23	UP	Vitvi018226	-1.267387296	2.99E-13	7.08E-11	DOWN
Vitvi037077	2.598027849	0.00000182	0.00000861	UP	Vitvi034323	-1.267458922	2E-32	6.56E-31	DOWN
Vitvi027488	2.596812344	0.016319596	0.038801782	UP	Vitvi027072	-1.268633454	0.012012343	0.029632587	DOWN
Vitvi027659	2.593615846	1.09E-35	4.12E-34	UP	Vitvi000604	-1.268721395	1.38E-17	1.82E-16	DOWN

ID	log2FoldChange	pvalue	padj	change	ID	log2FoldChange	pvalue	padj	change
Vitvi009751	2.585769478	5.8E-16	6.75E-15	UP	Vitvi023668	-1.269859149	0.006054896	0.015999355	DOWN
Vitvi030679	2.583062847	8.04E-40	3.65E-38	UP	Vitvi009227	-1.270113839	0.000443648	0.00147365	DOWN
Vitvi032944	2.581419723	8.54E-23	1.61E-21	UP	Vitvi025369	-1.270243231	1.01E-12	9.22E-12	DOWN
Vitvi021332	2.580408787	8.64E-65	1.15E-62	UP	Vitvi002056	-1.270354758	0.000000577	0.00000291	DOWN
Vitvi015371	2.579109045	9.25E-08	0.000000513	UP	Vitvi002303	-1.270818874	0.000000713	0.00000355	DOWN
Vitvi005122	2.57431734	0.000000905	0.00000445	UP	Vitvi002003	-1.270969153	7.29E-21	1.22E-19	DOWN
Vitvi010956	2.573014942	1.74E-81	4.3E-79	UP	Vitvi011576	-1.271518806	3.04E-18	4.2E-17	DOWN
Vitvi021766	2.572140539	7.23E-19	1.04E-17	UP	Vitvi029815	-1.273339214	0.00000359	0.0000163	DOWN
Vitvi033054	2.568211064	3.03E-58	3.37E-56	UP	Vitvi025171	-1.273696825	3.12E-08	0.000000183	DOWN
Vitvi021641	2.568112635	4.24E-149	5.74E-146	UP	Vitvi010066	-1.27389869	6.8E-11	1.08E-08	DOWN
Vitvi004232	2.565663111	1.04E-65	1.42E-63	UP	Vitvi012897	-1.274235447	4.64E-28	1.19E-26	DOWN
Vitvi036630	2.564381516	0.020672729	0.047651439	UP	Vitvi004254	-1.276678906	0.0000012	0.0000058	DOWN
Vitvi009554	2.564201096	3.55E-38	1.51E-36	UP	Vitvi028636	-1.27671353	1E-25	2.26E-24	DOWN
Vitvi001073	2.56190948	7.25E-19	1.05E-17	UP	Vitvi026786	-1.276865328	1.4E-51	1.13E-49	DOWN
Vitvi011236	2.561406426	1.36E-60	1.65E-58	UP	Vitvi023158	-1.276926756	2.4E-12	2.11E-11	DOWN
Vitvi007829	2.560576141	4.47E-85	1.26E-82	UP	Vitvi023983	-1.277080314	0.012548916	0.030791687	DOWN
Vitvi003481	2.559569101	1.23E-19	1.9E-18	UP	Vitvi014897	-1.277288237	0.00000687	0.0000301	DOWN
Vitvi033388	2.558948493	1.87E-08	0.000000112	UP	Vitvi000319	-1.278607337	3.7E-44	2.07E-42	DOWN
Vitvi009351	2.556256456	0.000000123	0.000000672	UP	Vitvi030281	-1.278752339	0.016123646	0.038394314	DOWN
Vitvi010939	2.554603684	3.33E-26	7.69E-25	UP	Vitvi027384	-1.278872608	8.25E-19	1.18E-17	DOWN
Vitvi035306	2.551367022	2.32E-19	3.48E-18	UP	Vitvi035628	-1.280747417	2.32E-09	1.53E-08	DOWN
Vitvi007900	2.54267808	0.0000555	0.000212166	UP	Vitvi031696	-1.281089287	3.1E-24	6.42E-23	DOWN
Vitvi013413	2.541191245	1.68E-40	7.85E-39	UP	Vitvi009944	-1.281143746	5.63E-11	4.32E-10	DOWN
Vitvi032540	2.536635668	9.97E-09	6.14E-08	UP	Vitvi009044	-1.282417818	2.55E-48	1.77E-46	DOWN
Vitvi013213	2.533624454	1.41E-44	8.19E-43	UP	Vitvi033901	-1.282756782	0.000394005	0.00131976	DOWN
Vitvi007734	2.529936676	8.32E-23	1.57E-21	UP	Vitvi004749	-1.283627316	0.001624391	0.004875093	DOWN
Vitvi008051	2.526719159	5.35E-21	9.09E-20	UP	Vitvi004073	-1.283938081	0.00000103	0.00000503	DOWN
Vitvi014216	2.525421224	9.35E-42	4.64E-40	UP	Vitvi018144	-1.284520932	6.98E-19	1.01E-17	DOWN
Vitvi010253	2.522629241	0.000319749	0.001087168	UP	Vitvi023342	-1.286456807	0.002306645	0.006680817	DOWN
Vitvi003011	2.522579951	8.39E-34	2.93E-32	UP	Vitvi017011	-1.287505856	1.54E-20	2.54E-19	DOWN
Vitvi002423	2.521670698	1.82E-22	3.36E-21	UP	Vitvi009718	-1.288098674	4.16E-24	8.55E-23	DOWN
Vitvi004267	2.519624216	6.52E-51	5.17E-49	UP	Vitvi008757	-1.288537407	6.93E-09	4.34E-08	DOWN
Vitvi030935	2.516628819	0.003112699	0.008763014	UP	Vitvi016157	-1.290019659	5.44E-26	1.25E-24	DOWN
Vitvi018098	2.511421416	0.003242853	0.00909916	UP	Vitvi023221	-1.291243231	3.72E-11	2.91E-10	DOWN
Vitvi031468	2.509971347	9.78E-14	9.68E-13	UP	Vitvi016433	-1.291347697	8.19E-08	0.00000685	DOWN
Vitvi023603	2.506236729	4.25E-45	2.52E-43	UP	Vitvi007591	-1.291697074	0.020167335	0.046666541	DOWN
Vitvi000740	2.505546125	5.78E-19	8.45E-18	UP	Vitvi013473	-1.292583626	1.97E-18	2.76E-17	DOWN
Vitvi004238	2.504597882	2.78E-23	5.39E-22	UP	Vitvi005530	-1.293031962	3.27E-08	0.000000191	DOWN
Vitvi026828	2.504021977	0.00000182	0.00000859	UP	Vitvi003893	-1.295090498	1.82E-14	1.9E-13	DOWN
Vitvi022972	2.49608399	1.08E-51	8.81E-50	UP	Vitvi033010	-1.295221391	3.7E-31	1.13E-29	DOWN
Vitvi021611	2.494555551	3.71E-77	7.77E-75	UP	Vitvi006750	-1.296197457	0.0000651	0.000246139	DOWN
Vitvi018342	2.494314868	2.62E-69	4.25E-67	UP	Vitvi005758	-1.29662657	3.79E-20	6.08E-19	DOWN
Vitvi034677	2.492122256	9.17E-15	9.77E-14	UP	Vitvi025104	-1.29694766	0.004130944	0.011345015	DOWN
Vitvi001812	2.490708987	0.014664267	0.035274822	UP	Vitvi011014	-1.297463346	0.000012	0.0000507	DOWN
Vitvi023367	2.48914485	3.16E-18	4.35E-17	UP	Vitvi026768	-1.297921288	0.000000134	0.000000731	DOWN
Vitvi010252	2.485919922	1.09E-23	2.16E-22	UP	Vitvi027267	-1.298028599	0.002254493	0.006544698	DOWN
Vitvi006860	2.484898346	1.47E-79	3.32E-77	UP	Vitvi032077	-1.298453528	8.97E-11	6.74E-10	DOWN
Vitvi010220	2.484141093	6.56E-109	3.25E-106	UP	Vitvi031285	-1.2988196	1.78E-11	1.43E-10	DOWN
Vitvi031318	2.482174341	3.39E-66	4.85E-64	UP	Vitvi031051	-1.298880902	6.56E-14	6.61E-13	DOWN
Vitvi023123	2.47134442	0.00000331	0.0000151	UP	Vitvi036208	-1.299293647	9.14E-23	1.72E-21	DOWN
Vitvi020424	2.467701159	0.00464401	0.012615906	UP	Vitvi006856	-1.299378955	6.62E-12	5.56E-11	DOWN
Vitvi011491	2.465279763	1.02E-09	0.000000007	UP	Vitvi009187	-1.299804589	3.18E-10	2.28E-09	DOWN
Vitvi009134	2.460835517	0.004101037	0.011272024	UP	Vitvi011423	-1.300430813	0.000000428	0.0000022	DOWN
Vitvi025969	2.451568094	4.63E-08	0.000000265	UP	Vitvi006558	-1.300805332	5.58E-15	6.05E-14	DOWN
Vitvi030405	2.451509361	0.0000469	0.001565936	UP	Vitvi002912	-1.30112322	1.02E-12	9.25E-12	DOWN
Vitvi005784	2.448954237	1.06E-57	1.14E-55	UP	Vitvi013764	-1.301145727	0.000000027	0.000000159	DOWN
Vitvi030742	2.447031674	3.57E-22	6.5E-21	UP	Vitvi024533	-1.301466741	0.000195986	0.000688612	DOWN

ID	log2FoldChange	pvalue	padj	change	ID	log2FoldChange	pvalue	padj	change
Vitvi011727	2.445710213	0.0000206	0.0000842	UP	Vitvi025099	-1.302407499	0.003781374	0.010477051	DOWN
Vitvi030729	2.441912888	0.010406869	0.026132318	UP	Vitvi003559	-1.30286478	4E-10	2.84E-09	DOWN
Vitvi009514	2.439280978	0.00000125	0.00000601	UP	Vitvi035644	-1.303051408	0.0000383	0.000150174	DOWN
Vitvi000438	2.438701961	1.5E-37	6.21E-36	UP	Vitvi033795	-1.304572521	2.96E-10	2.12E-09	DOWN
Vitvi023528	2.438121501	0.0000701	0.002152716	UP	Vitvi034653	-1.304863485	0.0000016	0.00000765	DOWN
Vitvi015214	2.436535296	0.001886685	0.005570917	UP	Vitvi005741	-1.305792616	0.000000304	0.00000159	DOWN
Vitvi020177	2.431464937	1.8E-41	8.82E-40	UP	Vitvi011283	-1.306117403	3.51E-09	2.28E-08	DOWN
Vitvi014186	2.423114898	0.000000555	0.00000281	UP	Vitvi016098	-1.307569053	0.00000661	0.000029	DOWN
Vitvi011647	2.421622589	2.07E-18	2.9E-17	UP	Vitvi017807	-1.308014734	0.001145858	0.003537183	DOWN
Vitvi023587	2.421239952	0.000591209	0.001922306	UP	Vitvi006630	-1.308060399	0.007649488	0.019774035	DOWN
Vitvi033436	2.414356801	1.42E-46	9.07E-45	UP	Vitvi021070	-1.30858753	0.000101536	0.000373895	DOWN
Vitvi007791	2.414232772	8.47E-30	2.39E-28	UP	Vitvi014577	-1.308763762	0.007150831	0.018590513	DOWN
Vitvi027051	2.41191183	2.24E-15	2.5E-14	UP	Vitvi013011	-1.309606434	1.84E-16	2.24E-15	DOWN
Vitvi005969	2.411717218	5.01E-09	0.000000032	UP	Vitvi025385	-1.310967202	6.9E-35	2.51E-33	DOWN
Vitvi029758	2.408283825	1.4E-28	3.68E-27	UP	Vitvi015442	-1.311005973	1.55E-17	2.04E-16	DOWN
Vitvi016147	2.407045073	0.00034423	0.001164361	UP	Vitvi035685	-1.311487521	1.48E-13	1.44E-12	DOWN
Vitvi015164	2.406476652	0.011759243	0.029075318	UP	Vitvi002117	-1.311826986	7.6E-39	3.34E-37	DOWN
Vitvi001066	2.40644576	0.00000012	0.00000066	UP	Vitvi015076	-1.312517933	0.0000186	0.0000766	DOWN
Vitvi000878	2.403029624	3.06E-18	4.23E-17	UP	Vitvi029573	-1.312832415	2.04E-36	8.01E-35	DOWN
Vitvi000765	2.401099391	1.13E-43	6.12E-42	UP	Vitvi029942	-1.313319989	1.35E-15	1.52E-14	DOWN
Vitvi012830	2.398082889	0.016628665	0.039439683	UP	Vitvi034022	-1.314545054	7.01E-18	9.43E-17	DOWN
Vitvi015831	2.397726913	1.22E-83	3.34E-81	UP	Vitvi017036	-1.314763862	0.000830808	0.002634717	DOWN
Vitvi004322	2.396257134	1.65E-106	7.62E-104	UP	Vitvi033229	-1.3148532	6.36E-11	4.85E-10	DOWN
Vitvi018610	2.395821072	0.00056969	0.00185739	UP	Vitvi018189	-1.315589653	2.77E-34	9.8E-33	DOWN
Vitvi004191	2.395795639	8.28E-63	1.04E-60	UP	Vitvi014886	-1.315818097	0.0000202	0.0000827	DOWN
Vitvi023375	2.395338063	1.4E-30	4.13E-29	UP	Vitvi007143	-1.316494875	0.000995203	0.003108497	DOWN
Vitvi013057	2.394620045	9.43E-131	8.32E-128	UP	Vitvi004487	-1.317026993	1.82E-16	2.22E-15	DOWN
Vitvi026635	2.393969725	0.006225318	0.016389976	UP	Vitvi032929	-1.317396157	1.14E-41	5.63E-40	DOWN
Vitvi008127	2.391927145	1.17E-80	2.73E-78	UP	Vitvi031208	-1.317756183	0.009638309	0.02436507	DOWN
Vitvi011111	2.390472253	0.000000568	0.00000287	UP	Vitvi014930	-1.317962717	2.7E-35	1.01E-33	DOWN
Vitvi036629	2.388990063	0.000142505	0.000511408	UP	Vitvi006812	-1.318559946	1.06E-21	1.88E-20	DOWN
Vitvi033687	2.385696252	2.54E-17	3.3E-16	UP	Vitvi003536	-1.319135457	3.61E-16	4.29E-15	DOWN
Vitvi018374	2.38477638	5.6E-11	4.3E-10	UP	Vitvi009939	-1.319158707	0.0000354	0.000139606	DOWN
Vitvi012555	2.379261175	0.000257733	0.000890317	UP	Vitvi014927	-1.319319677	3.99E-19	5.91E-18	DOWN
Vitvi022862	2.375366628	4.15E-80	9.58E-78	UP	Vitvi007812	-1.319330223	1.67E-12	1.49E-11	DOWN
Vitvi002674	2.373115127	4.17E-86	1.19E-83	UP	Vitvi015876	-1.320185096	0.0000115	0.0000488	DOWN
Vitvi015481	2.366351413	1.48E-08	8.94E-08	UP	Vitvi003860	-1.320306312	0.002227873	0.006479461	DOWN
Vitvi027649	2.364837287	5.22E-36	2.01E-34	UP	Vitvi010677	-1.320358068	2.93E-15	3.24E-14	DOWN
Vitvi033407	2.363537388	1.7E-66	2.5E-64	UP	Vitvi033483	-1.320371749	1.74E-10	1.27E-09	DOWN
Vitvi018613	2.362928761	2.84E-10	2.04E-09	UP	Vitvi002252	-1.320506196	0.000000052	0.000000297	DOWN
Vitvi020506	2.362576466	1.79E-86	5.33E-84	UP	Vitvi006153	-1.32071873	5.6E-11	4.3E-10	DOWN
Vitvi027151	2.361665209	0.000129131	0.000466815	UP	Vitvi030766	-1.32252493	0.000179905	0.000636624	DOWN
Vitvi013223	2.361513202	6.24E-43	3.28E-41	UP	Vitvi018671	-1.323383187	0.000117021	0.000425975	DOWN
Vitvi008769	2.358957872	4.35E-27	1.05E-25	UP	Vitvi018026	-1.323790984	0.0000817	0.000304778	DOWN
Vitvi002018	2.355096113	2.41E-49	1.78E-47	UP	Vitvi027255	-1.323815031	2.2E-10	1.59E-09	DOWN
Vitvi018383	2.349149457	1.02E-116	5.77E-114	UP	Vitvi029955	-1.323947404	0.001184753	0.003643958	DOWN
Vitvi007317	2.346019626	0.000000692	0.00000346	UP	Vitvi017891	-1.324540397	2.79E-11	2.21E-10	DOWN
Vitvi020554	2.343403238	4.88E-32	1.58E-30	UP	Vitvi015310	-1.324605217	0.0000235	0.0000953	DOWN
Vitvi037261	2.340446085	8.47E-34	2.96E-32	UP	Vitvi003804	-1.325288148	0.005227163	0.014009338	DOWN
Vitvi016410	2.337460293	0.000184959	0.000653369	UP	Vitvi000399	-1.325770271	3.67E-13	3.45E-12	DOWN
Vitvi023327	2.33560972	0.001997533	0.00586425	UP	Vitvi035845	-1.3259526	6.52E-08	0.000000367	DOWN
Vitvi000591	2.33515643	2.76E-43	1.47E-41	UP	Vitvi033126	-1.32605118	2.69E-12	2.35E-11	DOWN
Vitvi036607	2.333843607	4.43E-46	2.72E-44	UP	Vitvi017822	-1.326194138	9.29E-09	5.74E-08	DOWN
Vitvi015077	2.330460512	0.002266792	0.006574764	UP	Vitvi002074	-1.326950189	4.96E-09	3.17E-08	DOWN
Vitvi014357	2.32591864	3.81E-44	2.13E-42	UP	Vitvi023098	-1.327479546	0.012835659	0.031388322	DOWN
Vitvi017508	2.325351139	1.49E-39	6.69E-38	UP	Vitvi027567	-1.328031833	1.35E-48	9.56E-47	DOWN
Vitvi017998	2.32365585	0.003786238	0.010487666	UP	Vitvi001025	-1.328353669	0.001338295	0.004084052	DOWN

ID	log2FoldChange	pvalue	padj	change	ID	log2FoldChange	pvalue	padj	change
Vitvi019312	2.323506963	1.65E-23	3.23E-22	UP	Vitvi009961	-1.32854256	1.5E-28	3.92E-27	DOWN
Vitvi013266	2.319151486	0.0000344	0.000135791	UP	Vitvi012230	-1.328715391	1.52E-08	9.18E-08	DOWN
Vitvi014546	2.318898483	0.020044047	0.046460652	UP	Vitvi014929	-1.329682628	0.0000218	0.0000887	DOWN
Vitvi011447	2.316813539	1.05E-13	1.03E-12	UP	Vitvi020072	-1.32977156	6.03E-12	5.1E-11	DOWN
Vitvi016123	2.316302318	2.88E-24	5.98E-23	UP	Vitvi007926	-1.32987757	3.64E-30	1.05E-28	DOWN
Vitvi012045	2.310342996	4.38E-09	2.82E-08	UP	Vitvi017609	-1.330981744	9.82E-15	1.04E-13	DOWN
Vitvi022018	2.310115708	0.0000159	0.0000662	UP	Vitvi021740	-1.331131683	0.0000102	0.0000436	DOWN
Vitvi036060	2.308110238	1.11E-12	1E-11	UP	Vitvi005819	-1.331232039	0.0000102	0.0000436	DOWN
Vitvi028626	2.30067358	1.72E-50	1.32E-48	UP	Vitvi009728	-1.331593059	2.85E-16	3.43E-15	DOWN
Vitvi023136	2.298663212	0.00360107	0.010021226	UP	Vitvi018897	-1.332471824	0.0000767	0.000287488	DOWN
Vitvi028700	2.296758795	7.43E-56	7.22E-54	UP	Vitvi032994	-1.332495832	1.78E-08	0.000000106	DOWN
Vitvi021715	2.295041306	1.34E-32	4.45E-31	UP	Vitvi000126	-1.332551107	3.55E-08	0.000000206	DOWN
Vitvi000111	2.292048545	1.75E-93	6.14E-91	UP	Vitvi025891	-1.333562228	2.95E-11	2.33E-10	DOWN
Vitvi017715	2.290665208	8E-70	1.35E-67	UP	Vitvi028956	-1.333593981	0.0000704	0.000264967	DOWN
Vitvi036395	2.287455592	1.09E-74	2.1E-72	UP	Vitvi014441	-1.333699718	1.21E-41	5.97E-40	DOWN
Vitvi014412	2.28395276	0.004790964	0.012968291	UP	Vitvi023476	-1.334094889	0.018091484	0.042438414	DOWN
Vitvi000225	2.282407921	0.000188348	0.000664188	UP	Vitvi000665	-1.334586597	1.49E-08	8.98E-08	DOWN
Vitvi028227	2.281951084	0.00000221	0.0000104	UP	Vitvi027468	-1.334849485	5.67E-08	0.000000321	DOWN
Vitvi031738	2.281647513	9.25E-09	5.72E-08	UP	Vitvi021366	-1.335191832	1.85E-11	1.49E-10	DOWN
Vitvi002916	2.281413595	6.07E-60	7.21E-58	UP	Vitvi015476	-1.335438417	0.00000245	0.0000114	DOWN
Vitvi033779	2.280582027	2.39E-11	1.9E-10	UP	Vitvi003385	-1.336032908	7.46E-17	9.35E-16	DOWN
Vitvi029397	2.278487608	0.0000386	0.000151328	UP	Vitvi020143	-1.336454912	1.03E-27	2.58E-26	DOWN
Vitvi002682	2.275151591	1.98E-09	1.32E-08	UP	Vitvi016168	-1.336477215	1.18E-23	2.34E-22	DOWN
Vitvi017850	2.27500403	1.78E-14	1.87E-13	UP	Vitvi020818	-1.336482197	1.77E-41	8.68E-40	DOWN
Vitvi022284	2.274083712	1.18E-24	2.52E-23	UP	Vitvi001494	-1.336739863	0.0056705	0.015071866	DOWN
Vitvi030561	2.273378938	2.32E-33	7.95E-32	UP	Vitvi000811	-1.336782832	8.7E-16	1E-14	DOWN
Vitvi036223	2.272437453	8.67E-15	9.25E-14	UP	Vitvi010731	-1.33694237	0.001576067	0.004741975	DOWN
Vitvi019702	2.271175359	0.0000338	0.000133308	UP	Vitvi036680	-1.338501002	0.0000485	0.000187179	DOWN
Vitvi012757	2.270502546	1.28E-36	5.08E-35	UP	Vitvi025236	-1.33890457	0.003614932	0.010051541	DOWN
Vitvi001643	2.269822506	0.00000205	0.00000965	UP	Vitvi019955	-1.338915415	0.009079181	0.023127204	DOWN
Vitvi005747	2.266144207	3.08E-11	2.42E-10	UP	Vitvi033322	-1.338941094	1.2E-22	2.23E-21	DOWN
Vitvi023961	2.263045923	8.28E-53	7.19E-51	UP	Vitvi014418	-1.338989742	0.00000015	0.000000814	DOWN
Vitvi000849	2.262070471	2.24E-36	8.8E-35	UP	Vitvi018278	-1.33991426	6.64E-09	4.17E-08	DOWN
Vitvi017335	2.260303575	7.74E-23	1.46E-21	UP	Vitvi025040	-1.34140336	6.41E-33	2.15E-31	DOWN
Vitvi035266	2.25920029	0.000644476	0.002083494	UP	Vitvi031848	-1.341507451	1.59E-44	9.2E-43	DOWN
Vitvi011421	2.258525369	9.29E-10	6.39E-09	UP	Vitvi006763	-1.34168189	6.96E-36	2.66E-34	DOWN
Vitvi002049	2.247206089	0.000000136	0.000000744	UP	Vitvi007798	-1.342670456	0.001609574	0.004835626	DOWN
Vitvi014652	2.24617715	7.74E-28	1.96E-26	UP	Vitvi014931	-1.342699733	7.83E-34	2.74E-32	DOWN
Vitvi018224	2.24134994	2.91E-43	1.55E-41	UP	Vitvi010902	-1.34296028	1.16E-42	5.99E-41	DOWN
Vitvi031827	2.23826187	1.79E-12	1.59E-11	UP	Vitvi013169	-1.34348379	1.37E-08	8.28E-08	DOWN
Vitvi001797	2.237648755	0.0000018	0.00000854	UP	Vitvi017722	-1.343802989	1.71E-18	2.42E-17	DOWN
Vitvi005324	2.234595257	2.3E-66	3.35E-64	UP	Vitvi014952	-1.344133887	0.001731478	0.005165948	DOWN
Vitvi013411	2.234179228	4.14E-25	9.07E-24	UP	Vitvi017815	-1.344660806	6.11E-32	1.96E-30	DOWN
Vitvi013885	2.232791588	0.0000411	0.000160388	UP	Vitvi010879	-1.345925515	0.000000142	0.000000774	DOWN
Vitvi004205	2.2309957	0.0000155	0.0000647	UP	Vitvi002231	-1.346043697	0.0000131	0.0000549	DOWN
Vitvi002577	2.230872979	3.93E-42	2E-40	UP	Vitvi002181	-1.34608815	1.85E-15	2.07E-14	DOWN
Vitvi007068	2.228949308	1.13E-23	2.24E-22	UP	Vitvi009421	-1.346332586	7.84E-11	5.91E-10	DOWN
Vitvi032855	2.228643663	0.0000895	0.000331856	UP	Vitvi030105	-1.347214369	0.005684457	0.015106766	DOWN
Vitvi009142	2.22730245	0.00000377	0.0000171	UP	Vitvi035167	-1.347586726	0.00000884	0.0000381	DOWN
Vitvi019505	2.22689974	2.8E-24	5.82E-23	UP	Vitvi033624	-1.348628431	5.21E-12	4.42E-11	DOWN
Vitvi007133	2.225505444	3.83E-82	9.74E-80	UP	Vitvi008229	-1.348662105	1.77E-09	1.18E-08	DOWN
Vitvi003392	2.225194482	6.03E-16	7E-15	UP	Vitvi016453	-1.348694292	0.00006	0.000228249	DOWN
Vitvi026723	2.224775241	0.001024236	0.00319231	UP	Vitvi033160	-1.348739877	2.52E-46	1.58E-44	DOWN
Vitvi002101	2.224632871	3.58E-24	7.38E-23	UP	Vitvi009467	-1.348903819	9.42E-13	8.6E-12	DOWN
Vitvi015588	2.223326673	0.0000889	0.000329916	UP	Vitvi012210	-1.349303019	0.0000015	0.00000717	DOWN
Vitvi033060	2.222738035	2.66E-20	4.31E-19	UP	Vitvi020077	-1.349531353	1.36E-23	2.68E-22	DOWN
Vitvi027762	2.218521373	1.45E-12	1.3E-11	UP	Vitvi012880	-1.349794883	6.45E-12	5.43E-11	DOWN

ID	log2FoldChange	pvalue	padj	change	ID	log2FoldChange	pvalue	padj	change
Vitvi005602	2.215175547	0.00000254	0.0000118	UP	Vitvi005273	-1.349859615	6.55E-16	7.59E-15	DOWN
Vitvi035907	2.214999671	1.4E-12	1.25E-11	UP	Vitvi013907	-1.350149437	4.24E-12	3.63E-11	DOWN
Vitvi030714	2.214132705	0.007806953	0.020141602	UP	Vitvi014466	-1.350562043	2.1E-71	3.8E-69	DOWN
Vitvi012193	2.21397465	1.3E-13	1.28E-12	UP	Vitvi025059	-1.350689077	5.73E-08	0.000000325	DOWN
Vitvi002936	2.21377427	4.62E-19	6.8E-18	UP	Vitvi007562	-1.350789486	1.46E-10	1.08E-09	DOWN
Vitvi031898	2.209860848	8.43E-42	4.2E-40	UP	Vitvi015984	-1.351484921	0.003298208	0.009242992	DOWN
Vitvi010569	2.209279704	3.38E-29	9.18E-28	UP	Vitvi005699	-1.351954423	0.008587896	0.021981629	DOWN
Vitvi004549	2.208039159	4.98E-63	6.36E-61	UP	Vitvi025105	-1.35195549	1.47E-08	0.000000089	DOWN
Vitvi033540	2.205054113	0.000000149	0.000000808	UP	Vitvi021636	-1.352233843	3.71E-35	1.38E-33	DOWN
Vitvi015056	2.203741727	1.59E-11	1.28E-10	UP	Vitvi015678	-1.352526255	6.56E-09	4.13E-08	DOWN
Vitvi007830	2.199776523	1.86E-39	8.33E-38	UP	Vitvi037072	-1.352541061	4.53E-52	3.77E-50	DOWN
Vitvi025416	2.198426759	0.000878979	0.002773626	UP	Vitvi026332	-1.35297617	4.16E-09	2.69E-08	DOWN
Vitvi017177	2.198160695	1.93E-09	1.29E-08	UP	Vitvi008815	-1.353616236	0.00054731	0.001789883	DOWN
Vitvi023013	2.192338156	0.002251104	0.006538746	UP	Vitvi031792	-1.354130021	1.63E-16	1.99E-15	DOWN
Vitvi031465	2.191813338	4.48E-33	1.51E-31	UP	Vitvi008140	-1.354252218	1.57E-21	2.75E-20	DOWN
Vitvi013051	2.186344077	0.00016535	0.000588709	UP	Vitvi023336	-1.354953511	1.33E-11	1.09E-10	DOWN
Vitvi011728	2.183388239	2.78E-27	6.8E-26	UP	Vitvi031796	-1.354976731	5.14E-10	3.61E-09	DOWN
Vitvi031239	2.183225697	4.36E-35	1.62E-33	UP	Vitvi032665	-1.355940764	2.59E-32	8.45E-31	DOWN
Vitvi005923	2.17697449	1.71E-13	1.65E-12	UP	Vitvi000329	-1.356219656	2.08E-40	9.73E-39	DOWN
Vitvi005017	2.176918795	0.008075606	0.020768766	UP	Vitvi032238	-1.356796694	4.06E-19	6.02E-18	DOWN
Vitvi002431	2.176389293	0.000000286	0.0000015	UP	Vitvi001137	-1.35682115	0.01583848	0.037763963	DOWN
Vitvi012829	2.174795973	1.1E-31	3.46E-30	UP	Vitvi018270	-1.357723894	7.07E-42	3.54E-40	DOWN
Vitvi017726	2.173976872	0.0000241	0.0000975	UP	Vitvi003010	-1.357747622	0.000145022	0.000519887	DOWN
Vitvi016395	2.172520436	8.17E-09	5.08E-08	UP	Vitvi035809	-1.358285077	0.00000201	0.00000945	DOWN
Vitvi021458	2.169556975	0.000019	0.0000782	UP	Vitvi017758	-1.358400848	3.36E-14	3.45E-13	DOWN
Vitvi035751	2.169319609	1.68E-60	2.02E-58	UP	Vitvi023359	-1.360050233	2.24E-09	1.48E-08	DOWN
Vitvi023014	2.166870685	9.03E-39	3.95E-37	UP	Vitvi030186	-1.360512256	0.00038469	0.001291327	DOWN
Vitvi035880	2.165176322	3.16E-12	2.75E-11	UP	Vitvi022278	-1.36204585	0.002928993	0.008298746	DOWN
Vitvi016088	2.161387375	2.65E-81	6.26E-79	UP	Vitvi014964	-1.362228171	0.0000869	0.000323132	DOWN
Vitvi016309	2.160978295	4.56E-10	3.22E-09	UP	Vitvi021518	-1.36226985	0.000411904	0.00137518	DOWN
Vitvi012046	2.157335743	2.59E-47	1.74E-45	UP	Vitvi026448	-1.363005045	0.010830199	0.027054852	DOWN
Vitvi015796	2.155085121	0.01210945	0.029854005	UP	Vitvi020291	-1.363201737	0.000232977	0.000811424	DOWN
Vitvi025756	2.154516265	3.72E-08	0.000000215	UP	Vitvi023205	-1.364121074	6.4E-26	1.45E-24	DOWN
Vitvi030665	2.149385885	0.000691892	0.013095504	UP	Vitvi017156	-1.364801481	0.00000111	0.0000054	DOWN
Vitvi030717	2.147824453	1.58E-53	1.42E-51	UP	Vitvi013181	-1.364816533	7.32E-28	1.86E-26	DOWN
Vitvi031752	2.144924598	1.06E-15	1.21E-14	UP	Vitvi023506	-1.365113733	1.37E-19	2.11E-18	DOWN
Vitvi032058	2.140984134	8.12E-26	1.84E-24	UP	Vitvi018338	-1.365905487	0.000000904	0.00000444	DOWN
Vitvi021065	2.139479146	9.28E-10	6.39E-09	UP	Vitvi020121	-1.366008136	0.012342807	0.030362976	DOWN
Vitvi015372	2.138567329	4.85E-18	6.61E-17	UP	Vitvi006468	-1.367879541	1.86E-63	2.41E-61	DOWN
Vitvi002820	2.136216496	5.72E-08	0.000000324	UP	Vitvi035661	-1.367906838	1.26E-18	1.79E-17	DOWN
Vitvi009286	2.132102777	0.00000315	0.0000145	UP	Vitvi037185	-1.3684326	0.002280605	0.006611996	DOWN
Vitvi019319	2.13146705	0.001663815	0.004981635	UP	Vitvi011015	-1.369371573	6.28E-29	1.67E-27	DOWN
Vitvi000343	2.129824344	0.0000912	0.000338032	UP	Vitvi035853	-1.369420505	3.58E-16	4.26E-15	DOWN
Vitvi007245	2.129315341	3.78E-34	1.34E-32	UP	Vitvi033928	-1.369448654	6.29E-47	4.15E-45	DOWN
Vitvi035906	2.12923381	8.65E-40	3.92E-38	UP	Vitvi011993	-1.369754944	3.94E-14	4.03E-13	DOWN
Vitvi008064	2.125173838	3.99E-86	1.16E-83	UP	Vitvi015891	-1.370051598	1.86E-34	6.61E-33	DOWN
Vitvi002425	2.124240427	0.000187771	0.000662385	UP	Vitvi010888	-1.370279393	2.62E-26	6.1E-25	DOWN
Vitvi024233	2.124081644	6.37E-63	8.08E-61	UP	Vitvi001463	-1.37058105	3.82E-12	3.29E-11	DOWN
Vitvi004093	2.123760828	3.4E-41	1.63E-39	UP	Vitvi004085	-1.37068543	0.003032493	0.008564517	DOWN
Vitvi027551	2.12180388	1.03E-16	1.29E-15	UP	Vitvi032942	-1.371241644	2.98E-44	1.69E-42	DOWN
Vitvi004537	2.118216429	0.015019425	0.036018248	UP	Vitvi024633	-1.371567003	0.0000421	0.000164205	DOWN
Vitvi002627	2.110104675	2.46E-94	8.75E-92	UP	Vitvi013357	-1.371948061	1.15E-14	1.21E-13	DOWN
Vitvi026861	2.109219487	0.002618249	0.00749357	UP	Vitvi033944	-1.372816936	0.000000699	0.00000349	DOWN
Vitvi013402	2.108371595	3.84E-36	1.49E-34	UP	Vitvi001686	-1.373055239	8.18E-12	6.82E-11	DOWN
Vitvi031936	2.107089886	0.007330117	0.019020099	UP	Vitvi015679	-1.373641608	0.001055791	0.003283606	DOWN
Vitvi023118	2.106617856	0.012606703	0.03091854	UP	Vitvi027689	-1.373866807	0.00000154	0.00000737	DOWN
Vitvi000617	2.103252023	4.27E-17	5.44E-16	UP	Vitvi025366	-1.374397537	1.09E-41	5.38E-40	DOWN

ID	log2FoldChange	pvalue	padj	change	ID	log2FoldChange	pvalue	padj	change
Vitvi020187	2.099840391	5.17E-17	6.54E-16	UP	Vitvi032709	-1.376330248	7.22E-13	6.67E-12	DOWN
Vitvi019720	2.096506136	5.45E-24	1.11E-22	UP	Vitvi010887	-1.376356607	1.06E-13	1.04E-12	DOWN
Vitvi003880	2.093503319	3.61E-25	7.91E-24	UP	Vitvi007877	-1.376708372	0.000000298	0.00000156	DOWN
Vitvi025434	2.086963333	2.89E-20	4.68E-19	UP	Vitvi011075	-1.377800519	0.00000961	0.0000412	DOWN
Vitvi030657	2.083203764	1.36E-37	5.67E-36	UP	Vitvi016172	-1.379130263	2.71E-40	1.26E-38	DOWN
Vitvi026820	2.082705923	1.83E-54	1.69E-52	UP	Vitvi028738	-1.379545365	8.56E-13	7.86E-12	DOWN
Vitvi012986	2.081151941	1.14E-15	1.3E-14	UP	Vitvi034550	-1.379715247	4.4E-10	3.11E-09	DOWN
Vitvi014426	2.076370921	4.13E-48	2.86E-46	UP	Vitvi035280	-1.381195177	0.0000477	0.000184285	DOWN
Vitvi032899	2.075444713	3.09E-25	6.8E-24	UP	Vitvi005612	-1.381371677	1.65E-09	1.11E-08	DOWN
Vitvi035997	2.07407652	1.23E-16	1.51E-15	UP	Vitvi027109	-1.381483374	8.56E-20	1.34E-18	DOWN
Vitvi021146	2.072581765	9.57E-15	1.02E-13	UP	Vitvi027106	-1.383457157	3.79E-10	2.7E-09	DOWN
Vitvi011870	2.072459704	0.000000873	0.0000043	UP	Vitvi021620	-1.383666061	1.2E-13	1.18E-12	DOWN
Vitvi019980	2.064036634	6.8E-11	5.17E-10	UP	Vitvi031213	-1.385243311	8.44E-10	5.85E-09	DOWN
Vitvi021658	2.062236447	5.92E-15	6.4E-14	UP	Vitvi031079	-1.385280485	5.39E-18	7.31E-17	DOWN
Vitvi022752	2.06220708	8.42E-11	6.34E-10	UP	Vitvi013326	-1.385747914	3.23E-17	4.16E-16	DOWN
Vitvi036470	2.060970839	1E-22	1.87E-21	UP	Vitvi002523	-1.386068456	6.91E-15	7.42E-14	DOWN
Vitvi000607	2.057768931	2.13E-81	5.15E-79	UP	Vitvi015972	-1.38624087	1.63E-34	5.82E-33	DOWN
Vitvi024543	2.053044501	8.35E-72	1.53E-69	UP	Vitvi031061	-1.386632586	3.17E-33	1.08E-31	DOWN
Vitvi004319	2.052579127	2.08E-10	1.52E-09	UP	Vitvi017753	-1.388284677	1.45E-31	4.54E-30	DOWN
Vitvi012894	2.050477189	4.49E-35	1.66E-33	UP	Vitvi000393	-1.388508221	8.34E-14	8.35E-13	DOWN
Vitvi031014	2.046624658	1.11E-68	1.77E-66	UP	Vitvi001176	-1.388793142	4.95E-18	6.75E-17	DOWN
Vitvi036000	2.046597219	1.36E-46	8.73E-45	UP	Vitvi032857	-1.389783986	8.53E-12	7.1E-11	DOWN
Vitvi009770	2.045054105	4.4E-47	2.92E-45	UP	Vitvi002380	-1.389997083	6.35E-14	6.41E-13	DOWN
Vitvi008028	2.043062047	1.86E-52	1.58E-50	UP	Vitvi020058	-1.390182805	0.0000203	0.0000831	DOWN
Vitvi018612	2.041381416	0.00207718	0.030770311	UP	Vitvi031179	-1.390716733	0.0000667	0.000251761	DOWN
Vitvi018300	2.040190816	1.94E-53	1.73E-51	UP	Vitvi030958	-1.39073007	3.53E-14	3.62E-13	DOWN
Vitvi005014	2.038141689	1.56E-40	7.35E-39	UP	Vitvi007751	-1.391715906	0.000000264	0.00000139	DOWN
Vitvi023062	2.037450148	1.99E-33	6.85E-32	UP	Vitvi032826	-1.392378124	0.000000496	0.00000253	DOWN
Vitvi007394	2.036622495	7.04E-47	4.61E-45	UP	Vitvi019834	-1.392515678	2.25E-20	3.68E-19	DOWN
Vitvi031351	2.034623017	4.82E-10	3.39E-09	UP	Vitvi002598	-1.392668135	5.48E-15	5.95E-14	DOWN
Vitvi009513	2.034593154	3.4E-16	4.05E-15	UP	Vitvi020928	-1.392727189	1.72E-11	1.39E-10	DOWN
Vitvi000532	2.032752884	0.000415194	0.001385253	UP	Vitvi009558	-1.39295619	5.77E-11	4.43E-10	DOWN
Vitvi020019	2.026916558	9.29E-70	1.55E-67	UP	Vitvi013855	-1.393283028	0.009261206	0.023540685	DOWN
Vitvi023215	2.026024176	5.15E-65	7.02E-63	UP	Vitvi031189	-1.393600885	5.77E-44	3.18E-42	DOWN
Vitvi004230	2.024733497	3.76E-20	6.04E-19	UP	Vitvi005452	-1.393645211	2.97E-22	5.42E-21	DOWN
Vitvi019913	2.024233639	3.44E-140	3.68E-137	UP	Vitvi024377	-1.393993522	5.98E-24	1.21E-22	DOWN
Vitvi005922	2.022637189	8.87E-22	1.58E-20	UP	Vitvi008073	-1.394117563	0.018915711	0.044142454	DOWN
Vitvi033705	2.017067138	5.58E-19	8.16E-18	UP	Vitvi030659	-1.394998189	2.06E-10	1.5E-09	DOWN
Vitvi015790	2.01239766	0.018132732	0.04253026	UP	Vitvi002331	-1.395442411	1.51E-23	2.97E-22	DOWN
Vitvi006943	2.011901326	5.26E-12	4.46E-11	UP	Vitvi023612	-1.396285607	2.21E-32	7.22E-31	DOWN
Vitvi005783	2.008500035	6.03E-17	7.6E-16	UP	Vitvi019551	-1.39736062	9.65E-14	9.55E-13	DOWN
Vitvi009749	2.007776144	1.61E-23	3.16E-22	UP	Vitvi004279	-1.397382779	1.02E-43	5.51E-42	DOWN
Vitvi016082	2.005403426	9.13E-26	2.07E-24	UP	Vitvi013906	-1.397458685	0.000000134	0.000000734	DOWN
Vitvi007793	2.000330969	4.04E-11	3.15E-10	UP	Vitvi026406	-1.398117443	1.27E-15	1.44E-14	DOWN
Vitvi002461	1.999270043	8.04E-18	1.08E-16	UP	Vitvi007338	-1.398467946	2.79E-08	0.000000164	DOWN
Vitvi011633	1.996995797	2.61E-12	2.28E-11	UP	Vitvi012049	-1.398621423	7.65E-35	2.78E-33	DOWN
Vitvi006349	1.995887036	0.007032546	0.018318169	UP	Vitvi033741	-1.399414109	4.08E-21	6.98E-20	DOWN
Vitvi017259	1.994749457	3.68E-22	6.69E-21	UP	Vitvi000996	-1.400902185	0.010988229	0.027372199	DOWN
Vitvi032685	1.993012664	1.03E-08	6.31E-08	UP	Vitvi035896	-1.400990519	0.002261314	0.006560751	DOWN
Vitvi026767	1.992502417	7.97E-42	3.98E-40	UP	Vitvi012954	-1.401317358	9.44E-22	1.68E-20	DOWN
Vitvi031731	1.991645323	5.86E-31	1.77E-29	UP	Vitvi021863	-1.402034732	5.57E-10	3.9E-09	DOWN
Vitvi021356	1.990264993	0.008664594	0.022171225	UP	Vitvi022385	-1.402055164	8.82E-37	3.53E-35	DOWN
Vitvi000451	1.989666303	0.000000194	0.00000103	UP	Vitvi033685	-1.402103467	0.0000338	0.000133449	DOWN
Vitvi006961	1.989426805	1.12E-24	2.38E-23	UP	Vitvi010165	-1.402602537	2.6E-18	3.61E-17	DOWN
Vitvi005934	1.988019022	2.16E-17	2.82E-16	UP	Vitvi003643	-1.402765769	0.006536674	0.017145637	DOWN
Vitvi030622	1.987820643	3.64E-38	1.55E-36	UP	Vitvi019817	-1.402772213	0.006828098	0.017845126	DOWN
Vitvi029008	1.985570975	1.17E-51	9.53E-50	UP	Vitvi011599	-1.403184899	6.02E-30	1.72E-28	DOWN

ID	log2FoldChange	pvalue	padj	change	ID	log2FoldChange	pvalue	padj	change
Vitvi019900	1.984069755	1.97E-31	6.11E-30	UP	Vitvi025262	-1.404815475	7.41E-47	4.84E-45	DOWN
Vitvi032010	1.98381423	2.63E-98	1.03E-95	UP	Vitvi019137	-1.405178914	2.16E-41	1.05E-39	DOWN
Vitvi009818	1.981767098	1.57E-18	2.22E-17	UP	Vitvi009647	-1.405390775	5.23E-16	6.11E-15	DOWN
Vitvi018644	1.976888282	1.07E-44	6.27E-43	UP	Vitvi012659	-1.406310714	0.000664904	0.002144077	DOWN
Vitvi011377	1.973274416	6.33E-47	4.16E-45	UP	Vitvi026020	-1.406812383	2.45E-09	1.61E-08	DOWN
Vitvi028520	1.971276721	1.1E-15	1.25E-14	UP	Vitvi000184	-1.407610939	0.000774725	0.014300228	DOWN
Vitvi012943	1.969627739	1.57E-10	1.15E-09	UP	Vitvi033299	-1.407916016	1.84E-20	3.03E-19	DOWN
Vitvi005567	1.969045715	1.91E-44	1.09E-42	UP	Vitvi000108	-1.409036139	6.06E-19	8.82E-18	DOWN
Vitvi030440	1.967962013	5.71E-09	3.63E-08	UP	Vitvi017502	-1.409111514	0.000000111	0.000000613	DOWN
Vitvi018123	1.966669648	2.5E-18	3.47E-17	UP	Vitvi000112	-1.411013674	1.51E-10	1.11E-09	DOWN
Vitvi006964	1.963781629	3.17E-38	1.35E-36	UP	Vitvi023057	-1.411248277	5.32E-16	6.21E-15	DOWN
Vitvi019978	1.963655659	0.002356936	0.006810327	UP	Vitvi032016	-1.412330379	0.000000559	0.00000283	DOWN
Vitvi017569	1.958958252	1.96E-81	4.81E-79	UP	Vitvi000930	-1.412335369	3.66E-16	4.35E-15	DOWN
Vitvi024935	1.958735822	3.76E-36	1.46E-34	UP	Vitvi002501	-1.414291409	0.00000127	0.00000609	DOWN
Vitvi036016	1.956820531	0.0000253	0.0001022	UP	Vitvi035912	-1.414392613	1.71E-37	7.04E-36	DOWN
Vitvi027090	1.956786613	1.33E-34	4.77E-33	UP	Vitvi017593	-1.414443481	2.22E-16	2.69E-15	DOWN
Vitvi021186	1.955497883	2.32E-15	2.58E-14	UP	Vitvi018201	-1.414667231	6.82E-19	9.88E-18	DOWN
Vitvi036472	1.954666131	2.46E-49	1.81E-47	UP	Vitvi012633	-1.414946786	2.85E-19	4.26E-18	DOWN
Vitvi028157	1.95462792	0.0000218	0.0000887	UP	Vitvi003721	-1.415202176	3.87E-12	3.33E-11	DOWN
Vitvi009717	1.95077374	0.000024	0.0000973	UP	Vitvi006281	-1.41615885	2.06E-17	2.69E-16	DOWN
Vitvi029688	1.949263432	3.09E-17	3.98E-16	UP	Vitvi018887	-1.416232834	0.000124629	0.000451723	DOWN
Vitvi033052	1.948995765	3.05E-49	2.23E-47	UP	Vitvi017510	-1.416375596	0.0000114	0.0000484	DOWN
Vitvi021553	1.947871564	3.08E-09	2.01E-08	UP	Vitvi033041	-1.41695644	2.49E-24	5.19E-23	DOWN
Vitvi037021	1.946067838	3.93E-15	4.3E-14	UP	Vitvi011109	-1.416984968	2.56E-12	2.24E-11	DOWN
Vitvi019653	1.941225982	0.00632686	0.01664006	UP	Vitvi027626	-1.418721915	1.43E-12	1.28E-11	DOWN
Vitvi031348	1.941063681	0.0000474	0.000183223	UP	Vitvi023332	-1.421788749	0.000000266	0.0000014	DOWN
Vitvi027047	1.93978505	2.94E-44	1.67E-42	UP	Vitvi001242	-1.423282975	6.3E-39	2.78E-37	DOWN
Vitvi015830	1.937723402	1.75E-52	1.49E-50	UP	Vitvi014675	-1.423648248	2.2E-10	1.59E-09	DOWN
Vitvi026685	1.935399272	0.011792602	0.029150705	UP	Vitvi025687	-1.42389629	1.28E-09	8.68E-09	DOWN
Vitvi011353	1.935228993	3.34E-28	8.64E-27	UP	Vitvi019846	-1.424309518	6.35E-09	4.01E-08	DOWN
Vitvi010010	1.932940197	0.001500827	0.00453913	UP	Vitvi029133	-1.425180595	5.54E-25	1.2E-23	DOWN
Vitvi001675	1.932162716	1.99E-44	1.14E-42	UP	Vitvi030136	-1.425500684	0.003003928	0.008487382	DOWN
Vitvi018297	1.931848353	0.000000223	0.00000118	UP	Vitvi023414	-1.426653188	1.43E-26	3.38E-25	DOWN
Vitvi009456	1.930819202	1.39E-32	4.6E-31	UP	Vitvi003619	-1.426819968	0.001569623	0.004724685	DOWN
Vitvi000241	1.930785581	4.81E-42	2.42E-40	UP	Vitvi003949	-1.426820799	1.8E-15	2.01E-14	DOWN
Vitvi034100	1.930238384	1.24E-22	2.31E-21	UP	Vitvi001605	-1.427162187	1.34E-15	1.51E-14	DOWN
Vitvi017522	1.929780047	5.6E-24	1.14E-22	UP	Vitvi018129	-1.427187222	5.8E-15	6.27E-14	DOWN
Vitvi000471	1.929717099	4.18E-15	4.57E-14	UP	Vitvi029215	-1.427282787	1.2E-09	8.18E-09	DOWN
Vitvi010592	1.92939532	4.64E-24	9.47E-23	UP	Vitvi033605	-1.428660802	1.04E-16	1.29E-15	DOWN
Vitvi027639	1.928991604	2.82E-38	1.2E-36	UP	Vitvi006744	-1.428847374	2.43E-11	1.93E-10	DOWN
Vitvi009525	1.925775549	0.000254213	0.000878905	UP	Vitvi017576	-1.428851643	0.000000309	0.00000161	DOWN
Vitvi021861	1.924669758	0.000000952	0.00000466	UP	Vitvi032156	-1.429284108	3.54E-26	8.18E-25	DOWN
Vitvi031725	1.923536145	1.17E-17	1.55E-16	UP	Vitvi031003	-1.429363652	0.000425634	0.00141892	DOWN
Vitvi001414	1.921277416	0.005667102	0.015064806	UP	Vitvi033534	-1.429455356	9.82E-12	8.12E-11	DOWN
Vitvi025757	1.920221711	4.7E-10	3.32E-09	UP	Vitvi007806	-1.429770119	3.96E-10	2.82E-09	DOWN
Vitvi007074	1.918472533	0.000120417	0.000437474	UP	Vitvi021610	-1.430014091	0.000624956	0.002024256	DOWN
Vitvi028116	1.918445105	8.34E-30	2.36E-28	UP	Vitvi003952	-1.430247615	1.88E-34	6.67E-33	DOWN
Vitvi033646	1.918427467	9.81E-28	2.47E-26	UP	Vitvi001815	-1.430710318	3.84E-11	3E-10	DOWN
Vitvi009418	1.917964495	0.005227293	0.014009338	UP	Vitvi005385	-1.432063889	9.36E-27	2.23E-25	DOWN
Vitvi031265	1.917260634	0.002727741	0.007777354	UP	Vitvi021277	-1.432942362	0.0000806	0.000300821	DOWN
Vitvi014079	1.917001608	9.33E-15	9.93E-14	UP	Vitvi022061	-1.433257839	0.00000719	0.0000314	DOWN
Vitvi024859	1.91505076	1.41E-14	1.49E-13	UP	Vitvi005898	-1.433589929	4.8E-42	2.42E-40	DOWN
Vitvi010099	1.913714192	1.1E-19	1.69E-18	UP	Vitvi019914	-1.43366106	7.93E-25	1.7E-23	DOWN
Vitvi000844	1.910541093	1.81E-47	1.24E-45	UP	Vitvi030988	-1.434108534	9.11E-20	1.42E-18	DOWN
Vitvi004599	1.909982627	1.28E-14	1.35E-13	UP	Vitvi006321	-1.436317191	2.23E-24	4.66E-23	DOWN
Vitvi010835	1.909873748	3.41E-09	2.21E-08	UP	Vitvi014733	-1.43640136	0.000064	0.000242572	DOWN
Vitvi015295	1.908904233	1.5E-12	1.35E-11	UP	Vitvi033531	-1.436403828	9.01E-24	1.81E-22	DOWN

ID	log2FoldChange	pvalue	padj	change	ID	log2FoldChange	pvalue	padj	change
Vitvi031444	1.906044326	2.08E-47	1.4E-45	UP	Vitvi020348	-1.437384212	2.49E-45	1.49E-43	DOWN
Vitvi000912	1.904107699	4.91E-12	4.19E-11	UP	Vitvi031132	-1.437881819	2.5E-21	4.31E-20	DOWN
Vitvi025873	1.900681123	0.000000251	0.00000133	UP	Vitvi031655	-1.438626472	0.0000012	0.0000058	DOWN
Vitvi022656	1.900410056	2.92E-29	7.97E-28	UP	Vitvi014656	-1.439690925	2.67E-12	2.34E-11	DOWN
Vitvi033528	1.898994959	0.002522933	0.00724425	UP	Vitvi011901	-1.440522304	3.11E-17	4.01E-16	DOWN
Vitvi026179	1.898446762	1.74E-57	1.86E-55	UP	Vitvi005309	-1.442599112	7.09E-14	7.11E-13	DOWN
Vitvi025232	1.890992278	1.23E-16	1.51E-15	UP	Vitvi004432	-1.443469151	1.21E-17	1.6E-16	DOWN
Vitvi015812	1.884174835	1.51E-08	9.11E-08	UP	Vitvi004977	-1.443487784	4.35E-24	8.93E-23	DOWN
Vitvi031656	1.883601854	1.65E-32	5.44E-31	UP	Vitvi009756	-1.444520143	8.3E-15	8.87E-14	DOWN
Vitvi030656	1.882330834	7.82E-12	6.53E-11	UP	Vitvi026764	-1.444740929	7.21E-41	3.45E-39	DOWN
Vitvi031991	1.881474069	0.005961231	0.015780596	UP	Vitvi018527	-1.444778183	9.11E-21	1.52E-19	DOWN
Vitvi004271	1.877740725	0.000279233	0.000958719	UP	Vitvi002842	-1.445126331	2.02E-37	8.28E-36	DOWN
Vitvi010925	1.875519964	1.88E-22	3.46E-21	UP	Vitvi025349	-1.446113522	3.98E-12	3.42E-11	DOWN
Vitvi029804	1.872319698	1.58E-31	4.92E-30	UP	Vitvi003882	-1.446862972	0.001684822	0.005035626	DOWN
Vitvi004682	1.87011792	0.002080003	0.006089511	UP	Vitvi005613	-1.447031686	0.000307323	0.0010479	DOWN
Vitvi026386	1.868847937	0.014763547	0.035484223	UP	Vitvi012776	-1.447595664	4.93E-28	1.26E-26	DOWN
Vitvi005130	1.867712276	7.2E-28	1.83E-26	UP	Vitvi033772	-1.447629245	4.78E-16	5.6E-15	DOWN
Vitvi023775	1.867212966	3.49E-51	2.79E-49	UP	Vitvi006541	-1.448587782	0.001611845	0.004841018	DOWN
Vitvi000171	1.866594284	0.008913672	0.022748399	UP	Vitvi004583	-1.449271601	4.06E-49	2.93E-47	DOWN
Vitvi031312	1.865600467	0.007802503	0.020132677	UP	Vitvi017560	-1.450345291	2.01E-47	1.36E-45	DOWN
Vitvi010492	1.865366836	0.0000286	0.000114303	UP	Vitvi032662	-1.450629233	5.5E-11	4.23E-10	DOWN
Vitvi019593	1.864858633	0.0000027	0.0000125	UP	Vitvi018930	-1.45069124	5.98E-21	1.01E-19	DOWN
Vitvi017937	1.864107816	4.29E-19	6.35E-18	UP	Vitvi018745	-1.451632154	0.01372746	0.033321244	DOWN
Vitvi019474	1.863098899	3.92E-29	1.06E-27	UP	Vitvi020849	-1.452396295	1.17E-11	9.55E-11	DOWN
Vitvi016800	1.862127528	0.021353639	0.049026237	UP	Vitvi019534	-1.452467139	0.014898138	0.035765392	DOWN
Vitvi018066	1.861656771	1.38E-28	3.62E-27	UP	Vitvi004887	-1.452732381	3.23E-30	9.36E-29	DOWN
Vitvi021613	1.861582464	4.37E-13	4.08E-12	UP	Vitvi009214	-1.453376777	0.000000577	0.00000291	DOWN
Vitvi016017	1.860701474	0.000260763	0.000900172	UP	Vitvi011642	-1.453529087	0.004406022	0.012030532	DOWN
Vitvi032465	1.857506364	3.69E-08	0.000000214	UP	Vitvi026925	-1.45363621	5.75E-19	8.4E-18	DOWN
Vitvi016752	1.855546534	0.010566011	0.02645997	UP	Vitvi003629	-1.454351635	0.00000176	0.00000832	DOWN
Vitvi036335	1.855296046	9.8E-20	1.52E-18	UP	Vitvi033273	-1.454373155	8.63E-10	5.97E-09	DOWN
Vitvi003309	1.851146069	4.14E-43	2.19E-41	UP	Vitvi002156	-1.454558592	0.001647413	0.004938496	DOWN
Vitvi007184	1.850109175	1.15E-34	4.11E-33	UP	Vitvi012913	-1.455969224	7.01E-20	1.1E-18	DOWN
Vitvi000713	1.849294491	3.03E-55	2.88E-53	UP	Vitvi034601	-1.456155453	5.14E-14	5.22E-13	DOWN
Vitvi030743	1.849005921	1.81E-33	6.24E-32	UP	Vitvi028681	-1.456409504	0.000000241	0.00000127	DOWN
Vitvi005398	1.847898044	0.00000215	0.0000101	UP	Vitvi023688	-1.456433924	2.42E-09	0.000000016	DOWN
Vitvi019788	1.847639279	3.9E-24	8.02E-23	UP	Vitvi030024	-1.456771411	1.59E-10	1.17E-09	DOWN
Vitvi024713	1.847003807	1.06E-18	1.51E-17	UP	Vitvi006313	-1.457692608	1.29E-21	2.27E-20	DOWN
Vitvi008070	1.844793806	0.000101317	0.000373156	UP	Vitvi003045	-1.458050386	3.04E-25	6.7E-24	DOWN
Vitvi003317	1.844592535	8.42E-30	2.38E-28	UP	Vitvi005872	-1.458855067	6.9E-09	4.33E-08	DOWN
Vitvi037365	1.844009467	2.02E-08	0.00000012	UP	Vitvi015658	-1.459279742	1.1E-11	9.08E-11	DOWN
Vitvi033647	1.842611163	8.42E-60	9.83E-58	UP	Vitvi004169	-1.460866328	2E-17	2.62E-16	DOWN
Vitvi030748	1.841217114	9.4E-13	8.59E-12	UP	Vitvi028839	-1.461123293	1.04E-28	7.62E-26	DOWN
Vitvi030889	1.839099449	0.007760073	0.020033375	UP	Vitvi025666	-1.46112721	0.000000294	0.00000154	DOWN
Vitvi031385	1.838282515	7.9E-19	1.14E-17	UP	Vitvi031158	-1.46182363	3.3E-20	5.33E-19	DOWN
Vitvi005158	1.83745704	3.62E-58	3.99E-56	UP	Vitvi033343	-1.4619112	6.37E-51	5.07E-49	DOWN
Vitvi030804	1.837279597	1.58E-16	1.93E-15	UP	Vitvi000360	-1.462035597	1.65E-15	1.85E-14	DOWN
Vitvi006650	1.836826048	0.001890471	0.005579663	UP	Vitvi035792	-1.4624477	0.020705414	0.047715948	DOWN
Vitvi013459	1.835199962	3.35E-10	2.39E-09	UP	Vitvi006542	-1.463646909	6.05E-37	2.44E-35	DOWN
Vitvi003391	1.832521135	5.24E-34	1.84E-32	UP	Vitvi032681	-1.464557203	6.34E-11	4.84E-10	DOWN
Vitvi021261	1.830362125	1.6E-27	3.96E-26	UP	Vitvi033535	-1.465298851	3.63E-31	1.11E-29	DOWN
Vitvi018856	1.830356323	5.28E-52	4.38E-50	UP	Vitvi007619	-1.465965706	0.007892201	0.020338288	DOWN
Vitvi027761	1.824995196	0.00000114	0.00000554	UP	Vitvi027684	-1.467074993	0.0000722	0.000271318	DOWN
Vitvi021278	1.823072376	0.000834168	0.002643311	UP	Vitvi014337	-1.467435178	0.01434728	0.034635364	DOWN
Vitvi027271	1.820124664	0.00000313	0.0000144	UP	Vitvi021565	-1.467974586	0.009133716	0.023251541	DOWN
Vitvi012927	1.820015091	9.47E-34	3.3E-32	UP	Vitvi013221	-1.468136628	1.31E-11	1.07E-10	DOWN
Vitvi018044	1.818521806	1.6E-66	2.41E-64	UP	Vitvi030077	-1.468523123	1.79E-12	1.6E-11	DOWN

ID	log2FoldChange	pvalue	padj	change	ID	log2FoldChange	pvalue	padj	change
Vitvi030745	1.817065397	1.47E-09	9.94E-09	UP	Vitvi021324	-1.468761841	1.37E-27	3.42E-26	DOWN
Vitvi030769	1.815011206	3.94E-57	4.17E-55	UP	Vitvi015365	-1.470068173	0.000637211	0.002061649	DOWN
Vitvi010081	1.814618768	7.94E-50	5.93E-48	UP	Vitvi014465	-1.470333897	1.12E-66	1.71E-64	DOWN
Vitvi018301	1.814540292	0.01088221	0.027162945	UP	Vitvi021901	-1.472317601	0.0000064	0.0000281	DOWN
Vitvi033611	1.813092577	5.01E-45	2.97E-43	UP	Vitvi009465	-1.473389622	0.000000512	0.00000261	DOWN
Vitvi018712	1.811637959	1.08E-31	3.41E-30	UP	Vitvi003834	-1.474205942	3.94E-33	1.33E-31	DOWN
Vitvi013220	1.810880958	3.91E-37	1.58E-35	UP	Vitvi003998	-1.474389486	0.00000264	0.0000122	DOWN
Vitvi002138	1.81087345	1.08E-12	9.79E-12	UP	Vitvi032803	-1.474431231	9.3E-23	1.75E-21	DOWN
Vitvi007048	1.809984388	2.59E-26	6.05E-25	UP	Vitvi029386	-1.474878834	0.00000199	0.00000936	DOWN
Vitvi025651	1.806784537	2.47E-33	8.44E-32	UP	Vitvi021425	-1.475159043	2.52E-11	2.01E-10	DOWN
Vitvi010086	1.805844669	9.56E-49	6.79E-47	UP	Vitvi031366	-1.475575876	3.62E-17	4.63E-16	DOWN
Vitvi008021	1.805298366	4.97E-17	6.29E-16	UP	Vitvi025094	-1.476957955	2.49E-11	1.98E-10	DOWN
Vitvi017712	1.80474008	3.41E-22	6.22E-21	UP	Vitvi031450	-1.477256017	1.93E-29	5.31E-28	DOWN
Vitvi005508	1.803727395	2.76E-12	2.41E-11	UP	Vitvi017575	-1.477428528	0.002314502	0.006702618	DOWN
Vitvi031600	1.801753574	5.7E-09	3.62E-08	UP	Vitvi000311	-1.478261803	0.014002359	0.033915564	DOWN
Vitvi022268	1.801524193	0.002164238	0.006312463	UP	Vitvi031358	-1.478276908	5.02E-25	1.09E-23	DOWN
Vitvi005425	1.799616787	5.66E-09	0.000000036	UP	Vitvi021228	-1.478318359	0.0000387	0.000151753	DOWN
Vitvi001529	1.796952344	9.06E-24	1.81E-22	UP	Vitvi034087	-1.47994793	0.0000015	0.00000719	DOWN
Vitvi033669	1.79667692	5.48E-09	3.49E-08	UP	Vitvi002253	-1.480653183	4.99E-08	0.000000285	DOWN
Vitvi005958	1.795552141	2.17E-09	1.44E-08	UP	Vitvi019477	-1.480678533	8.31E-08	0.000000463	DOWN
Vitvi034052	1.795528078	1.54E-37	6.36E-36	UP	Vitvi007131	-1.481718175	4.15E-60	4.95E-58	DOWN
Vitvi035754	1.794885603	4E-28	1.03E-26	UP	Vitvi008312	-1.483810209	0.00000247	0.0000115	DOWN
Vitvi016069	1.786679745	7.13E-31	2.13E-29	UP	Vitvi019869	-1.483851608	0.017671992	0.04160815	DOWN
Vitvi017725	1.784435658	1.87E-32	6.14E-31	UP	Vitvi021902	-1.48389141	0.000596549	0.001938425	DOWN
Vitvi030992	1.784008962	4.72E-11	3.64E-10	UP	Vitvi029579	-1.484541598	0.0000249	0.000100705	DOWN
Vitvi009168	1.783486409	4E-10	2.84E-09	UP	Vitvi012664	-1.484562424	2.32E-19	3.49E-18	DOWN
Vitvi008273	1.783040335	0.0000344	0.000135534	UP	Vitvi023161	-1.484660619	1.06E-08	6.48E-08	DOWN
Vitvi031367	1.783033795	0.000257228	0.000888878	UP	Vitvi018330	-1.48494085	5.26E-14	5.33E-13	DOWN
Vitvi033058	1.782660056	1.18E-61	1.46E-59	UP	Vitvi023308	-1.48624878	2.84E-32	9.24E-31	DOWN
Vitvi032845	1.781367349	0.0000356	0.000140207	UP	Vitvi021724	-1.486595265	5.29E-42	2.66E-40	DOWN
Vitvi005563	1.778916614	2.16E-22	3.97E-21	UP	Vitvi013020	-1.487201084	0.001428647	0.004338917	DOWN
Vitvi000767	1.778684624	1.37E-30	4.05E-29	UP	Vitvi007502	-1.488074063	3.21E-23	6.18E-22	DOWN
Vitvi007863	1.776670514	4.46E-35	1.65E-33	UP	Vitvi022016	-1.488532404	0.019919746	0.046204166	DOWN
Vitvi017333	1.776513044	9.99E-10	6.86E-09	UP	Vitvi026737	-1.488610652	5.95E-24	1.2E-22	DOWN
Vitvi017360	1.773508288	7.73E-22	1.38E-20	UP	Vitvi014674	-1.488898481	0.009859108	0.024870615	DOWN
Vitvi012828	1.773167009	2.41E-17	3.14E-16	UP	Vitvi015311	-1.490022733	1.14E-11	9.36E-11	DOWN
Vitvi020060	1.770391537	3.52E-12	3.05E-11	UP	Vitvi023159	-1.491137365	0.0000163	0.0000676	DOWN
Vitvi000194	1.76997998	4.19E-22	7.58E-21	UP	Vitvi025056	-1.491367192	4.3E-18	5.88E-17	DOWN
Vitvi029642	1.768864643	0.001724207	0.005147281	UP	Vitvi018405	-1.491597265	0.00000684	0.0000299	DOWN
Vitvi005074	1.768693205	2.68E-11	2.13E-10	UP	Vitvi011769	-1.491879051	8.26E-44	4.51E-42	DOWN
Vitvi023920	1.767715986	0.001766438	0.005254046	UP	Vitvi033522	-1.492415136	1.38E-18	1.96E-17	DOWN
Vitvi007154	1.766237011	1.27E-46	8.14E-45	UP	Vitvi029574	-1.49379323	2.91E-26	6.76E-25	DOWN
Vitvi021164	1.765056046	7.76E-22	1.38E-20	UP	Vitvi037110	-1.494277647	7.92E-20	1.24E-18	DOWN
Vitvi019820	1.763300827	0.000722989	0.01353023	UP	Vitvi020139	-1.496496903	0.000996676	0.003112619	DOWN
Vitvi027361	1.76156053	8.07E-20	1.27E-18	UP	Vitvi002903	-1.496574365	3.78E-39	1.68E-37	DOWN
Vitvi014273	1.761548453	0.001583525	0.004761594	UP	Vitvi018669	-1.497875822	3.68E-25	8.05E-24	DOWN
Vitvi002629	1.761459467	1.26E-19	1.95E-18	UP	Vitvi021131	-1.498237294	1.6E-23	3.13E-22	DOWN
Vitvi009135	1.760266097	0.001742271	0.00519281	UP	Vitvi013602	-1.498327896	2.36E-20	3.84E-19	DOWN
Vitvi008075	1.756672407	0.000480201	0.00158754	UP	Vitvi002249	-1.498863077	4.74E-31	1.44E-29	DOWN
Vitvi033131	1.753533392	3.69E-52	3.1E-50	UP	Vitvi021809	-1.49909149	0.000516299	0.001696935	DOWN
Vitvi002240	1.75338975	1.98E-42	1.02E-40	UP	Vitvi002785	-1.499814031	9.94E-11	7.43E-10	DOWN
Vitvi008015	1.748911037	0.009509524	0.024078466	UP	Vitvi036048	-1.500078136	1.79E-95	6.48E-93	DOWN
Vitvi004623	1.748717983	1.1E-92	3.8E-90	UP	Vitvi019919	-1.502314682	6.52E-12	5.48E-11	DOWN
Vitvi001876	1.74595149	6.35E-09	4.01E-08	UP	Vitvi002154	-1.502344676	7.74E-27	1.85E-25	DOWN
Vitvi033166	1.745720772	2.18E-09	1.44E-08	UP	Vitvi000965	-1.50281803	1.58E-22	2.93E-21	DOWN
Vitvi005233	1.745623762	1.76E-30	5.16E-29	UP	Vitvi007968	-1.502969749	1.4E-21	2.46E-20	DOWN
Vitvi032145	1.741796035	5.25E-25	1.14E-23	UP	Vitvi013956	-1.503441471	0.017677635	0.041616611	DOWN

ID	log2FoldChange	pvalue	padj	change	ID	log2FoldChange	pvalue	padj	change
Vitvi019714	1.741011946	3.71E-16	4.4E-15	UP	Vitvi009019	-1.503468821	0.000463014	0.001531968	DOWN
Vitvi014851	1.739336386	0.01799137	0.042247481	UP	Vitvi001107	-1.5038679	0.00000221	0.0000104	DOWN
Vitvi005930	1.737526994	0.00000679	0.0000297	UP	Vitvi028401	-1.503905726	0.0000206	0.0000841	DOWN
Vitvi026166	1.733019973	0.003647612	0.010135471	UP	Vitvi006900	-1.504415661	3.8E-49	2.76E-47	DOWN
Vitvi009837	1.728728588	4.62E-29	1.24E-27	UP	Vitvi021147	-1.506226642	3.11E-46	1.93E-44	DOWN
Vitvi000285	1.72391504	5.48E-27	1.32E-25	UP	Vitvi011954	-1.507429705	0.00000106	0.00000518	DOWN
Vitvi026379	1.723361504	1.52E-26	3.59E-25	UP	Vitvi008122	-1.508046001	1.34E-24	2.84E-23	DOWN
Vitvi033932	1.721928113	5.41E-56	5.33E-54	UP	Vitvi031674	-1.508586258	3.54E-13	3.33E-12	DOWN
Vitvi026633	1.720091722	2.47E-54	2.27E-52	UP	Vitvi017326	-1.509419461	0.000000857	0.00000423	DOWN
Vitvi002312	1.719498313	6.8E-09	4.27E-08	UP	Vitvi010067	-1.509604143	1.07E-37	4.48E-36	DOWN
Vitvi037048	1.71922113	6.11E-10	4.27E-09	UP	Vitvi031306	-1.509993295	5.2E-36	2E-34	DOWN
Vitvi027456	1.718507363	5E-26	1.15E-24	UP	Vitvi015505	-1.51077709	0.00000297	0.0000137	DOWN
Vitvi007373	1.715035167	4.51E-11	3.49E-10	UP	Vitvi037061	-1.510866257	2.36E-18	3.29E-17	DOWN
Vitvi032765	1.71358242	5.81E-22	1.04E-20	UP	Vitvi015695	-1.511873573	0.00000325	0.0000149	DOWN
Vitvi008265	1.711767122	4.32E-15	4.71E-14	UP	Vitvi032212	-1.51190471	1.35E-33	4.67E-32	DOWN
Vitvi002493	1.710869342	5.1E-10	3.59E-09	UP	Vitvi023449	-1.512716612	0.0000716	0.000269067	DOWN
Vitvi004983	1.710536135	1.1E-36	4.38E-35	UP	Vitvi008494	-1.514653276	3.1E-20	5.02E-19	DOWN
Vitvi023595	1.710483007	7.61E-31	2.27E-29	UP	Vitvi032928	-1.515873663	0.000000309	0.00000161	DOWN
Vitvi010023	1.7099462	0.000000888	0.00000437	UP	Vitvi004491	-1.51608649	2.02E-100	8.21E-98	DOWN
Vitvi004600	1.708858174	6.89E-11	5.23E-10	UP	Vitvi002567	-1.516650701	1.09E-46	7.04E-45	DOWN
Vitvi016732	1.707935843	0.014457302	0.034863672	UP	Vitvi015309	-1.516710621	0.000000891	0.00000438	DOWN
Vitvi034106	1.70756023	0.00000107	0.00000519	UP	Vitvi021958	-1.517569569	1.37E-46	8.77E-45	DOWN
Vitvi032135	1.707008879	1.5E-13	1.46E-12	UP	Vitvi010900	-1.518971269	8.2E-47	5.34E-45	DOWN
Vitvi002122	1.704796626	4.84E-21	8.25E-20	UP	Vitvi013247	-1.52060047	0.002757489	0.007858864	DOWN
Vitvi016613	1.704396897	4.42E-12	3.78E-11	UP	Vitvi001667	-1.521207413	0.0000633	0.000239748	DOWN
Vitvi034000	1.703227826	0.000503654	0.001659404	UP	Vitvi036209	-1.523436941	2.01E-24	4.21E-23	DOWN
Vitvi019671	1.702802372	4.99E-28	1.28E-26	UP	Vitvi024395	-1.52436859	0.007862408	0.020274372	DOWN
Vitvi004342	1.702774619	5.64E-19	8.25E-18	UP	Vitvi032175	-1.524522542	3.13E-08	0.000000183	DOWN
Vitvi028474	1.702494346	2.66E-41	1.29E-39	UP	Vitvi001553	-1.52487703	0.000442776	0.001471235	DOWN
Vitvi035740	1.702469254	1.2E-66	1.82E-64	UP	Vitvi000452	-1.525473127	7.74E-24	1.56E-22	DOWN
Vitvi004196	1.701370715	0.00000015	0.000000813	UP	Vitvi016166	-1.526805121	1.47E-55	1.42E-53	DOWN
Vitvi021260	1.700187772	1.8E-11	1.45E-10	UP	Vitvi027209	-1.527727337	0.00000243	0.0000113	DOWN
Vitvi029685	1.699668388	0.000358493	0.001208984	UP	Vitvi031077	-1.528621658	0.009433218	0.023921039	DOWN
Vitvi011872	1.699096334	0.011148639	0.027727581	UP	Vitvi015805	-1.52923847	0.016428801	0.039024886	DOWN
Vitvi030676	1.69582795	0.00000801	0.0000347	UP	Vitvi013305	-1.530603413	0.0000991	0.000365691	DOWN
Vitvi014980	1.694186314	8.82E-29	2.34E-27	UP	Vitvi009497	-1.531601907	8.31E-41	3.95E-39	DOWN
Vitvi026042	1.69341734	2.18E-17	2.84E-16	UP	Vitvi017757	-1.531865869	0.004899988	0.013222872	DOWN
Vitvi000146	1.692784118	2.99E-50	2.26E-48	UP	Vitvi033114	-1.532911896	1.1E-10	1.61E-08	DOWN
Vitvi030512	1.689944494	9.78E-10	6.72E-09	UP	Vitvi032067	-1.533216845	0.0000179	0.000074	DOWN
Vitvi006079	1.686735906	2.42E-20	3.94E-19	UP	Vitvi021392	-1.535429316	1.46E-09	9.88E-09	DOWN
Vitvi018168	1.685458897	9.97E-47	6.47E-45	UP	Vitvi002100	-1.537548342	9.58E-18	1.27E-16	DOWN
Vitvi002494	1.684642813	2.28E-11	1.82E-10	UP	Vitvi016007	-1.539088828	0.0000768	0.000287614	DOWN
Vitvi001870	1.684363341	4.64E-56	4.62E-54	UP	Vitvi029291	-1.53927726	1E-18	1.43E-17	DOWN
Vitvi031026	1.684314506	0.015053762	0.036087809	UP	Vitvi032801	-1.540900482	1.38E-09	9.32E-09	DOWN
Vitvi028735	1.684263267	0.002247119	0.006531695	UP	Vitvi019646	-1.540932859	3.3E-26	7.64E-25	DOWN
Vitvi019750	1.683051755	1.82E-48	1.28E-46	UP	Vitvi023350	-1.541057374	8.76E-08	0.000000487	DOWN
Vitvi030946	1.682487355	3.7E-10	2.64E-09	UP	Vitvi036218	-1.541304354	1.21E-08	7.35E-08	DOWN
Vitvi000704	1.682305146	0.000000998	0.00000487	UP	Vitvi034701	-1.542240425	3.97E-10	2.82E-09	DOWN
Vitvi028806	1.680006883	1.71E-37	7.05E-36	UP	Vitvi005178	-1.542761728	1.09E-23	2.16E-22	DOWN
Vitvi017979	1.679958586	1.08E-58	1.22E-56	UP	Vitvi001784	-1.54289319	1.04E-15	1.18E-14	DOWN
Vitvi033219	1.678650595	4.77E-09	3.06E-08	UP	Vitvi032252	-1.543046868	0.000536945	0.001758818	DOWN
Vitvi014345	1.677273889	0.000126151	0.000456916	UP	Vitvi009182	-1.543278027	6.11E-20	9.67E-19	DOWN
Vitvi021925	1.67507164	3.57E-37	1.45E-35	UP	Vitvi020026	-1.543568739	4.74E-12	4.04E-11	DOWN
Vitvi036597	1.673697128	4.73E-42	2.4E-40	UP	Vitvi014421	-1.543746275	6.6E-16	7.64E-15	DOWN
Vitvi031181	1.673667472	7.82E-37	3.15E-35	UP	Vitvi007919	-1.543959664	2.04E-18	2.86E-17	DOWN
Vitvi016146	1.672331627	4.55E-12	3.88E-11	UP	Vitvi018165	-1.544607365	2.24E-14	2.33E-13	DOWN
Vitvi016027	1.670856877	0.000000642	0.00000322	UP	Vitvi000760	-1.546159343	1.37E-74	2.62E-72	DOWN

ID	log2FoldChange	pvalue	padj	change	ID	log2FoldChange	pvalue	padj	change
Vitvi031719	1.669330299	0.000793484	0.002525819	UP	Vitvi008199	-1.54664086	4.71E-16	5.52E-15	DOWN
Vitvi022823	1.667573299	0.00000119	0.00000575	UP	Vitvi027671	-1.547211381	5.89E-16	6.85E-15	DOWN
Vitvi021184	1.665059159	9.9E-12	8.18E-11	UP	Vitvi008084	-1.54728929	1.4E-19	2.14E-18	DOWN
Vitvi036442	1.664241194	0.000000217	0.00000115	UP	Vitvi013166	-1.547333657	0.000000192	0.00000103	DOWN
Vitvi006548	1.663950352	6.56E-18	8.84E-17	UP	Vitvi002604	-1.547365691	0.021521885	0.049356727	DOWN
Vitvi000467	1.663054979	0.002286366	0.006626973	UP	Vitvi026981	-1.54747991	5.87E-76	1.21E-73	DOWN
Vitvi006663	1.661434541	0.000000737	0.00000366	UP	Vitvi032902	-1.547958358	1.87E-13	1.8E-12	DOWN
Vitvi030747	1.661403121	1.07E-52	9.21E-51	UP	Vitvi010696	-1.548303297	0.000000034	0.000000198	DOWN
Vitvi005694	1.660239534	0.000175976	0.000624244	UP	Vitvi005520	-1.548468687	0.00000408	0.0000184	DOWN
Vitvi010012	1.658535088	0.001892556	0.005584196	UP	Vitvi013461	-1.549026953	2.75E-17	3.56E-16	DOWN
Vitvi020166	1.656847771	7.46E-37	3.01E-35	UP	Vitvi001526	-1.550284753	8.33E-15	8.9E-14	DOWN
Vitvi009192	1.656720768	3.22E-32	1.04E-30	UP	Vitvi016357	-1.550742639	0.000877347	0.002768903	DOWN
Vitvi008741	1.65242894	0.0000291	0.000116337	UP	Vitvi001394	-1.550917931	2.79E-12	2.43E-11	DOWN
Vitvi021443	1.651851422	4.38E-33	1.48E-31	UP	Vitvi028834	-1.551550793	1.36E-64	1.8E-62	DOWN
Vitvi021813	1.651294773	5.37E-16	6.26E-15	UP	Vitvi012785	-1.551683751	7.72E-24	1.55E-22	DOWN
Vitvi032264	1.650834692	7.84E-32	2.5E-30	UP	Vitvi002238	-1.552831014	0.000136186	0.000490375	DOWN
Vitvi019951	1.650571036	0.000000182	0.000000974	UP	Vitvi036358	-1.554138914	0.000674452	0.002172107	DOWN
Vitvi000728	1.649419144	3.61E-66	5.13E-64	UP	Vitvi023339	-1.554404288	1.81E-08	0.000000109	DOWN
Vitvi017835	1.645473413	4.67E-30	1.34E-28	UP	Vitvi011348	-1.554608984	0.000604376	0.00196229	DOWN
Vitvi002171	1.645440795	0.00000324	0.0000148	UP	Vitvi021614	-1.554636167	0.00000459	0.0000206	DOWN
Vitvi013637	1.645195745	1.31E-51	1.06E-49	UP	Vitvi003597	-1.554719297	0.012225754	0.030104197	DOWN
Vitvi023455	1.643785051	1.15E-43	6.21E-42	UP	Vitvi013211	-1.555064894	0.0000089	0.0000384	DOWN
Vitvi016748	1.643231477	0.014588448	0.035121554	UP	Vitvi014747	-1.555210292	6.85E-09	0.000000043	DOWN
Vitvi018102	1.641704101	8.1E-19	1.16E-17	UP	Vitvi007352	-1.556118074	0.000321728	0.001093529	DOWN
Vitvi036467	1.638390235	0.00000132	0.00000633	UP	Vitvi036436	-1.556574133	0.00000502	0.0000224	DOWN
Vitvi021445	1.638103565	0.000000335	0.00000174	UP	Vitvi005383	-1.556618223	1.04E-13	1.02E-12	DOWN
Vitvi017901	1.636568365	0.00527203	0.014119921	UP	Vitvi013088	-1.556834968	8.32E-66	1.15E-63	DOWN
Vitvi010933	1.634807282	3.82E-33	1.29E-31	UP	Vitvi007565	-1.556918576	4E-31	1.22E-29	DOWN
Vitvi008035	1.633043336	1.07E-08	6.54E-08	UP	Vitvi017731	-1.557950973	0.0000275	0.000110244	DOWN
Vitvi021417	1.632874008	8.96E-30	2.52E-28	UP	Vitvi002230	-1.558540149	8.72E-13	7.98E-12	DOWN
Vitvi005231	1.631789135	1.6E-40	7.51E-39	UP	Vitvi021505	-1.558668179	0.019002578	0.0443248	DOWN
Vitvi023104	1.630379267	4.06E-18	5.56E-17	UP	Vitvi014928	-1.55949535	0.00042103	0.001404495	DOWN
Vitvi001061	1.629600321	2.02E-45	1.21E-43	UP	Vitvi025742	-1.559642259	0.00014286	0.0005125	DOWN
Vitvi001450	1.629556366	1.62E-13	1.57E-12	UP	Vitvi012544	-1.560071753	6.95E-23	1.32E-21	DOWN
Vitvi011414	1.629352542	9.26E-24	1.85E-22	UP	Vitvi015672	-1.560662963	1.33E-49	9.89E-48	DOWN
Vitvi005301	1.629050027	0.000344523	0.001165161	UP	Vitvi007302	-1.560959557	1.7E-19	2.58E-18	DOWN
Vitvi032098	1.628494365	0.00000936	0.0000401	UP	Vitvi003623	-1.561240226	0.001740751	0.005189043	DOWN
Vitvi015012	1.628285628	1.91E-11	1.54E-10	UP	Vitvi015292	-1.562027036	2.32E-09	1.53E-08	DOWN
Vitvi015867	1.628107202	0.001694036	0.026362679	UP	Vitvi006982	-1.562043862	3.6E-17	4.61E-16	DOWN
Vitvi005994	1.62774413	1.56E-16	1.91E-15	UP	Vitvi031995	-1.562084506	0.004114335	0.011303986	DOWN
Vitvi009686	1.626882541	2.22E-14	2.3E-13	UP	Vitvi009860	-1.563948594	4.71E-21	8.03E-20	DOWN
Vitvi033696	1.626797531	0.000370847	0.00124878	UP	Vitvi027107	-1.564902498	4.8E-09	3.08E-08	DOWN
Vitvi016611	1.625369639	1.6E-13	1.56E-12	UP	Vitvi029262	-1.567055335	0.0000891	0.000330706	DOWN
Vitvi016703	1.625101669	0.00000114	0.00000554	UP	Vitvi000323	-1.56772826	1.69E-08	0.000000101	DOWN
Vitvi000066	1.624807893	0.0000689	0.000259726	UP	Vitvi028663	-1.567800431	9.46E-12	7.84E-11	DOWN
Vitvi035597	1.623535466	1.08E-23	2.14E-22	UP	Vitvi026698	-1.567860067	0.0000516	0.000198475	DOWN
Vitvi017524	1.623252766	6.8E-18	9.15E-17	UP	Vitvi025004	-1.568075319	6.09E-12	5.14E-11	DOWN
Vitvi025949	1.622584459	5.58E-40	2.55E-38	UP	Vitvi033899	-1.56923816	6.73E-45	3.96E-43	DOWN
Vitvi005227	1.622238679	0.000000558	0.00000282	UP	Vitvi006609	-1.572599431	0.00000226	0.0000106	DOWN
Vitvi032598	1.618062674	3.56E-15	3.91E-14	UP	Vitvi019581	-1.572837714	2.04E-42	1.04E-40	DOWN
Vitvi002203	1.616049771	0.000000219	0.00000116	UP	Vitvi005315	-1.573596603	2.02E-23	3.94E-22	DOWN
Vitvi032464	1.613112463	1.26E-30	3.74E-29	UP	Vitvi034058	-1.57390832	7.73E-20	1.22E-18	DOWN
Vitvi005816	1.611613962	4.12E-13	3.86E-12	UP	Vitvi027687	-1.576332842	0.00000987	0.0000422	DOWN
Vitvi002310	1.610775793	4.31E-28	1.11E-26	UP	Vitvi010704	-1.576613753	0.0000115	0.0000487	DOWN
Vitvi033156	1.610087822	3.18E-19	4.75E-18	UP	Vitvi021334	-1.576900811	6.62E-38	2.8E-36	DOWN
Vitvi033072	1.607864498	9.04E-14	8.99E-13	UP	Vitvi015104	-1.576909985	3.06E-20	4.95E-19	DOWN
Vitvi016302	1.607073041	1.42E-11	1.16E-10	UP	Vitvi031930	-1.57801832	9.54E-27	2.27E-25	DOWN

ID	log2FoldChange	pvalue	padj	change	ID	log2FoldChange	pvalue	padj	change
Vitvi030749	1.606788412	3.9E-43	2.06E-41	UP	Vitvi031523	-1.578783169	0.006304375	0.016586662	DOWN
Vitvi031048	1.604463245	9.13E-44	4.96E-42	UP	Vitvi018526	-1.578916252	4.58E-13	4.26E-12	DOWN
Vitvi020895	1.603812191	0.00000482	0.0000216	UP	Vitvi025137	-1.580244622	4.25E-13	3.97E-12	DOWN
Vitvi009536	1.603478907	1.31E-28	3.45E-27	UP	Vitvi009251	-1.580268413	6.72E-48	4.64E-46	DOWN
Vitvi018373	1.602629753	9.1E-28	2.3E-26	UP	Vitvi027076	-1.580494002	6.92E-31	2.08E-29	DOWN
Vitvi033176	1.601278007	0.002300825	0.006665862	UP	Vitvi007779	-1.581193871	1.93E-18	2.72E-17	DOWN
Vitvi010183	1.598712612	5.48E-27	1.32E-25	UP	Vitvi003883	-1.581539432	1.02E-50	7.98E-49	DOWN
Vitvi022253	1.597913607	2.97E-11	2.34E-10	UP	Vitvi010929	-1.582796871	0.000000237	0.00000125	DOWN
Vitvi011189	1.596846387	0.00025047	0.000866704	UP	Vitvi021486	-1.583795791	5.78E-16	6.73E-15	DOWN
Vitvi011349	1.596487058	5.29E-17	6.68E-16	UP	Vitvi006138	-1.584004786	3.84E-12	3.31E-11	DOWN
Vitvi003504	1.596328852	1.35E-23	2.67E-22	UP	Vitvi025343	-1.584133231	0.0000219	0.0000893	DOWN
Vitvi033960	1.595040262	9.71E-29	2.56E-27	UP	Vitvi001654	-1.584537008	0.0000567	0.00021657	DOWN
Vitvi007965	1.594764881	0.0000335	0.000132387	UP	Vitvi015427	-1.584639361	0.00000159	0.0000076	DOWN
Vitvi023837	1.594353757	4.15E-09	2.68E-08	UP	Vitvi020005	-1.584753062	0.000000049	0.00000028	DOWN
Vitvi019901	1.594250157	7.86E-35	2.85E-33	UP	Vitvi018678	-1.585263763	4.13E-71	7.37E-69	DOWN
Vitvi031620	1.59210191	2.17E-21	3.76E-20	UP	Vitvi000052	-1.585360312	1.23E-08	7.48E-08	DOWN
Vitvi031519	1.589990856	6.78E-31	2.04E-29	UP	Vitvi000280	-1.586922014	9.15E-13	8.37E-12	DOWN
Vitvi029319	1.588075426	1.44E-47	9.88E-46	UP	Vitvi031129	-1.587295055	0.003764199	0.01043516	DOWN
Vitvi028926	1.588061514	7.82E-27	1.87E-25	UP	Vitvi029078	-1.587489363	0.002469547	0.007104962	DOWN
Vitvi032251	1.586051327	1.56E-24	3.29E-23	UP	Vitvi011275	-1.587696566	0.000279405	0.000959148	DOWN
Vitvi002058	1.585580546	3.2E-21	5.49E-20	UP	Vitvi033466	-1.588614956	0.004541112	0.012362829	DOWN
Vitvi003879	1.585522199	7.11E-55	6.71E-53	UP	Vitvi018840	-1.589576193	0.00000864	0.0000373	DOWN
Vitvi020023	1.585114651	1.44E-23	2.82E-22	UP	Vitvi005717	-1.589905208	0.000000265	0.00000139	DOWN
Vitvi032244	1.5806228	7.43E-08	0.000000416	UP	Vitvi029861	-1.59021361	0.000000084	0.000000467	DOWN
Vitvi009715	1.579418026	6.19E-16	7.19E-15	UP	Vitvi014507	-1.590782597	0.011872795	0.029334656	DOWN
Vitvi033177	1.578725319	3.11E-11	2.45E-10	UP	Vitvi004383	-1.590969089	2.31E-17	3.01E-16	DOWN
Vitvi036820	1.578537899	0.00000194	0.00000914	UP	Vitvi011016	-1.591922044	1.97E-09	1.31E-08	DOWN
Vitvi011410	1.578318367	0.000128697	0.000465471	UP	Vitvi009560	-1.592456018	3.25E-11	2.55E-10	DOWN
Vitvi018387	1.578167537	0.007914805	0.020391363	UP	Vitvi023430	-1.592762411	2.2E-15	2.45E-14	DOWN
Vitvi031082	1.577286117	3.07E-66	4.42E-64	UP	Vitvi031155	-1.592806993	6.09E-14	6.16E-13	DOWN
Vitvi013770	1.576951196	4.71E-17	5.98E-16	UP	Vitvi025147	-1.593187816	0.010433998	0.026184169	DOWN
Vitvi004361	1.576196444	1.27E-30	3.74E-29	UP	Vitvi018291	-1.593230078	5.51E-31	1.67E-29	DOWN
Vitvi022019	1.575859924	3.19E-16	3.81E-15	UP	Vitvi031967	-1.593230777	6.48E-35	2.37E-33	DOWN
Vitvi026893	1.575299491	1.13E-31	3.56E-30	UP	Vitvi025539	-1.594093459	0.0000302	0.000120239	DOWN
Vitvi014800	1.574286045	0.0000731	0.000274247	UP	Vitvi029001	-1.595986371	0.000000582	0.00000294	DOWN
Vitvi014284	1.573873135	9.04E-12	7.5E-11	UP	Vitvi023310	-1.596941329	3.1E-13	2.92E-12	DOWN
Vitvi031766	1.573336775	2.74E-109	1.39E-106	UP	Vitvi022979	-1.597246693	1.63E-26	3.85E-25	DOWN
Vitvi035058	1.570954895	3.45E-19	5.12E-18	UP	Vitvi006866	-1.597422859	0.000936438	0.002938502	DOWN
Vitvi017656	1.567239733	8.58E-10	5.94E-09	UP	Vitvi021230	-1.597798786	7.65E-32	8.95E-29	DOWN
Vitvi032982	1.56697649	5.2E-29	1.39E-27	UP	Vitvi004202	-1.597929723	1.87E-18	2.63E-17	DOWN
Vitvi008602	1.56668524	0.016781108	0.039782665	UP	Vitvi018267	-1.598482974	0.011635509	0.028814978	DOWN
Vitvi025211	1.566670216	7.73E-21	1.29E-19	UP	Vitvi008183	-1.599122776	0.000000743	0.00000369	DOWN
Vitvi004222	1.566585278	4.17E-44	2.32E-42	UP	Vitvi009015	-1.600372923	9.42E-12	7.82E-11	DOWN
Vitvi019882	1.565031687	3.76E-17	4.81E-16	UP	Vitvi008803	-1.600559863	5.67E-08	0.000000321	DOWN
Vitvi022119	1.564588728	8.33E-18	1.11E-16	UP	Vitvi019837	-1.600666669	0.002095903	0.006131641	DOWN
Vitvi033165	1.563853123	0.000000315	0.00000164	UP	Vitvi000690	-1.600838426	4E-30	1.15E-28	DOWN
Vitvi013105	1.562303393	7.62E-13	7.02E-12	UP	Vitvi020591	-1.600862994	0.000000022	0.000000131	DOWN
Vitvi023366	1.561367975	9.51E-17	1.18E-15	UP	Vitvi011436	-1.601179817	1.6E-46	1.01E-44	DOWN
Vitvi009596	1.56007988	1.5E-31	4.69E-30	UP	Vitvi022011	-1.601371621	8.18E-35	2.96E-33	DOWN
Vitvi014302	1.559455379	0.0000175	0.0000722	UP	Vitvi000730	-1.601664754	0.015232299	0.036481364	DOWN
Vitvi015906	1.558532771	0.001621827	0.004868838	UP	Vitvi010904	-1.602907763	4.58E-39	2.03E-37	DOWN
Vitvi005840	1.558135797	8.24E-15	8.82E-14	UP	Vitvi016669	-1.603072918	0.000494825	0.001632167	DOWN
Vitvi027515	1.557952271	0.000303801	0.001036415	UP	Vitvi028809	-1.603718188	0.010332149	0.025960739	DOWN
Vitvi005223	1.557631361	0.0000127	0.0000536	UP	Vitvi023162	-1.605034337	2.55E-12	2.23E-11	DOWN
Vitvi002353	1.556700518	7.94E-33	2.66E-31	UP	Vitvi030229	-1.605531374	0.0000155	0.0000647	DOWN
Vitvi000353	1.555925624	0.000012	0.0000507	UP	Vitvi013679	-1.606176515	1.39E-15	1.57E-14	DOWN
Vitvi004927	1.55478114	1.99E-52	1.68E-50	UP	Vitvi035793	-1.606852706	0.0000168	0.0000695	DOWN

ID	log2FoldChange	pvalue	padj	change	ID	log2FoldChange	pvalue	padj	change
Vitvi007333	1.552114626	4.81E-14	4.9E-13	UP	Vitvi002081	-1.607108721	3.03E-35	1.13E-33	DOWN
Vitvi010269	1.551577004	3.79E-37	1.54E-35	UP	Vitvi021187	-1.607893267	0.0000305	0.00012167	DOWN
Vitvi031092	1.549935437	1.44E-31	4.51E-30	UP	Vitvi021344	-1.610440481	0.003055643	0.008621506	DOWN
Vitvi002414	1.549521634	1.52E-14	1.6E-13	UP	Vitvi017039	-1.61088715	3.02E-11	2.38E-10	DOWN
Vitvi034053	1.546846695	1.07E-35	4.06E-34	UP	Vitvi033213	-1.611537124	6.41E-22	1.15E-20	DOWN
Vitvi032898	1.545872713	7.98E-27	1.91E-25	UP	Vitvi018502	-1.612151863	2.01E-70	3.52E-68	DOWN
Vitvi011786	1.545088687	0.010422836	0.026165943	UP	Vitvi015958	-1.613503656	9.49E-29	2.51E-27	DOWN
Vitvi013635	1.544975039	6.86E-25	1.48E-23	UP	Vitvi036682	-1.614448359	0.000267958	0.000922972	DOWN
Vitvi031278	1.543635079	3.14E-08	0.000000183	UP	Vitvi035866	-1.615816221	0.010664384	0.026676721	DOWN
Vitvi025530	1.543099474	1.71E-11	1.39E-10	UP	Vitvi032779	-1.618373476	6.22E-39	2.74E-37	DOWN
Vitvi029208	1.541982555	0.003183698	0.008944297	UP	Vitvi034648	-1.618708041	3.51E-22	6.4E-21	DOWN
Vitvi027587	1.541974739	0.000012	0.0000508	UP	Vitvi013364	-1.619387183	7.48E-21	1.25E-19	DOWN
Vitvi013293	1.540650982	3.39E-17	4.35E-16	UP	Vitvi004556	-1.619599473	0.000826328	0.002622148	DOWN
Vitvi013171	1.540252593	4.31E-24	8.84E-23	UP	Vitvi025386	-1.619739635	9.17E-16	1.05E-14	DOWN
Vitvi036361	1.538903527	5.62E-31	1.7E-29	UP	Vitvi033935	-1.620771971	3.75E-56	3.75E-54	DOWN
Vitvi002410	1.535551404	1.61E-16	1.96E-15	UP	Vitvi005175	-1.620952304	1.97E-09	1.31E-08	DOWN
Vitvi031805	1.534386931	2.28E-15	2.53E-14	UP	Vitvi019877	-1.621072078	6.04E-08	0.000000341	DOWN
Vitvi001830	1.532716857	1.2E-24	2.55E-23	UP	Vitvi009013	-1.622136866	0.014265651	0.03446698	DOWN
Vitvi033445	1.530747809	9.68E-39	4.22E-37	UP	Vitvi032671	-1.622144455	4.15E-15	4.53E-14	DOWN
Vitvi004372	1.530627798	2.28E-43	1.22E-41	UP	Vitvi018651	-1.622596121	5.88E-69	9.47E-67	DOWN
Vitvi007361	1.527812586	0.000000165	0.000000891	UP	Vitvi018779	-1.623109626	1.09E-25	2.45E-24	DOWN
Vitvi029682	1.527380048	2.43E-20	3.95E-19	UP	Vitvi003645	-1.624731285	1.25E-23	2.48E-22	DOWN
Vitvi008487	1.52704647	2.95E-16	3.54E-15	UP	Vitvi036569	-1.625244675	0.0000192	0.0000789	DOWN
Vitvi010867	1.526325331	7.8E-31	2.32E-29	UP	Vitvi005611	-1.625299549	5.49E-08	0.000000312	DOWN
Vitvi009033	1.525539952	9.39E-26	2.12E-24	UP	Vitvi008321	-1.625378902	9.83E-14	9.73E-13	DOWN
Vitvi010124	1.525293972	7.85E-78	1.68E-75	UP	Vitvi000269	-1.6276447	4.27E-11	3.31E-10	DOWN
Vitvi014590	1.524917961	7.86E-15	8.41E-14	UP	Vitvi007853	-1.628225683	2.03E-26	4.75E-25	DOWN
Vitvi031410	1.524676017	7.21E-26	1.64E-24	UP	Vitvi014524	-1.628711801	6.69E-28	1.7E-26	DOWN
Vitvi030920	1.52396141	0.001588736	0.004775141	UP	Vitvi009835	-1.630463099	6E-17	7.57E-16	DOWN
Vitvi033606	1.523376398	0.00014438	0.000517679	UP	Vitvi033121	-1.631904516	0.002504565	0.007196594	DOWN
Vitvi000312	1.522091757	1.88E-09	1.26E-08	UP	Vitvi003761	-1.633136997	7.9E-76	1.6E-73	DOWN
Vitvi017711	1.521793482	3.74E-16	4.43E-15	UP	Vitvi002008	-1.6334568	5.59E-32	1.8E-30	DOWN
Vitvi020196	1.521638835	3.8E-20	6.09E-19	UP	Vitvi003581	-1.635391173	1.61E-21	2.82E-20	DOWN
Vitvi037104	1.52006818	2.45E-15	2.72E-14	UP	Vitvi024106	-1.635665306	1.85E-15	2.07E-14	DOWN
Vitvi017873	1.519406698	1.23E-08	7.46E-08	UP	Vitvi029470	-1.635912855	0.000000168	0.000000905	DOWN
Vitvi003228	1.518363277	0.000436832	0.001453387	UP	Vitvi018055	-1.637010645	8.63E-78	1.83E-75	DOWN
Vitvi008805	1.517730083	1.53E-08	9.23E-08	UP	Vitvi005616	-1.638271807	1.84E-13	1.78E-12	DOWN
Vitvi009833	1.517416009	1.09E-08	6.65E-08	UP	Vitvi016124	-1.638332643	3.17E-43	1.68E-41	DOWN
Vitvi030765	1.515864854	4.3E-13	4.02E-12	UP	Vitvi035691	-1.641291799	1.69E-66	2.5E-64	DOWN
Vitvi006528	1.514245912	7.69E-42	3.85E-40	UP	Vitvi031377	-1.643650648	1.19E-27	2.99E-26	DOWN
Vitvi001677	1.514221191	4.39E-11	3.4E-10	UP	Vitvi035804	-1.644296677	0.002342896	0.00677615	DOWN
Vitvi007643	1.513573427	1.54E-16	1.88E-15	UP	Vitvi018471	-1.644826712	1.91E-29	5.26E-28	DOWN
Vitvi018159	1.513364872	6.65E-25	1.44E-23	UP	Vitvi022074	-1.644891973	2.18E-10	1.58E-09	DOWN
Vitvi005033	1.512218193	4.11E-11	3.19E-10	UP	Vitvi033289	-1.645734018	7.51E-12	6.28E-11	DOWN
Vitvi030746	1.511074562	3.91E-20	6.26E-19	UP	Vitvi026873	-1.645823249	4.37E-26	1E-24	DOWN
Vitvi037284	1.510839593	0.001030703	0.003211972	UP	Vitvi021630	-1.647441303	7.71E-23	1.46E-21	DOWN
Vitvi019972	1.510818698	1.58E-17	2.08E-16	UP	Vitvi028638	-1.647783117	0.00744748	0.01930244	DOWN
Vitvi018505	1.510766846	0.0000251	0.000101406	UP	Vitvi031163	-1.649507532	5.73E-14	5.81E-13	DOWN
Vitvi013013	1.509988738	1.54E-24	3.25E-23	UP	Vitvi026664	-1.649548532	2.46E-19	3.69E-18	DOWN
Vitvi006580	1.508970136	1.22E-19	1.89E-18	UP	Vitvi015610	-1.650017827	8.84E-09	5.47E-08	DOWN
Vitvi000474	1.508114083	0.0000325	0.000128574	UP	Vitvi019568	-1.650089878	1.14E-08	6.99E-08	DOWN
Vitvi015225	1.504165006	6.12E-19	8.9E-18	UP	Vitvi021682	-1.651609532	1.3E-35	4.89E-34	DOWN
Vitvi007043	1.504023908	3.27E-16	3.91E-15	UP	Vitvi013752	-1.652361667	0.002891523	0.0081983	DOWN
Vitvi019395	1.503555772	5.46E-14	5.54E-13	UP	Vitvi036490	-1.653335588	8.8E-44	4.79E-42	DOWN
Vitvi000580	1.503342182	0.0000338	0.000133324	UP	Vitvi029117	-1.654305938	4.99E-44	2.76E-42	DOWN
Vitvi037354	1.503265626	0.001253768	0.003844005	UP	Vitvi027067	-1.654936561	2.74E-11	2.17E-10	DOWN
Vitvi032892	1.502416602	0.000000051	0.000000291	UP	Vitvi015054	-1.655176972	6.63E-10	4.62E-09	DOWN

ID	log2FoldChange	pvalue	padj	change	ID	log2FoldChange	pvalue	padj	change
Vitvi024860	1.501977283	1.66E-18	2.35E-17	UP	Vitvi033230	-1.656247393	0.0000103	0.0000438	DOWN
Vitvi025612	1.500768835	0.0000112	0.0000473	UP	Vitvi010466	-1.657031292	1.91E-47	1.3E-45	DOWN
Vitvi035705	1.500354122	9.63E-20	1.49E-18	UP	Vitvi005599	-1.65854185	1.13E-25	2.54E-24	DOWN
Vitvi023981	1.499262922	0.001925372	0.005670313	UP	Vitvi031788	-1.659068556	0.011906205	0.029402899	DOWN
Vitvi030518	1.498332647	0.020450831	0.047204236	UP	Vitvi030133	-1.659843087	0.01056432	0.026458997	DOWN
Vitvi036540	1.498220467	1E-38	4.36E-37	UP	Vitvi008834	-1.66021234	0.00000155	0.0000074	DOWN
Vitvi016444	1.496162277	0.0000539	0.000206743	UP	Vitvi001856	-1.660608548	0.012638101	0.030978494	DOWN
Vitvi014467	1.495022692	0.008349122	0.021423367	UP	Vitvi022842	-1.661350642	0.005685118	0.015106766	DOWN
Vitvi017159	1.494485218	1.39E-32	4.6E-31	UP	Vitvi003545	-1.662677109	1.18E-13	1.16E-12	DOWN
Vitvi018048	1.493532642	4.15E-21	7.09E-20	UP	Vitvi015019	-1.663891339	6.33E-19	9.21E-18	DOWN
Vitvi013121	1.492584712	0.000102131	0.000375949	UP	Vitvi022309	-1.66454691	1.46E-64	1.92E-62	DOWN
Vitvi031458	1.491631652	1.06E-23	2.11E-22	UP	Vitvi007902	-1.665784689	0.003339123	0.009344759	DOWN
Vitvi018308	1.49009682	0.009502024	0.024064479	UP	Vitvi010467	-1.665786418	8.2E-14	8.22E-13	DOWN
Vitvi027457	1.489695303	3.56E-10	2.54E-09	UP	Vitvi033049	-1.666542359	1.89E-25	4.19E-24	DOWN
Vitvi025433	1.489134638	6.14E-30	1.75E-28	UP	Vitvi000300	-1.666855694	2.15E-56	2.19E-54	DOWN
Vitvi016636	1.488982174	0.00000025	0.00000132	UP	Vitvi036459	-1.667047448	0.0001185	0.000430815	DOWN
Vitvi019128	1.488424649	3.73E-21	6.4E-20	UP	Vitvi002361	-1.66836668	1.66E-37	6.87E-36	DOWN
Vitvi018169	1.486807454	0.000116233	0.000423439	UP	Vitvi031992	-1.668431842	1.94E-09	1.29E-08	DOWN
Vitvi029681	1.486552985	0.0000115	0.0000488	UP	Vitvi020010	-1.668593292	5.76E-16	6.7E-15	DOWN
Vitvi036406	1.486004092	1.12E-11	9.2E-11	UP	Vitvi023154	-1.669052223	9.47E-09	5.84E-08	DOWN
Vitvi021320	1.485173316	7.11E-11	5.39E-10	UP	Vitvi017033	-1.66974563	5.4E-20	8.56E-19	DOWN
Vitvi021868	1.485041618	4.32E-28	1.11E-26	UP	Vitvi000745	-1.670535135	3.76E-12	3.25E-11	DOWN
Vitvi001642	1.484646114	0.000000229	0.00000121	UP	Vitvi036531	-1.673330609	8.2E-09	0.000000051	DOWN
Vitvi004317	1.484377131	7.19E-18	9.66E-17	UP	Vitvi018717	-1.673768549	1.15E-30	3.41E-29	DOWN
Vitvi001656	1.483312641	1.12E-17	1.49E-16	UP	Vitvi020479	-1.674077972	1.63E-09	1.09E-08	DOWN
Vitvi011187	1.483045122	2.6E-27	6.36E-26	UP	Vitvi015226	-1.674091348	1.15E-20	1.91E-19	DOWN
Vitvi000776	1.482684331	2.88E-17	3.72E-16	UP	Vitvi021752	-1.674493897	1.34E-09	9.1E-09	DOWN
Vitvi009884	1.4817824	8.27E-53	7.19E-51	UP	Vitvi030317	-1.674858478	0.0000408	0.000159396	DOWN
Vitvi021262	1.481761693	8.53E-13	7.83E-12	UP	Vitvi001174	-1.679434054	0.008268814	0.021233394	DOWN
Vitvi011783	1.481562111	5.5E-16	6.41E-15	UP	Vitvi006820	-1.679465317	2.46E-43	1.31E-41	DOWN
Vitvi010265	1.478904678	0.000325042	0.00110387	UP	Vitvi000664	-1.679949532	6.79E-11	5.16E-10	DOWN
Vitvi006620	1.478638737	0.0000252	0.000101816	UP	Vitvi035101	-1.679985956	2.62E-17	3.4E-16	DOWN
Vitvi015256	1.476861166	3.07E-12	2.67E-11	UP	Vitvi006014	-1.680629096	9.19E-51	7.23E-49	DOWN
Vitvi023368	1.476171117	0.007686472	0.019863565	UP	Vitvi007309	-1.680757608	1.1E-14	1.16E-13	DOWN
Vitvi016081	1.474179389	1.27E-24	2.68E-23	UP	Vitvi007277	-1.682122963	5.22E-36	2.01E-34	DOWN
Vitvi036370	1.472648305	0.000141688	0.000508834	UP	Vitvi011294	-1.682329573	2.15E-10	1.56E-09	DOWN
Vitvi000102	1.472495708	6.45E-70	1.1E-67	UP	Vitvi033742	-1.682704832	2.73E-11	2.17E-10	DOWN
Vitvi019789	1.472302172	1.06E-12	9.6E-12	UP	Vitvi004471	-1.682867923	0.009016276	0.022987148	DOWN
Vitvi016296	1.471137269	1.92E-27	4.74E-26	UP	Vitvi016353	-1.683480442	0.000000171	0.000000921	DOWN
Vitvi002465	1.470068001	6.39E-15	6.89E-14	UP	Vitvi010846	-1.684847818	0.0000336	0.000132812	DOWN
Vitvi025248	1.470043974	0.021131269	0.048592589	UP	Vitvi014841	-1.685150969	0.000156624	0.000558819	DOWN
Vitvi025956	1.469935341	3.8E-12	3.28E-11	UP	Vitvi000347	-1.687654549	0.000813981	0.002586605	DOWN
Vitvi019761	1.468522739	9.67E-24	1.93E-22	UP	Vitvi000716	-1.68808059	6.29E-14	6.35E-13	DOWN
Vitvi030692	1.468244976	7.44E-11	5.63E-10	UP	Vitvi006834	-1.691761584	0.000000353	0.00000184	DOWN
Vitvi019971	1.468103041	0.000571521	0.001862166	UP	Vitvi021770	-1.692688064	0.000000591	0.00000298	DOWN
Vitvi014475	1.467231006	0.000142691	0.003690779	UP	Vitvi037342	-1.693078971	0.00000352	0.000016	DOWN
Vitvi021862	1.46716268	1.47E-09	9.9E-09	UP	Vitvi013315	-1.69565248	4.21E-31	1.28E-29	DOWN
Vitvi025142	1.465922864	0.0000155	0.0000645	UP	Vitvi017496	-1.69799084	4.43E-14	4.51E-13	DOWN
Vitvi029210	1.464319067	0.012772533	0.031253546	UP	Vitvi033057	-1.699294106	4.71E-23	9.04E-22	DOWN
Vitvi011835	1.463221268	1E-22	1.87E-21	UP	Vitvi002076	-1.699479286	9.49E-10	6.52E-09	DOWN
Vitvi034970	1.462262334	0.00000332	0.0000152	UP	Vitvi000361	-1.699684086	6.5E-12	5.47E-11	DOWN
Vitvi018269	1.461755879	6.14E-12	5.19E-11	UP	Vitvi030807	-1.700048499	0.017618094	0.041505304	DOWN
Vitvi019412	1.460422362	4.12E-14	4.2E-13	UP	Vitvi023088	-1.700596873	0.000000356	0.00000185	DOWN
Vitvi005294	1.459344127	1.05E-52	9.07E-51	UP	Vitvi013542	-1.701971084	0.0000384	0.000150376	DOWN
Vitvi031460	1.457546658	8.14E-08	0.000000454	UP	Vitvi018929	-1.702144929	1.04E-14	1.1E-13	DOWN
Vitvi018005	1.457035286	0.000000176	0.000000943	UP	Vitvi035855	-1.704406047	1.57E-24	3.31E-23	DOWN
Vitvi014250	1.456867383	6.45E-36	2.47E-34	UP	Vitvi003724	-1.70473336	7.89E-31	2.34E-29	DOWN

ID	log2FoldChange	pvalue	padj	change	ID	log2FoldChange	pvalue	padj	change
Vitvi002456	1.456684409	3.7E-46	2.29E-44	UP	Vitvi006428	-1.70614914	7.39E-52	6.1E-50	DOWN
Vitvi010781	1.456503617	0.011548273	0.028630539	UP	Vitvi033167	-1.706151235	7.2E-17	9.03E-16	DOWN
Vitvi006505	1.455619391	8.98E-20	1.4E-18	UP	Vitvi033238	-1.708163234	0.00000258	0.000012	DOWN
Vitvi005435	1.454956875	3.22E-29	8.76E-28	UP	Vitvi032807	-1.708219727	0.00000655	0.0000287	DOWN
Vitvi000595	1.453367316	0.00000928	0.0000398	UP	Vitvi010151	-1.708601809	0.00000457	0.0000205	DOWN
Vitvi024718	1.451062384	0.0000135	0.0000567	UP	Vitvi037383	-1.711442856	8.25E-70	1.39E-67	DOWN
Vitvi009280	1.451020373	1.25E-38	5.44E-37	UP	Vitvi000538	-1.711469047	8.12E-19	1.17E-17	DOWN
Vitvi030924	1.450517869	4.26E-29	1.15E-27	UP	Vitvi007727	-1.713282974	1.22E-21	2.15E-20	DOWN
Vitvi029659	1.449913412	0.004360749	0.011914923	UP	Vitvi030621	-1.713389227	0.000008	0.0000347	DOWN
Vitvi016031	1.449839575	2.01E-18	2.82E-17	UP	Vitvi005793	-1.713417088	3.54E-29	9.58E-28	DOWN
Vitvi016085	1.446961288	1.68E-44	9.65E-43	UP	Vitvi002054	-1.713445971	2.47E-31	7.61E-30	DOWN
Vitvi030854	1.446064285	3.48E-16	4.14E-15	UP	Vitvi019816	-1.714100394	0.000000473	0.00000242	DOWN
Vitvi015762	1.444418893	1.04E-16	1.29E-15	UP	Vitvi006829	-1.715074586	1.45E-11	1.17E-10	DOWN
Vitvi000337	1.444378509	0.001719578	0.005134214	UP	Vitvi033059	-1.719565702	2.59E-50	1.97E-48	DOWN
Vitvi002311	1.443245994	8.53E-18	1.14E-16	UP	Vitvi020953	-1.719697576	1.1E-11	9.08E-11	DOWN
Vitvi031057	1.443199694	2.5E-10	1.8E-09	UP	Vitvi005532	-1.719711146	4.75E-11	3.67E-10	DOWN
Vitvi020386	1.442471496	1.2E-14	1.27E-13	UP	Vitvi019257	-1.719929948	0.009571014	0.024207004	DOWN
Vitvi031046	1.441748765	3.66E-13	3.44E-12	UP	Vitvi004738	-1.720426182	0.003453781	0.009637751	DOWN
Vitvi000207	1.441482978	1.13E-54	1.06E-52	UP	Vitvi016463	-1.720529319	0.000000326	0.0000017	DOWN
Vitvi034614	1.441468008	0.020050358	0.046464674	UP	Vitvi027651	-1.720985598	2.48E-30	7.23E-29	DOWN
Vitvi020816	1.441200712	9.14E-20	1.42E-18	UP	Vitvi036300	-1.723877507	8.88E-34	3.09E-32	DOWN
Vitvi011506	1.440832598	0.000000299	0.00000156	UP	Vitvi025917	-1.724657837	0.018713092	0.043770189	DOWN
Vitvi035679	1.438811818	3.97E-12	3.42E-11	UP	Vitvi032254	-1.725765194	0.001204103	0.003700113	DOWN
Vitvi033812	1.437482268	2.05E-22	3.78E-21	UP	Vitvi004485	-1.727549058	3.94E-16	4.65E-15	DOWN
Vitvi035119	1.437038649	0.0000024	0.0000112	UP	Vitvi027601	-1.727883873	1.63E-25	3.64E-24	DOWN
Vitvi003503	1.43602753	0.00000835	0.0000361	UP	Vitvi010615	-1.728752298	2.52E-44	1.43E-42	DOWN
Vitvi009836	1.435869896	0.00000414	0.0000186	UP	Vitvi011499	-1.729607476	0.000355716	0.001200414	DOWN
Vitvi019920	1.435583038	3.73E-15	4.1E-14	UP	Vitvi011336	-1.729663297	1.15E-13	1.13E-12	DOWN
Vitvi023400	1.434960331	0.000228666	0.000796822	UP	Vitvi029022	-1.731484122	1.55E-10	1.14E-09	DOWN
Vitvi030875	1.434378761	0.000531375	0.001742259	UP	Vitvi021717	-1.731726213	0.000068	0.000256459	DOWN
Vitvi019667	1.434000652	0.006381631	0.016775213	UP	Vitvi037408	-1.731774588	1.22E-08	7.41E-08	DOWN
Vitvi031691	1.433294006	2.05E-53	1.83E-51	UP	Vitvi016097	-1.731847081	1.06E-08	6.47E-08	DOWN
Vitvi013310	1.433254087	1.5E-32	4.96E-31	UP	Vitvi016275	-1.732672151	0.000525321	0.0017238	DOWN
Vitvi023060	1.432396822	6.72E-65	9.1E-63	UP	Vitvi033347	-1.734254275	2.49E-18	3.46E-17	DOWN
Vitvi002656	1.432179849	5.42E-11	4.17E-10	UP	Vitvi020796	-1.735070298	3.27E-10	2.34E-09	DOWN
Vitvi036189	1.431972206	7.66E-09	4.78E-08	UP	Vitvi022569	-1.735320168	0.000779162	0.002484122	DOWN
Vitvi003541	1.431151286	2.27E-13	2.17E-12	UP	Vitvi013562	-1.735932921	1.52E-30	4.47E-29	DOWN
Vitvi010014	1.430645035	1.48E-15	1.67E-14	UP	Vitvi030733	-1.73852779	0.000528031	0.001732133	DOWN
Vitvi026610	1.430236965	0.003131393	0.008808317	UP	Vitvi030627	-1.741265404	0.000000454	0.00000233	DOWN
Vitvi007864	1.428620066	1.82E-14	1.91E-13	UP	Vitvi015255	-1.741566726	0.000932204	0.002926572	DOWN
Vitvi035531	1.427899809	0.000273612	0.000940851	UP	Vitvi008315	-1.741780347	9.23E-60	1.07E-57	DOWN
Vitvi003560	1.427637947	6.11E-22	1.1E-20	UP	Vitvi005413	-1.743230652	0.00000993	0.0000424	DOWN
Vitvi026491	1.425542781	1.28E-17	1.69E-16	UP	Vitvi030598	-1.743638958	4.86E-08	0.000000278	DOWN
Vitvi027408	1.425318444	7.22E-17	9.05E-16	UP	Vitvi002871	-1.743954334	1.1E-24	2.34E-23	DOWN
Vitvi016023	1.424871354	4.37E-12	3.74E-11	UP	Vitvi001363	-1.744287038	0.009784157	0.024699951	DOWN
Vitvi031756	1.423933794	0.000000483	0.00000247	UP	Vitvi016324	-1.748133031	1.9E-13	1.83E-12	DOWN
Vitvi023365	1.422905811	4.82E-09	3.08E-08	UP	Vitvi030300	-1.749561351	2.06E-27	5.08E-26	DOWN
Vitvi027105	1.42269822	0.001869087	0.005525381	UP	Vitvi036036	-1.751056545	0.00000023	0.00000122	DOWN
Vitvi015785	1.422354839	2.05E-12	1.82E-11	UP	Vitvi019664	-1.752640656	3.7E-71	6.65E-69	DOWN
Vitvi025130	1.421439039	3.33E-10	2.38E-09	UP	Vitvi025146	-1.753612204	7.22E-32	2.31E-30	DOWN
Vitvi030638	1.419855915	0.000104162	0.000382938	UP	Vitvi007231	-1.755145244	0.000428447	0.00142736	DOWN
Vitvi004935	1.419361387	2.78E-20	4.5E-19	UP	Vitvi010443	-1.756272585	2.29E-13	2.19E-12	DOWN
Vitvi012135	1.419055923	6.97E-08	0.000000391	UP	Vitvi017121	-1.756295587	0.000120534	0.00043782	DOWN
Vitvi029175	1.418844799	1.14E-44	6.62E-43	UP	Vitvi036217	-1.756427449	1.2E-21	2.11E-20	DOWN
Vitvi019530	1.414554685	2.83E-19	4.24E-18	UP	Vitvi004355	-1.756768859	5.93E-35	2.18E-33	DOWN
Vitvi003755	1.413797904	0.00000252	0.0000117	UP	Vitvi025643	-1.75699087	2.81E-19	4.21E-18	DOWN
Vitvi002243	1.411866014	8.27E-11	6.23E-10	UP	Vitvi010070	-1.759368654	7.18E-35	2.61E-33	DOWN

ID	log2FoldChange	pvalue	padj	change	ID	log2FoldChange	pvalue	padj	change
Vitvi031537	1.409816986	1.41E-11	1.15E-10	UP	Vitvi034616	-1.759393396	0.009906509	0.024977781	DOWN
Vitvi023241	1.40949382	4.36E-18	5.96E-17	UP	Vitvi023091	-1.762082819	3.56E-24	7.34E-23	DOWN
Vitvi003967	1.408707191	4.2E-36	1.63E-34	UP	Vitvi030780	-1.764257746	2.01E-10	1.47E-09	DOWN
Vitvi009430	1.406653489	4E-13	3.75E-12	UP	Vitvi012439	-1.765791671	5.46E-15	5.93E-14	DOWN
Vitvi022437	1.40609976	0.000729615	0.002340483	UP	Vitvi018638	-1.766165615	2.67E-54	2.43E-52	DOWN
Vitvi032607	1.40573683	1.87E-10	1.37E-09	UP	Vitvi010931	-1.766195236	1.37E-09	9.28E-09	DOWN
Vitvi018184	1.405119913	4.69E-31	1.42E-29	UP	Vitvi020285	-1.770364753	1.22E-10	9.03E-10	DOWN
Vitvi015189	1.404679128	0.00000043	0.00000221	UP	Vitvi014659	-1.770474573	0.002596971	0.007436863	DOWN
Vitvi006196	1.400661618	8.96E-31	2.66E-29	UP	Vitvi032957	-1.773097058	6.12E-22	1.1E-20	DOWN
Vitvi002756	1.399487287	3.48E-17	4.47E-16	UP	Vitvi020081	-1.775231857	0.013897797	0.03370249	DOWN
Vitvi021480	1.399165666	9.16E-27	2.18E-25	UP	Vitvi001863	-1.77531679	3.23E-30	9.36E-29	DOWN
Vitvi010051	1.398287287	0.006967644	0.018165419	UP	Vitvi004127	-1.77594082	1.02E-48	7.24E-47	DOWN
Vitvi013627	1.395976824	9.52E-14	9.43E-13	UP	Vitvi007644	-1.776800021	1.31E-37	5.47E-36	DOWN
Vitvi004388	1.395932602	2.13E-08	0.000000126	UP	Vitvi001183	-1.778653138	2.87E-14	2.96E-13	DOWN
Vitvi005761	1.395797249	0.0000179	0.0000739	UP	Vitvi005772	-1.779635903	1.09E-27	2.74E-26	DOWN
Vitvi002155	1.393066985	2.04E-31	6.31E-30	UP	Vitvi007192	-1.78001001	0.004135016	0.011351915	DOWN
Vitvi009223	1.38974603	8.09E-24	1.63E-22	UP	Vitvi029610	-1.780780598	0.000117176	0.00042646	DOWN
Vitvi031055	1.38933313	1.94E-45	1.17E-43	UP	Vitvi035794	-1.780894107	0.0000382	0.000149586	DOWN
Vitvi011568	1.388260233	5.87E-24	1.19E-22	UP	Vitvi003787	-1.780991195	1.47E-13	1.44E-12	DOWN
Vitvi020043	1.386033677	2.02E-08	0.00000012	UP	Vitvi029051	-1.781923347	1.46E-09	9.88E-09	DOWN
Vitvi011125	1.383363224	5.09E-12	4.33E-11	UP	Vitvi025029	-1.782062916	0.00000318	0.0000146	DOWN
Vitvi022321	1.383069633	1.61E-08	9.66E-08	UP	Vitvi033136	-1.784009243	1.34E-38	5.79E-37	DOWN
Vitvi005878	1.382769641	4.45E-14	4.54E-13	UP	Vitvi005295	-1.787852975	1.13E-09	7.68E-09	DOWN
Vitvi000542	1.382322064	1.53E-28	4E-27	UP	Vitvi000868	-1.790563999	7.9E-11	5.96E-10	DOWN
Vitvi006904	1.381712714	5.86E-08	0.000000332	UP	Vitvi000953	-1.791542381	4.96E-34	1.75E-32	DOWN
Vitvi007132	1.381099398	0.000106509	0.000391072	UP	Vitvi011669	-1.792969028	1.33E-10	9.84E-10	DOWN
Vitvi033506	1.380285674	0.000000564	0.00000285	UP	Vitvi009491	-1.795637929	2.7E-17	3.5E-16	DOWN
Vitvi030309	1.37878886	2.9E-13	2.75E-12	UP	Vitvi021068	-1.796094039	0.00000509	0.0000227	DOWN
Vitvi032125	1.378407571	5.23E-09	3.34E-08	UP	Vitvi002661	-1.797416458	5.15E-35	1.9E-33	DOWN
Vitvi010674	1.378262906	4.44E-08	0.000000255	UP	Vitvi020114	-1.797731108	8.71E-51	6.88E-49	DOWN
Vitvi014472	1.377187037	3.93E-13	3.69E-12	UP	Vitvi034017	-1.79909682	1.24E-91	4.13E-89	DOWN
Vitvi009188	1.376706043	0.000000135	0.000000738	UP	Vitvi014519	-1.799730893	6.47E-09	4.08E-08	DOWN
Vitvi023270	1.376380757	1.38E-13	1.35E-12	UP	Vitvi017585	-1.799837412	3.54E-49	2.59E-47	DOWN
Vitvi002596	1.376077208	8.81E-16	1.01E-14	UP	Vitvi026001	-1.800551253	8.64E-89	2.7E-86	DOWN
Vitvi015563	1.375233754	0.000870071	0.002746795	UP	Vitvi032689	-1.800705436	1.68E-48	1.18E-46	DOWN
Vitvi033178	1.372936221	0.00271532	0.00774705	UP	Vitvi008887	-1.801799844	6.5E-10	4.53E-09	DOWN
Vitvi022370	1.37206456	0.001080024	0.003352812	UP	Vitvi027427	-1.802179194	0.0000832	0.00030984	DOWN
Vitvi019716	1.37159185	6.97E-31	2.09E-29	UP	Vitvi002167	-1.80305924	0.003720321	0.010321972	DOWN
Vitvi002284	1.369494072	0.004653789	0.012632335	UP	Vitvi007628	-1.803298913	0.0000694	0.000261388	DOWN
Vitvi022298	1.367939217	1.08E-52	9.29E-51	UP	Vitvi002250	-1.804467944	1.87E-29	5.16E-28	DOWN
Vitvi013438	1.364744027	0.000349801	0.001181435	UP	Vitvi000006	-1.805965953	1.44E-12	1.29E-11	DOWN
Vitvi031413	1.363865932	2.29E-29	6.3E-28	UP	Vitvi011270	-1.806177079	5.08E-13	4.72E-12	DOWN
Vitvi020516	1.362073141	0.000188912	0.00066583	UP	Vitvi025071	-1.806505338	1.31E-56	1.34E-54	DOWN
Vitvi000127	1.362024597	2.64E-16	3.18E-15	UP	Vitvi034690	-1.80705742	4.4E-13	4.1E-12	DOWN
Vitvi012494	1.360807425	0.0000127	0.0000532	UP	Vitvi030065	-1.80991376	0.000576687	0.001877791	DOWN
Vitvi011572	1.36014618	1.3E-21	2.29E-20	UP	Vitvi019718	-1.809941892	6.16E-41	2.95E-39	DOWN
Vitvi035172	1.359867223	3.12E-09	2.04E-08	UP	Vitvi023467	-1.810836232	2.01E-13	1.92E-12	DOWN
Vitvi010148	1.359648854	0.0000534	0.000204756	UP	Vitvi008862	-1.811206942	1.61E-11	1.3E-10	DOWN
Vitvi027481	1.359545951	0.003481105	0.00971133	UP	Vitvi030254	-1.813318679	5.23E-18	7.1E-17	DOWN
Vitvi005222	1.359156068	1.07E-17	1.42E-16	UP	Vitvi009213	-1.814847799	0.00000311	0.0000143	DOWN
Vitvi009515	1.357564978	0.001745261	0.005200961	UP	Vitvi018790	-1.816228819	4.25E-27	1.03E-25	DOWN
Vitvi030741	1.355387382	0.005799971	0.015389813	UP	Vitvi027625	-1.816955202	1.69E-25	3.76E-24	DOWN
Vitvi009778	1.353826385	5.89E-28	1.5E-26	UP	Vitvi028871	-1.817195665	1.47E-10	1.08E-09	DOWN
Vitvi023282	1.35154659	2.05E-21	3.56E-20	UP	Vitvi032207	-1.818606339	2.42E-09	1.59E-08	DOWN
Vitvi036316	1.349881019	1.19E-11	9.74E-11	UP	Vitvi005669	-1.820134161	1.5E-10	1.1E-09	DOWN
Vitvi027482	1.349177044	2.05E-20	3.37E-19	UP	Vitvi004386	-1.820193732	1.47E-14	1.54E-13	DOWN
Vitvi000561	1.346542032	2.94E-17	3.8E-16	UP	Vitvi003725	-1.820271631	1.1E-16	1.36E-15	DOWN

ID	log2FoldChange	pvalue	padj	change	ID	log2FoldChange	pvalue	padj	change
Vitvi028893	1.345779188	2.02E-12	1.79E-11	UP	Vitvi028274	-1.820385099	5.82E-23	1.11E-21	DOWN
Vitvi009699	1.345526901	2.06E-37	8.43E-36	UP	Vitvi019839	-1.821503848	1.64E-34	5.84E-33	DOWN
Vitvi025346	1.34528088	6.47E-10	4.51E-09	UP	Vitvi036521	-1.822520997	9.31E-28	2.35E-26	DOWN
Vitvi003979	1.344773097	8.94E-39	3.92E-37	UP	Vitvi012343	-1.823668627	0.002461841	0.007083854	DOWN
Vitvi027337	1.344457592	0.002253759	0.006544698	UP	Vitvi036519	-1.826640964	0.00000253	0.0000117	DOWN
Vitvi035867	1.344298342	0.012623205	0.030951538	UP	Vitvi029073	-1.82765345	0.000000126	0.000000691	DOWN
Vitvi025023	1.341763801	0.000148422	0.003809054	UP	Vitvi023554	-1.827785275	0.001926175	0.005671858	DOWN
Vitvi033898	1.340343986	7.99E-16	9.21E-15	UP	Vitvi000744	-1.827790971	4.39E-24	8.98E-23	DOWN
Vitvi031118	1.33904832	2.74E-31	8.43E-30	UP	Vitvi000516	-1.829075859	2.47E-33	8.44E-32	DOWN
Vitvi001532	1.338727577	9.44E-23	1.77E-21	UP	Vitvi031384	-1.829606107	0.011821389	0.029218307	DOWN
Vitvi029667	1.337866035	0.0000631	0.000239328	UP	Vitvi001612	-1.831813346	1.1E-09	7.52E-09	DOWN
Vitvi001381	1.337762105	0.000353195	0.001192304	UP	Vitvi006832	-1.833663591	9.77E-08	0.00000054	DOWN
Vitvi026112	1.337345887	0.004288785	0.011732497	UP	Vitvi036448	-1.836969181	1.22E-25	2.73E-24	DOWN
Vitvi020854	1.336952992	5.83E-25	1.27E-23	UP	Vitvi008108	-1.837409371	8.15E-20	1.28E-18	DOWN
Vitvi013524	1.334976093	2.61E-14	2.7E-13	UP	Vitvi018399	-1.837489894	0.001220517	0.003748284	DOWN
Vitvi031727	1.332810644	0.000000171	0.00000092	UP	Vitvi010945	-1.837974449	1.52E-14	1.6E-13	DOWN
Vitvi035383	1.332432514	0.000764846	0.002442117	UP	Vitvi000864	-1.839572918	4.94E-57	5.17E-55	DOWN
Vitvi013209	1.332420376	0.0000197	0.0000807	UP	Vitvi006312	-1.839602838	6.05E-32	1.94E-30	DOWN
Vitvi015338	1.332329137	0.015965657	0.038049328	UP	Vitvi021530	-1.842455932	7.71E-48	5.31E-46	DOWN
Vitvi016020	1.331554309	3.35E-16	3.99E-15	UP	Vitvi024159	-1.842592767	0.0000207	0.0000846	DOWN
Vitvi030947	1.331553864	0.000749007	0.002396635	UP	Vitvi003452	-1.842704799	3.55E-17	4.56E-16	DOWN
Vitvi004280	1.331423853	2.34E-12	2.06E-11	UP	Vitvi033889	-1.844052489	5.16E-47	3.42E-45	DOWN
Vitvi030764	1.330969234	0.017319389	0.040891716	UP	Vitvi015983	-1.845746388	1.09E-35	4.14E-34	DOWN
Vitvi014898	1.329164165	1.01E-12	9.23E-12	UP	Vitvi001900	-1.846394522	1.67E-51	1.34E-49	DOWN
Vitvi017071	1.328720945	1.96E-12	1.74E-11	UP	Vitvi000480	-1.846444902	9.49E-41	4.49E-39	DOWN
Vitvi008343	1.327849893	8.28E-53	7.19E-51	UP	Vitvi015600	-1.846543497	0.000930844	0.002922754	DOWN
Vitvi018179	1.327330593	2.87E-24	5.96E-23	UP	Vitvi026246	-1.849069198	0.00000495	0.0000221	DOWN
Vitvi006550	1.325909836	8.4E-18	1.12E-16	UP	Vitvi030971	-1.850343169	0.0000266	0.000106896	DOWN
Vitvi004540	1.325735626	2.42E-08	0.000000143	UP	Vitvi010083	-1.850749966	0.000623161	0.002018764	DOWN
Vitvi017515	1.325383015	2.79E-19	4.18E-18	UP	Vitvi027343	-1.852053277	0.007913957	0.020391363	DOWN
Vitvi004201	1.324476976	0.00000415	0.0000187	UP	Vitvi002576	-1.853782947	2.41E-41	1.17E-39	DOWN
Vitvi024631	1.323258621	2.93E-21	5.04E-20	UP	Vitvi007884	-1.854717011	0.007701323	0.019899411	DOWN
Vitvi000753	1.31985798	0.000000907	0.00000445	UP	Vitvi012629	-1.855496714	1.78E-86	5.33E-84	DOWN
Vitvi004374	1.319836419	6.82E-10	4.75E-09	UP	Vitvi014803	-1.85605864	0.000000719	0.00000358	DOWN
Vitvi029476	1.319748418	1.24E-08	7.54E-08	UP	Vitvi027377	-1.856700515	2.01E-32	6.6E-31	DOWN
Vitvi031945	1.319523108	0.000366319	0.001234557	UP	Vitvi001465	-1.856813343	3.95E-49	2.85E-47	DOWN
Vitvi013100	1.318359105	0.000173964	0.000617323	UP	Vitvi033155	-1.857791614	0.0000369	0.000144992	DOWN
Vitvi010978	1.317394187	0.000000197	0.00000105	UP	Vitvi022742	-1.858056052	6.03E-74	1.14E-71	DOWN
Vitvi015795	1.317196354	1.88E-38	8.09E-37	UP	Vitvi036450	-1.858903749	0.00587407	0.015566094	DOWN
Vitvi032700	1.316966599	3.81E-39	1.69E-37	UP	Vitvi008037	-1.861471283	2.47E-10	1.78E-09	DOWN
Vitvi022974	1.316539491	0.000219331	0.000765735	UP	Vitvi010145	-1.862735264	0.0000134	0.0000562	DOWN
Vitvi033937	1.315067367	1.34E-12	1.21E-11	UP	Vitvi023208	-1.864103513	0.00000726	0.0000317	DOWN
Vitvi021232	1.314819237	2.62E-23	5.09E-22	UP	Vitvi026543	-1.864290385	8.81E-11	6.63E-10	DOWN
Vitvi032768	1.313828519	4.22E-27	1.03E-25	UP	Vitvi025517	-1.864465568	3.27E-22	5.97E-21	DOWN
Vitvi033660	1.313513982	2.81E-12	2.45E-11	UP	Vitvi023605	-1.86691463	0.001232811	0.003783958	DOWN
Vitvi033564	1.312612008	0.000000894	0.00000439	UP	Vitvi003722	-1.867377404	0.00000163	0.00000777	DOWN
Vitvi004975	1.311963614	0.000114703	0.000418554	UP	Vitvi014386	-1.87010018	4.07E-08	0.000000235	DOWN
Vitvi027239	1.311130973	8.76E-49	6.27E-47	UP	Vitvi000508	-1.870575868	6.49E-19	9.42E-18	DOWN
Vitvi000858	1.310540012	0.000790255	0.002517118	UP	Vitvi004907	-1.875809103	0.013380118	0.032580642	DOWN
Vitvi024964	1.308955591	0.0000152	0.0000633	UP	Vitvi002893	-1.87588675	1.01E-70	1.79E-68	DOWN
Vitvi006869	1.308241376	1.08E-13	1.06E-12	UP	Vitvi006047	-1.876206537	6.44E-12	5.42E-11	DOWN
Vitvi018611	1.308212528	0.003050359	0.008607793	UP	Vitvi031297	-1.876502274	4.08E-08	0.000000236	DOWN
Vitvi033127	1.307754716	3.66E-49	2.66E-47	UP	Vitvi012422	-1.876545038	0.000384993	0.001291917	DOWN
Vitvi020027	1.307115064	2.22E-14	2.3E-13	UP	Vitvi016218	-1.880979054	9.52E-16	1.09E-14	DOWN
Vitvi002874	1.304228764	2.18E-19	3.29E-18	UP	Vitvi028689	-1.882923946	6.38E-19	9.27E-18	DOWN
Vitvi029809	1.303641613	2.01E-24	4.21E-23	UP	Vitvi028875	-1.883412914	3.22E-41	1.55E-39	DOWN
Vitvi026977	1.300850282	1.65E-49	1.22E-47	UP	Vitvi009416	-1.883922935	0.00000423	0.000019	DOWN

ID	log2FoldChange	pvalue	padj	change	ID	log2FoldChange	pvalue	padj	change
Vitvi037309	1.300568099	0.019924982	0.04620645	UP	Vitvi016360	-1.88669815	8.56E-09	5.31E-08	DOWN
Vitvi025509	1.300160971	7.48E-12	6.26E-11	UP	Vitvi022303	-1.888689257	2.1E-27	5.16E-26	DOWN
Vitvi015494	1.300159272	0.0000269	0.000107967	UP	Vitvi035103	-1.893938807	0.002967357	0.0083969	DOWN
Vitvi031069	1.299993528	1.87E-08	0.000000112	UP	Vitvi027595	-1.896184833	2.28E-14	2.37E-13	DOWN
Vitvi027479	1.298985978	1.61E-19	2.45E-18	UP	Vitvi007566	-1.9000678	0.00350175	0.009766242	DOWN
Vitvi010645	1.297002154	0.017230163	0.040728391	UP	Vitvi031679	-1.902204415	8.26E-08	0.00000046	DOWN
Vitvi037393	1.295972138	4.1E-17	5.23E-16	UP	Vitvi010030	-1.903810839	1.53E-29	4.23E-28	DOWN
Vitvi001063	1.295792914	3.88E-31	1.18E-29	UP	Vitvi006254	-1.904313251	3.81E-66	5.37E-64	DOWN
Vitvi031751	1.295133839	9.15E-20	1.42E-18	UP	Vitvi004331	-1.904335818	1.56E-19	2.38E-18	DOWN
Vitvi030694	1.295045885	1.39E-10	1.02E-09	UP	Vitvi029030	-1.904551991	2.93E-53	2.6E-51	DOWN
Vitvi023526	1.295034959	2.19E-22	4.03E-21	UP	Vitvi011784	-1.904954116	0.00000258	0.000012	DOWN
Vitvi013569	1.295023734	8.81E-09	5.46E-08	UP	Vitvi026270	-1.906155954	0.014383417	0.034706103	DOWN
Vitvi027529	1.294898887	0.020513819	0.047321705	UP	Vitvi035917	-1.907779941	0.000215809	0.000754088	DOWN
Vitvi037401	1.294429509	4.33E-19	6.4E-18	UP	Vitvi031755	-1.908812507	9.35E-13	8.54E-12	DOWN
Vitvi031390	1.293220876	1.31E-10	9.67E-10	UP	Vitvi010823	-1.909494195	1.16E-16	1.43E-15	DOWN
Vitvi028635	1.292311391	0.017301565	0.040854382	UP	Vitvi033821	-1.911470482	1.12E-46	7.2E-45	DOWN
Vitvi003389	1.292045767	1.25E-10	9.25E-10	UP	Vitvi016370	-1.915224722	1.94E-16	2.35E-15	DOWN
Vitvi021643	1.290928693	5.22E-12	4.43E-11	UP	Vitvi033890	-1.916723954	1.7E-14	1.78E-13	DOWN
Vitvi022021	1.289581372	5.08E-09	3.24E-08	UP	Vitvi013116	-1.91705591	2.26E-22	4.15E-21	DOWN
Vitvi015024	1.288927373	0.0000226	0.0000917	UP	Vitvi026762	-1.917135817	0.00000214	0.00001	DOWN
Vitvi031021	1.288378567	1.44E-17	1.9E-16	UP	Vitvi006794	-1.91886662	5.43E-21	9.22E-20	DOWN
Vitvi035174	1.288300411	0.002124849	0.031135236	UP	Vitvi031451	-1.921663211	1.84E-23	3.61E-22	DOWN
Vitvi008863	1.287681449	1.57E-19	2.39E-18	UP	Vitvi009063	-1.921793719	1.91E-23	3.73E-22	DOWN
Vitvi016891	1.285576511	1.4E-29	3.91E-28	UP	Vitvi023873	-1.923236004	0.00000109	0.00000531	DOWN
Vitvi005313	1.285336893	2.79E-11	2.21E-10	UP	Vitvi033788	-1.923724175	0.00000101	0.00000494	DOWN
Vitvi025981	1.284742342	3.58E-24	7.38E-23	UP	Vitvi000058	-1.924942755	0.007220297	0.018751909	DOWN
Vitvi036973	1.282861126	0.002466391	0.03485912	UP	Vitvi002549	-1.925184969	6.08E-09	3.84E-08	DOWN
Vitvi017668	1.282489906	0.00000489	0.0000218	UP	Vitvi015227	-1.925785466	1.2E-51	9.77E-50	DOWN
Vitvi015583	1.28223701	0.0000195	0.00008	UP	Vitvi002256	-1.92625693	0.008011463	0.020627307	DOWN
Vitvi000750	1.28089287	4.33E-20	6.9E-19	UP	Vitvi035699	-1.931422423	0.0000195	0.00008	DOWN
Vitvi031690	1.280558067	1.59E-08	9.58E-08	UP	Vitvi001232	-1.932381216	2.17E-10	1.58E-09	DOWN
Vitvi018320	1.280524204	9.26E-21	1.54E-19	UP	Vitvi002244	-1.935661544	0.003714346	0.010306804	DOWN
Vitvi000316	1.277603066	0.0000124	0.0000522	UP	Vitvi006008	-1.935679062	0.00000986	0.0000421	DOWN
Vitvi009892	1.276817258	1.56E-21	2.73E-20	UP	Vitvi027053	-1.936496653	0.001717168	0.026675375	DOWN
Vitvi004011	1.276707198	6.41E-36	2.46E-34	UP	Vitvi003999	-1.938004218	0.00000677	0.0000296	DOWN
Vitvi012526	1.275411317	6.07E-21	1.02E-19	UP	Vitvi018288	-1.938504014	4.71E-28	1.21E-26	DOWN
Vitvi022002	1.273936697	2.8E-18	3.88E-17	UP	Vitvi028845	-1.938711587	3.22E-23	6.21E-22	DOWN
Vitvi019571	1.273594398	2.38E-38	1.02E-36	UP	Vitvi005467	-1.939071725	9.03E-14	8.99E-13	DOWN
Vitvi025901	1.270194053	2.82E-22	5.17E-21	UP	Vitvi007019	-1.939460416	1.66E-19	2.52E-18	DOWN
Vitvi002441	1.268954639	0.004088614	0.011242442	UP	Vitvi002037	-1.939769893	0.002289206	0.032938295	DOWN
Vitvi013148	1.26770455	4.84E-40	2.23E-38	UP	Vitvi014814	-1.94041097	1.78E-37	7.3E-36	DOWN
Vitvi031670	1.266743348	7.18E-09	4.49E-08	UP	Vitvi007732	-1.940560289	0.001036288	0.003226406	DOWN
Vitvi024097	1.264658013	4.7E-11	3.63E-10	UP	Vitvi012654	-1.941319214	0.00673756	0.017631193	DOWN
Vitvi032781	1.259718987	1.35E-08	8.17E-08	UP	Vitvi011452	-1.941731151	0.0000078	0.0000339	DOWN
Vitvi001112	1.259476655	1.46E-27	3.65E-26	UP	Vitvi010650	-1.941778929	4.16E-45	2.48E-43	DOWN
Vitvi023142	1.258906174	1.3E-16	1.6E-15	UP	Vitvi020251	-1.944086451	2.09E-19	3.16E-18	DOWN
Vitvi015535	1.258858302	5.66E-10	3.97E-09	UP	Vitvi015781	-1.945516398	9.37E-51	7.35E-49	DOWN
Vitvi035775	1.257253241	7.99E-13	7.35E-12	UP	Vitvi003618	-1.94595265	0.00000101	0.00000492	DOWN
Vitvi013229	1.255532079	1.14E-17	1.51E-16	UP	Vitvi013244	-1.946596196	4.35E-57	4.58E-55	DOWN
Vitvi014710	1.254617338	4.34E-12	3.71E-11	UP	Vitvi023421	-1.947138903	0.004895346	0.0132121	DOWN
Vitvi021192	1.254588202	0.00000768	0.0000334	UP	Vitvi014692	-1.94926226	3.27E-12	2.84E-11	DOWN
Vitvi030770	1.254441728	0.000409476	0.001368197	UP	Vitvi010822	-1.949312703	1.58E-13	1.54E-12	DOWN
Vitvi021169	1.254347192	3.17E-15	3.49E-14	UP	Vitvi031309	-1.94973853	0.0000646	0.000244545	DOWN
Vitvi036347	1.252329886	1.6E-27	3.96E-26	UP	Vitvi024876	-1.950049296	7.63E-17	9.55E-16	DOWN
Vitvi023643	1.25189612	0.0000305	0.000121653	UP	Vitvi003232	-1.951316852	0.001489153	0.004508522	DOWN
Vitvi023407	1.251683542	9.73E-17	1.21E-15	UP	Vitvi020951	-1.954296934	0.016190721	0.038540481	DOWN
Vitvi021051	1.251076034	3.19E-30	9.26E-29	UP	Vitvi013061	-1.954717661	0.0000697	0.000262481	DOWN

ID	log2FoldChange	pvalue	padj	change	ID	log2FoldChange	pvalue	padj	change
Vitvi014499	1.250930825	0.00079695	0.002535659	UP	Vitvi011091	-1.956276587	3.87E-52	3.23E-50	DOWN
Vitvi027093	1.250818827	0.0000334	0.000131954	UP	Vitvi016090	-1.961021456	4.22E-13	3.95E-12	DOWN
Vitvi016726	1.249963182	3.78E-29	1.02E-27	UP	Vitvi018657	-1.961118375	0.000000936	0.00000459	DOWN
Vitvi006041	1.249629655	4.86E-30	1.4E-28	UP	Vitvi033433	-1.962766842	0.000000137	0.000000747	DOWN
Vitvi023453	1.24881664	0.001292932	0.003957509	UP	Vitvi017565	-1.963800118	2.02E-08	0.00000012	DOWN
Vitvi025479	1.248655769	7.06E-15	7.57E-14	UP	Vitvi015947	-1.964666175	0.006860962	0.017917186	DOWN
Vitvi011594	1.248632828	6.34E-08	0.000000357	UP	Vitvi030536	-1.966614665	0.000216955	0.000757961	DOWN
Vitvi020909	1.248537428	3.49E-18	4.8E-17	UP	Vitvi001520	-1.967119841	0.0000586	0.000223437	DOWN
Vitvi032690	1.248209057	7.09E-18	9.53E-17	UP	Vitvi007216	-1.96830559	1.56E-29	4.33E-28	DOWN
Vitvi018208	1.247816533	3.64E-09	2.36E-08	UP	Vitvi001432	-1.971017255	0.000138419	0.000497974	DOWN
Vitvi014708	1.245454746	0.000000115	0.000000631	UP	Vitvi003712	-1.971433976	0.006699547	0.01754302	DOWN
Vitvi014220	1.244825375	4.09E-11	3.18E-10	UP	Vitvi015327	-1.972520218	0.000203464	0.00071353	DOWN
Vitvi019479	1.244776013	1.35E-18	1.92E-17	UP	Vitvi023471	-1.97422907	0.000385338	0.001292649	DOWN
Vitvi018366	1.244342619	7.18E-20	1.13E-18	UP	Vitvi008246	-1.974577359	3.07E-16	3.68E-15	DOWN
Vitvi025480	1.243460491	0.0000215	0.0000878	UP	Vitvi011992	-1.976857514	1.28E-40	6.03E-39	DOWN
Vitvi016029	1.241812176	2.63E-29	7.2E-28	UP	Vitvi023484	-1.97692893	1.85E-14	1.94E-13	DOWN
Vitvi007035	1.241174937	3.12E-12	2.71E-11	UP	Vitvi005275	-1.981377016	1.72E-18	2.42E-17	DOWN
Vitvi032253	1.240586154	0.0000317	0.000126037	UP	Vitvi000896	-1.985193852	3.82E-15	4.19E-14	DOWN
Vitvi032917	1.240502932	8.17E-19	1.17E-17	UP	Vitvi001125	-1.986625486	1.88E-11	1.52E-10	DOWN
Vitvi030736	1.240421379	7.06E-25	1.52E-23	UP	Vitvi031594	-1.986818247	2.16E-14	2.25E-13	DOWN
Vitvi029513	1.240260404	8.44E-09	5.24E-08	UP	Vitvi010245	-1.988835649	8.74E-18	1.16E-16	DOWN
Vitvi012723	1.239749407	8.44E-32	2.68E-30	UP	Vitvi030234	-1.989307071	0.00000122	0.00000586	DOWN
Vitvi033744	1.239704403	8.57E-27	2.04E-25	UP	Vitvi023692	-1.989432674	2.39E-40	1.11E-38	DOWN
Vitvi023054	1.23965073	1.23E-20	2.04E-19	UP	Vitvi006389	-1.994249766	3.58E-56	3.6E-54	DOWN
Vitvi006405	1.239502721	7.19E-08	0.000000403	UP	Vitvi009360	-1.994478573	6.29E-09	3.97E-08	DOWN
Vitvi036352	1.239010387	0.000983875	0.003075954	UP	Vitvi031761	-1.996626476	9.2E-19	1.32E-17	DOWN
Vitvi005765	1.237399426	1.23E-14	1.3E-13	UP	Vitvi009212	-1.996814773	5.29E-10	3.71E-09	DOWN
Vitvi001826	1.236827123	2.34E-10	1.69E-09	UP	Vitvi018232	-1.99783458	0.000773461	0.002466719	DOWN
Vitvi033432	1.236787157	1.4E-11	1.14E-10	UP	Vitvi024219	-2.000291149	0.0000726	0.000272579	DOWN
Vitvi030701	1.235796716	6.02E-08	0.00000034	UP	Vitvi027717	-2.000875389	2.24E-11	1.79E-10	DOWN
Vitvi023417	1.23496872	0.00000183	0.00000864	UP	Vitvi023002	-2.001564067	4.58E-31	1.39E-29	DOWN
Vitvi022757	1.234921227	1.76E-30	5.16E-29	UP	Vitvi000510	-2.003827821	8.75E-20	1.37E-18	DOWN
Vitvi013783	1.234900049	0.002632586	0.007530361	UP	Vitvi014676	-2.009257859	0.000021	0.0000856	DOWN
Vitvi009698	1.234273688	0.000000359	0.00000186	UP	Vitvi000509	-2.01032221	1.23E-36	4.9E-35	DOWN
Vitvi011347	1.234002378	0.00146093	0.004428356	UP	Vitvi010970	-2.011962578	1.12E-31	3.53E-30	DOWN
Vitvi019126	1.233889123	3.29E-12	2.86E-11	UP	Vitvi025645	-2.012885959	1.57E-15	1.77E-14	DOWN
Vitvi033237	1.233734417	9.55E-16	1.09E-14	UP	Vitvi004031	-2.013090054	1.13E-25	2.54E-24	DOWN
Vitvi014558	1.232340873	0.000332692	0.001127966	UP	Vitvi012864	-2.014821403	1.15E-15	1.31E-14	DOWN
Vitvi000047	1.23195305	7.62E-31	2.27E-29	UP	Vitvi001941	-2.015082428	0.000535059	0.001753204	DOWN
Vitvi035593	1.228610482	0.000164815	0.000587011	UP	Vitvi012758	-2.01545626	2.31E-66	3.35E-64	DOWN
Vitvi027545	1.226209744	1.18E-12	1.06E-11	UP	Vitvi030646	-2.023256503	0.002553538	0.007327983	DOWN
Vitvi028126	1.22604308	3.3E-32	1.07E-30	UP	Vitvi016556	-2.025309008	0.002626019	0.007513692	DOWN
Vitvi034108	1.225965566	1.8E-16	2.2E-15	UP	Vitvi015143	-2.02644854	2.69E-16	3.23E-15	DOWN
Vitvi019794	1.224659701	3.61E-13	3.4E-12	UP	Vitvi000048	-2.030120263	3E-12	2.61E-11	DOWN
Vitvi000860	1.223830227	0.00000178	0.00000844	UP	Vitvi002042	-2.030439153	0.001817943	0.005392239	DOWN
Vitvi015770	1.223821407	6.18E-23	1.18E-21	UP	Vitvi032560	-2.030655881	1.01E-23	2.01E-22	DOWN
Vitvi025041	1.222787439	8.12E-12	6.78E-11	UP	Vitvi015445	-2.030854238	1.03E-21	1.84E-20	DOWN
Vitvi027403	1.221662697	0.001137957	0.003514932	UP	Vitvi025718	-2.031152668	3.56E-44	2E-42	DOWN
Vitvi014535	1.221155643	0.000000028	0.000000165	UP	Vitvi005240	-2.034777663	3.91E-13	3.67E-12	DOWN
Vitvi003940	1.220766211	3.14E-36	1.23E-34	UP	Vitvi012509	-2.039708104	0.004495775	0.012247609	DOWN
Vitvi010988	1.219531926	8.83E-10	6.1E-09	UP	Vitvi012612	-2.040913817	8.75E-35	3.16E-33	DOWN
Vitvi025454	1.219379148	0.0011836	0.003640964	UP	Vitvi031588	-2.041474799	2.1E-18	2.93E-17	DOWN
Vitvi018402	1.219178785	1.04E-10	1.53E-08	UP	Vitvi033354	-2.042053168	2.4E-15	2.66E-14	DOWN
Vitvi012600	1.218584223	0.005242152	0.014047308	UP	Vitvi005649	-2.045977887	0.013847164	0.033591735	DOWN
Vitvi029272	1.218344203	3.3E-28	8.55E-27	UP	Vitvi032841	-2.049774576	3.96E-46	2.44E-44	DOWN
Vitvi019859	1.218271463	5.94E-27	4.17E-24	UP	Vitvi026799	-2.051610728	0.007585385	0.019619807	DOWN
Vitvi010149	1.218032206	1.46E-15	1.64E-14	UP	Vitvi009622	-2.052011906	0.001864915	0.005515455	DOWN

ID	log2FoldChange	pvalue	padj	change	ID	log2FoldChange	pvalue	padj	change
Vitvi023038	1.217956152	0.00000371	0.0000169	UP	Vitvi010737	-2.053572971	1.84E-13	1.78E-12	DOWN
Vitvi034283	1.217220399	6.22E-32	1.99E-30	UP	Vitvi004215	-2.060155496	0.000000723	0.0000036	DOWN
Vitvi014963	1.216568692	1.27E-16	1.56E-15	UP	Vitvi025972	-2.062395047	8.6E-59	9.76E-57	DOWN
Vitvi016327	1.215174361	2.23E-32	7.27E-31	UP	Vitvi011608	-2.062862071	1.54E-22	2.84E-21	DOWN
Vitvi033552	1.214966953	1.43E-39	6.45E-38	UP	Vitvi033084	-2.064006416	9.39E-14	9.32E-13	DOWN
Vitvi024618	1.214242983	0.007539943	0.019512209	UP	Vitvi020950	-2.065163776	0.00256186	0.007349789	DOWN
Vitvi018411	1.213184169	0.004040123	0.011118138	UP	Vitvi012821	-2.06867966	0.00000962	0.0000412	DOWN
Vitvi001051	1.211951699	2.09E-18	2.93E-17	UP	Vitvi013044	-2.072951828	2.87E-14	2.96E-13	DOWN
Vitvi019647	1.211122121	1.42E-32	4.72E-31	UP	Vitvi034588	-2.07340663	2.67E-08	0.000000158	DOWN
Vitvi021163	1.210529405	3.14E-20	5.07E-19	UP	Vitvi025929	-2.073976909	8.21E-32	2.61E-30	DOWN
Vitvi010977	1.20891945	8.4E-24	1.69E-22	UP	Vitvi021905	-2.075644604	1.93E-08	0.000000115	DOWN
Vitvi020048	1.208325947	1.63E-17	2.14E-16	UP	Vitvi007214	-2.075677751	5.43E-09	3.46E-08	DOWN
Vitvi032226	1.208062596	9.74E-21	1.62E-19	UP	Vitvi002143	-2.08390779	0.000000952	0.00000466	DOWN
Vitvi000676	1.207485776	0.000238186	0.000827864	UP	Vitvi031293	-2.083927809	3.32E-30	9.6E-29	DOWN
Vitvi001056	1.207424247	8.53E-29	2.26E-27	UP	Vitvi014016	-2.085144212	0.001471704	0.004458352	DOWN
Vitvi031485	1.207236217	1.02E-12	9.28E-12	UP	Vitvi015789	-2.086482769	4.11E-11	3.19E-10	DOWN
Vitvi015492	1.20693717	0.0000043	0.0000193	UP	Vitvi026862	-2.087671733	8.44E-13	7.75E-12	DOWN
Vitvi010987	1.20638236	1.26E-16	1.54E-15	UP	Vitvi026885	-2.087873315	8.28E-17	1.04E-15	DOWN
Vitvi002679	1.205479118	4.11E-17	5.25E-16	UP	Vitvi019908	-2.089444318	1.62E-18	2.3E-17	DOWN
Vitvi025695	1.203205824	3.18E-18	4.37E-17	UP	Vitvi020244	-2.090342674	1.4E-19	2.15E-18	DOWN
Vitvi031793	1.201800067	1.76E-33	6.07E-32	UP	Vitvi002348	-2.092354933	6.38E-09	4.02E-08	DOWN
Vitvi002387	1.201301097	4.78E-09	3.07E-08	UP	Vitvi007878	-2.093688567	0.000505845	0.001665813	DOWN
Vitvi004568	1.199682026	0.000000216	0.00000115	UP	Vitvi010427	-2.098832048	3.18E-11	2.5E-10	DOWN
Vitvi010241	1.199192905	3.86E-10	2.74E-09	UP	Vitvi007857	-2.099700004	0.000000861	0.00000424	DOWN
Vitvi002245	1.199020805	0.020249437	0.046824517	UP	Vitvi032842	-2.103380968	0.00000768	0.0000334	DOWN
Vitvi019858	1.198465609	3.48E-15	1E-12	UP	Vitvi024168	-2.10656925	7.1E-30	2.01E-28	DOWN
Vitvi033420	1.197310317	4.54E-19	6.69E-18	UP	Vitvi033694	-2.108293058	7.84E-23	1.48E-21	DOWN
Vitvi007152	1.197173971	6.26E-08	0.000000352	UP	Vitvi028758	-2.11027192	0.000148024	0.000530028	DOWN
Vitvi014416	1.19579637	8.84E-11	6.64E-10	UP	Vitvi000379	-2.110464323	8.53E-17	1.07E-15	DOWN
Vitvi023352	1.195553013	1.18E-18	1.68E-17	UP	Vitvi031316	-2.111707726	6.24E-12	5.26E-11	DOWN
Vitvi021276	1.194887128	4.06E-13	3.8E-12	UP	Vitvi014504	-2.11183611	6.67E-13	6.17E-12	DOWN
Vitvi000926	1.19372291	6.93E-31	2.08E-29	UP	Vitvi032265	-2.113807395	1.2E-42	6.18E-41	DOWN
Vitvi022395	1.193632521	1.06E-12	9.6E-12	UP	Vitvi017497	-2.11757697	9.08E-49	3.19E-45	DOWN
Vitvi031837	1.192786389	2.4E-10	1.74E-09	UP	Vitvi011046	-2.120241284	5.35E-12	4.54E-11	DOWN
Vitvi011096	1.192496816	0.005495018	0.014645691	UP	Vitvi016065	-2.120399689	4.92E-17	6.24E-16	DOWN
Vitvi019268	1.192395006	2.38E-10	1.72E-09	UP	Vitvi015946	-2.122292977	0.005193308	0.013932964	DOWN
Vitvi023967	1.190742757	4.18E-18	5.72E-17	UP	Vitvi006905	-2.122646863	5.96E-17	7.51E-16	DOWN
Vitvi014219	1.188631595	2.05E-23	4E-22	UP	Vitvi019775	-2.122799539	1.14E-12	1.03E-11	DOWN
Vitvi001475	1.188538682	1.73E-12	1.54E-11	UP	Vitvi030393	-2.12712507	1.88E-14	1.96E-13	DOWN
Vitvi036503	1.187565054	3.72E-08	0.000000216	UP	Vitvi023966	-2.129257227	1.25E-26	2.97E-25	DOWN
Vitvi021099	1.187397529	1.41E-22	2.62E-21	UP	Vitvi004437	-2.130803643	1.29E-11	1.05E-10	DOWN
Vitvi003993	1.186883251	2.66E-23	5.15E-22	UP	Vitvi002855	-2.131216002	6.47E-31	1.95E-29	DOWN
Vitvi011630	1.186525699	2.42E-36	9.49E-35	UP	Vitvi010121	-2.131279138	0.000597123	0.00193998	DOWN
Vitvi032243	1.184699106	2.52E-33	8.6E-32	UP	Vitvi020455	-2.13161775	4.98E-58	5.43E-56	DOWN
Vitvi037468	1.183807698	4.49E-25	9.8E-24	UP	Vitvi001166	-2.132937989	0.0000824	0.000307019	DOWN
Vitvi000344	1.183637069	4.65E-19	6.85E-18	UP	Vitvi000823	-2.134311751	1.07E-23	2.13E-22	DOWN
Vitvi022033	1.183050425	7.74E-41	3.69E-39	UP	Vitvi033911	-2.135380565	7.94E-20	1.25E-18	DOWN
Vitvi023103	1.183003848	6.23E-08	0.000000351	UP	Vitvi011093	-2.135674157	0.000834654	0.002644439	DOWN
Vitvi008325	1.181801886	1E-29	2.81E-28	UP	Vitvi020065	-2.138677271	0.00000309	0.0000142	DOWN
Vitvi023119	1.181793362	3.04E-23	5.88E-22	UP	Vitvi016323	-2.140058862	0.00767835	0.0198451	DOWN
Vitvi031495	1.180324415	0.00119793	0.020173659	UP	Vitvi021423	-2.14608154	4.36E-21	7.45E-20	DOWN
Vitvi003966	1.180314	2.2E-09	1.46E-08	UP	Vitvi021520	-2.150295219	3.03E-44	1.71E-42	DOWN
Vitvi000598	1.179865534	1.27E-12	1.15E-11	UP	Vitvi001956	-2.154489509	0.0000163	0.0000675	DOWN
Vitvi031611	1.179077811	2.19E-21	3.79E-20	UP	Vitvi014520	-2.155100569	9.95E-19	1.42E-17	DOWN
Vitvi008725	1.176084506	0.000000028	0.000000164	UP	Vitvi003622	-2.156579785	0.007108505	0.018494678	DOWN
Vitvi007930	1.172722937	2.93E-11	2.32E-10	UP	Vitvi003598	-2.159430865	6.51E-27	1.57E-25	DOWN
Vitvi004479	1.172361835	0.001977391	0.005810034	UP	Vitvi022330	-2.15945375	2.81E-18	3.89E-17	DOWN

ID	log2FoldChange	pvalue	padj	change	ID	log2FoldChange	pvalue	padj	change
Vitvi001847	1.172109198	3.3E-16	3.93E-15	UP	Vitvi004287	-2.160815196	1.85E-26	4.33E-25	DOWN
Vitvi034654	1.171270532	6E-26	1.37E-24	UP	Vitvi028760	-2.161861595	7.27E-55	6.84E-53	DOWN
Vitvi033939	1.171171161	1.87E-30	5.49E-29	UP	Vitvi001967	-2.175263695	0.012715308	0.031132273	DOWN
Vitvi011605	1.168527311	8.31E-08	0.000000463	UP	Vitvi035102	-2.175588713	0.0000028	0.0000129	DOWN
Vitvi011422	1.167985987	0.000000001	6.89E-09	UP	Vitvi017943	-2.176344213	0.000207187	0.000725834	DOWN
Vitvi030510	1.167567867	0.003852136	0.010657122	UP	Vitvi018530	-2.176786258	4.44E-08	0.000000255	DOWN
Vitvi018506	1.167540707	0.0088451	0.022587588	UP	Vitvi011237	-2.177052955	3.27E-26	7.57E-25	DOWN
Vitvi002152	1.166590616	0.0000552	0.000211019	UP	Vitvi010150	-2.182221829	0.0000058	0.0000257	DOWN
Vitvi010646	1.166096881	0.000123867	0.000449203	UP	Vitvi019579	-2.186499396	3E-56	3.03E-54	DOWN
Vitvi023203	1.165617263	8.09E-18	1.08E-16	UP	Vitvi017670	-2.186767718	0.004411303	0.012041712	DOWN
Vitvi009429	1.165085371	4.24E-08	0.000000244	UP	Vitvi011990	-2.188134072	1.98E-08	0.000000118	DOWN
Vitvi008276	1.164065919	0.002562789	0.00735087	UP	Vitvi002159	-2.190444753	1.98E-12	1.76E-11	DOWN
Vitvi026548	1.162991825	5.84E-10	4.09E-09	UP	Vitvi007579	-2.190644311	2.94E-13	2.77E-12	DOWN
Vitvi035975	1.161284499	2.66E-15	2.95E-14	UP	Vitvi026394	-2.194990432	1.08E-21	1.91E-20	DOWN
Vitvi015554	1.161034089	9.9E-20	1.53E-18	UP	Vitvi000872	-2.195025082	7.49E-40	3.41E-38	DOWN
Vitvi018444	1.159246983	3.26E-08	0.00000019	UP	Vitvi031763	-2.202393502	1.07E-24	2.29E-23	DOWN
Vitvi032981	1.1591366	0.000106858	0.000392142	UP	Vitvi031961	-2.204173614	1.43E-29	3.97E-28	DOWN
Vitvi016617	1.158465392	0.000590504	0.01150467	UP	Vitvi014811	-2.205105582	1.91E-24	4E-23	DOWN
Vitvi025779	1.15730109	0.00000605	0.0000267	UP	Vitvi032757	-2.206349175	1.13E-12	1.02E-11	DOWN
Vitvi033527	1.156494364	2.35E-18	3.28E-17	UP	Vitvi032130	-2.208008674	3.8E-46	2.35E-44	DOWN
Vitvi027100	1.1559568	0.000281901	0.000966735	UP	Vitvi030628	-2.212666485	0.00000655	0.0000287	DOWN
Vitvi031846	1.155683413	7.58E-10	5.27E-09	UP	Vitvi008237	-2.212749958	0.00000913	0.0000392	DOWN
Vitvi007890	1.155639634	1.75E-28	4.55E-27	UP	Vitvi016629	-2.219428585	1.63E-09	1.09E-08	DOWN
Vitvi004304	1.155209137	7.58E-23	1.44E-21	UP	Vitvi012112	-2.22345667	0.000000451	0.00000232	DOWN
Vitvi028929	1.154765193	4.47E-08	0.000000257	UP	Vitvi016326	-2.227667467	6.95E-57	7.24E-55	DOWN
Vitvi031520	1.154724546	3.51E-10	2.51E-09	UP	Vitvi003693	-2.239996952	0.000282958	0.000969869	DOWN
Vitvi010456	1.153036488	4.42E-08	0.000000254	UP	Vitvi004512	-2.24361509	5.73E-15	6.2E-14	DOWN
Vitvi016315	1.15235475	5.64E-31	1.7E-29	UP	Vitvi005387	-2.244024884	1.19E-35	4.5E-34	DOWN
Vitvi013378	1.151842652	4.45E-25	9.73E-24	UP	Vitvi004005	-2.245858432	5.97E-11	4.57E-10	DOWN
Vitvi018057	1.151744151	1.18E-20	1.95E-19	UP	Vitvi000683	-2.246471196	2.32E-11	1.85E-10	DOWN
Vitvi013552	1.151534491	2.1E-12	1.85E-11	UP	Vitvi023619	-2.248524624	2.76E-08	0.00000257	DOWN
Vitvi012848	1.151173323	0.00000615	0.0000271	UP	Vitvi014340	-2.250061234	2.25E-12	1.98E-11	DOWN
Vitvi005833	1.150696121	3.65E-24	7.51E-23	UP	Vitvi011384	-2.252525124	3.74E-10	2.66E-09	DOWN
Vitvi012373	1.150631154	0.002709032	0.037444366	UP	Vitvi016049	-2.253717128	0.0000488	0.000188035	DOWN
Vitvi010906	1.14866115	7.36E-12	6.17E-11	UP	Vitvi015175	-2.254366747	0.000272993	0.000939392	DOWN
Vitvi022448	1.148448843	0.004554896	0.01239703	UP	Vitvi003865	-2.256306265	0.003924252	0.010843343	DOWN
Vitvi030244	1.147995442	0.000000541	0.00000275	UP	Vitvi027248	-2.261471276	0.00000212	0.00000996	DOWN
Vitvi024956	1.147355529	0.000138756	0.000499097	UP	Vitvi031680	-2.262074566	0.003508679	0.009782881	DOWN
Vitvi017771	1.146821039	2.79E-09	1.83E-08	UP	Vitvi028937	-2.262577572	2.22E-21	3.84E-20	DOWN
Vitvi006501	1.146710015	6.12E-30	1.74E-28	UP	Vitvi031365	-2.265590598	2.55E-28	6.61E-27	DOWN
Vitvi005584	1.146661006	0.00000379	0.0000172	UP	Vitvi018365	-2.269352568	0.000137718	0.000495628	DOWN
Vitvi010865	1.143915775	1.09E-11	8.99E-11	UP	Vitvi016183	-2.271538504	4.04E-16	4.76E-15	DOWN
Vitvi031244	1.143649515	7.75E-08	0.000000433	UP	Vitvi033593	-2.272084215	0.001756394	0.005228767	DOWN
Vitvi015244	1.143607876	1.06E-19	1.63E-18	UP	Vitvi021049	-2.273551219	9.37E-39	4.09E-37	DOWN
Vitvi031598	1.143146896	6.4E-10	4.46E-09	UP	Vitvi003639	-2.274562527	0.01171455	0.028985983	DOWN
Vitvi005018	1.14101996	1.87E-13	1.8E-12	UP	Vitvi021118	-2.275283251	8.58E-23	1.62E-21	DOWN
Vitvi022300	1.140970465	7.9E-13	7.27E-12	UP	Vitvi000540	-2.277874204	1.46E-22	2.71E-21	DOWN
Vitvi015639	1.14036849	0.00034375	0.001162933	UP	Vitvi010018	-2.282269558	2.84E-10	2.04E-09	DOWN
Vitvi021257	1.140031243	1.95E-16	2.37E-15	UP	Vitvi024629	-2.282383896	1.86E-41	9.08E-40	DOWN
Vitvi000590	1.13995689	0.000186711	0.004587702	UP	Vitvi028877	-2.283517212	9.03E-15	9.63E-14	DOWN
Vitvi036206	1.13919772	5.78E-22	1.04E-20	UP	Vitvi035878	-2.285700642	0.012338421	0.030355864	DOWN
Vitvi003901	1.139013128	6.3E-16	7.3E-15	UP	Vitvi029081	-2.288328954	1.64E-32	5.4E-31	DOWN
Vitvi004143	1.138947897	2.18E-13	2.09E-12	UP	Vitvi003621	-2.290878422	2.44E-14	2.53E-13	DOWN
Vitvi035753	1.138938362	0.00000587	0.000026	UP	Vitvi021411	-2.291164421	8.44E-30	2.38E-28	DOWN
Vitvi033227	1.138591666	1.27E-19	1.96E-18	UP	Vitvi018410	-2.291800108	1.5E-28	3.93E-27	DOWN
Vitvi000075	1.138551717	1.93E-11	3.26E-09	UP	Vitvi032953	-2.293477391	0.00000379	0.0000172	DOWN
Vitvi011598	1.137667219	0.019595996	0.045552056	UP	Vitvi033677	-2.294776784	0.011504999	0.028533521	DOWN

ID	log2FoldChange	pvalue	padj	change	ID	log2FoldChange	pvalue	padj	change
Vitvi013801	1.137442597	0.000395494	0.001324312	UP	Vitvi018956	-2.296675444	2.01E-17	2.63E-16	DOWN
Vitvi029295	1.137403395	4.5E-12	3.85E-11	UP	Vitvi034483	-2.297724154	1.87E-09	1.25E-08	DOWN
Vitvi007935	1.137169605	1.77E-14	1.85E-13	UP	Vitvi002628	-2.302430591	1.76E-18	2.48E-17	DOWN
Vitvi033316	1.136963347	1.14E-22	2.14E-21	UP	Vitvi000261	-2.310618818	0.00000153	0.00000731	DOWN
Vitvi029664	1.136954614	0.0000277	0.000110932	UP	Vitvi004580	-2.311603969	8.8E-18	1.17E-16	DOWN
Vitvi010964	1.136315428	3.84E-29	1.04E-27	UP	Vitvi027497	-2.314561943	0.001908508	0.005626358	DOWN
Vitvi003899	1.135978167	3.39E-30	9.79E-29	UP	Vitvi020454	-2.317153722	0.0000273	0.000109644	DOWN
Vitvi007688	1.13595797	0.006991048	0.018217084	UP	Vitvi000051	-2.318631714	4.92E-73	9.25E-71	DOWN
Vitvi007306	1.135532682	0.00000264	0.0000122	UP	Vitvi000385	-2.328314038	1.86E-14	1.94E-13	DOWN
Vitvi000332	1.135418282	2.33E-08	0.000000138	UP	Vitvi028241	-2.333146374	4.07E-08	0.000000235	DOWN
Vitvi000980	1.135247296	2.75E-08	0.000000162	UP	Vitvi035158	-2.335074728	0.00927189	0.023556051	DOWN
Vitvi029971	1.134785083	0.000000988	0.00000483	UP	Vitvi020013	-2.335913971	0.017551186	0.04136207	DOWN
Vitvi031462	1.134261045	0.009459344	0.02397298	UP	Vitvi020452	-2.341626974	0.009334708	0.023703786	DOWN
Vitvi004367	1.133971823	1.4E-09	9.48E-09	UP	Vitvi001641	-2.342064439	3.16E-18	4.35E-17	DOWN
Vitvi031459	1.133696613	0.0000303	0.00012091	UP	Vitvi029077	-2.343436779	0.019267454	0.044891091	DOWN
Vitvi020926	1.133531766	8.22E-20	1.29E-18	UP	Vitvi020329	-2.345502175	0.00375993	0.010426172	DOWN
Vitvi002196	1.133119802	1.07E-42	5.57E-41	UP	Vitvi028685	-2.347581446	8.18E-35	2.96E-33	DOWN
Vitvi009933	1.13236076	3.83E-21	6.56E-20	UP	Vitvi026913	-2.349590193	2.63E-42	1.34E-40	DOWN
Vitvi003838	1.131237095	1.36E-21	2.38E-20	UP	Vitvi003862	-2.355343848	0.000555281	0.00181245	DOWN
Vitvi013050	1.130956386	1.37E-16	1.69E-15	UP	Vitvi004276	-2.357896555	4.64E-58	5.09E-56	DOWN
Vitvi013796	1.130832163	0.007963265	0.020511011	UP	Vitvi033470	-2.358614615	4.76E-36	1.84E-34	DOWN
Vitvi036598	1.130809347	0.006940372	0.018103613	UP	Vitvi027596	-2.359408276	0.00000942	0.0000404	DOWN
Vitvi011860	1.130247514	0.00000912	0.0000392	UP	Vitvi021159	-2.363008355	0.000000155	0.000000837	DOWN
Vitvi001001	1.129896054	6.8E-25	1.47E-23	UP	Vitvi011577	-2.364901268	1.17E-60	1.43E-58	DOWN
Vitvi016161	1.129568816	6.59E-09	4.14E-08	UP	Vitvi034027	-2.372821189	0.000335088	0.001135521	DOWN
Vitvi025209	1.12950139	3.49E-12	3.03E-11	UP	Vitvi017473	-2.374939443	0.00000227	0.0000106	DOWN
Vitvi008450	1.129195635	0.0000188	0.0000773	UP	Vitvi013891	-2.376368475	7.31E-13	6.74E-12	DOWN
Vitvi013317	1.128601617	9.04E-36	3.44E-34	UP	Vitvi006635	-2.381780445	0.0212497	0.048815191	DOWN
Vitvi036542	1.128125021	2.27E-26	5.3E-25	UP	Vitvi020394	-2.382432052	0.00000521	0.0000232	DOWN
Vitvi027015	1.127843862	6.56E-21	1.1E-19	UP	Vitvi002386	-2.382784331	3.54E-19	5.25E-18	DOWN
Vitvi007051	1.1274936	7.25E-13	6.69E-12	UP	Vitvi019369	-2.385898016	4.98E-20	7.91E-19	DOWN
Vitvi009511	1.126836528	4.59E-21	7.83E-20	UP	Vitvi009739	-2.386427354	0.000282709	0.000969178	DOWN
Vitvi001531	1.126470579	7.95E-12	6.64E-11	UP	Vitvi011322	-2.401941161	3.57E-47	2.39E-45	DOWN
Vitvi022067	1.126286905	1.52E-25	3.38E-24	UP	Vitvi010020	-2.407447319	0.001326199	0.004049572	DOWN
Vitvi011526	1.125631666	3.04E-18	4.2E-17	UP	Vitvi027615	-2.410989769	0.012848013	0.031411753	DOWN
Vitvi019705	1.125428454	1.16E-08	7.09E-08	UP	Vitvi013544	-2.413990682	4.21E-14	4.3E-13	DOWN
Vitvi024954	1.123470785	0.008804769	0.022490251	UP	Vitvi018861	-2.418385348	2.04E-33	7.01E-32	DOWN
Vitvi015622	1.122552176	1.33E-13	1.31E-12	UP	Vitvi035684	-2.418911693	2.55E-23	4.96E-22	DOWN
Vitvi000629	1.122502592	0.000000639	0.0000032	UP	Vitvi023194	-2.422608167	2.37E-58	2.65E-56	DOWN
Vitvi033860	1.121473303	0.018848394	0.044020763	UP	Vitvi027405	-2.42915716	9.88E-90	3.18E-87	DOWN
Vitvi012874	1.120500209	1.35E-08	8.15E-08	UP	Vitvi007224	-2.429249765	1.1E-21	1.94E-20	DOWN
Vitvi000167	1.120114991	2.62E-08	0.000000154	UP	Vitvi009495	-2.431518293	8.96E-49	6.38E-47	DOWN
Vitvi020220	1.119205972	3.23E-16	3.86E-15	UP	Vitvi005458	-2.432897356	0.000159228	0.00056771	DOWN
Vitvi022670	1.119196412	0.015206657	0.036428542	UP	Vitvi023134	-2.435387172	1.27E-22	2.37E-21	DOWN
Vitvi001917	1.117000866	7.13E-11	5.4E-10	UP	Vitvi028054	-2.438611265	7.13E-12	5.98E-11	DOWN
Vitvi035584	1.115453904	0.019328572	0.045023159	UP	Vitvi028352	-2.444546902	0.000000165	0.000000888	DOWN
Vitvi009569	1.114558961	2.45E-09	1.61E-08	UP	Vitvi014024	-2.450049535	1.4E-22	2.6E-21	DOWN
Vitvi033509	1.114326507	0.000823513	0.002614085	UP	Vitvi003625	-2.459516517	1.18E-10	8.82E-10	DOWN
Vitvi026918	1.114056569	0.00747531	0.019367155	UP	Vitvi022118	-2.464544822	0.0000273	0.000109499	DOWN
Vitvi027434	1.113436051	8.37E-10	5.8E-09	UP	Vitvi014836	-2.47344495	0.000149084	0.000533601	DOWN
Vitvi029538	1.111474084	0.000542971	0.001776776	UP	Vitvi033842	-2.484360741	4.45E-08	0.000000255	DOWN
Vitvi012480	1.110773946	0.002623483	0.036549704	UP	Vitvi000962	-2.487661751	1.63E-09	1.09E-08	DOWN
Vitvi034673	1.110580811	1.65E-12	1.47E-11	UP	Vitvi030540	-2.48979355	0.00385626	0.010667077	DOWN
Vitvi014474	1.109351883	1.19E-24	7.73E-22	UP	Vitvi008850	-2.492685731	9.26E-10	6.38E-09	DOWN
Vitvi022864	1.109320738	0.012520301	0.030732611	UP	Vitvi032996	-2.492818436	2.99E-55	2.85E-53	DOWN
Vitvi004917	1.108211328	1.56E-14	1.63E-13	UP	Vitvi016048	-2.493944369	4.27E-38	1.81E-36	DOWN
Vitvi029670	1.108204305	0.000134094	0.000483356	UP	Vitvi027628	-2.500419425	1.59E-79	3.55E-77	DOWN

ID	log2FoldChange	pvalue	padj	change	ID	log2FoldChange	pvalue	padj	change
Vitvi007299	1.107516682	4.71E-15	5.14E-14	UP	Vitvi003692	-2.500921252	1.51E-09	1.02E-08	DOWN
Vitvi018081	1.105993148	0.000000552	0.0000028	UP	Vitvi005881	-2.507603944	1.05E-37	4.43E-36	DOWN
Vitvi001351	1.105621658	4.09E-25	2.76E-22	UP	Vitvi030970	-2.508944369	0.000000358	0.00000186	DOWN
Vitvi000116	1.105266537	2.3E-11	1.84E-10	UP	Vitvi031839	-2.511307193	2.69E-106	1.21E-103	DOWN
Vitvi024949	1.105212167	0.006922684	0.018062115	UP	Vitvi034135	-2.519334031	0.007850081	0.020247725	DOWN
Vitvi015557	1.104042988	0.002707027	0.007725887	UP	Vitvi010268	-2.520598869	1.3E-11	1.06E-10	DOWN
Vitvi033094	1.102327243	1.58E-12	1.41E-11	UP	Vitvi018418	-2.523706727	0.000000425	0.00000219	DOWN
Vitvi019709	1.101514166	1.42E-09	9.56E-09	UP	Vitvi023193	-2.534883641	9.43E-129	7.66E-126	DOWN
Vitvi013053	1.101052309	9.35E-20	1.45E-18	UP	Vitvi019595	-2.539859226	2.18E-61	2.67E-59	DOWN
Vitvi006589	1.101031908	2.75E-36	1.08E-34	UP	Vitvi000297	-2.540882433	1.7E-15	1.91E-14	DOWN
Vitvi031137	1.100890258	6.95E-34	2.44E-32	UP	Vitvi011563	-2.541659691	0.001017336	0.003173239	DOWN
Vitvi020243	1.10075107	1.6E-13	1.56E-12	UP	Vitvi004371	-2.544456048	6.98E-14	7.01E-13	DOWN
Vitvi031350	1.100072176	2.11E-13	2.02E-12	UP	Vitvi003907	-2.549537025	0.00000489	0.0000218	DOWN
Vitvi017044	1.099818068	1.87E-10	1.37E-09	UP	Vitvi026640	-2.551578658	0.0000133	0.000056	DOWN
Vitvi018495	1.098993189	1.73E-11	1.4E-10	UP	Vitvi037340	-2.553435098	0.000550013	0.001797274	DOWN
Vitvi035934	1.098386062	7.13E-10	0.00000009	UP	Vitvi020453	-2.555723579	0.000523262	0.001717874	DOWN
Vitvi016152	1.098364334	1.37E-15	1.54E-14	UP	Vitvi037237	-2.556732328	0.021580577	0.049474593	DOWN
Vitvi033563	1.097115049	1.09E-17	1.44E-16	UP	Vitvi015412	-2.559120796	2.99E-46	1.86E-44	DOWN
Vitvi030251	1.096738641	7.74E-09	4.82E-08	UP	Vitvi028943	-2.583431526	6.48E-11	4.94E-10	DOWN
Vitvi011140	1.094647719	0.000000138	0.00000075	UP	Vitvi015417	-2.5968166	0.008288747	0.021276511	DOWN
Vitvi002660	1.09431776	2.25E-34	7.98E-33	UP	Vitvi014801	-2.597828315	0.0000507	0.000195222	DOWN
Vitvi034658	1.092704544	0.00000028	0.00000147	UP	Vitvi009940	-2.607079746	1.7E-18	2.4E-17	DOWN
Vitvi023438	1.092605979	1.46E-10	1.08E-09	UP	Vitvi006950	-2.614996638	3.96E-15	4.34E-14	DOWN
Vitvi019867	1.091976584	9.21E-09	5.69E-08	UP	Vitvi035862	-2.616688037	1.65E-25	3.66E-24	DOWN
Vitvi006140	1.090835969	0.01240224	0.030501792	UP	Vitvi007752	-2.617751453	0.0000486	0.000187598	DOWN
Vitvi029063	1.090757125	1.82E-09	1.22E-08	UP	Vitvi036517	-2.619743463	6.16E-45	3.64E-43	DOWN
Vitvi024909	1.090489159	0.001316306	0.021594793	UP	Vitvi032457	-2.627888682	0.00000747	0.0000325	DOWN
Vitvi026026	1.089897603	5.4E-13	5.01E-12	UP	Vitvi012456	-2.629524203	0.0000817	0.000304607	DOWN
Vitvi028716	1.089200325	2.92E-13	2.77E-12	UP	Vitvi005546	-2.643670877	1.53E-46	9.68E-45	DOWN
Vitvi001846	1.087837103	1.17E-14	1.23E-13	UP	Vitvi036586	-2.645679612	3.74E-21	6.4E-20	DOWN
Vitvi000606	1.086896843	1.26E-15	1.42E-14	UP	Vitvi021675	-2.65716749	0.00000138	0.0000066	DOWN
Vitvi002932	1.086337232	0.004707383	0.012760757	UP	Vitvi035117	-2.668948411	2.96E-14	3.05E-13	DOWN
Vitvi026719	1.085919668	8.46E-13	7.77E-12	UP	Vitvi005606	-2.672024434	8.32E-10	5.77E-09	DOWN
Vitvi036522	1.085201823	7.74E-22	1.38E-20	UP	Vitvi005555	-2.683581767	6.66E-19	9.67E-18	DOWN
Vitvi021124	1.084996624	0.000000271	0.00000142	UP	Vitvi024534	-2.693433775	4.14E-13	3.87E-12	DOWN
Vitvi023779	1.08218724	2.05E-16	2.49E-15	UP	Vitvi002254	-2.719375789	0.000555102	0.001812155	DOWN
Vitvi007767	1.08183692	2.49E-24	5.19E-23	UP	Vitvi025044	-2.721432989	2.36E-09	1.56E-08	DOWN
Vitvi002934	1.080480278	2.28E-16	2.76E-15	UP	Vitvi029185	-2.722076754	1.8E-42	9.26E-41	DOWN
Vitvi011489	1.080021269	0.011638392	0.028818606	UP	Vitvi000865	-2.725610143	0.011575463	0.028687263	DOWN
Vitvi026860	1.079981642	2.96E-16	3.56E-15	UP	Vitvi026720	-2.727045657	8.59E-41	4.08E-39	DOWN
Vitvi024982	1.07974926	4.34E-33	1.46E-31	UP	Vitvi014341	-2.72820331	5.17E-26	1.18E-24	DOWN
Vitvi017553	1.079484879	6.05E-26	1.38E-24	UP	Vitvi011250	-2.730881302	3.72E-40	1.72E-38	DOWN
Vitvi006641	1.079367433	3.65E-19	5.42E-18	UP	Vitvi023570	-2.736373645	0.000047	0.000182071	DOWN
Vitvi003189	1.079329039	0.000000532	0.0000027	UP	Vitvi010765	-2.750078371	1.03E-50	8.01E-49	DOWN
Vitvi025931	1.078460793	0.001473692	0.004463709	UP	Vitvi002067	-2.753848528	0.0000269	0.000108077	DOWN
Vitvi027525	1.077513957	5.76E-23	1.1E-21	UP	Vitvi013055	-2.761421757	1.82E-24	3.82E-23	DOWN
Vitvi036177	1.075752713	2E-12	1.77E-11	UP	Vitvi005354	-2.763246925	0.00000859	0.0000371	DOWN
Vitvi022559	1.0755514	0.000000245	0.00000129	UP	Vitvi014505	-2.765295829	2.91E-11	2.3E-10	DOWN
Vitvi002335	1.07467127	2.43E-12	2.13E-11	UP	Vitvi004253	-2.76642787	1.08E-12	9.8E-12	DOWN
Vitvi022513	1.074474032	0.000000568	0.00000287	UP	Vitvi033801	-2.776109142	1.16E-10	8.66E-10	DOWN
Vitvi021757	1.074417739	0.004706625	0.012760406	UP	Vitvi000346	-2.781247054	0.002573775	0.007378759	DOWN
Vitvi028492	1.07440632	0.000059	0.000224896	UP	Vitvi011394	-2.784253121	2.78E-50	2.11E-48	DOWN
Vitvi035493	1.072708075	0.009757995	0.024644808	UP	Vitvi024641	-2.800821717	1.39E-12	1.25E-11	DOWN
Vitvi016859	1.07233943	1.02E-20	1.7E-19	UP	Vitvi009975	-2.803229039	2.28E-11	1.82E-10	DOWN
Vitvi008066	1.072248895	2.87E-18	3.96E-17	UP	Vitvi003450	-2.803806429	0.00000043	0.00000221	DOWN
Vitvi003830	1.0716078	2.46E-20	4E-19	UP	Vitvi003596	-2.805217389	0.010051522	0.025318269	DOWN
Vitvi020183	1.071342252	4.3E-26	9.89E-25	UP	Vitvi019916	-2.80562058	1.53E-29	4.23E-28	DOWN

ID	log2FoldChange	pvalue	padj	change	ID	log2FoldChange	pvalue	padj	change
Vitvi020456	1.069264353	3.34E-33	1.13E-31	UP	Vitvi000257	-2.806456293	1.92E-08	0.000000115	DOWN
Vitvi000448	1.068795465	1.36E-09	9.23E-09	UP	Vitvi032715	-2.810239986	2.41E-10	1.74E-09	DOWN
Vitvi010580	1.068648874	0.010376176	0.02606169	UP	Vitvi032806	-2.811411124	0.000303777	0.001036415	DOWN
Vitvi016863	1.068165312	1.31E-12	1.18E-11	UP	Vitvi000296	-2.812480707	0.000193586	0.000681121	DOWN
Vitvi017037	1.06816523	0.008412057	0.021579401	UP	Vitvi017770	-2.836557358	0.009717728	0.02455056	DOWN
Vitvi019398	1.067220454	0.004187472	0.011478533	UP	Vitvi034325	-2.840100097	0.00000618	0.0000272	DOWN
Vitvi014812	1.065781903	5.03E-19	7.38E-18	UP	Vitvi011539	-2.852277477	0.000764905	0.002442117	DOWN
Vitvi034030	1.064973632	3.18E-15	3.5E-14	UP	Vitvi032623	-2.860351927	6.36E-60	7.47E-58	DOWN
Vitvi022064	1.064044779	1.65E-13	1.6E-12	UP	Vitvi003691	-2.869316028	7.82E-16	9.02E-15	DOWN
Vitvi025576	1.064027283	0.00000905	0.0000389	UP	Vitvi001787	-2.874026243	0.00000437	0.0000197	DOWN
Vitvi014525	1.063971753	1.97E-16	2.39E-15	UP	Vitvi011613	-2.892987386	2.48E-14	2.57E-13	DOWN
Vitvi004198	1.063785606	5.45E-08	0.00000031	UP	Vitvi022349	-2.900205427	0.00000222	0.000123598	DOWN
Vitvi016293	1.063163324	0.0000389	0.000152408	UP	Vitvi030325	-2.904509035	3.55E-37	1.44E-35	DOWN
Vitvi014743	1.062658213	6.3E-16	7.3E-15	UP	Vitvi012953	-2.906354561	7.51E-11	5.68E-10	DOWN
Vitvi023384	1.062431144	1.51E-12	1.35E-11	UP	Vitvi005466	-2.910794592	0.000000506	0.00000258	DOWN
Vitvi022447	1.061705241	4.68E-11	3.62E-10	UP	Vitvi000748	-2.929445832	3.44E-12	2.98E-11	DOWN
Vitvi030719	1.060595187	7.17E-21	1.21E-19	UP	Vitvi023075	-2.945415439	0.001651463	0.004948298	DOWN
Vitvi015330	1.060569806	3.13E-08	0.000000183	UP	Vitvi016890	-2.946811423	2.58E-24	5.38E-23	DOWN
Vitvi035589	1.060454576	0.000000498	0.00000254	UP	Vitvi003617	-2.950409955	0.005659269	0.015046546	DOWN
Vitvi030738	1.06006255	0.0000268	0.000107584	UP	Vitvi029492	-2.96304315	0.021422934	0.049155726	DOWN
Vitvi014494	1.059575634	0.000448666	0.001488616	UP	Vitvi017789	-2.978382222	0.00000311	0.0000143	DOWN
Vitvi027225	1.05942626	0.00158171	0.024924009	UP	Vitvi006928	-2.984289238	3.26E-29	8.86E-28	DOWN
Vitvi005483	1.059135697	0.000000274	0.00000144	UP	Vitvi030275	-2.99216288	5.82E-46	3.56E-44	DOWN
Vitvi016015	1.058992791	9.97E-24	1.99E-22	UP	Vitvi002597	-2.998348153	2.05E-21	3.55E-20	DOWN
Vitvi036311	1.058700551	4.53E-10	3.2E-09	UP	Vitvi023640	-3.007683294	2.62E-10	1.89E-09	DOWN
Vitvi007240	1.057367034	0.020236714	0.046800962	UP	Vitvi001781	-3.035230572	6.3E-11	4.81E-10	DOWN
Vitvi009531	1.05726433	4.1E-16	4.82E-15	UP	Vitvi015306	-3.039053155	0.000837534	0.002651494	DOWN
Vitvi009032	1.056464634	0.005252746	0.01407384	UP	Vitvi016417	-3.042348784	0.007541895	0.019514773	DOWN
Vitvi020559	1.054750556	1.03E-26	2.45E-25	UP	Vitvi026508	-3.051477803	1.63E-12	1.45E-11	DOWN
Vitvi008205	1.054521076	2.79E-08	0.000000164	UP	Vitvi030132	-3.064336703	0.014787539	0.035533477	DOWN
Vitvi021932	1.053951146	3.95E-09	2.55E-08	UP	Vitvi031002	-3.067472496	8.35E-08	0.000000464	DOWN
Vitvi013008	1.053730589	0.000788211	0.002511427	UP	Vitvi000831	-3.071763324	1.84E-84	5.11E-82	DOWN
Vitvi031993	1.053375302	1.69E-20	2.79E-19	UP	Vitvi013158	-3.07361385	1.47E-83	3.98E-81	DOWN
Vitvi013165	1.053073446	1.3E-11	1.06E-10	UP	Vitvi013059	-3.075682057	0.016323081	0.038805527	DOWN
Vitvi001224	1.052843806	9.14E-11	6.86E-10	UP	Vitvi000951	-3.08981418	0.000656652	0.002119484	DOWN
Vitvi017664	1.052332449	0.00000001	6.18E-08	UP	Vitvi011614	-3.098962234	0.000820585	0.002606292	DOWN
Vitvi004071	1.051715538	0.000762201	0.002435868	UP	Vitvi036006	-3.101635153	0.0000915	0.000338826	DOWN
Vitvi005462	1.051111778	1.55E-27	3.85E-26	UP	Vitvi028667	-3.111701701	0.000494355	0.001631148	DOWN
Vitvi005853	1.049739882	1.7E-12	1.52E-11	UP	Vitvi031360	-3.156628974	1.48E-10	1.09E-09	DOWN
Vitvi005792	1.049391527	1.41E-19	2.15E-18	UP	Vitvi007500	-3.174181618	1.01E-89	3.21E-87	DOWN
Vitvi002647	1.0487581	1.71E-31	5.33E-30	UP	Vitvi015103	-3.179476377	1.48E-106	7.01E-104	DOWN
Vitvi003556	1.048524199	4.13E-08	0.000000239	UP	Vitvi029445	-3.190253702	0.0000948	0.00035039	DOWN
Vitvi002846	1.048499599	5.91E-12	5E-11	UP	Vitvi028835	-3.233847635	0.008708971	0.022265151	DOWN
Vitvi004197	1.048038133	2.61E-13	2.48E-12	UP	Vitvi015136	-3.261874497	0.020114495	0.046578654	DOWN
Vitvi006692	1.048004362	7.12E-10	4.95E-09	UP	Vitvi035887	-3.266967765	0.017660788	0.041586591	DOWN
Vitvi007962	1.045629049	7.43E-22	1.33E-20	UP	Vitvi007137	-3.273280796	0.0192195	0.044789637	DOWN
Vitvi025669	1.045268304	0.00000135	0.00000647	UP	Vitvi030403	-3.291630327	0.0000197	0.0000809	DOWN
Vitvi020813	1.044891108	8.3E-14	8.31E-13	UP	Vitvi030537	-3.294778367	0.000347117	0.001173541	DOWN
Vitvi015564	1.044134231	9.95E-28	2.5E-26	UP	Vitvi001777	-3.347922343	0.015497074	0.037028182	DOWN
Vitvi035726	1.043371178	3.4E-27	8.28E-26	UP	Vitvi006429	-3.358653669	0.015272918	0.036565712	DOWN
Vitvi012997	1.042867632	9.73E-16	1.11E-14	UP	Vitvi013253	-3.360163134	4.75E-22	8.58E-21	DOWN
Vitvi011416	1.041747262	4.19E-20	6.7E-19	UP	Vitvi035683	-3.365789745	6.85E-16	7.91E-15	DOWN
Vitvi014646	1.041363532	8.48E-10	5.88E-09	UP	Vitvi014478	-3.380192509	0.000764062	0.002440576	DOWN
Vitvi016188	1.040317036	0.000274199	0.00094255	UP	Vitvi013406	-3.388605043	0.007575776	0.019597448	DOWN
Vitvi010189	1.040008299	0.000244116	0.00084688	UP	Vitvi017521	-3.404557198	8.4E-33	2.81E-31	DOWN
Vitvi003415	1.039986205	0.00000392	0.0000177	UP	Vitvi000250	-3.413903246	0.005585643	0.014864673	DOWN
Vitvi016134	1.039811134	1.22E-23	2.41E-22	UP	Vitvi036394	-3.415380212	1.04E-14	1.1E-13	DOWN

ID	log2FoldChange	pvalue	padj	change	ID	log2FoldChange	pvalue	padj	change
Vitvi036795	1.038874452	0.012213436	0.030084806	UP	Vitvi011712	-3.464474769	1.28E-10	9.5E-10	DOWN
Vitvi008777	1.03870314	2.15E-15	2.4E-14	UP	Vitvi008829	-3.474242279	0.010864855	0.027128081	DOWN
Vitvi002906	1.037446093	7.81E-20	1.23E-18	UP	Vitvi033555	-3.487934349	0.000186075	0.000656972	DOWN
Vitvi002447	1.037319911	6.62E-12	5.56E-11	UP	Vitvi006633	-3.539533638	0.003703039	0.010279639	DOWN
Vitvi011523	1.037285761	1.15E-09	7.83E-09	UP	Vitvi010830	-3.591454209	0.002750893	0.007841165	DOWN
Vitvi010628	1.037062918	0.001755344	0.005226407	UP	Vitvi029092	-3.59407976	2.66E-23	5.15E-22	DOWN
Vitvi011782	1.03700225	0.00000102	0.00000497	UP	Vitvi018586	-3.595568949	0.005872814	0.015564795	DOWN
Vitvi000825	1.036528878	1.23E-08	7.46E-08	UP	Vitvi003636	-3.617626626	0.006047899	0.015987106	DOWN
Vitvi014787	1.036320638	5.49E-26	1.25E-24	UP	Vitvi006186	-3.637281171	0.0000298	0.00011887	DOWN
Vitvi014775	1.035887969	1.53E-13	1.49E-12	UP	Vitvi011488	-3.641451461	0.002591185	0.007422388	DOWN
Vitvi008036	1.035769703	0.00000437	0.0000196	UP	Vitvi003615	-3.668471613	0.017930205	0.042128205	DOWN
Vitvi006846	1.035455977	5.47E-15	5.93E-14	UP	Vitvi000130	-3.672891358	0.005479679	0.014617826	DOWN
Vitvi020372	1.034305558	2.34E-35	8.81E-34	UP	Vitvi010623	-3.684867544	0.0147616	0.035483743	DOWN
Vitvi012478	1.033809443	0.002987048	0.040272384	UP	Vitvi005501	-3.685996553	0.005438094	0.014524467	DOWN
Vitvi017981	1.033107161	9.36E-09	5.77E-08	UP	Vitvi016170	-3.691008139	0.017066234	0.04037849	DOWN
Vitvi019829	1.032316127	1.53E-22	2.84E-21	UP	Vitvi013653	-3.698774044	4.82E-08	0.000000276	DOWN
Vitvi021410	1.032064414	4.47E-16	5.24E-15	UP	Vitvi012036	-3.717066446	0.020288525	0.046893551	DOWN
Vitvi032285	1.031677703	0.000376526	0.008031036	UP	Vitvi026503	-3.717283285	0.0000113	0.0000477	DOWN
Vitvi016847	1.030639834	1.35E-17	1.78E-16	UP	Vitvi004969	-3.766031879	7.33E-11	5.55E-10	DOWN
Vitvi019721	1.029791813	8.86E-12	7.36E-11	UP	Vitvi007225	-3.798365556	0.002965346	0.008392379	DOWN
Vitvi018012	1.029514899	1.18E-10	8.79E-10	UP	Vitvi011380	-3.817452557	1.61E-09	1.08E-08	DOWN
Vitvi002915	1.028147774	0.000000181	0.000000971	UP	Vitvi011888	-3.83480783	0.007886574	0.020328182	DOWN
Vitvi016252	1.028006351	0.000116635	0.000424667	UP	Vitvi004006	-4.101270857	0.002867499	0.008138137	DOWN
Vitvi035553	1.027826792	0.009080722	0.02312823	UP	Vitvi033598	-4.113760845	0.000836279	0.002648759	DOWN
Vitvi031864	1.027814359	0.006148673	0.016207092	UP	Vitvi025531	-4.168784483	0.006502325	0.017066188	DOWN
Vitvi004075	1.026455503	0.002109708	0.006169362	UP	Vitvi036052	-4.189613542	3.84E-47	2.56E-45	DOWN
Vitvi034110	1.026062304	4.4E-10	3.11E-09	UP	Vitvi037337	-4.336283406	0.000459166	0.001520379	DOWN
Vitvi020090	1.025453426	1.02E-08	6.28E-08	UP	Vitvi008527	-4.543423315	0.000147663	0.000528891	DOWN
Vitvi008554	1.024805013	0.0000544	0.000208321	UP	Vitvi009282	-4.591963288	0.000412062	0.001375482	DOWN
Vitvi035961	1.024485274	0.001985227	0.005830528	UP	Vitvi003620	-4.743934437	0.000245929	0.000852298	DOWN
Vitvi025037	1.024202042	0.000107496	0.000394127	UP	Vitvi035489	-5.339627474	0.0000146	0.0000611	DOWN
Vitvi003012	1.023614425	3.19E-09	2.08E-08	UP	Vitvi004010	-5.497268679	6.15E-08	0.000000347	DOWN
Vitvi017970	1.023009757	0.000110891	0.000405913	UP					
Vitvi020095	1.022912112	1.74E-12	1.55E-11	UP					
Vitvi003910	1.022699037	1.98E-13	1.9E-12	UP					
Vitvi024378	1.022498426	2.1E-18	2.93E-17	UP					
Vitvi009805	1.021869018	0.000000183	0.000000977	UP					
Vitvi033567	1.021394574	6.15E-35	2.26E-33	UP					
Vitvi011550	1.021285779	0.000158037	0.000563758	UP					
Vitvi036207	1.018545406	2.85E-11	2.25E-10	UP					
Vitvi010128	1.017859163	2.86E-16	3.44E-15	UP					
Vitvi032737	1.017341091	0.001021451	0.003185584	UP					
Vitvi032152	1.016783828	4.76E-10	3.35E-09	UP					
Vitvi002121	1.016150376	9.59E-11	7.18E-10	UP					
Vitvi002949	1.015997097	0.0000835	0.000311112	UP					
Vitvi022613	1.015921447	0.000118053	0.000429343	UP					
Vitvi012987	1.015451252	1.52E-58	1.71E-56	UP					
Vitvi035051	1.014705293	0.000453608	0.009356802	UP					
Vitvi027469	1.013965267	0.000201005	0.004887048	UP					
Vitvi005574	1.013392289	4.62E-18	6.31E-17	UP					
Vitvi006567	1.0131701	2.82E-11	2.23E-10	UP					
Vitvi019710	1.013072002	6.59E-28	1.68E-26	UP					
Vitvi027550	1.012370461	1.2E-10	8.93E-10	UP					
Vitvi031296	1.012291214	6.33E-17	7.97E-16	UP					
Vitvi006991	1.011825542	7.21E-18	9.68E-17	UP					
Vitvi031917	1.011693779	1.45E-38	6.26E-37	UP					
Vitvi023818	1.010741531	0.0000934	0.000345588	UP					

ID	log2FoldChange	pvalue	padj	change	ID	log2FoldChange	pvalue	padj	change
Vitvi005395	1.009924289	7.55E-08	0.000000422	UP					
Vitvi035588	1.009707221	3.12E-10	2.24E-09	UP					
Vitvi014602	1.008265491	5.19E-40	2.38E-38	UP					
Vitvi003073	1.008110205	7.8E-19	1.12E-17	UP					
Vitvi018645	1.007861775	2.2E-38	9.46E-37	UP					
Vitvi031528	1.007038214	1.76E-21	3.07E-20	UP					
Vitvi026793	1.004876427	4.99E-18	6.79E-17	UP					
Vitvi025021	1.004640266	8.11E-17	1.01E-15	UP					
Vitvi034498	1.003166513	2.73E-11	2.17E-10	UP					
Vitvi002610	1.003122183	0.000323825	0.001099921	UP					
Vitvi029014	1.0030429	3E-28	7.76E-27	UP					
Vitvi019665	1.002055921	0.0000606	0.00023042	UP					
Vitvi021213	1.001669598	6.49E-15	6.99E-14	UP					
Vitvi023229	1.001601267	2.81E-14	2.9E-13	UP					
Vitvi033168	1.000605316	0.01423801	0.034416573	UP					
Vitvi030115	1.00024561	1.44E-25	3.21E-24	UP					

Table. S.11. GO enrichment of differentially expressed gene sets in transcriptome analysis

Funtional Annotation Clustering

Annotatior Enrichment Score: 21.97085985185348

Category	Term	Count	%	PValue	Genes	List Total	Pop Hits	Pop Total	Fold Enrichmen	Bonferroni	Benjamini	FDR
GOTERM	GO:0004712~protein serine/threonine/tyrosine kinase activity	164	6.25	4.82E-29	Q9LVL5	1714	742	19438	2.506574995	4.74E-26	4.75E-26	4.64E-26
GOTERM	GO:0006468~protein phosphorylation	169	6.44054878	1.26E-26	Q9LVL5	1857	897	23397	2.373791295	2.33E-23	2.33E-23	2.28E-23
GOTERM	GO:0004674~protein serine/threonine kinase activity	161	6.135670732	5.16E-21	Q9LVL5	1714	852	19438	2.143024033	5.08E-18	2.45E-18	2.39E-18
GOTERM	GO:0005524~ATP binding	357	13.60518293	7.46E-21	Q9SCJ4,	1714	2552	19438	1.586457004	7.34E-18	2.45E-18	2.39E-18
GOTERM	GO:0004672~protein kinase activity	134	5.106707317	5.97E-16	Q9LVL5	1714	738	19438	2.059155749	5.46E-13	1.47E-13	1.43E-13

Annotatior Enrichment Score: 9.401235452401474

Category	Term	Count	%	PValue	Genes	List Total	Pop Hits	Pop Total	Fold Enrichmen	Bonferroni	Benjamini	FDR
GOTERM	GO:0008017~microtubule binding	47	1.791158537	1.96E-11	Q9SCJ4,	1714	179	19438	2.977731857	1.93E-08	3.85E-09	3.76E-09
GOTERM	GO:0005874~microtubule	45	1.714939024	1.00E-10	Q9SCJ4,	1899	207	25998	2.976165945	3.16E-08	3.52E-09	3.13E-09
GOTERM	GO:0007018~microtubule-based movement	25	0.952743902	1.14E-10	Q9SCJ4,	1857	67	23397	4.701251417	2.10E-07	5.25E-08	5.14E-08
GOTERM	GO:0003777~microtubule motor activity	24	0.914634146	2.82E-09	Q8VWI7	1714	65	19438	4.187344045	2.77E-06	4.63E-07	4.52E-07
GOTERM	GO:0005871~kinesin complex	15	0.571646341	1.57E-08	Q8VWI7	1899	32	25998	6.41735782	4.95E-06	3.81E-07	3.38E-07

Annotatior Enrichment Score: 3.3679229689861967

Category	Term	Count	%	PValue	Genes	List Total	Pop Hits	Pop Total	Fold Enrichmen	Bonferroni	Benjamini	FDR
GOTERM	GO:0009523~photosystem II	15	0.571646341	1.47E-05	O49668,	1899	52	25998	3.949143274	0.004621601	2.11E-04	1.87E-04
GOTERM	GO:0018298~protein-chromophore linkage	14	0.533536585	2.39E-04	O64511,	1857	54	23397	3.266499132	0.356512399	0.016310125	0.015964796
GOTERM	GO:0009765~photosynthesis, light harvesting	9	0.342987805	4.90E-04	Q8LCQ4	1857	25	23397	4.535767367	0.594626522	0.025062097	0.024531467
GOTERM	GO:0009522~photosystem I	10	0.381097561	5.68E-04	Q8LCQ4	1899	34	25998	4.026577456	0.164380509	0.006190721	0.00550504
GOTERM	GO:0016168~chlorophyll binding	11	0.419207317	8.27E-04	Q8LCQ4	1714	36	19438	3.465221055	0.556767314	0.042850033	0.041849474
GOTERM	GO:0009768~photosynthesis, light harvesting in photosystem I	7	0.266768293	0.007689312	Q8LCQ4	1857	23	23397	3.834585938	0.999999337	0.224820825	0.220060775

Annotatior Enrichment Score: 3.347070645737774

Category	Term	Count	%	PValue	Genes	List Total	Pop Hits	Pop Total	Fold Enrichmen	Bonferroni	Benjamini	FDR
GOTERM	GO:0009654~photosystem II oxygen evolving complex	10	0.381097561	5.76E-05	P82538,	1899	26	25998	5.265524365	0.018052784	7.59E-04	6.75E-04
GOTERM	GO:0019898~extrinsic component of membrane	12	0.457317073	5.54E-04	P82538,	1899	48	25998	3.422590837	0.160533255	0.006190721	0.00550504
GOTERM	GO:0015979~photosynthesis	24	0.914634146	0.00284965	P82538,	1857	156	23397	1.938362123	0.994801592	0.10712359	0.104855501

Annotatior Enrichment Score: 1.9293907194788256

Category	Term	Count	%	PValue	Genes	List Total	Pop Hits	Pop Total	Fold Enrichmen	Bonferroni	Benjamini	FDR
GOTERM	GO:0060866~leaf abscission	5	0.19054878	0.002129646	Q9XF23,	1857	8	23397	7.874596123	0.980339096	0.081725172	0.079994834
GOTERM	GO:0050829~defense response to Gram-negative bacterium	7	0.266768293	0.0095769	Q38834,	1857	24	23397	3.674811524	0.99999998	0.248713409	0.24344749
GOTERM	GO:0009862~systemic acquired resistance, salicylic acid mediate	5	0.19054878	0.079853776	Q9XF23,	1857	21	23397	2.999846142	1	0.865239147	0.846919751

Annotatior Enrichment Score: 1.65142403025676

Category	Term	Count	%	PValue	Genes	List Total	Pop Hits	Pop Total	Fold Enrichmen	Bonferroni	Benjamini	FDR
GOTERM	GO:0009231~riboflavin biosynthetic process	7	0.266768293	0.001992161	Q84MD8	1857	18	23397	4.899748699	0.974656054	0.079773069	0.078084063
GOTERM	GO:0008703~5-amino-6-(5-phosphoribosylamino)uracil reducta:	4	0.152439024	0.002552909	Q45GQ7	1714	4	19438	11.34072345	0.919159272	0.104775637	0.102329099
GOTERM	GO:0005980~glycogen catabolic process	4	0.152439024	0.004410883	Q45GQ7	1857	5	23397	10.07948304	0.999710466	0.145900687	0.142811585
GOTERM	GO:0008184~glycogen phosphorylase activity	4	0.152439024	0.005963078	Q45GQ7	1714	5	19438	9.072578763	0.997219803	0.187727546	0.18334406
GOTERM	GO:0004645~phosphorylase activity	4	0.152439024	0.005963078	Q45GQ7	1714	5	19438	9.072578763	0.997219803	0.187727546	0.18334406
GOTERM	GO:0007129~synapsis	7	0.266768293	0.007689312	Q9FX64,	1857	23	23397	3.834585938	0.999999337	0.224820825	0.220060775
GOTERM	GO:0008835~diaminohydroxyphosphoribosylaminopyrimidine d	4	0.152439024	0.011146566	Q45GQ7	1714	6	19438	7.560482303	0.999983789	0.322922563	0.315382239
GOTERM	GO:0008138~protein tyrosine/serine/threonine phosphatase activ	6	0.228658537	0.073321624	Q9LT75,	1714	26	19438	2.617090028	1	0.98492081	0.961922659
GOTERM	GO:0004725~protein tyrosine phosphatase activity	7	0.266768293	0.073994051	Q9LT75,	1714	34	19438	2.334854829	1	0.98492081	0.961922659
GOTERM	GO:0050661~NADP binding	11	0.419207317	0.099371687	A8MRX(	1714	72	19438	1.732610528	1	1	0.977642276
GOTERM	GO:0010119~regulation of stomatal movement	10	0.381097561	0.107977103	Q8LBL1	1857	71	23397	1.774556873	1	0.974969727	0.954327046
GOTERM	GO:0043622~cortical microtubule organization	8	0.304878049	0.163399151	Q84WL6	1857	57	23397	1.768330357	1	1	0.979359044
GOTERM	GO:0004721~phosphoprotein phosphatase activity	6	0.228658537	0.52282907	Q9M1S3	1714	54	19438	1.260080384	1	1	0.977642276

Annotatior Enrichment Score: 1.628205667750791

Category	Term	Count	%	PValue	Genes	List Total	Pop Hits	Pop Total	Fold Enrichmen	Bonferroni	Benjamini	FDR
GOTERM	GO:0000307~cyclin-dependent protein kinase holoenzyme compl	11	0.419207317	0.007697415	Q9SN11,	1899	57	25998	2.641999945	0.912995286	0.064010082	0.056920358
GOTERM	GO:0044772~mitotic cell cycle phase transition	8	0.304878049	0.013365789	Q9SN11,	1857	33	23397	3.054388799	1	0.332699764	0.325655632
GOTERM	GO:0000079~regulation of cyclin-dependent protein serine/threo	9	0.342987805	0.033756298	Q9SN11,	1857	48	23397	2.362378837	1	0.577314367	0.565091098
GOTERM	GO:0016538~cyclin-dependent protein serine/threonine kinase re	8	0.304878049	0.088406263	Q9SN11,	1714	44	19438	2.061949719	1	1	0.977642276

Annotatior Enrichment Score: 1.5703788452050662

Category	Term	Count	%	PValue	Genes	List Total	Pop Hits	Pop Total	Fold Enrichmen	Bonferroni	Benjamini	FDR
GOTERM	GO:0010020~chloroplast fission	8	0.304878049	0.003348299	P52410,	1857	26	23397	3.876724245	0.997932198	0.120932679	0.118372215
GOTERM	GO:0006182~cGMP biosynthetic process	3	0.114329268	0.017871807	Q9SJU9	1857	3	23397	12.5993538	1	0.401461806	0.392961801
GOTERM	GO:0004383~guanylate cyclase activity	4	0.152439024	0.018237583	Q9SJU9,	1714	7	19438	6.480413402	0.999999986	0.485514033	0.474177157
GOTERM	GO:0000301~retrograde transport, vesicle recycling within Golgi	3	0.114329268	0.033868322	Q9SJU9	1857	4	23397	9.449515347	1	0.577314367	0.565091098
GOTERM	GO:0031985~Golgi cisterna	3	0.114329268	0.045918033	Q9SJU9	1899	5	25998	8.214218009	0.999999646	0.279040355	0.248133986
GOTERM	GO:0007030~Golgi organization	6	0.228658537	0.222838388	Q9SJU9,	1857	41	23397	1.843807873	1	1	0.979359044

Annotatior Enrichment Score: 1.3850244022246574

Category	Term	Count	%	PValue	Genes	List Total	Pop Hits	Pop Total	Fold Enrichmen	Bonferroni	Benjamini	FDR
GOTERM	GO:0015301~anion:anion antiporter activity	7	0.266768293	0.004607912	Q9FEP7,	1714	19	19438	4.178161272	0.989376256	0.161810055	0.158031749
GOTERM	GO:0008271~secondary active sulfate transmembrane transporte	4	0.152439024	0.082328643	Q9FEP7,	1714	12	19438	3.780241151	1	1	0.977642276
GOTERM	GO:0015116~sulfate transmembrane transporter activity	4	0.152439024	0.184447043	Q9FEP7,	1714	17	19438	2.668405519	1	1	0.977642276

Annotatior Enrichment Score: 1.3542714244582472

Category	Term	Count	%	PValue	Genes	List Total	Pop Hits	Pop Total	Fold Enrichmen	Bonferroni	Benjamini	FDR
GOTERM	GO:0071486~cellular response to high light intensity	6	0.228658537	3.00E-04	Q9SJ02,	1857	9	23397	8.399569198	0.424802689	0.019057212	0.01865372
GOTERM	GO:0071492~cellular response to UV-A	4	0.152439024	0.008302106	Q9SJ02,	1857	6	23397	8.399569198	0.999999788	0.23170422	0.226798431
GOTERM	GO:0048481~plant ovule development	9	0.342987805	0.14128791	Q94F62,	1857	65	23397	1.74452591	1	1	0.979359044
GOTERM	GO:0000977~RNA polymerase II regulatory region sequence-spe	8	0.304878049	0.495184835	Q9SJ02,	1714	75	19438	1.209677168	1	1	0.977642276
GOTERM	GO:0045944~positive regulation of transcription from RNA poly	8	0.304878049	0.9714368	Q9SJ02,	1857	158	23397	0.637941964	1	1	0.979359044

Annotatior Enrichment Score: 1.2586085979750805

Category	Term	Count	%	PValue	Genes	List Total	Pop Hits	Pop Total	Fold Enrichmen	Bonferroni	Benjamini	FDR
GOTERM	GO:0015301~anion:anion antiporter activity	7	0.266768293	0.004607912	Q9FEP7,	1714	19	19438	4.178161272	0.989376256	0.161810055	0.158031749
GOTERM	GO:0006820~anion transport	5	0.19054878	0.049220397	Q9LYG9	1857	18	23397	3.499820499	1	0.729305513	0.713864191
GOTERM	GO:0005452~inorganic anion exchanger activity	3	0.114329268	0.12105692	Q8VYR7	1714	7	19438	4.860310052	1	1	0.977642276
GOTERM	GO:0050801~ion homeostasis	3	0.114329268	0.336454804	Q8VYR7	1857	15	23397	2.519870759	1	1	0.979359044

Annotatior Enrichment Score: 1.2390998740255994

Category	Term	Count	%	PValue	Genes	List Total	Pop Hits	Pop Total	Fold Enrichmen	Bonferroni	Benjamini	FDR
GOTERM	GO:0006949~syncytium formation	6	0.228658537	0.004776035	Q38865,	1857	15	23397	5.039741519	0.999852741	0.151680268	0.148468797
GOTERM	GO:0009828~plant-type cell wall loosening	6	0.228658537	0.166331293	Q38865,	1857	37	23397	2.043138453	1	1	0.979359044
GOTERM	GO:0009664~plant-type cell wall organization	10	0.381097561	0.241356478	Q38865,	1857	86	23397	1.465041139	1	1	0.979359044

Annotatior Enrichment Score: 1.2111021330085678

Category	Term	Count	%	PValue	Genes	List Total	Pop Hits	Pop Total	Fold Enrichmen	Bonferroni	Benjamini	FDR
GOTERM	GO:0010052~guard cell differentiation	5	0.19054878	0.040762538	Q9M8K6	1857	17	23397	3.705692293	1	0.652909514	0.639085697
GOTERM	GO:0010374~stomatal complex development	8	0.304878049	0.056462414	Q9M8K6	1857	44	23397	2.290791599	1	0.78172648	0.765175267
GOTERM	GO:2000122~negative regulation of stomatal complex developm	3	0.114329268	0.101081795	Q1PEY6	1857	7	23397	5.399723056	1	0.945140436	0.925129319

Annotatior Enrichment Score: 1.1927814655723645

Category	Term	Count	%	PValue	Genes	List Total	Pop Hits	Pop Total	Fold Enrichmen	Bonferroni	Benjamini	FDR
GOTERM	GO:0018171~peptidyl-cysteine oxidation	3	0.114329268	0.053506615	Q1G3U6	1857	5	23397	7.559612278	1	0.758147569	0.742095584
GOTERM	GO:0017172~cysteine dioxygenase activity	3	0.114329268	0.0648355	Q1G3U6	1714	5	19438	6.804434072	1	0.886985655	0.866274315
GOTERM	GO:0070483~detection of hypoxia	3	0.114329268	0.076108869	Q1G3U6	1857	6	23397	6.299676898	1	0.854832541	0.83673348

Annotatior Enrichment Score: 1.176889869191389

Category	Term	Count	%	PValue	Genes	List Total	Pop Hits	Pop Total	Fold Enrichmen	Bonferroni	Benjamini	FDR
GOTERM	GO:0050136~NADH dehydrogenase (quinone) activity	5	0.19054878	0.029339857	Q94BV7	1714	14	19438	4.050258376	1	0.628255626	0.613585697
GOTERM	GO:0010181~FMN binding	7	0.266768293	0.082962682	Q6NQE2	1714	35	19438	2.268144691	1	1	0.977642276
GOTERM	GO:0008753~NADPH dehydrogenase (quinone) activity	3	0.114329268	0.12105692	Q6NQE2	1714	7	19438	4.860310052	1	1	0.977642276

Annotatior Enrichment Score: 1.1614559374128253

Category	Term	Count	%	PValue	Genes	List Total	Pop Hits	Pop Total	Fold Enrichmen	Bonferroni	Benjamini	FDR
GOTERM	GO:0080085~signal recognition particle, chloroplast targeting	3	0.114329268	0.015204577	O22265,	1899	3	25998	13.69036335	0.992105169	0.111735958	0.09936014
GOTERM	GO:0045038~protein import into chloroplast thylakoid membran	4	0.152439024	0.0206047	O22265,	1857	8	23397	6.299676898	1	0.436251232	0.427014642
GOTERM	GO:0070208~protein heterotrimerization	3	0.114329268	0.033868322	O22265,	1857	4	23397	9.449515347	1	0.577314367	0.565091098
GOTERM	GO:0005786~signal recognition particle, endoplasmic reticulum t	3	0.114329268	0.244593952	O22265,	1899	13	25998	3.159314619	1	0.950124778	0.844889439
GOTERM	GO:0032991~macromolecular complex	4	0.152439024	0.600537732	Q94F62,	1899	42	25998	1.303844128	1	1	0.892063492

Annotatior Enrichment Score: 1.1390616684855206

Category	Term	Count	%	PValue	Genes	List Total	Pop Hits	Pop Total	Fold Enrichmen	Bonferroni	Benjamini	FDR
GOTERM	GO:0042910~xenobiotic transporter activity	10	0.381097561	0.060312753	O82752,	1714	57	19438	1.989600606	1	0.886985655	0.866274315
GOTERM	GO:0015297~antiporter activity	18	0.68597561	0.064572355	Q9FHB6	1714	130	19438	1.570254017	1	0.886985655	0.866274315
GOTERM	GO:1990961~drug transmembrane export	8	0.304878049	0.09825603	O82752,	1857	50	23397	2.015896607	1	0.945140436	0.925129319

Annotatior Enrichment Score: 1.0314167567638897

Category	Term	Count	%	PValue	Genes	List Total	Pop Hits	Pop Total	Fold Enrichmen	Bonferroni	Benjamini	FDR
GOTERM	GO:0019222~regulation of metabolic process	5	0.19054878	0.005615019	Q9FZ99,	1857	10	23397	6.299676898	0.999968879	0.172381074	0.168731312
GOTERM	GO:0034613~cellular protein localization	6	0.228658537	0.094838006	O81001,	1857	31	23397	2.438584606	1	0.919429511	0.899962763
GOTERM	GO:0099402~plant organ development	6	0.228658537	0.364169063	Q38997,	1857	50	23397	1.511922456	1	1	0.979359044
GOTERM	GO:0045309~protein phosphorylated amino acid binding	3	0.114329268	0.386096889	O81001,	1714	15	19438	2.268144691	1	1	0.977642276

Annotatior Enrichment Score: 1.0250962531836847

Category	Term	Count	%	PValue	Genes	List Total	Pop Hits	Pop Total	Fold Enrichmen	Bonferroni	Benjamini	FDR
GOTERM	GO:0009755~hormone-mediated signaling pathway	34	1.295731707	0.029913514	Q9FLF4,	1857	296	23397	1.447223071	1	0.568048384	0.556021301
GOTERM	GO:1901701~cellular response to oxygen-containing compound	48	1.829268293	0.086986252	Q9FKH6	1857	484	23397	1.249522691	1	0.900161098	0.881102313
GOTERM	GO:0071396~cellular response to lipid	29	1.105182927	0.323141834	Q9FLF4,	1857	320	23397	1.141816438	1	1	0.979359044

Annotatior Enrichment Score: 1.0192224058644324

Category	Term	Count	%	PValue	Genes	List Total	Pop Hits	Pop Total	Fold Enrichmen	Bonferroni	Benjamini	FDR
GOTERM	GO:0006531~aspartate metabolic process	3	0.114329268	0.076108869	P46643,	1857	6	23397	6.299676898	1	0.854832541	0.83673348
GOTERM	GO:0006103~2-oxoglutarate metabolic process	3	0.114329268	0.076108869	P46643,	1857	6	23397	6.299676898	1	0.854832541	0.83673348
GOTERM	GO:0006536~glutamate metabolic process	4	0.152439024	0.078222899	Q42521,	1857	13	23397	3.876724245	1	0.865239147	0.846919751
GOTERM	GO:0004069~L-aspartate:2-oxoglutarate aminotransferase activi	3	0.114329268	0.184886811	P46643,	1714	9	19438	3.780241151	1	1	0.977642276

Annotatior Enrichment Score: 0.9375750145463615

Category	Term	Count	%	PValue	Genes	List Total	Pop Hits	Pop Total	Fold Enrichmen	Bonferroni	Benjamini	FDR
GOTERM	GO:0000234~phosphoethanolamine N-methyltransferase activity	3	0.114329268	0.0648355	Q944H0,	1714	5	19438	6.804434072	1	0.886985655	0.866274315
GOTERM	GO:0052667~phosphomethylethanolamine N-methyltransferase a	3	0.114329268	0.0648355	Q944H0,	1714	5	19438	6.804434072	1	0.886985655	0.866274315
GOTERM	GO:0006656~phosphatidylcholine biosynthetic process	3	0.114329268	0.366141733	Q944H0,	1857	16	23397	2.362378837	1	1	0.979359044

Annotatior Enrichment Score: 0.91165573193179

Category	Term	Count	%	PValue	Genes	List Total	Pop Hits	Pop Total	Fold Enrichmen	Bonferroni	Benjamini	FDR
GOTERM	GO:0005262~calcium channel activity	8	0.304878049	0.051709059	Q84W41	1714	39	19438	2.326302247	1	0.834974154	0.815477296
GOTERM	GO:0071230~cellular response to amino acid stimulus	5	0.19054878	0.091744928	Q84W41	1857	22	23397	2.863489499	1	0.919429511	0.899962763
GOTERM	GO:0015276~ligand-gated ion channel activity	5	0.19054878	0.093461692	Q84W41	1714	20	19438	2.835180863	1	1	0.977642276
GOTERM	GO:0005217~intracellular ligand-gated ion channel activity	5	0.19054878	0.093461692	Q84W41	1714	20	19438	2.835180863	1	1	0.977642276
GOTERM	GO:0008066~glutamate receptor activity	5	0.19054878	0.093461692	Q84W41	1714	20	19438	2.835180863	1	1	0.977642276
GOTERM	GO:0006816~calcium ion transport	6	0.228658537	0.153189789	Q84W41	1857	36	23397	2.099892299	1	1	0.979359044
GOTERM	GO:0006874~cellular calcium ion homeostasis	6	0.228658537	0.166331293	Q84W41	1857	37	23397	2.043138453	1	1	0.979359044
GOTERM	GO:0038023~signaling receptor activity	7	0.266768293	0.18771931	Q84W41	1714	44	19438	1.804206004	1	1	0.977642276
GOTERM	GO:0019722~calcium-mediated signaling	9	0.342987805	0.336772065	Q84W41	1857	83	23397	1.36619499	1	1	0.979359044

Annotatior Enrichment Score: 0.909035872556212

Category	Term	Count	%	PValue	Genes	List Total	Pop Hits	Pop Total	Fold Enrichmen	Bonferroni	Benjamini	FDR
GOTERM	GO:0030322~stabilization of membrane potential	3	0.114329268	0.101081795	Q8LBL1	1857	7	23397	5.399723056	1	0.945140436	0.925129319
GOTERM	GO:0022841~potassium ion leak channel activity	3	0.114329268	0.12105692	Q8LBL1	1714	7	19438	4.860310052	1	1	0.977642276
GOTERM	GO:0071805~potassium ion transmembrane transport	6	0.228658537	0.153189789	Q84TI7,	1857	36	23397	2.099892299	1	1	0.979359044

Annotatior Enrichment Score: 0.8884754741498356

Category	Term	Count	%	PValue	Genes	List Total	Pop Hits	Pop Total	Fold Enrichmen	Bonferroni	Benjamini	FDR
GOTERM	GO:0005093~Rab GDP-dissociation inhibitor activity	3	0.114329268	0.041286531	Q9SFC6,	1714	4	19438	8.50554259	1	0.72620059	0.709243622
GOTERM	GO:0007264~small GTPase mediated signal transduction	5	0.19054878	0.068784369	O82480,	1857	20	23397	3.149838449	1	0.854832541	0.83673348
GOTERM	GO:0005096~GTPase activator activity	7	0.266768293	0.760808626	Q9LVH4	1714	84	19438	0.945060288	1	1	0.977642276

Annotatior Enrichment Score: 0.8738880525505944

Category	Term	Count	%	PValue	Genes	List Total	Pop Hits	Pop Total	Fold Enrichmen	Bonferroni	Benjamini	FDR
GOTERM	GO:0019829~cation-transporting ATPase activity	7	0.266768293	0.027419632	Q9LY77	1714	27	19438	2.940187562	1	0.613825849	0.59949286
GOTERM	GO:0005388~calcium-transporting ATPase activity	6	0.228658537	0.03920467	Q9LY77	1714	22	19438	3.092924578	1	0.72620059	0.709243622
GOTERM	GO:0016836~hydro-lyase activity	5	0.19054878	0.139118816	Q9SIB9,	1714	23	19438	2.465374664	1	1	0.977642276
GOTERM	GO:0070588~calcium ion transmembrane transport	6	0.228658537	0.179898181	Q9L636,	1857	38	23397	1.989371652	1	1	0.979359044
GOTERM	GO:0004089~carbonate dehydratase activity	4	0.152439024	0.330749954	O22218,	1714	23	19438	1.972299731	1	1	0.977642276
GOTERM	GO:0006730~one-carbon metabolic process	4	0.152439024	0.641741067	O22218,	1857	41	23397	1.229205248	1	1	0.979359044

Annotatior Enrichment Score: 0.7831554734670197

Category	Term	Count	%	PValue	Genes	List Total	Pop Hits	Pop Total	Fold Enrichmen	Bonferroni	Benjamini	FDR
GOTERM	GO:0047780~citrate dehydratase activity	3	0.114329268	0.0648355	Q9SIB9,	1714	5	19438	6.804434072	1	0.886985655	0.866274315
GOTERM	GO:0003994~aconitate hydratase activity	3	0.114329268	0.091679967	Q9SIB9,	1714	6	19438	5.670361727	1	1	0.977642276
GOTERM	GO:0006101~citrate metabolic process	3	0.114329268	0.156135239	Q9SIB9,	1857	9	23397	4.199784599	1	1	0.979359044
GOTERM	GO:0006102~isocitrate metabolic process	3	0.114329268	0.306360798	Q9SIB9,	1857	14	23397	2.699861528	1	1	0.979359044
GOTERM	GO:0006099~tricarboxylic acid cycle	7	0.266768293	0.426973773	Q9SN86,	1857	66	23397	1.3362951	1	1	0.979359044

Annotatio Enrichment Score: 0.7574661845073845

Category	Term	Count	%	PValue	Genes	List Total	Pop Hits	Pop Total	Fold Enrichmen	Bonferroni	Benjamini	FDR
GOTERM	GO:0000103~sulfate assimilation	5	0.19054878	0.131973438	Q9SRW7	1857	25	23397	2.519870759	1	1	0.979359044
GOTERM	GO:0070814~hydrogen sulfide biosynthetic process	3	0.114329268	0.185374093	Q9SRW7	1857	10	23397	3.779806139	1	1	0.979359044
GOTERM	GO:0004020~adenylylsulfate kinase activity	3	0.114329268	0.218306102	Q9SRW7	1714	10	19438	3.402217036	1	1	0.977642276

Annotatio Enrichment Score: 0.7527827444890541

Category	Term	Count	%	PValue	Genes	List Total	Pop Hits	Pop Total	Fold Enrichmen	Bonferroni	Benjamini	FDR
GOTERM	GO:0034990~nuclear mitotic cohesin complex	3	0.114329268	0.087479813	Q9FQ19,	1899	7	25998	5.867298578	1	0.468535948	0.416641144
GOTERM	GO:0008278~cohesin complex	3	0.114329268	0.136232694	Q9FQ19,	1899	9	25998	4.56345445	1	0.662300481	0.588944415
GOTERM	GO:0007062~sister chromatid cohesion	4	0.152439024	0.147478544	Q9FQ19,	1857	17	23397	2.964553834	1	1	0.979359044
GOTERM	GO:0007064~mitotic sister chromatid cohesion	3	0.114329268	0.554563128	Q9FQ19,	1857	23	23397	1.643393973	1	1	0.979359044

Annotatio Enrichment Score: 0.7427137320698703

Category	Term	Count	%	PValue	Genes	List Total	Pop Hits	Pop Total	Fold Enrichmen	Bonferroni	Benjamini	FDR
GOTERM	GO:0004048~anthranilate phosphoribosyltransferase activity	3	0.114329268	0.02191972	Q02166	1714	3	19438	11.34072345	1	0.552350042	0.539452529
GOTERM	GO:0000162~tryptophan biosynthetic process	6	0.228658537	0.075362859	Q42565,	1857	29	23397	2.606762854	1	0.854832541	0.83673348
GOTERM	GO:0000290~deadenylation-dependent decapping of nuclear-tran	3	0.114329268	0.156135239	Q02166	1857	9	23397	4.199784599	1	1	0.979359044
GOTERM	GO:1990110~callus formation	5	0.19054878	0.246253678	Q940D0,	1857	32	23397	1.968649031	1	1	0.979359044
GOTERM	GO:0000932~P-body	8	0.304878049	0.342691755	Q9LUZ4	1899	78	25998	1.404139831	1	1	0.892063492
GOTERM	GO:0033962~cytoplasmic mRNA processing body assembly	3	0.114329268	0.423817656	Q02166	1857	18	23397	2.099892299	1	1	0.979359044
GOTERM	GO:0009585~red, far-red light phototransduction	5	0.19054878	0.685559492	O82239,	1857	58	23397	1.086151189	1	1	0.979359044

Annotatio Enrichment Score: 0.7406306397298437

Category	Term	Count	%	PValue	Genes	List Total	Pop Hits	Pop Total	Fold Enrichmen	Bonferroni	Benjamini	FDR
GOTERM	GO:0009749~response to glucose	8	0.304878049	0.143118746	Q38997,	1857	55	23397	1.832633279	1	1	0.979359044
GOTERM	GO:0009744~response to sucrose	10	0.381097561	0.201114965	Q8LC03,	1857	82	23397	1.536506561	1	1	0.979359044
GOTERM	GO:0009750~response to fructose	4	0.152439024	0.208433332	O80462,	1857	20	23397	2.519870759	1	1	0.979359044

Annotatio Enrichment Score: 0.6861518010096737

Category	Term	Count	%	PValue	Genes	List Total	Pop Hits	Pop Total	Fold Enrichmen	Bonferroni	Benjamini	FDR
GOTERM	GO:0009407~toxin catabolic process	7	0.266768293	0.154455442	Q9FUS8,	1857	46	23397	1.917292969	1	1	0.979359044
GOTERM	GO:0004364~glutathione transferase activity	9	0.342987805	0.211304854	Q9FUS8,	1714	65	19438	1.570254017	1	1	0.977642276
GOTERM	GO:0006749~glutathione metabolic process	8	0.304878049	0.267813157	Q9FUS8,	1857	66	23397	1.5271944	1	1	0.979359044

Annotatio Enrichment Score: 0.6299510260135286

Category	Term	Count	%	PValue	Genes	List Total	Pop Hits	Pop Total	Fold Enrichmen	Bonferroni	Benjamini	FDR
GOTERM	GO:0008878~glucose-1-phosphate adenylyltransferase activity	3	0.114329268	0.152312108	Q9LIK9,	1714	8	19438	4.252771295	1	1	0.977642276
GOTERM	GO:0005978~glycogen biosynthetic process	3	0.114329268	0.21528626	Q9LIK9,	1857	11	23397	3.436187399	1	1	0.979359044
GOTERM	GO:0019252~starch biosynthetic process	5	0.19054878	0.393003286	F4KFB3,	1857	40	23397	1.574919225	1	1	0.979359044

Annotatio Enrichment Score: 0.6087668176443294

Category	Term	Count	%	PValue	Genes	List Total	Pop Hits	Pop Total	Fold Enrichmen	Bonferroni	Benjamini	FDR
GOTERM	GO:0006612~protein targeting to membrane	6	0.228658537	0.153189789	Q9C533,	1857	36	23397	2.099892299	1	1	0.979359044
GOTERM	GO:0018230~peptidyl-L-cysteine S-palmitoylation	4	0.152439024	0.273758717	Q9C533,	1857	23	23397	2.191191965	1	1	0.979359044
GOTERM	GO:0019706~protein-cysteine S-palmitoyltransferase activity	4	0.152439024	0.355714666	Q9C533,	1714	24	19438	1.890120576	1	1	0.977642276

Annotatio Enrichment Score: 0.592526568595606

Category	Term	Count	%	PValue	Genes	List Total	Pop Hits	Pop Total	Fold Enrichmen	Bonferroni	Benjamini	FDR
GOTERM	GO:0043138~3'-5' DNA helicase activity	4	0.152439024	0.119626787	Q0WVW	1714	14	19438	3.240206701	1	1	0.977642276
GOTERM	GO:0032508~DNA duplex unwinding	4	0.152439024	0.296031815	F4KAB8	1857	24	23397	2.099892299	1	1	0.979359044
GOTERM	GO:0003678~DNA helicase activity	6	0.228658537	0.471252285	Q9XFH4	1714	51	19438	1.334202759	1	1	0.977642276

Annotatio Enrichment Score: 0.5599694191189363

Category	Term	Count	%	PValue	Genes	List Total	Pop Hits	Pop Total	Fold Enrichmen	Bonferroni	Benjamini	FDR
GOTERM	GO:0009504~cell plate	9	0.342987805	0.015125328	Q9SU08,	1899	45	25998	2.73807267	0.991901844	0.111735958	0.09936014
GOTERM	GO:0061025~membrane fusion	5	0.19054878	0.194508708	Q9ZPV9	1857	29	23397	2.172302379	1	1	0.979359044
GOTERM	GO:0048278~vesicle docking	4	0.152439024	0.363124161	Q9ZPV9	1857	27	23397	1.866570933	1	1	0.979359044
GOTERM	GO:0031201~SNARE complex	6	0.228658537	0.37416375	Q9ZPV9	1899	55	25998	1.493494184	1	1	0.892063492
GOTERM	GO:0006906~vesicle fusion	5	0.19054878	0.411399491	Q9ZPV9	1857	41	23397	1.536506561	1	1	0.979359044
GOTERM	GO:0005484~SNAP receptor activity	6	0.228658537	0.453656431	Q9ZPV9	1714	50	19438	1.360886814	1	1	0.977642276
GOTERM	GO:0000149~SNARE binding	7	0.266768293	0.560798975	Q9ZPV9	1714	68	19438	1.167427414	1	1	0.977642276
GOTERM	GO:0006887~exocytosis	4	0.152439024	0.791928383	Q9ZPV9	1857	52	23397	0.969181061	1	1	0.979359044

Annotatio Enrichment Score: 0.5409464528152396

Category	Term	Count	%	PValue	Genes	List Total	Pop Hits	Pop Total	Fold Enrichmen	Bonferroni	Benjamini	FDR
GOTERM	GO:0006782~protoporphyrinogen IX biosynthetic process	4	0.152439024	0.167130173	Q93ZB6,	1857	18	23397	2.799856399	1	1	0.979359044
GOTERM	GO:0006783~heme biosynthetic process	3	0.114329268	0.336454804	P55826,	1857	15	23397	2.519870759	1	1	0.979359044
GOTERM	GO:0006779~porphyrin-containing compound biosynthetic proce	3	0.114329268	0.423817656	P55826,	1857	18	23397	2.099892299	1	1	0.979359044

Annotatio Enrichment Score: 0.5259020472309128

Category	Term	Count	%	PValue	Genes	List Total	Pop Hits	Pop Total	Fold Enrichmen	Bonferroni	Benjamini	FDR
GOTERM	GO:0016987~sigma factor activity	3	0.114329268	0.091679967	Q9LD95,	1714	6	19438	5.670361727	1	1	0.977642276
GOTERM	GO:0071482~cellular response to light stimulus	8	0.304878049	0.385113669	Q9FNB0	1857	75	23397	1.343931072	1	1	0.979359044
GOTERM	GO:0006352~DNA-templated transcription, initiation	3	0.114329268	0.748911463	Q9LD95,	1857	33	23397	1.1453958	1	1	0.979359044

Annotatio Enrichment Score: 0.5039693508161092

Category	Term	Count	%	PValue	Genes	List Total	Pop Hits	Pop Total	Fold Enrichmen	Bonferroni	Benjamini	FDR
GOTERM	GO:0004861~cyclin-dependent protein serine/threonine kinase in	3	0.114329268	0.184886811	Q94CL9,	1714	9	19438	3.780241151	1	1	0.977642276
GOTERM	GO:0045736~negative regulation of cyclin-dependent protein ser	3	0.114329268	0.185374093	Q94CL9,	1857	10	23397	3.779806139	1	1	0.979359044
GOTERM	GO:0005654~nucleoplasm	9	0.342987805	0.897711887	Q94CL9,	1899	158	25998	0.779830824	1	1	0.897711887

Annotatio Enrichment Score: 0.5002297925474327

Category	Term	Count	%	PValue	Genes	List Total	Pop Hits	Pop Total	Fold Enrichmen	Bonferroni	Benjamini	FDR
GOTERM	GO:0042162~telomeric DNA binding	4	0.152439024	0.184447043	Q9FZ99,	1714	17	19438	2.668405519	1	1	0.977642276
GOTERM	GO:0000781~chromosome, telomeric region	3	0.114329268	0.244593952	Q9FFY9,	1899	13	25998	3.159314619	1	0.950124778	0.844889439
GOTERM	GO:0000723~telomere maintenance	3	0.114329268	0.699831056	Q9FFY9,	1857	30	23397	1.25993538	1	1	0.979359044

Annotatio Enrichment Score: 0.43308000458450185

Category	Term	Count	%	PValue	Genes	List Total	Pop Hits	Pop Total	Fold Enrichmen	Bonferroni	Benjamini	FDR
GOTERM	GO:0006863~purine nucleobase transport	4	0.152439024	0.229863258	O49725,	1857	21	23397	2.399876914	1	1	0.979359044
GOTERM	GO:0005345~purine nucleobase transmembrane transporter activ	4	0.152439024	0.355714666	O49725,	1714	24	19438	1.890120576	1	1	0.977642276
GOTERM	GO:0015211~purine nucleoside transmembrane transporter activ	3	0.114329268	0.614028679	O49725,	1714	23	19438	1.479224798	1	1	0.977642276

Annotatio Enrichment Score: 0.39598854871337075

Category	Term	Count	%	PValue	Genes	List Total	Pop Hits	Pop Total	Fold Enrichmen	Bonferroni	Benjamini	FDR
GOTERM	GO:0070181~small ribosomal subunit rRNA binding	4	0.152439024	0.140211407	Q9ASV6	1714	15	19438	3.024192921	1	1	0.977642276

GOTERM	GO:0050135~NAD(P)+ nucleosidase activity	4	0.152439024	0.680182576	Q8VY91	1714	39	19438	1.163151123	1	1	0.977642276
GOTERM	GO:0061809~NAD+ nucleotidase, cyclic ADP-ribose generating	4	0.152439024	0.680182576	Q8VY91	1714	39	19438	1.163151123	1	1	0.977642276

Annotatio Enrichment Score: 0.3423329361692113

Category	Term	Count	%	PValue	Genes	List Total	Pop Hits	Pop Total	Fold Enrichmen	Bonferroni	Benjamini	FDR
GOTERM	GO:0080027~response to herbivore	5	0.19054878	0.146754148	O64865,	1857	26	23397	2.422952653	1	1	0.979359044
GOTERM	GO:0016102~diterpenoid biosynthetic process	3	0.114329268	0.763665496	Q93YV0	1857	34	23397	1.111707688	1	1	0.979359044
GOTERM	GO:0010333~terpene synthase activity	3	0.114329268	0.838508827	Q93YV0	1714	36	19438	0.945060288	1	1	0.977642276

Annotatio Enrichment Score: 0.25385846824157365

Category	Term	Count	%	PValue	Genes	List Total	Pop Hits	Pop Total	Fold Enrichmen	Bonferroni	Benjamini	FDR
GOTERM	GO:0051753~mannan synthase activity	4	0.152439024	0.429486599	Q0WVN.	1714	27	19438	1.680107178	1	1	0.977642276
GOTERM	GO:0030244~cellulose biosynthetic process	5	0.19054878	0.483448035	Q0WVN.	1857	45	23397	1.3999282	1	1	0.979359044
GOTERM	GO:0009833~plant-type primary cell wall biogenesis	3	0.114329268	0.662857142	Q0WVN.	1857	28	23397	1.349930764	1	1	0.979359044
GOTERM	GO:0016760~cellulose synthase (UDP-forming) activity	3	0.114329268	0.701207851	Q0WVN.	1714	27	19438	1.260080384	1	1	0.977642276

Annotatio Enrichment Score: 0.2128198092345491

Category	Term	Count	%	PValue	Genes	List Total	Pop Hits	Pop Total	Fold Enrichmen	Bonferroni	Benjamini	FDR
GOTERM	GO:0045490~pectin catabolic process	12	0.457317073	0.184653131	Q9SRX4	1857	102	23397	1.482276917	1	1	0.979359044
GOTERM	GO:0045330~aspartyl esterase activity	7	0.266768293	0.589816942	Q9SRX4	1714	70	19438	1.134072345	1	1	0.977642276
GOTERM	GO:0042545~cell wall modification	7	0.266768293	0.629139626	Q9SRX4	1857	81	23397	1.088833044	1	1	0.979359044
GOTERM	GO:0030599~pectinesterase activity	7	0.266768293	0.846314303	Q9SRX4	1714	94	19438	0.844521959	1	1	0.977642276
GOTERM	GO:0046910~pectinesterase inhibitor activity	6	0.228658537	0.929460661	Q9SRX4	1714	95	19438	0.716256218	1	1	0.977642276
GOTERM	GO:0004857~enzyme inhibitor activity	6	0.228658537	0.980612649	Q9SRX4	1714	117	19438	0.581575562	1	1	0.980612649

Annotatio Enrichment Score: 0.2070060552746151

Category	Term	Count	%	PValue	Genes	List Total	Pop Hits	Pop Total	Fold Enrichmen	Bonferroni	Benjamini	FDR
GOTERM	GO:0019843~rRNA binding	14	0.533536585	0.15457172	Q9ASV6	1714	108	19438	1.470093781	1	1	0.977642276
GOTERM	GO:0005840~ribosome	19	0.724085366	0.961606826	Q9ASV6	1899	353	25998	0.736875081	1	1	0.961606826
GOTERM	GO:0003735~structural constituent of ribosome	21	0.800304878	0.999650535	Q9ASV6	1714	427	19438	0.557740498	1	1	0.999650535
GOTERM	GO:0006412~translation	22	0.838414634	1	Q9ASV6	1857	1030	23397	0.269112411	1	1	1

Annotatio Enrichment Score: 0.20124508014493506

Category	Term	Count	%	PValue	Genes	List Total	Pop Hits	Pop Total	Fold Enrichmen	Bonferroni	Benjamini	FDR
GOTERM	GO:0006813~potassium ion transport	8	0.304878049	0.196032039	Q84TI7,	1857	60	23397	1.67991384	1	1	0.979359044
GOTERM	GO:0015385~sodium:proton antiporter activity	3	0.114329268	0.800733756	Q9M353	1714	33	19438	1.030974859	1	1	0.977642276
GOTERM	GO:0006885~regulation of pH	3	0.114329268	0.803482876	Q9M353	1857	37	23397	1.021569227	1	1	0.979359044
GOTERM	GO:0015299~solute:proton antiporter activity	3	0.114329268	0.838508827	Q9M353	1714	36	19438	0.945060288	1	1	0.977642276
GOTERM	GO:1902600~hydrogen ion transmembrane transport	4	0.152439024	0.932127116	Q9M353	1857	72	23397	0.6999641	1	1	0.979359044

Annotatio Enrichment Score: 0.1888590018499799

Category	Term	Count	%	PValue	Genes	List Total	Pop Hits	Pop Total	Fold Enrichmen	Bonferroni	Benjamini	FDR
GOTERM	GO:0020037~heme binding	38	1.448170732	0.382270769	F4JW83,	1714	397	19438	1.085510054	1	1	0.977642276
GOTERM	GO:0005506~iron ion binding	32	1.219512195	0.555750622	Q1G3U6	1714	356	19438	1.019390872	1	1	0.977642276
GOTERM	GO:0016705~oxidoreductase activity, acting on paired donors, w	19	0.724085366	0.906226659	Q93VK5	1714	266	19438	0.810051675	1	1	0.977642276
GOTERM	GO:0004497~monooxygenase activity	19	0.724085366	0.912170686	Q93VK5	1714	268	19438	0.804006514	1	1	0.977642276

Annotatio Enrichment Score: 0.13795089583468909

Category	Term	Count	%	PValue	Genes	List Total	Pop Hits	Pop Total	Fold Enrichmen	Bonferroni	Benjamini	FDR
GOTERM	GO:0051259~protein oligomerization	3	0.114329268	0.395296824	Q9FHQ3	1857	17	23397	2.223415376	1	1	0.979359044
GOTERM	GO:0006457~protein folding	8	0.304878049	0.98164005	Q9FHQ3	1857	167	23397	0.603561859	1	1	0.98164005
GOTERM	GO:0051082~unfolded protein binding	5	0.19054878	0.99373759	Q9FHQ3	1714	117	19438	0.484646301	1	1	0.99373759

Annotatio Enrichment Score: 0.10767010185170979

Category	Term	Count	%	PValue	Genes	List Total	Pop Hits	Pop Total	Fold Enrichmen	Bonferroni	Benjamini	FDR
GOTERM	GO:0019005~SCF ubiquitin ligase complex	7	0.266768293	0.628260647	Q8W104	1899	88	25998	1.089006175	1	1	0.892063492
GOTERM	GO:0031146~SCF-dependent proteasomal ubiquitin-dependent p	7	0.266768293	0.75657589	Q8W104	1857	93	23397	0.948338458	1	1	0.979359044
GOTERM	GO:0006511~ubiquitin-dependent protein catabolic process	11	0.419207317	0.999993875	Q8W104	1857	368	23397	0.376611119	1	1	0.999993875

Annotatio Enrichment Score: 0.10476849074550987

Category	Term	Count	%	PValue	Genes	List Total	Pop Hits	Pop Total	Fold Enrichmen	Bonferroni	Benjamini	FDR
GOTERM	GO:0000978~RNA polymerase II core promoter proximal region	23	0.87652439	0.661792026	O22533,	1714	267	19438	0.976916253	1	1	0.977642276
GOTERM	GO:0006357~regulation of transcription from RNA polymerase I	26	0.990853659	0.833489292	Q8S3D1,	1857	372	23397	0.880599997	1	1	0.979359044
GOTERM	GO:0000981~RNA polymerase II transcription factor activity, se	23	0.87652439	0.879170229	Q8S3D1,	1714	309	19438	0.844131519	1	1	0.977642276

Annotatio Enrichment Score: 0.08983167876978032

Category	Term	Count	%	PValue	Genes	List Total	Pop Hits	Pop Total	Fold Enrichmen	Bonferroni	Benjamini	FDR
GOTERM	GO:0006470~protein dephosphorylation	9	0.342987805	0.566283802	O81760,	1857	102	23397	1.111707688	1	1	0.979359044
GOTERM	GO:0004722~protein serine/threonine phosphatase activity	4	0.152439024	0.961487887	O81760,	1714	73	19438	0.621409504	1	1	0.977642276
GOTERM	GO:0017018~myosin phosphatase activity	6	0.228658537	0.987477041	O81760,	1714	124	19438	0.548744683	1	1	0.987477041

Annotatio Enrichment Score: 0.0642302548905889

Category	Term	Count	%	PValue	Genes	List Total	Pop Hits	Pop Total	Fold Enrichmen	Bonferroni	Benjamini	FDR
GOTERM	GO:0005764~lysosome	4	0.152439024	0.662475106	Q9FMH8	1899	46	25998	1.190466378	1	1	0.892063492
GOTERM	GO:0051603~proteolysis involved in cellular protein catabolic pr	3	0.114329268	0.975285567	Q9FMH8	1857	68	23397	0.555853844	1	1	0.979359044
GOTERM	GO:0004197~cysteine-type endopeptidase activity	3	0.114329268	0.993134057	Q9FMH8	1714	77	19438	0.441846368	1	1	0.993134057

Annotatio Enrichment Score: 0.05602884571597219

Category	Term	Count	%	PValue	Genes	List Total	Pop Hits	Pop Total	Fold Enrichmen	Bonferroni	Benjamini	FDR
GOTERM	GO:0004683~calmodulin-dependent protein kinase activity	5	0.19054878	0.725888822	Q9CAL3	1714	55	19438	1.030974859	1	1	0.977642276
GOTERM	GO:0009931~calcium-dependent protein serine/threonine kinase	4	0.152439024	0.874048129	Q9CAL3	1714	55	19438	0.824779888	1	1	0.977642276
GOTERM	GO:0035556~intracellular signal transduction	12	0.457317073	0.946168079	Q38997,	1857	208	23397	0.726885796	1	1	0.979359044
GOTERM	GO:0018105~peptidyl-serine phosphorylation	4	0.152439024	0.994284958	Q9CAL3	1857	111	23397	0.454030767	1	1	0.994284958

Annotatio Enrichment Score: 0.03700712699262434

Category	Term	Count	%	PValue	Genes	List Total	Pop Hits	Pop Total	Fold Enrichmen	Bonferroni	Benjamini	FDR
GOTERM	GO:0022626~cytosolic ribosome	14	0.533536585	0.821984269	O50061,	1899	220	25998	0.87120494	1	1	0.892063492
GOTERM	GO:0042788~polysomal ribosome	5	0.19054878	0.948273893	O50061,	1899	103	25998	0.664580745	1	1	0.948273893
GOTERM	GO:0022625~cytosolic large ribosomal subunit	6	0.228658537	0.993530763	O50061,	1899	163	25998	0.503939755	1	1	0.993530763

Table. S.12. The performance of genomic selection models with different SNPs in cross validation (binary phenotype)

SNPs	1				10				50				100				500				2000				5000				All			
cv	Logistic Regression	Naive Bayes	Random Forest	SVC	Logistic Regression	Naive Bayes	Random Forest	SVC	Logistic Regression	Naive Bayes	Random Forest	SVC	Logistic Regression	Naive Bayes	Random Forest	SVC	Logistic Regression	Naive Bayes	Random Forest	SVC	Logistic Regression	Naive Bayes	Random Forest	SVC	Logistic Regression	Naive Bayes	Random Forest	SVC	Logistic Regression	Naive Bayes	Random Forest	SVC
1	0.57	0.6	0.57	0.57	0.67	0.69	0.69	0.69	0.79	0.74	0.71	0.76	0.86	0.76	0.76	0.81	0.9	0.76	0.79	0.86	0.93	0.83	0.79	0.9	0.88	0.81	0.74	0.88	0.64	0.71	0.71	0.67
2	0.64	0.64	0.64	0.64	0.81	0.74	0.86	0.88	0.95	0.81	0.83	0.93	0.98	0.79	0.88	0.93	1	0.79	0.83	0.98	1	0.86	0.83	0.95	0.98	0.88	0.83	1	0.71	0.79	0.79	0.69
3	0.67	0.57	0.67	0.67	0.81	0.71	0.79	0.81	0.9	0.76	0.79	0.88	0.79	0.71	0.81	0.79	0.88	0.74	0.74	0.95	0.95	0.79	0.86	0.93	0.93	0.83	0.81	0.93	0.71	0.74	0.71	0.71
4	0.67	0.67	0.67	0.67	0.76	0.76	0.81	0.79	0.83	0.81	0.86	0.86	0.95	0.86	0.88	0.93	0.88	0.83	0.83	0.9	0.95	0.88	0.9	0.9	0.98	0.88	0.88	0.98	0.71	0.79	0.76	0.74
5	0.6	0.57	0.6	0.6	0.9	0.9	0.9	0.95	0.9	0.9	0.93	0.9	0.9	0.9	0.93	0.9	0.95	0.9	0.9	0.98	1	0.93	0.93	1	0.95	0.95	0.93	0.95	0.69	0.83	0.83	0.79
6	0.69	0.69	0.69	0.69	0.86	0.81	0.79	0.86	0.93	0.83	0.93	0.93	0.98	0.83	0.93	0.93	0.95	0.81	0.83	0.93	0.98	0.93	0.86	0.98	0.98	0.88	0.88	0.98	0.76	0.76	0.74	0.74
7	0.64	0.71	0.64	0.64	0.83	0.81	0.81	0.88	0.9	0.86	0.9	0.9	0.98	0.88	0.88	0.86	0.9	0.88	0.9	1	0.93	0.88	0.93	0.88	0.93	0.93	0.93	0.95	0.74	0.83	0.81	0.76
8	0.52	0.48	0.52	0.52	0.76	0.79	0.81	0.79	0.81	0.83	0.76	0.71	0.88	0.83	0.88	0.83	0.9	0.81	0.86	0.88	0.9	0.83	0.88	0.9	0.9	0.86	0.86	0.88	0.62	0.76	0.74	0.67
9	0.71	0.6	0.71	0.71	0.74	0.64	0.69	0.79	0.74	0.62	0.71	0.74	0.76	0.57	0.64	0.76	0.81	0.6	0.67	0.88	0.88	0.76	0.71	0.88	0.86	0.81	0.67	0.86	0.57	0.57	0.6	0.6
10	0.5	0.48	0.5	0.5	0.71	0.79	0.81	0.74	0.9	0.79	0.93	0.88	0.95	0.81	0.93	0.86	0.93	0.79	0.88	0.9	0.98	0.88	0.88	0.95	0.98	0.93	0.86	0.98	0.69	0.79	0.76	0.69
11	0.6	0.62	0.6	0.6	0.74	0.76	0.79	0.79	0.9	0.81	0.83	0.9	0.95	0.79	0.83	0.93	0.93	0.81	0.83	0.93	1	0.9	0.86	0.98	0.98	0.86	0.83	0.98	0.71	0.74	0.74	0.71
12	0.57	0.62	0.57	0.57	0.86	0.74	0.86	0.86	0.88	0.86	0.83	0.86	0.9	0.86	0.88	0.93	1	0.9	0.98	0.98	0.98	0.93	1	0.98	0.98	0.95	1	0.95	0.76	0.86	0.83	0.81
13	0.55	0.62	0.55	0.55	0.79	0.83	0.74	0.83	0.76	0.76	0.74	0.71	0.83	0.81	0.79	0.81	0.88	0.83	0.81	0.86	0.88	0.83	0.81	0.86	0.86	0.83	0.81	0.86	0.71	0.81	0.74	0.69
14	0.71	0.69	0.71	0.71	0.79	0.69	0.79	0.79	0.9	0.79	0.81	0.95	0.93	0.79	0.86	0.83	0.93	0.79	0.76	0.93	0.93	0.88	0.81	0.9	0.95	0.88	0.83	0.95	0.62	0.71	0.74	0.64
15	0.57	0.55	0.57	0.57	0.81	0.79	0.81	0.86	0.93	0.76	0.88	0.9	0.9	0.81	0.9	0.9	0.98	0.81	0.88	0.98	0.98	0.86	0.86	0.98	0.95	0.88	0.86	0.95	0.79	0.79	0.79	0.76
16	0.57	0.55	0.57	0.57	0.74	0.79	0.81	0.74	0.9	0.79	0.83	0.93	0.9	0.83	0.88	0.81	0.9	0.86	0.86	0.9	0.9	0.88	0.88	0.9	0.9	0.88	0.86	0.9	0.71	0.79	0.74	0.71
17	0.64	0.52	0.52	0.64	0.81	0.67	0.83	0.79	0.88	0.74	0.74	0.86	0.9	0.79	0.81	0.79	0.93	0.76	0.86	0.9	0.95	0.86	0.76	0.93	0.95	0.88	0.74	0.95	0.6	0.67	0.69	0.64
18	0.67	0.57	0.67	0.67	0.76	0.67	0.69	0.76	0.81	0.64	0.74	0.74	0.9	0.69	0.79	0.81	0.9	0.67	0.81	0.9	0.95	0.76	0.74	0.93	0.93	0.81	0.76	0.93	0.55	0.62	0.62	0.55
19	0.69	0.62	0.69	0.69	0.71	0.64	0.74	0.74	0.76	0.64	0.69	0.81	0.86	0.62	0.71	0.81	0.86	0.67	0.71	0.83	0.9	0.71	0.71	0.93	0.88	0.81	0.71	0.86	0.6	0.64	0.67	0.62
20	0.52	0.52	0.52	0.52	0.76	0.76	0.76	0.79	0.93	0.81	0.83	0.9	0.98	0.86	0.86	0.88	0.95	0.86	0.83	0.95	0.98	0.9	0.9	0.98	0.95	0.9	0.9	0.95	0.74	0.76	0.76	0.76
21	0.55	0.52	0.55	0.55	0.76	0.79	0.74	0.76	0.83	0.83	0.81	0.83	0.9	0.79	0.79	0.86	0.88	0.86	0.81	0.81	0.86	0.9	0.83	0.86	0.88	0.93	0.86	0.81	0.64	0.81	0.74	0.69
22	0.45	0.5	0.45	0.45	0.76	0.71	0.74	0.76	0.81	0.79	0.74	0.79	0.88	0.79	0.76	0.81	0.88	0.83	0.83	0.88	0.95	0.86	0.81	0.9	0.93	0.93	0.83	0.88	0.64	0.71	0.74	0.69
23	0.64	0.55	0.64	0.64	0.79	0.76	0.81	0.81	0.81	0.76	0.83	0.81	0.9	0.79	0.86	0.86	0.88	0.79	0.81	0.88	0.95	0.88	0.83	0.95	0.95	0.88	0.86	0.95	0.71	0.76	0.74	0.71
24	0.52	0.57	0.52	0.52	0.79	0.76	0.76	0.79	0.9	0.86	0.86	0.88	0.93	0.86	0.88	0.86	0.95	0.88	0.9	0.93	0.93	0.9	0.88	0.93	0.98	0.93	0.86	0.98	0.81	0.76	0.76	0.76
25	0.64	0.57	0.64	0.64	0.79	0.76	0.67	0.79	0.9	0.76	0.79	0.86	0.9	0.81	0.83	0.86	0.93	0.81	0.81	0.86	0.93	0.86	0.88	0.86	0.9	0.86	0.83	0.83	0.62	0.74	0.69	0.67
26	0.55	0.52	0.55	0.55	0.71	0.69	0.83	0.76	0.86	0.62	0.9	0.83	0.93	0.67	0.83	0.93	0.95	0.79	0.83	0.95	1	0.79	0.81	0.98	0.98	0.88	0.81	0.98	0.69	0.67	0.67	0.64
27	0.57	0.57	0.57	0.57	0.79	0.81	0.86	0.86	0.9	0.83	0.88	0.88	0.93	0.83	0.83	0.81	0.86	0.83	0.86	0.86	0.95	0.9	0.88	0.9	0.9	0.9	0.88	0.93	0.64	0.83	0.74	0.69
28	0.5	0.48	0.48	0.48	0.74	0.64	0.71	0.76	0.71	0.62	0.71	0.74	0.76	0.64	0.76	0.76	0.83	0.62	0.71	0.83	0.86	0.74	0.71	0.81	0.83	0.74	0.71	0.83	0.62	0.62	0.6	0.6
29	0.62	0.69	0.62	0.62	0.76	0.76	0.74	0.79	0.88	0.83	0.86	0.9	0.98	0.83	0.86	0.95	0.9	0.86	0.88	0.95	0.98	0.93	0.88	0.95	0.95	0.88	0.86	0.95	0.69	0.79	0.76	0.69
30	0.5	0.62	0.5	0.5	0.74	0.69	0.74	0.74	0.81	0.67	0.76	0.74	0.83	0.71	0.76	0.81	0.93	0.76	0.74	0.93	0.93	0.83	0.81	0.9	0.86	0.83	0.79	0.88	0.6	0.71	0.67	0.62
31	0.55	0.52	0.55	0.55	0.69	0.62	0.71	0.69	0.83	0.69	0.79	0.81	0.86	0.76	0.79	0.88	0.86	0.74	0.81	0.86	0.83	0.79	0.81	0.83	0.86	0.76	0.79	0.86	0.69	0.69	0.69	0.69
32	0.5	0.48	0.5	0.5	0.9	0.76	0.83	0.88	0.88	0.83	0.88	0.86	0.98	0.83	0.9	0.86	0.93	0.86	0.88	0.93	0.95	0.9	0.9	0.95	0.93	0.9	0.93	0.95	0.76	0.79	0.76	0.79
33	0.52	0.52	0.52	0.52	0.67	0.67	0.67	0.64	0.83	0.71	0.74	0.76	0.86	0.76	0.76	0.79	0.86	0.74	0.74	0.83	0.9	0.81	0.76	0.83	0.9	0.83	0.76	0.9	0.57	0.67	0.64	0.6
34	0.55	0.45	0.55	0.55	0.74	0.69	0.79	0.81	0.86	0.74	0.76	0.86	0.93	0.74	0.88	0.9	0.9	0.74	0.81	0.88	0.9	0.81	0.76	0.86	0.9	0.81	0.76	0.88	0.69	0.67	0.64	0.67
35	0.67	0.62	0.67	0.67	0.9	0.88	0.86	0.95	0.98	0.93	0.88	0.9	0.95	0.9	0.9	0.88	0.88	0.88	0.9	0.88	1	0.95	0.9	0.93	0.98	0.93	0.93	0.98	0.64	0.83	0.79	0.67
36	0.6	0.57	0.6	0.6	0.83	0.76	0.83	0.79	0.93	0.81	0.9	0.86	0.9	0.81	0.93	0.9	0.98	0.86	0.9	0.93	1	0.88	0.88	0.98	1	0.95	0.93	1	0.76	0.76	0.76	0.79
37	0.69	0.62	0.69	0.69	0.79	0.76	0.81	0.83	0.86	0.79	0.81	0.81	0.86	0.81	0.83	0.83	0.93	0.83	0.86	0.93	0.95	0.86	0.83	0.93	0.9	0.88	0.86	0.9	0.74	0.76	0.76	0.71
38	0.55	0.6	0.55	0.55	0.71	0.71	0.83	0.76	0.81	0.71	0.81	0.74	0.93	0.79	0.81	0.81	0.88	0.79	0.86	0.88	0.93	0.81	0.88	0.93	0.9	0.86	0.86	0.9	0.64	0.74	0.69	0.64
39	0.62	0.52	0.62	0.62	0.81	0.76	0.81	0.81	0.9	0.76	0.86	0.93	0.88	0.76	0.83	0.86	0.9	0.81	0.86	0.9	0.93	0.9	0.81	0.9	0.95	0.9	0.83	0.95	0.64	0.74	0.74	0.62
40	0.55	0.45	0.45	0.55	0.71	0.71	0.74	0.79	0.81	0.81	0.83	0.86	0.88	0.81	0.83	0.86	0.98	0.81	0.88	0.93	1	0.9	0.9	0.98	1	0.9	0.86	1	0.81	0.76	0.74	0.74
41	0.64	0.67	0.64	0.64	0.76	0.74	0.69	0.79	0.81	0.81	0.79	0.81	0.86	0.83	0.83	0.86	0.93	0.79	0.86	0.9	0.93	0.88	0.83	0.93	0.93	0.88	0.88	0.93	0.6	0.69	0.64	0.64
42	0.64	0.6	0.64	0.64	0.81	0.69	0.79	0.76	0.81	0.76	0.76	0.81	0.86	0.79	0.86	0.83	0.86	0.81	0.86	0.9	0.95	0.86	0.88	0.95	0.9	0.88	0.81	0.9	0.64	0.69	0.69	0.69
43	0.67	0.67	0.67	0.67	0.81	0.79	0.79	0.83	0.93	0.83	0.86	0.9	0.95	0.83	0.81	0.88	0.95	0.86	0.9	0.88	0.95	0.88	0.88	0.9	0.98	0.88	0.88	0.98	0.62	0.83	0.74	0.67
44	0.67	0.67	0.67	0.67	0.76	0.71	0.74	0.74	0.88	0.81	0.86	0.83	0.88	0.79	0.86	0.83	0.93	0.81	0.81	0.93	0.93	0.9	0.83	0.93	0.95	0.9	0.88	0.95	0.69	0.74	0.74	0.71
45	0.6	0.52	0.6	0.6	0.81	0.74	0.83	0.83	0.9	0.74	0.76	0.9	0.83	0.81	0.79	0.81	0.9	0.81	0.83	0.88	0.9	0.88	0.83	0.9	0.9	0.88	0.81	0.9	0.64	0.71	0.67	0.69
46	0.69	0.57	0.69	0.69	0.76	0.76	0.76	0.86	0.86	0.79	0.83	0.88	0.93	0.81	0.83	0.86	0.95	0.81	0.83	0.88	0.93	0.83	0.86	0.86	0.93	0.9	0.83	0.95	0.67	0.76	0.76	0.69
47	0.71	0.69	0.71	0.71	0.93	0.83	0.93	0.9	0.93	0.83	0.86	0.9	0.88	0.83	0.9	0.86	0.9	0.9	0.95	0.88	0.98	0.95	0.95	0.95	0.98	0.93	0.9	0.95	0.67	0.86	0.76	0.69
48	0.6	0.6	0.6	0.6	0.86	0.83	0.81	0.83	0.86	0.88	0.88	0.86	0.95	0.88	0.9	0.88	0.9	0.93	0.98	0.93	0.95	0.95	0.98	0.95	0.93	0.95	0.93	0.93	0.76	0.83	0.79	0.74
49	0.62	0.62	0.62	0.62	0.81	0.79	0.83	0.81	0.95	0.81	0.93	0.86	0.93	0.81	0.93	0.9	0.98	0.83	0.9	0.95	1	0.88	0.88	0.98	1	0.95	0.95	1	0.81	0.79	0.79	0.79
50	0.5	0.48	0.5	0.5	0.67	0.69	0.64	0.69	0.79	0.76	0.71	0.79	0.76	0.81	0.69	0.74	0.93	0.76	0.76	0.86	0.95	0.81	0.76	0.9	0.95	0.88	0.79	0.95	0.6	0.71	0.67	0.62
51	0.55	0.52	0.55	0.55	0.79	0.74	0.81	0.81	0.95	0.81	0.88	0.95	0.9	0.79	0.86	0.86	0.93	0.83	0.83	0.9	0.9	0.88	0.88	0.93	0.9	0.9	0.83	0.88	0.69	0.74	0.74	0.67
52	0.45	0.43	0.55	0.55	0.69	0.71	0.74	0.71	0.88	0.71	0.71	0.83	0.83	0.71	0.71	0.76	0.83	0.71	0.69	0.79	0.88	0.81	0.76	0.86	0.86	0.88	0.74	0.88	0.6	0.71	0.64	0.64
53	0.64	0.62	0.64	0.64	0.86	0.79	0.83	0.83	0.86	0.76	0.79	0.81	0.9	0.79	0.81	0.86	0.9	0.79	0.81	0.93	0.98	0.86	0.88	0.93	0.98	0.81	0.86	0.98	0.67	0.76	0.74	0.69
54	0.71	0.69	0.71	0.71	0.71	0.69	0.79	0.74	0.86	0.74	0.79	0.81	0.9	0.81	0.81	0.86	0.9	0.79	0.88	0.88	0.98	0.88	0.86	0.95	0.95	0.9	0.83	0.95	0.71	0.74	0.71	0.71
55	0.57	0.57	0.57	0.57	0.76	0.71	0.79	0.79	0.88	0.83	0.86	0.86	0.95	0.86	0.83	0.9	0.98	0.86	0.88	0.93	0.98	0.88	0.9	0.95	0.98	0.88	0.9	0.95	0.69	0.79	0.79	0.69
56	0.5	0.5	0.5	0.5	0.81	0.64	0.83	0.79	0.88	0.71	0.76	0.88	0.88	0.74	0.81	0.79	0.9	0.76	0.88	0.9	1	0.79	0.83	1	0.95	0.83	0.76	0.93	0.69	0.67	0.67	0.62
57	0.57	0.55	0.57	0.57	0.71	0.69	0.71	0.69	0.86	0.69	0.79	0.88	0.9	0.67	0.79	0.81	0.93	0.67	0.74	0.95	0.9	0.79	0.81	0.9	0.86	0.79	0.71	0.86	0.62	0.64	0.64	0.62
58	0.62	0.6	0.62	0.62	0.83	0.76	0.83	0.81	0.86	0.76	0.86	0.88	0.95	0.81	0.88	0.88	0.88	0.83	0.88	0.9	0.9	0.86	0.81	0.9	0.95	0.86	0.88	0.93	0.64	0.76	0.74	0.71
59	0.38	0.4	0.38	0.38	0.79	0.69	0.74	0.79	0.88	0.76	0.83	0.86	0.93	0.76	0.81	0.88	0.88	0.79	0.79	0.86	0.95	0.9	0.83	0.93	0.93	0.88	0.79	0.88	0.71	0.74	0.69	0.69
60	0.6	0.62	0.6	0.6	0.83	0.81	0.88	0.83	0.9	0.86	0.88	0.86	0.93	0.88	0.93	0.9	0.93	0.9	0.93	0.95	0.98	0.98	0.93	0.93	0.95	0.95	0.88	0.98	0.76	0.88	0.83	0.74
61	0.6	0.6	0.74	0.6	0.81	0.76	0.74	0.83	0.83	0.81	0.86	0.83	0.88	0.79	0.79	0.86	0.93	0.81	0.86	0.88	0.93	0.88	0.83	0.93	0.98	0.88	0.86	0.98	0.71	0.74	0.74	0.71
62	0.52	0.43	0.52	0.52	0.71	0.62	0.67	0.79	0.86	0.69	0.83	0.83	0.88	0.67	0.76	0.79	0.86	0.69	0.71	0.81	0.95	0.83	0.71	0.88	0.9	0.81	0.69	0.88	0.57	0.64	0.62	0.57
63	0.57	0.55	0.57	0.57	0.76	0.86	0.79	0.81	0.88	0.86	0.83	0.9	0.95	0.83	0.83	0.88	0.9	0.86	0.88	0.9	0.95	0.93	0.83	0.9	0.95	0.9	0.88	0.88	0.64	0.81	0.79	0.69
64	0.6	0.48	0.6	0.6	0.79	0.74	0.83	0.83	0.93	0.81	0.88	0.83	0.88	0.83	0.81	0.79	0.81	0.76	0.79	0.86	0.98	0.86	0.79	0.98	0.95	0.86	0.83	0.95	0.64	0.71	0.71	0.64
65	0.55	0.52	0.55	0.55	0.81	0.79	0.79	0.81	0.93	0.81	0.88	0.88	0.93	0.86	0.88	0.88	0.98	0.86	0.9	0.95	0.98	0.93	0.86	0.95	0.93	0.9	0.9	0.93	0.74	0.83	0.76	0.76
66	0.64	0.57	0.64	0.64	0.83	0.86	0.79	0.86	0.86	0.74	0.86	0.88	0.9	0.81	0.86	0.86	0.9	0.79	0.88	0.9	0.93	0.88	0.83	0.88	0.95	0.93	0.88	0.93	0.62	0.76	0.74	0.69
67	0.55	0.71	0.55	0.55	0.88	0.88	0.81	0.88	0.93	0.88	0.86	0.9	0.86	0.83	0.9	0.86	0.98	0.88	0.9	0.95	0.95	0.9	0.88	0.93	0.95	0.93	0.93	0.95	0.67	0.81	0.79	0.64
68	0.69	0.69	0.69	0.69	0.79	0.76	0.76	0.81	0.86	0.81	0.86	0.83	0.93	0.88	0.88	0.83	0.95	0.86	0.88	0.95	0.98	0.88	0.86	0.93	0.95	0.88	0.88	0.93	0.64	0.76	0.76	0.74
69	0.67	0.6	0.67	0.67	0.9	0.67	0.76	0.83	0.83	0.74	0.86	0.83	0.88	0.79	0.86	0.88	0.9	0.79	0.86	0.9	0.93	0.86	0.83	0.93	0.95	0.86	0.79	0.95	0.64	0.69	0.69	0.67
70	0.6	0.64	0.6	0.6	0.86	0.74	0.76	0.79	0.83	0.74	0.81	0.86	0.93	0.81	0.88	0.93	0.95	0.76	0.9	0.98	0.95	0.86	0.81	0.93	0.95	0.9	0.88	0.95	0.64	0.71	0.71	0.64
71	0.67	0.69	0.67	0.67	0.81	0.76	0.86	0.83	0.86	0.83	0.86	0.81	0.9	0.88	0.93	0.86	0.95	0.86	0.93	1	1	0.86	0.93	0.98	0.98	0.93	0.9	0.98	0.76	0.79	0.79	0.74
72	0.6	0.52	0.6	0.6	0.71	0.67	0.69	0.76	0.88	0.69	0.76	0.86	0.86	0.76	0.76	0.79	0.88	0.71	0.79	0.86	0.88	0.79	0.76	0.86	0.9	0.74	0.74	0.93	0.62	0.64	0.67	0.67
73	0.67	0.67	0.67	0.67	0.81	0.76	0.79	0.83	0.9	0.86	0.86	0.86	0.98	0.86	0.93	0.9	0.98	0.88	0.88	0.98	0.9	0.9	0.93	0.9	0.93	0.9	0.93	0.93	0.71	0.81	0.79	0.74
74	0.57	0.55	0.57	0.57	0.76	0.69	0.71	0.74	0.9	0.74	0.83	0.86	0.88	0.81	0.83	0.83	0.98	0.76	0.81	0.98	0.95	0.83	0.79	0.93	0.93	0.86	0.79	0.9	0.69	0.71	0.71	0.67
75	0.69	0.6	0.69	0.69	0.83	0.81	0.81	0.88	0.88	0.83	0.88	0.88	0.93	0.83	0.95	0.95	0.98	0.83	0.93	0.95	0.98	0.88	0.86	0.93	0.98	0.9	0.83	0.95	0.69	0.83	0.79	0.74
76	0.6	0.52	0.6	0.6	0.81	0.71	0.83	0.81	0.9	0.76	0.88	0.95	0.93	0.74	0.81	0.83	0.98	0.83	0.88	0.98	0.98	0.93	0.88	0.98	0.98	0.88	0.88	0.95	0.74	0.71	0.76	0.76
77	0.45	0.5	0.45	0.45	0.79	0.81	0.81	0.88	0.86	0.83	0.9	0.83	0.93	0.83	0.88	0.9	0.98	0.9	0.9	1	0.98	0.93	0.93	0.98	0.98	0.9	0.95	0.98	0.81	0.81	0.81	0.79
78	0.57	0.55	0.57	0.57	0.88	0.76	0.83	0.88	0.81	0.81	0.83	0.79	0.93	0.83	0.83	0.81	0.88	0.83	0.86	0.88	0.95	0.9	0.86	0.93	0.93	0.86	0.83	0.93	0.69	0.74	0.76	0.71
79	0.6	0.48	0.6	0.6	0.76	0.69	0.79	0.79	0.9	0.71	0.79	0.83	0.86	0.74	0.74	0.83	0.9	0.74	0.76	0.83	0.93	0.83	0.83	0.88	0.95	0.86	0.74	0.98	0.67	0.71	0.67	0.67
80	0.5	0.5	0.5	0.5	0.79	0.64	0.81	0.81	0.86	0.69	0.83	0.76	0.86	0.71	0.86	0.86	0.86	0.74	0.83	0.93	0.93	0.81	0.81	0.9	0.95	0.81	0.86	0.93	0.57	0.64	0.62	0.55
81	0.67	0.64	0.67	0.67	0.83	0.79	0.81	0.81	0.81	0.79	0.83	0.9	0.98	0.81	0.9	0.9	0.9	0.81	0.83	0.93	0.9	0.81	0.83	0.93	0.93	0.83	0.83	0.93	0.69	0.76	0.74	0.67
82	0.6	0.57	0.6	0.6	0.74	0.79	0.74	0.74	0.86	0.81	0.76	0.86	0.88	0.83	0.86	0.79	0.95	0.81	0.81	0.9	0.95	0.83	0.86	0.93	1	0.9	0.86	1	0.71	0.79	0.74	0.71
83	0.57	0.6	0.57	0.57	0.79	0.79	0.79	0.79	0.83	0.83	0.81	0.88	0.88	0.79	0.86	0.88	0.9	0.86	0.88	0.93	0.95	0.88	0.9	0.93	0.9	0.9	0.88	0.9	0.64	0.79	0.76	0.67
84	0.57	0.55	0.57	0.57	0.74	0.76	0.76	0.79	0.76	0.79	0.79	0.74	0.93	0.81	0.83	0.86	0.86	0.81	0.79	0.86	0.95	0.9	0.81	0.93	0.93	0.9	0.81	0.9	0.64	0.79	0.76	0.67
85	0.52	0.57	0.52	0.52	0.83	0.81	0.81	0.86	0.93	0.81	0.83	0.9	0.9	0.79	0.86	0.86	1	0.81	0.83	0.98	0.95	0.83	0.86	0.95	0.93	0.86	0.86	0.95	0.69	0.76	0.76	0.74
86	0.74	0.74	0.74	0.74	0.88	0.83	0.88	0.9	0.93	0.81	0.9	0.93	0.9	0.83	0.86	0.88	0.95	0.86	0.93	0.93	0.98	0.9	0.9	0.98	0.95	0.93	0.93	0.98	0.69	0.83	0.83	0.79
87	0.52	0.52	0.52	0.52	0.76	0.74	0.81	0.81	0.88	0.76	0.86	0.88	0.95	0.76	0.86	0.86	0.95	0.83	0.81	1	0.98	0.86	0.86	0.98	0.95	0.86	0.81	0.95	0.74	0.76	0.74	0.69
88	0.57	0.52	0.57	0.57	0.69	0.67	0.79	0.79	0.88	0.83	0.83	0.88	0.93	0.79	0.86	0.9	0.9	0.81	0.88	0.88	0.95	0.88	0.83	0.9	0.93	0.88	0.81	0.9	0.62	0.76	0.71	0.69
89	0.57	0.64	0.57	0.57	0.79	0.76	0.76	0.83	0.81	0.79	0.81	0.71	0.86	0.81	0.86	0.86	0.95	0.83	0.9	0.93	0.9	0.83	0.86	0.9	0.9	0.86	0.86	0.93	0.69	0.79	0.76	0.76
90	0.6	0.6	0.6	0.6	0.76	0.69	0.71	0.79	0.81	0.69	0.74	0.81	0.93	0.81	0.83	0.79	0.88	0.79	0.76	0.9	0.95	0.83	0.81	0.95	0.98	0.86	0.83	0.98	0.64	0.67	0.62	0.6
91	0.5	0.45	0.5	0.5	0.76	0.83	0.83	0.76	0.88	0.88	0.81	0.86	0.88	0.9	0.83	0.83	0.9	0.93	0.9	0.93	0.93	0.95	0.88	0.9	0.9	0.93	0.88	0.88	0.74	0.83	0.79	0.74
92	0.57	0.5	0.57	0.57	0.79	0.67	0.71	0.74	0.81	0.69	0.79	0.79	0.86	0.76	0.83	0.86	0.93	0.79	0.83	0.9	0.95	0.83	0.83	0.93	0.9	0.88	0.79	0.9	0.64	0.69	0.64	0.62
93	0.57	0.48	0.57	0.57	0.74	0.64	0.74	0.74	0.83	0.69	0.71	0.74	0.83	0.71	0.76	0.76	0.93	0.71	0.74	0.9	0.95	0.81	0.81	0.95	0.95	0.81	0.74	0.95	0.6	0.64	0.62	0.6
94	0.62	0.55	0.62	0.62	0.69	0.67	0.76	0.76	0.86	0.79	0.74	0.86	0.9	0.81	0.83	0.79	0.79	0.79	0.79	0.76	0.88	0.86	0.79	0.86	0.86	0.88	0.86	0.86	0.52	0.74	0.6	0.55

95	0.57	0.55	0.57	0.57	0.71	0.62	0.69	0.69	0.79	0.69	0.71	0.79	0.79	0.71	0.74	0.74	0.86	0.67	0.64	0.86	0.88	0.79	0.79	0.88	0.86	0.81	0.71	0.86	0.6	0.64	0.64	0.6
96	0.48	0.45	0.6	0.45	0.79	0.71	0.76	0.81	0.79	0.71	0.81	0.79	0.81	0.74	0.81	0.76	0.86	0.74	0.79	0.86	0.93	0.81	0.79	0.9	0.83	0.79	0.79	0.88	0.67	0.74	0.69	0.69
97	0.62	0.62	0.62	0.62	0.83	0.86	0.81	0.88	0.95	0.9	0.9	0.95	0.98	0.88	0.88	0.93	0.95	0.93	0.95	0.95	0.98	0.98	0.93	0.95	0.98	0.93	0.9	0.95	0.79	0.86	0.83	0.83
98	0.62	0.5	0.62	0.62	0.83	0.76	0.83	0.83	0.93	0.79	0.83	0.88	0.93	0.81	0.83	0.83	0.93	0.76	0.86	0.93	0.98	0.9	0.81	0.95	0.98	0.9	0.81	0.98	0.64	0.71	0.71	0.64
99	0.62	0.5	0.62	0.62	0.81	0.64	0.74	0.76	0.86	0.74	0.79	0.83	0.9	0.76	0.86	0.9	0.98	0.79	0.86	0.93	0.98	0.88	0.79	0.93	0.93	0.88	0.86	0.95	0.67	0.67	0.67	0.64
100	0.6	0.52	0.6	0.6	0.74	0.67	0.71	0.81	0.88	0.71	0.83	0.88	0.88	0.71	0.83	0.86	0.88	0.76	0.81	0.88	0.9	0.81	0.83	0.88	0.9	0.86	0.83	0.93	0.6	0.67	0.64	0.62

cv	SVC_linear	SVC_poly	SVC_rbf	SVC_linear	SVC_poly	SVC_rbf	SVC_linear	SVC_poly	SVC_rbf	SVC_linear	SVC_poly	SVC_rbf	SVC_linear	SVC_poly	SVC_rbf	SVC_linear	SVC_poly	SVC_rbf	SVC_linear	SVC_poly	SVC_rbf	SVC_linear	SVC_poly	SVC_rbf
1	0.57	0.57	0.57	0.69	0.76	0.71	0.76	0.71	0.71	0.79	0.81	0.76	0.93	0.86	0.88	0.93	0.88	0.9	0.88	0.86	0.88	0.6	0.67	0.62
2	0.64	0.64	0.64	0.88	0.81	0.86	0.93	0.88	0.86	0.83	0.93	0.9	0.95	0.98	0.98	0.95	0.95	0.95	1	0.93	1	0.69	0.69	0.69
3	0.67	0.67	0.67	0.81	0.69	0.79	0.88	0.79	0.76	0.76	0.79	0.86	0.86	0.95	0.93	0.9	0.9	0.93	0.93	0.9	0.9	0.71	0.71	0.71
4	0.67	0.67	0.67	0.79	0.81	0.83	0.86	0.81	0.76	0.83	0.93	0.81	0.88	0.9	0.93	0.9	0.9	0.9	0.98	0.95	1	0.69	0.74	0.71
5	0.6	0.6	0.6	0.95	0.83	0.79	0.9	0.9	0.83	0.86	0.9	0.9	0.98	0.98	0.98	0.98	1	1	0.95	0.93	0.95	0.67	0.79	0.71
6	0.69	0.69	0.69	0.86	0.86	0.83	0.93	0.9	0.9	0.88	0.93	0.95	0.88	0.93	0.93	0.98	0.98	0.98	0.98	0.95	0.98	0.76	0.74	0.74
7	0.64	0.64	0.64	0.88	0.86	0.79	0.9	0.9	0.83	0.83	0.86	0.88	0.93	1	0.95	0.88	0.88	0.88	0.95	0.95	0.95	0.74	0.76	0.74
8	0.52	0.52	0.52	0.79	0.81	0.81	0.71	0.74	0.67	0.79	0.83	0.86	0.93	0.88	0.9	0.88	0.88	0.9	0.88	0.88	0.88	0.62	0.67	0.62
9	0.71	0.71	0.71	0.79	0.67	0.69	0.74	0.74	0.71	0.64	0.76	0.71	0.79	0.88	0.81	0.88	0.86	0.88	0.86	0.86	0.86	0.57	0.6	0.57
10	0.5	0.5	0.5	0.74	0.81	0.86	0.88	0.83	0.83	0.86	0.86	0.93	0.95	0.9	0.95	0.95	0.98	0.95	0.98	0.98	0.98	0.67	0.69	0.67
11	0.6	0.6	0.6	0.79	0.71	0.74	0.9	0.79	0.74	0.88	0.93	0.9	0.95	0.93	0.93	0.98	0.98	0.98	0.98	0.98	0.95	0.69	0.71	0.69
12	0.57	0.57	0.57	0.86	0.86	0.86	0.86	0.79	0.79	0.86	0.93	0.88	0.93	0.98	0.95	0.95	0.98	0.98	0.95	0.98	0.95	0.76	0.81	0.81
13	0.55	0.55	0.55	0.83	0.83	0.83	0.71	0.76	0.71	0.81	0.81	0.81	0.88	0.86	0.86	0.86	0.83	0.86	0.86	0.83	0.83	0.71	0.69	0.71
14	0.71	0.71	0.71	0.79	0.81	0.79	0.95	0.9	0.9	0.88	0.83	0.86	0.95	0.93	0.9	0.9	0.9	0.9	0.95	0.95	0.93	0.62	0.64	0.62
15	0.57	0.57	0.57	0.86	0.79	0.83	0.9	0.88	0.79	0.9	0.9	0.9	0.93	0.98	0.95	0.98	0.93	0.98	0.95	0.93	0.95	0.79	0.76	0.76
16	0.57	0.57	0.57	0.74	0.76	0.79	0.93	0.86	0.83	0.79	0.81	0.83	0.88	0.9	0.9	0.9	0.9	0.9	0.9	0.93	0.93	0.71	0.71	0.71
17	0.52	0.52	0.64	0.79	0.81	0.83	0.86	0.79	0.76	0.88	0.79	0.81	0.86	0.9	0.88	0.93	0.95	0.93	0.95	0.9	0.93	0.57	0.64	0.62
18	0.67	0.67	0.67	0.76	0.71	0.74	0.74	0.81	0.79	0.76	0.81	0.79	0.9	0.9	0.86	0.9	0.9	0.93	0.93	0.93	0.93	0.57	0.55	0.55
19	0.69	0.69	0.69	0.74	0.69	0.71	0.81	0.79	0.74	0.86	0.81	0.81	0.81	0.83	0.83	0.9	0.88	0.93	0.86	0.86	0.88	0.6	0.62	0.62
20	0.52	0.52	0.52	0.79	0.74	0.76	0.9	0.86	0.86	0.93	0.88	0.88	1	0.95	0.98	0.95	0.98	0.98	0.95	0.95	0.95	0.71	0.76	0.74
21	0.55	0.55	0.55	0.76	0.79	0.79	0.83	0.76	0.76	0.86	0.86	0.83	0.76	0.81	0.83	0.88	0.86	0.86	0.81	0.83	0.81	0.62	0.69	0.69
22	0.45	0.45	0.45	0.76	0.76	0.81	0.79	0.76	0.79	0.88	0.81	0.86	0.88	0.88	0.88	0.88	0.9	0.9	0.88	0.88	0.88	0.62	0.69	0.64
23	0.64	0.64	0.64	0.81	0.83	0.83	0.81	0.81	0.76	0.88	0.86	0.88	0.86	0.88	0.88	0.93	0.95	0.95	0.95	0.93	0.95	0.69	0.71	0.71
24	0.52	0.52	0.52	0.79	0.76	0.79	0.88	0.83	0.76	0.83	0.86	0.83	0.95	0.93	0.93	0.9	0.93	0.93	0.98	0.95	0.98	0.79	0.76	0.76
25	0.64	0.64	0.64	0.79	0.74	0.76	0.86	0.76	0.79	0.81	0.86	0.83	0.93	0.86	0.93	0.86	0.88	0.86	0.83	0.88	0.86	0.6	0.67	0.64
26	0.55	0.55	0.55	0.76	0.79	0.79	0.83	0.88	0.83	0.83	0.93	0.9	0.95	0.95	0.95	0.9	0.93	0.98	0.98	0.93	0.98	0.76	0.64	0.69
27	0.57	0.57	0.57	0.86	0.81	0.86	0.88	0.79	0.69	0.81	0.81	0.88	0.83	0.86	0.79	0.9	0.88	0.9	0.93	0.83	0.86	0.64	0.69	0.64
28	0.48	0.48	0.48	0.76	0.67	0.69	0.74	0.71	0.71	0.76	0.76	0.79	0.79	0.83	0.81	0.79	0.81	0.81	0.83	0.81	0.81	0.62	0.6	0.62
29	0.62	0.62	0.62	0.79	0.81	0.83	0.9	0.86	0.81	0.86	0.95	0.9	0.9	0.95	0.93	0.95	0.95	0.95	0.95	0.95	0.93	0.67	0.69	0.69
30	0.5	0.5	0.5	0.74	0.74	0.69	0.74	0.71	0.67	0.76	0.81	0.86	0.86	0.93	0.81	0.93	0.9	0.9	0.88	0.9	0.88	0.6	0.62	0.6
31	0.55	0.55	0.55	0.69	0.71	0.74	0.81	0.76	0.79	0.86	0.88	0.88	0.86	0.86	0.88	0.86	0.81	0.83	0.86	0.86	0.86	0.69	0.69	0.69
32	0.5	0.5	0.5	0.88	0.81	0.86	0.86	0.83	0.81	0.83	0.86	0.81	0.93	0.93	0.93	0.95	0.93	0.95	0.95	0.95	0.93	0.71	0.79	0.74
33	0.52	0.52	0.52	0.64	0.67	0.64	0.76	0.79	0.74	0.83	0.79	0.81	0.79	0.83	0.81	0.83	0.81	0.83	0.9	0.88	0.88	0.55	0.6	0.57
34	0.55	0.55	0.55	0.81	0.74	0.74	0.86	0.79	0.81	0.88	0.9	0.83	0.88	0.88	0.88	0.88	0.88	0.86	0.88	0.88	0.9	0.69	0.67	0.67
35	0.67	0.67	0.67	0.95	0.83	0.88	0.9	0.83	0.81	0.93	0.88	0.9	0.86	0.88	0.88	0.93	0.9	0.93	0.98	0.9	0.98	0.62	0.67	0.62
36	0.6	0.6	0.6	0.79	0.81	0.83	0.86	0.88	0.83	0.83	0.9	0.86	0.93	0.93	0.95	0.95	0.98	0.98	1	1	1	0.74	0.79	0.76
37	0.69	0.69	0.69	0.83	0.74	0.83	0.81	0.83	0.83	0.79	0.83	0.81	0.88	0.93	0.9	0.93	0.93	0.93	0.9	0.9	0.9	0.71	0.71	0.74
38	0.55	0.55	0.55	0.76	0.83	0.79	0.74	0.83	0.79	0.86	0.81	0.83	0.9	0.88	0.93	0.93	0.95	0.93	0.9	0.93	0.95	0.64	0.64	0.62
39	0.62	0.62	0.62	0.81	0.83	0.83	0.93	0.81	0.76	0.79	0.86	0.81	0.9	0.9	0.9	0.9	0.88	0.9	0.95	0.93	0.95	0.6	0.62	0.62
40	0.55	0.55	0.55	0.79	0.71	0.76	0.86	0.86	0.83	0.74	0.86	0.86	0.98	0.93	0.93	0.9	0.98	0.98	1	0.98	0.98	0.81	0.74	0.79
41	0.64	0.64	0.64	0.79	0.71	0.64	0.81	0.76	0.76	0.83	0.86	0.83	0.93	0.9	0.9	0.95	0.9	0.93	0.93	0.88	0.9	0.57	0.64	0.6
42	0.64	0.64	0.64	0.76	0.81	0.81	0.81	0.76	0.71	0.83	0.83	0.83	0.93	0.9	0.9	0.93	0.93	0.95	0.9	0.86	0.9	0.67	0.69	0.67
43	0.67	0.67	0.67	0.83	0.76	0.81	0.9	0.76	0.74	0.86	0.88	0.9	0.88	0.88	0.9	0.9	0.9	0.9	0.98	0.9	0.9	0.64	0.67	0.67
44	0.67	0.67	0.67	0.74	0.81	0.76	0.83	0.79	0.74	0.83	0.83	0.86	0.9	0.93	0.9	0.95	0.93	0.93	0.95	0.95	0.95	0.67	0.71	0.71
45	0.6	0.6	0.6	0.83	0.83	0.81	0.9	0.79	0.76	0.76	0.81	0.81	0.9	0.88	0.9	0.9	0.9	0.9	0.9	0.88	0.9	0.64	0.69	0.64
46	0.69	0.69	0.69	0.86	0.81	0.79	0.88	0.83	0.86	0.76	0.86	0.86	0.9	0.88	0.88	0.83	0.88	0.86	0.95	0.9	0.93	0.67	0.69	0.67
47	0.71	0.71	0.71	0.9	0.93	0.9	0.9	0.9	0.79	0.83	0.86	0.81	0.86	0.88	0.83	0.93	0.95	0.95	0.95	0.93	0.93	0.64	0.69	0.64
48	0.6	0.6	0.6	0.83	0.81	0.81	0.86	0.79	0.79	0.86	0.88	0.88	0.88	0.93	0.9	0.93	0.98	0.95	0.93	0.93	0.93	0.76	0.74	0.74
49	0.62	0.62	0.62	0.81	0.86	0.81	0.86	0.9	0.88	0.95	0.9	0.9	0.93	0.95	1	0.98	1	0.98	1	0.98	1	0.81	0.79	0.79
50	0.5	0.5	0.5	0.69	0.69	0.67	0.79	0.69	0.71	0.67	0.74	0.69	0.93	0.86	0.9	0.9	0.9	0.9	0.95	0.88	0.9	0.57	0.62	0.62
51	0.55	0.55	0.55	0.81	0.81	0.83	0.95	0.93	0.93	0.88	0.86	0.86	0.95	0.9	0.9	0.95	0.93	0.93	0.88	0.9	0.9	0.64	0.67	0.67
52	0.55	0.55	0.55	0.71	0.76	0.76	0.83	0.69	0.76	0.71	0.76	0.76	0.83	0.79	0.86	0.83	0.86	0.86	0.88	0.86	0.83	0.57	0.64	0.62
53	0.64	0.64	0.64	0.83	0.86	0.86	0.81	0.81	0.81	0.81	0.86	0.86	0.9	0.93	0.9	0.93	0.93	0.93	0.98	0.93	0.95	0.67	0.69	0.69
54	0.71	0.71	0.71	0.74	0.79	0.79	0.81	0.81	0.76	0.83	0.86	0.81	0.9	0.88	0.88	0.95	0.95	0.95	0.95	0.95	0.93	0.71	0.71	0.71
55	0.57	0.57	0.57	0.79	0.79	0.79	0.86	0.88	0.81	0.88	0.9	0.88	0.88	0.93	0.93	0.93	0.93	0.95	0.95	0.95	0.98	0.67	0.69	0.71
56	0.5	0.5	0.5	0.79	0.79	0.81	0.88	0.81	0.83	0.81	0.79	0.83	0.9	0.9	0.88	0.98	0.95	1	0.93	0.93	0.93	0.69	0.62	0.67
57	0.57	0.57	0.57	0.69	0.76	0.69	0.88	0.81	0.83	0.88	0.81	0.9	0.9	0.95	0.93	0.9	0.9	0.9	0.86	0.88	0.86	0.57	0.62	0.6
58	0.62	0.62	0.62	0.81	0.83	0.74	0.88	0.88	0.88	0.86	0.88	0.88	0.88	0.9	0.93	0.9	0.9	0.9	0.93	0.93	0.93	0.62	0.71	0.71
59	0.38	0.38	0.38	0.79	0.83	0.81	0.86	0.81	0.79	0.79	0.88	0.86	0.83	0.86	0.86	0.95	0.9	0.93	0.88	0.88	0.88	0.69	0.69	0.69
60	0.6	0.6	0.6	0.83	0.88	0.88	0.86	0.83	0.81	0.88	0.9	0.83	0.88	0.95	0.93	0.9	0.93	0.93	0.98	0.93	0.95	0.74	0.74	0.74
61	0.6	0.6	0.6	0.83	0.79	0.83	0.83	0.86	0.83	0.81	0.86	0.81	0.9	0.88	0.9	0.88	0.9	0.93	0.98	0.93	0.95	0.69	0.71	0.71
62	0.52	0.52	0.52	0.79	0.64	0.67	0.83	0.76	0.79	0.76	0.79	0.76	0.81	0.81	0.83	0.88	0.83	0.88	0.88	0.83	0.9	0.57	0.57	0.57
63	0.57	0.57	0.57	0.81	0.79	0.76	0.9	0.86	0.83	0.83	0.88	0.86	0.88	0.9	0.88	0.9	0.93	0.9	0.88	0.93	0.9	0.67	0.69	0.67
64	0.48	0.48	0.6	0.83	0.81	0.81	0.83	0.81	0.81	0.86	0.79	0.83	0.9	0.86	0.88	0.98	0.93	0.98	0.95	0.9	0.95	0.64	0.64	0.64
65	0.55	0.55	0.55	0.81	0.81	0.86	0.88	0.9	0.86	0.93	0.88	0.93	0.98	0.95	0.98	0.95	0.98	0.95	0.93	0.93	0.93	0.69	0.76	0.74
66	0.64	0.64	0.64	0.86	0.81	0.83	0.88	0.86	0.76	0.86	0.86	0.88	0.93	0.9	0.86	0.88	0.93	0.88	0.93	0.9	0.93	0.6	0.69	0.64
67	0.55	0.55	0.55	0.88	0.79	0.83	0.9	0.76	0.76	0.81	0.86	0.81	0.95	0.95	0.95	0.93	0.95	0.93	0.95	0.95	0.98	0.67	0.64	0.67
68	0.69	0.69	0.69	0.81	0.81	0.76	0.83	0.83	0.83	0.86	0.83	0.86	0.9	0.95	0.9	0.93	0.93	0.93	0.93	0.93	0.95	0.64	0.74	0.69
69	0.67	0.67	0.67	0.83	0.76	0.79	0.83	0.81	0.81	0.9	0.88	0.86	0.9	0.9	0.9	0.93	0.9	0.93	0.95	0.9	0.95	0.64	0.67	0.67
70	0.6	0.6	0.6	0.79	0.81	0.76	0.86	0.83	0.81	0.88	0.93	0.9	0.88	0.98	0.9	0.93	0.93	0.93	0.95	0.93	0.95	0.67	0.64	0.64
71	0.67	0.67	0.67	0.83	0.88	0.88	0.81	0.86	0.81	0.81	0.86	0.86	0.98	1	0.98	0.98	1	0.98	0.98	1	0.98	0.79	0.74	0.71
72	0.6	0.6	0.6	0.76	0.79	0.76	0.86	0.81	0.83	0.83	0.79	0.86	0.86	0.86	0.86	0.86	0.83	0.86	0.93	0.86	0.88	0.64	0.67	0.64
73	0.67	0.67	0.67	0.83	0.81	0.81	0.86	0.81	0.79	0.79	0.9	0.83	0.98	0.98	0.95	0.9	0.93	0.9	0.93	0.95	0.93	0.67	0.74	0.74
74	0.57	0.57	0.57	0.74	0.81	0.79	0.86	0.81	0.86	0.76	0.83	0.81	0.93	0.98	0.93	0.9	0.9	0.93	0.9	0.9	0.9	0.67	0.67	0.64
75	0.69	0.69	0.69	0.88	0.79	0.83	0.88	0.83	0.81	0.86	0.95	0.93	0.88	0.95	0.9	0.93	0.93	0.93	0.95	0.95	0.98	0.69	0.74	0.69
76	0.6	0.6	0.6	0.81	0.81	0.81	0.95	0.9	0.9	0.81	0.83	0.86	1	0.98	0.98	0.98	1	0.98	0.95	0.98	0.95	0.74	0.76	0.71
77	0.45	0.45	0.45	0.88	0.79	0.79	0.83	0.79	0.76	0.88	0.9	0.83	0.95	1	0.95	0.98	0.98	0.98	0.98	0.98	0.98	0.81	0.79	0.79
78	0.57	0.57	0.57	0.88	0.9	0.9	0.79	0.83	0.81	0.83	0.81	0.79	0.88	0.88	0.88	0.93	0.9	0.93	0.93	0.88	0.9	0.71	0.71	0.67
79	0.48	0.48	0.6	0.79	0.76	0.79	0.83	0.79	0.81	0.79	0.83	0.81	0.86	0.83	0.9	0.88	0.88	0.88	0.98	0.9	0.95	0.64	0.67	0.67
80	0.5	0.5	0.5	0.81	0.81	0.81	0.76	0.83	0.74	0.76	0.86	0.83	0.81	0.93	0.86	0.86	0.93	0.9	0.93	0.95	0.95	0.57	0.55	0.55
81	0.67	0.67	0.67	0.81	0.83	0.81	0.9	0.86	0.86	0.93	0.9	0.88	0.93	0.93	0.93	0.93	0.88	0.93	0.93	0.93	0.93	0.67	0.67	0.67
82	0.6	0.6	0.6	0.74	0.76	0.76	0.86	0.76	0.74	0.76	0.79	0.79	0.93	0.9	0.9	0.88	0.93	0.93	1	0.93	0.95	0.69	0.71	0.71
83	0.57	0.57	0.57	0.79	0.79	0.76	0.88	0.83	0.76	0.79	0.88	0.81	0.95	0.93	0.93	0.93	0.93	0.93	0.9	0.9	0.93	0.64	0.67	0.67
84	0.57	0.57	0.57	0.79	0.71	0.74	0.74	0.71	0.74	0.88	0.86	0.81	0.83	0.86	0.83	0.88	0.88	0.93	0.9	0.86	0.9	0.64	0.67	0.67
85	0.52	0.52	0.52	0.86	0.81	0.88	0.9	0.83	0.86	0.81	0.86	0.88	0.98	0.98	1	0.93	0.95	0.95	0.95	0.98	0.95	0.69	0.74	0.69
86	0.74	0.74	0.74	0.9	0.88	0.88	0.93	0.83	0.86	0.88	0.88	0.86	0.95	0.93	0.93	0.98	0.95	0.98	0.98	0.95	0.95	0.69	0.79	0.76
87	0.52	0.52	0.52	0.81	0.79	0.83	0.88	0.86	0.83	0.86	0.86	0.88	0.9	1	0.95	0.98	0.95	0.98	0.95	0.93	0.95	0.76	0.69	0.71
88	0.57	0.57	0.57	0.79	0.71	0.79	0.88	0.79	0.81	0.88	0.9	0.9	0.9	0.88	0.9	0.88	0.88	0.9	0.9	0.9	0.9	0.62	0.69	0.69
89	0.57	0.57	0.57	0.83	0.83	0.76	0.71	0.74	0.74	0.81	0.86	0.79	0.9	0.93	0.93	0.88	0.93	0.9	0.93	0.9	0.9	0.69	0.76	0.74
90	0.6	0.6	0.6	0.79	0.74	0.76	0.81	0.81	0.71	0.81	0.79	0.79	0.88	0.9	0.95	0.95	0.93	0.95	0.98	0.95	0.98	0.67	0.6	0.6

91	0.5	0.5	0.5	0.76	0.81	0.81	0.86	0.79	0.74	0.81	0.83	0.83	0.86	0.93	0.9	0.9	0.9	0.9	0.88	0.88	0.83	0.71	0.74	0.74
92	0.57	0.57	0.57	0.74	0.76	0.76	0.79	0.83	0.81	0.88	0.86	0.86	0.93	0.9	0.93	0.95	0.9	0.93	0.9	0.88	0.9	0.57	0.62	0.62
93	0.57	0.57	0.57	0.74	0.71	0.71	0.74	0.71	0.71	0.88	0.76	0.86	0.93	0.9	0.9	0.95	0.93	0.95	0.95	0.93	0.95	0.6	0.6	0.6
94	0.62	0.62	0.62	0.76	0.69	0.76	0.86	0.74	0.64	0.81	0.79	0.81	0.67	0.76	0.79	0.86	0.86	0.86	0.86	0.83	0.83	0.52	0.55	0.55
95	0.57	0.57	0.57	0.69	0.74	0.74	0.79	0.76	0.71	0.74	0.74	0.74	0.88	0.86	0.81	0.88	0.86	0.88	0.86	0.83	0.86	0.6	0.6	0.6
96	0.45	0.45	0.45	0.81	0.76	0.76	0.79	0.76	0.74	0.76	0.76	0.79	0.83	0.86	0.86	0.9	0.86	0.9	0.88	0.83	0.83	0.64	0.69	0.67
97	0.62	0.62	0.62	0.88	0.81	0.83	0.95	0.88	0.9	0.98	0.93	0.9	0.98	0.95	0.95	0.95	0.95	0.95	0.95	0.98	0.95	0.81	0.83	0.79
98	0.62	0.62	0.62	0.83	0.79	0.83	0.88	0.9	0.88	0.81	0.83	0.79	0.9	0.93	0.9	0.9	0.95	0.95	0.98	0.95	0.98	0.67	0.64	0.64
99	0.5	0.5	0.62	0.76	0.76	0.79	0.83	0.76	0.76	0.83	0.9	0.81	0.93	0.93	0.95	0.9	0.9	0.93	0.95	0.88	0.93	0.64	0.64	0.6
100	0.6	0.6	0.6	0.81	0.71	0.69	0.88	0.81	0.79	0.79	0.86	0.81	0.86	0.88	0.88	0.88	0.88	0.88	0.93	0.9	0.86	0.62	0.62	0.6

Table. S.13. The performance of genomic selection models with different SNPs in cross validation (continuous phenotype)

SNPs	10					52					100					500				
cv	Ridge	Lasso	ElasticNet	Random Fores	SVR	Ridge	Lasso	ElasticNet	Random Fores	SVR	Ridge	Lasso	ElasticNet	Random Fores	SVR	Ridge	Lasso	ElasticNet	Random Fores	SVR
1	0.69	0.69	0.69	0.61	0.68	0.75	0.69	0.75	0.65	0.77	0.77	0.67	0.76	0.69	0.77	0.78	0.63	0.77	0.76	0.83
2	0.64	0.64	0.64	0.61	0.64	0.82	0.71	0.81	0.74	0.80	0.82	0.72	0.82	0.78	0.80	0.76	0.73	0.87	0.77	0.88
3	0.71	0.72	0.72	0.68	0.73	0.81	0.72	0.82	0.70	0.77	0.78	0.72	0.83	0.74	0.74	0.84	0.68	0.88	0.72	0.86
4	0.76	0.76	0.76	0.72	0.78	0.83	0.76	0.84	0.77	0.77	0.80	0.77	0.83	0.80	0.78	0.82	0.78	0.85	0.80	0.85
5	0.79	0.79	0.79	0.72	0.78	0.86	0.78	0.86	0.80	0.81	0.87	0.77	0.86	0.83	0.88	0.87	0.81	0.91	0.86	0.91
6	0.76	0.76	0.76	0.77	0.77	0.71	0.67	0.71	0.70	0.65	0.79	0.68	0.80	0.79	0.77	0.82	0.73	0.87	0.82	0.88
7	0.81	0.81	0.81	0.76	0.81	0.84	0.75	0.85	0.78	0.83	0.86	0.73	0.87	0.83	0.83	0.87	0.77	0.90	0.84	0.87
8	0.67	0.64	0.68	0.61	0.67	0.79	0.74	0.81	0.72	0.77	0.83	0.74	0.84	0.75	0.81	0.76	0.73	0.85	0.76	0.84
9	0.64	0.59	0.63	0.60	0.65	0.78	0.61	0.77	0.66	0.81	0.78	0.63	0.77	0.63	0.77	0.82	0.56	0.81	0.64	0.85
10	0.72	0.74	0.74	0.64	0.72	0.76	0.68	0.79	0.75	0.76	0.77	0.68	0.81	0.78	0.76	0.82	0.69	0.90	0.84	0.87
11	0.61	0.62	0.61	0.61	0.59	0.76	0.61	0.75	0.68	0.74	0.79	0.60	0.78	0.72	0.74	0.79	0.66	0.85	0.74	0.86
12	0.83	0.82	0.83	0.83	0.83	0.84	0.76	0.87	0.83	0.82	0.86	0.75	0.89	0.85	0.83	0.68	0.80	0.89	0.90	0.86
13	0.75	0.71	0.75	0.68	0.72	0.88	0.83	0.88	0.78	0.87	0.89	0.83	0.89	0.81	0.88	0.82	0.78	0.87	0.82	0.87
14	0.66	0.66	0.66	0.54	0.66	0.70	0.70	0.70	0.62	0.71	0.76	0.71	0.77	0.72	0.70	0.75	0.67	0.84	0.77	0.83
15	0.73	0.72	0.73	0.71	0.71	0.86	0.79	0.85	0.80	0.85	0.89	0.79	0.88	0.81	0.89	0.80	0.76	0.86	0.84	0.87
16	0.81	0.80	0.81	0.76	0.80	0.83	0.82	0.86	0.80	0.80	0.85	0.83	0.86	0.83	0.84	0.88	0.79	0.90	0.85	0.89
17	0.74	0.72	0.74	0.70	0.73	0.81	0.74	0.81	0.73	0.83	0.81	0.74	0.82	0.76	0.79	0.75	0.69	0.83	0.81	0.84
18	0.70	0.70	0.70	0.66	0.69	0.68	0.65	0.68	0.65	0.65	0.73	0.66	0.74	0.71	0.67	0.79	0.64	0.80	0.74	0.75
19	0.59	0.60	0.59	0.49	0.62	0.72	0.60	0.72	0.64	0.68	0.76	0.59	0.75	0.64	0.75	0.83	0.63	0.84	0.66	0.83
20	0.72	0.74	0.74	0.63	0.69	0.84	0.73	0.84	0.84	0.82	0.85	0.73	0.85	0.85	0.83	0.77	0.70	0.85	0.84	0.86
21	0.79	0.80	0.79	0.76	0.75	0.73	0.75	0.75	0.66	0.72	0.78	0.74	0.79	0.72	0.77	0.80	0.77	0.87	0.79	0.84
22	0.78	0.79	0.79	0.69	0.78	0.82	0.79	0.83	0.76	0.80	0.81	0.81	0.84	0.79	0.80	0.88	0.73	0.89	0.80	0.91
23	0.72	0.73	0.72	0.74	0.72	0.82	0.76	0.82	0.76	0.81	0.86	0.76	0.86	0.77	0.83	0.78	0.72	0.86	0.79	0.81
24	0.69	0.69	0.70	0.67	0.70	0.82	0.75	0.82	0.78	0.79	0.86	0.77	0.88	0.86	0.84	0.88	0.75	0.91	0.83	0.93
25	0.78	0.77	0.78	0.76	0.78	0.79	0.77	0.79	0.78	0.80	0.85	0.79	0.85	0.84	0.85	0.82	0.76	0.87	0.86	0.86
26	0.65	0.66	0.66	0.59	0.69	0.75	0.71	0.78	0.74	0.69	0.82	0.72	0.83	0.74	0.77	0.80	0.66	0.83	0.80	0.88
27	0.65	0.66	0.67	0.62	0.66	0.82	0.66	0.82	0.77	0.79	0.82	0.68	0.84	0.78	0.84	0.82	0.68	0.84	0.78	0.86
28	0.62	0.57	0.62	0.56	0.59	0.73	0.58	0.77	0.70	0.70	0.86	0.58	0.84	0.75	0.85	0.87	0.56	0.87	0.72	0.89
29	0.72	0.72	0.72	0.68	0.73	0.76	0.69	0.78	0.74	0.73	0.84	0.70	0.84	0.76	0.85	0.78	0.68	0.82	0.78	0.83
30	0.69	0.70	0.69	0.68	0.69	0.77	0.62	0.76	0.70	0.73	0.83	0.63	0.84	0.78	0.80	0.82	0.72	0.90	0.81	0.88
31	0.60	0.49	0.60	0.55	0.55	0.79	0.66	0.78	0.62	0.74	0.72	0.64	0.73	0.62	0.72	0.81	0.57	0.82	0.61	0.80
32	0.74	0.73	0.74	0.68	0.75	0.87	0.79	0.88	0.84	0.81	0.84	0.80	0.90	0.86	0.81	0.86	0.73	0.92	0.87	0.91
33	0.67	0.66	0.67	0.59	0.65	0.77	0.67	0.76	0.67	0.75	0.75	0.68	0.78	0.68	0.73	0.81	0.65	0.85	0.64	0.84
34	0.69	0.68	0.69	0.65	0.69	0.77	0.72	0.78	0.78	0.73	0.79	0.73	0.82	0.77	0.76	0.79	0.75	0.83	0.82	0.86
35	0.74	0.72	0.74	0.62	0.74	0.83	0.75	0.84	0.80	0.82	0.83	0.73	0.84	0.79	0.79	0.81	0.72	0.85	0.82	0.86
36	0.81	0.81	0.81	0.82	0.81	0.81	0.73	0.81	0.79	0.81	0.86	0.72	0.86	0.82	0.85	0.84	0.74	0.90	0.82	0.90
37	0.66	0.67	0.66	0.59	0.68	0.69	0.66	0.70	0.66	0.69	0.73	0.68	0.75	0.71	0.70	0.76	0.69	0.79	0.75	0.80
38	0.75	0.74	0.75	0.70	0.76	0.85	0.77	0.86	0.81	0.83	0.84	0.78	0.86	0.82	0.81	0.83	0.73	0.85	0.80	0.86
39	0.69	0.70	0.69	0.64	0.69	0.77	0.67	0.77	0.72	0.75	0.79	0.68	0.79	0.72	0.78	0.82	0.70	0.84	0.77	0.83
40	0.69	0.75	0.71	0.63	0.65	0.76	0.76	0.79	0.78	0.74	0.80	0.77	0.82	0.81	0.79	0.81	0.77	0.87	0.81	0.88
41	0.58	0.54	0.58	0.54	0.57	0.79	0.74	0.79	0.75	0.78	0.86	0.73	0.86	0.83	0.84	0.83	0.65	0.84	0.77	0.87
42	0.55	0.54	0.55	0.51	0.57	0.85	0.78	0.85	0.80	0.82	0.85	0.76	0.85	0.79	0.82	0.80	0.73	0.86	0.78	0.88
43	0.79	0.80	0.81	0.68	0.76	0.76	0.75	0.78	0.76	0.71	0.81	0.72	0.83	0.78	0.76	0.85	0.84	0.90	0.84	0.88
44	0.71	0.68	0.71	0.67	0.71	0.81	0.71	0.80	0.71	0.83	0.83	0.70	0.84	0.72	0.81	0.80	0.64	0.84	0.75	0.85
45	0.81	0.81	0.82	0.79	0.82	0.90	0.77	0.90	0.85	0.90	0.91	0.79	0.90	0.88	0.90	0.83	0.77	0.88	0.86	0.89
46	0.57	0.55	0.58	0.59	0.57	0.74	0.52	0.72	0.61	0.75	0.81	0.53	0.78	0.69	0.81	0.75	0.64	0.82	0.76	0.82
47	0.83	0.84	0.83	0.77	0.84	0.80	0.81	0.82	0.77	0.77	0.87	0.83	0.89	0.83	0.86	0.88	0.80	0.91	0.84	0.90
48	0.76	0.77	0.77	0.67	0.76	0.79	0.71	0.81	0.76	0.78	0.84	0.74	0.85	0.79	0.81	0.77	0.72	0.83	0.83	0.85
49	0.77	0.77	0.77	0.71	0.77	0.72	0.69	0.74	0.75	0.68	0.83	0.68	0.82	0.84	0.80	0.84	0.68	0.87	0.79	0.87
50	0.72	0.71	0.72	0.66	0.72	0.79	0.74	0.79	0.78	0.77	0.81	0.71	0.82	0.80	0.78	0.83	0.75	0.87	0.82	0.88
51	0.74	0.74	0.74	0.68	0.72	0.84	0.78	0.84	0.84	0.84	0.87	0.79	0.88	0.84	0.85	0.82	0.80	0.86	0.84	0.87
52	0.77	0.75	0.77	0.64	0.77	0.81	0.73	0.81	0.72	0.80	0.83	0.70	0.83	0.75	0.80	0.81	0.68	0.85	0.77	0.89
53	0.72	0.71	0.72	0.64	0.71	0.81	0.63	0.81	0.80	0.83	0.87	0.64	0.87	0.82	0.86	0.85	0.68	0.90	0.86	0.89
54	0.73	0.72	0.73	0.61	0.73	0.77	0.69	0.78	0.76	0.78	0.78	0.70	0.79	0.74	0.78	0.71	0.68	0.79	0.76	0.80
55	0.79	0.78	0.79	0.71	0.79	0.85	0.86	0.86	0.81	0.80	0.86	0.85	0.86	0.84	0.83	0.84	0.84	0.89	0.85	0.89
56	0.73	0.73	0.73	0.69	0.73	0.76	0.66	0.79	0.76	0.72	0.77	0.67	0.81	0.77	0.77	0.76	0.65	0.87	0.83	0.84
57	0.64	0.61	0.64	0.59	0.63	0.75	0.58	0.75	0.65	0.76	0.76	0.59	0.77	0.66	0.72	0.80	0.59	0.83	0.75	0.85
58	0.69	0.67	0.69	0.66	0.68	0.84	0.78	0.84	0.74	0.84	0.86	0.78	0.87	0.79	0.84	0.77	0.75	0.85	0.82	0.84
59	0.68	0.68	0.69	0.63	0.68	0.83	0.76	0.84	0.79	0.80	0.85	0.76	0.88	0.83	0.83	0.84	0.78	0.88	0.86	0.88
60	0.78	0.78	0.79	0.76	0.77	0.84	0.75	0.87	0.86	0.86	0.88	0.78	0.90	0.87	0.86	0.87	0.82	0.91	0.91	0.92
61	0.66	0.65	0.66	0.67	0.66	0.71	0.55	0.71	0.64	0.71	0.74	0.54	0.74	0.65	0.70	0.69	0.53	0.78	0.75	0.79
62	0.65	0.61	0.65	0.59	0.63	0.80	0.74	0.80	0.69	0.82	0.81	0.76	0.81	0.77	0.76	0.81	0.72	0.85	0.76	0.84
63	0.74	0.74	0.74	0.69	0.74	0.74	0.74	0.75	0.77	0.74	0.78	0.73	0.78	0.79	0.77	0.82	0.70	0.85	0.76	0.85
64	0.67	0.66	0.67	0.61	0.68	0.81	0.72	0.80	0.78	0.76	0.90	0.74	0.89	0.83	0.85	0.77	0.70	0.85	0.73	0.82
65	0.64	0.60	0.64	0.60	0.65	0.79	0.76	0.81	0.74	0.75	0.84	0.77	0.83	0.79	0.80	0.86	0.70	0.89	0.80	0.88
66	0.74	0.68	0.74	0.74	0.71	0.87	0.73	0.86	0.78	0.85	0.89	0.73	0.89	0.82	0.84	0.83	0.73	0.86	0.82	0.90
67	0.74	0.70	0.74	0.68	0.73	0.82	0.74	0.83	0.77	0.82	0.87	0.75	0.89	0.83	0.84	0.86	0.74	0.88	0.81	0.88
68	0.51	0.43	0.51	0.49	0.52	0.73	0.53	0.72	0.67	0.71	0.77	0.54	0.74	0.71	0.74	0.78	0.54	0.80	0.76	0.79
69	0.69	0.67	0.69	0.66	0.69	0.82	0.69	0.82	0.78	0.81	0.87	0.69	0.88	0.81	0.84	0.79	0.56	0.81	0.68	0.81
70	0.79	0.77	0.79	0.73	0.79	0.88	0.78	0.88	0.78	0.84	0.92	0.77	0.90	0.83	0.91	0.80	0.67	0.87	0.83	0.89
71	0.69	0.68	0.69	0.67	0.70	0.82	0.79	0.83	0.81	0.83	0.79	0.78	0.80	0.82	0.75	0.83	0.77	0.90	0.83	0.89
72	0.60	0.57	0.60	0.52	0.60	0.82	0.67	0.81	0.72	0.80	0.84	0.67	0.82	0.71	0.80	0.82	0.71	0.88	0.70	0.88
73	0.55	0.58	0.56	0.50	0.54	0.74	0.67	0.78	0.69	0.72	0.81	0.67	0.84	0.75	0.82	0.82	0.63	0.84	0.81	0.84
74	0.66	0.65	0.66	0.64	0.65	0.76	0.70	0.76	0.74	0.76	0.83	0.71	0.82	0.77	0.84	0.81	0.66	0.88	0.77	0.87
75	0.79	0.79	0.80	0.76	0.79	0.85	0.83	0.85	0.85	0.82	0.81	0.83	0.84	0.86	0.80	0.89	0.82	0.90	0.87	0.91
76	0.82	0.83	0.82	0.75	0.81	0.85	0.75	0.85	0.84	0.85	0.87	0.75	0.89	0.87	0.83	0.90	0.79	0.91	0.85	0.92
77	0.82	0.80	0.82	0.74	0.82	0.81	0.78	0.81	0.79	0.84	0.80	0.74	0.82	0.82	0.78	0.86	0.81	0.91	0.88	0.90
78	0.69	0.69	0.69	0.64	0.70	0.79	0.74	0.78	0.78	0.76	0.84	0.75	0.84	0.79	0.83	0.77	0.70	0.86	0.81	0.86
79	0.70	0.71	0.71	0.61	0.69	0.79	0.71	0.78	0.77	0.75	0.80	0.71	0.80	0.76	0.78	0.71	0.64	0.81	0.75	0.80
80	0.60	0.61	0.61	0.52	0.63	0.76	0.62	0.78	0.76	0.73	0.79	0.62	0.81	0.76	0.77	0.81	0.61	0.83	0.73	0.85
81	0.76	0.74	0.76	0.73	0.72	0.80	0.78	0.82	0.72	0.80	0.82	0.78	0.85	0.76	0.79	0.84	0.71	0.87	0.78	0.85
82	0.75	0.75	0.75	0.66	0.74	0.78	0.69	0.79	0.75	0.72	0.81	0.70	0.82	0.76	0.79	0.84	0.71	0.89	0.82	0.88
83	0.70	0.67	0.70	0.69	0.69	0.79	0.64	0.79	0.71	0.79	0.79	0.64	0.79	0.71	0.79	0.79	0.69	0.82	0.74	0.82
84	0.78	0.77	0.78	0.75	0.78	0.81	0.73	0.80	0.71	0.81	0.81	0.73	0.81	0.79	0.78	0.83	0.69	0.85	0.83	0.86
85	0.69	0.68	0.69	0.52	0.68	0.78	0.73	0.80	0.79	0.77	0.79	0.71	0.80	0.82	0.78	0.88	0.71	0.90	0.84	0.90
86	0.77	0.76	0.77	0.73	0.77	0.79	0.68	0.80	0.80	0.79	0.80	0.67	0.82	0.80	0.80	0.81	0.71	0.88	0.82	0.85
87	0.75	0.75	0.75	0.68	0.76	0.85	0.77	0.85	0.76	0.84	0.82	0.72	0.83	0.77	0.81	0.78	0.75	0.85	0.81	0.88
88	0.81	0.81	0.81	0.68	0.81	0.82	0.86	0.82	0.83	0.77	0.82	0.85	0.84	0.84	0.81	0.74	0.82	0.83	0.86	0.81
89	0.76	0.76	0.76	0.73	0.76	0.88	0.77	0.88	0.75	0.85	0.82	0.73	0.83	0.77	0.79	0.84	0.72	0.87	0.80	0.88
90	0.63	0.63	0.63	0.56	0.64	0.78	0.72	0.79	0.75	0.74	0.80	0.71	0.81	0.76	0.74	0.72	0.68	0.87	0.79	0.84
91	0.76	0.77	0.76	0.64	0.75	0.83	0.83	0.84	0.80	0.84	0.82	0.80	0.83	0.76	0.83	0.80	0.83	0.87	0.80	0.88
92	0.64	0.64	0.66	0.62	0.64	0.78	0.71	0.79	0.74	0.79	0.82	0.72	0.83	0.80	0.79	0.80	0.72	0.83	0.82	0.81
93	0.62	0.63	0.63	0.58	0.65	0.79	0.75	0.79	0.73	0.78	0.77	0.74	0.78	0.76	0.77	0.85	0.70	0.86	0.71	0.87
94	0.68	0.70	0.69	0.61	0.65	0.78	0.75	0.79	0.71	0.75	0.80	0.76	0.84	0.80	0.79	0.79	0.69	0.83	0.74	0.85
95	0.71	0.70	0.71	0.69	0.71	0.83	0.75	0.82	0.78	0.81	0.82	0.75	0.81	0.78	0.81	0.81	0.65	0.85	0.78	0.84
96	0.68	0.66	0.69	0.63	0.66	0.76	0.67	0.80	0.71	0.75	0.81	0.66	0.86	0.72	0.79	0.87	0.72	0.90	0.76	0.86
97	0.85	0.83	0.84	0.81	0.84	0.92	0.86	0.93	0.90	0.92	0.90	0.86	0.92	0.93	0.89	0.91	0.91	0.92	0.93	0.93
98	0.74	0.72	0.74	0.67	0.75	0.83	0.79	0.83	0.76	0.82	0.88	0.80	0.88	0.84	0.87	0.83	0.78	0.89	0.85	0.90
99	0.65	0.62	0.65	0.57	0.66	0.77	0.69	0.76	0.78	0.76	0.84	0.68	0.83	0.78	0.82	0.78	0.72	0.85	0.80	0.86
100	0.63	0.64	0.64	0.56	0.63	0.72	0.59	0.74	0.71	0.74	0.75	0.60	0.76	0.70	0.74	0.66	0.53	0.77	0.63	0.78

SNPs	2000					5000					10000					All				
cv	Ridge	Lasso	ElasticNet	Random Fores	SVR	Ridge	Lasso	ElasticNet	Random Fores	SVR	Ridge	Lasso	ElasticNet	Random Fores	SVR	Ridge	Lasso	ElasticNet	Random Fores	SVR
1	0.73	0.61	0.86	0.72	0.86	0.79	0.59	0.83	0.73	0.85	0.84	0.55	0.85	0.73	0.87	0.44	0.49	0.49	0.49	0.53
2	0.73	0.68	0.85	0.76	0.88	0.85	0.63	0.86	0.78	0.91	0.91	0.64	0.90	0.75	0.93	0.49	0.49	0.63	0.56	0.49
3	0.64	0.70	0.88	0.76	0.90	0.88	0.61	0.87	0.74	0.89	0.90	0.66	0.88	0.74	0.92	0.51	0.56	0.50	0.55	0.55
4	0.61	0.75	0.87	0.79	0.88	0.82	0.67	0.87	0.83	0.89	0.91	0.73	0.91	0.82	0.92	0.49	0.51	0.74	0.66	0.60
5	0.71	0.76	0.92	0.83	0.92	0.91	0.69	0.91	0.81	0.93	0.91	0.68	0.90	0.80	0.94	0.43	0.39	0.51	0.55	0.51
6	0.70	0.71	0.91	0.78	0.92	0.90	0.57	0.91	0.79	0.91	0.91	0.67	0.89	0.76	0.93	0.45	0.43	0.60	0.42	0.49
7	0.60	0.80	0.90	0.82	0.93	0.90	0.73	0.93	0.85	0.94	0.91	0.73	0.91	0.85	0.93	0.61	0.57	0.49	0.59	0.61
8	0.82	0.68	0.88	0.73	0.89	0.87	0.59	0.86	0.77	0.89	0.87	0.54	0.88	0.72	0.89	0.35	0.45	0.45	0.46	0.38
9	0.66	0.58	0.82	0.56	0.86	0.85	0.51	0.80	0.51	0.87	0.86	0.50	0.84	0.54	0.88	0.29	0.27	0.50	0.27	0.34
10	0.61	0.67	0.89	0.83	0.91	0.90	0.68	0.92	0.82	0.93	0.90	0.65	0.88	0.82	0.93	0.55	0.48	0.57	0.61	0.58
11	0.71	0.72	0.90	0.75	0.92	0.91	0.55	0.88	0.77	0.92	0.90	0.60	0.88	0.74	0.93	0.41	0.39	0.44	0.44	0.42
12	0.77	0.82	0.95	0.88	0.95	0.92	0.76	0.92	0.89	0.95	0.92	0.75	0.91	0.88	0.95	0.62	0.60	0.50	0.73	0.63
13	0.62	0.78	0.88	0.80	0.90	0.85	0.74	0.86	0.81	0.91	0.88	0.73	0.89	0.82	0.92	0.53	0.52	0.39	0.57	0.56
14	0.66	0.67	0.84	0.73	0.86	0.83	0.58	0.82	0.72	0.88	0.88	0.60	0.89	0.77	0.90	0.42	0.48	0.66	0.47	0.48
15	0.64	0.71	0.90	0.81	0.90	0.85	0.63	0.90	0.81	0.91	0.89	0.65	0.88	0.83	0.92	0.60	0.56	0.45	0.63	0.60
16	0.75	0.77	0.88	0.84	0.92	0.89	0.69	0.90	0.86	0.93	0.89	0.68	0.90	0.83	0.93	0.56	0.61	0.32	0.62	0.57
17	0.64	0.67	0.88	0.80	0.91	0.84	0.62	0.83	0.79	0.88	0.88	0.61	0.85	0.76	0.90	0.38	0.47	0.58	0.52	0.47
18	0.60	0.66	0.85	0.74	0.88	0.84	0.61	0.87	0.71	0.89	0.83	0.55	0.80	0.70	0.88	0.42	0.44	0.65	0.52	0.44
19	0.79	0.63	0.83	0.62	0.89	0.85	0.55	0.82	0.66	0.88	0.89	0.55	0.85	0.67	0.89	0.36	0.36	0.64	0.33	0.34
20	0.77	0.72	0.90	0.82	0.93	0.88	0.68	0.90	0.82	0.92	0.91	0.68	0.91	0.82	0.94	0.53	0.58	0.70	0.55	0.59
21	0.68	0.73	0.82	0.77	0.85	0.88	0.69	0.83	0.77	0.87	0.90	0.70	0.90	0.77	0.90	0.51	0.59	0.37	0.63	0.54
22	0.73	0.72	0.88	0.79	0.90	0.88	0.71	0.87	0.80	0.91	0.89	0.66	0.87	0.77	0.91	0.51	0.51	0.49	0.56	0.55
23	0.63	0.69	0.87	0.79	0.89	0.81	0.58	0.84	0.78	0.89	0.85	0.58	0.88	0.80	0.91	0.55	0.52	0.59	0.65	0.58
24	0.67	0.71	0.90	0.84	0.93	0.90	0.61	0.88	0.84	0.93	0.90	0.70	0.89	0.85	0.93	0.54	0.50	0.31	0.57	0.60
25	0.71	0.74	0.89	0.81	0.89	0.90	0.69	0.87	0.81	0.89	0.89	0.74	0.88	0.81	0.89	0.50	0.64	0.63	0.63	0.57
26	0.73	0.67	0.87	0.81	0.90	0.91	0.56	0.91	0.79	0.93	0.91	0.61	0.91	0.80	0.93	0.47	0.47	0.66	0.41	0.47
27	0.83	0.60	0.88	0.80	0.90	0.88	0.69	0.89	0.78	0.91	0.90	0.63	0.86	0.76	0.92	0.46	0.50	0.60	0.57	0.52
28	0.82	0.55	0.83	0.72	0.85	0.86	0.49	0.82	0.72	0.87	0.87	0.46	0.84	0.69	0.86	0.41	0.39	0.63	0.41	0.42
29	0.77	0.65	0.86	0.77	0.90	0.88	0.61	0.90	0.77	0.89	0.90	0.59	0.91	0.72	0.92	0.41	0.52	0.59	0.46	0.48
30	0.74	0.75	0.90	0.81	0.92	0.88	0.66	0.86	0.82	0.91	0.91	0.62	0.90	0.82	0.92	0.57	0.49	0.60	0.56	0.60
31	0.66	0.59	0.82	0.70	0.84	0.82	0.57	0.84	0.70	0.86	0.86	0.54	0.87	0.69	0.88	0.49	0.50	0.58	0.45	0.50
32	0.74	0.75	0.93	0.84	0.94	0.93	0.72	0.90	0.86	0.95	0.93	0.69	0.90	0.84	0.96	0.59	0.54	0.56	0.61	0.64
33	0.70	0.64	0.88	0.64	0.88	0.84	0.56	0.87	0.67	0.86	0.86	0.58	0.87	0.67	0.88	0.38	0.48	0.30	0.43	0.43
34	0.64	0.66	0.85	0.81	0.89	0.85	0.60	0.85	0.78	0.88	0.90	0.57	0.88	0.76	0.91	0.44	0.51	0.46	0.50	0.49
35	0.71	0.76	0.89	0.80	0.90	0.83	0.65	0.86	0.82	0.91	0.89	0.63	0.87	0.77	0.93	0.49	0.47	0.65	0.50	0.50
36	0.67	0.69	0.90	0.80	0.93	0.90	0.63	0.89	0.83	0.94	0.92	0.70	0.94	0.83	0.96	0.52	0.53	0.44	0.51	0.53
37	0.66	0.71	0.85	0.75	0.85	0.81	0.50	0.83	0.76	0.86	0.84	0.46	0.83	0.73	0.88	0.42	0.47	0.59	0.47	0.49
38	0.73	0.69	0.88	0.80	0.89	0.87	0.68	0.85	0.79	0.90	0.88	0.65	0.88	0.78	0.91	0.44	0.56	0.40	0.51	0.52
39	0.73	0.68	0.86	0.78	0.88	0.85	0.65	0.84	0.78	0.87	0.85	0.67	0.81	0.79	0.89	0.55	0.56	0.71	0.53	0.57
40	0.78	0.74	0.88	0.82	0.93	0.90	0.68	0.89	0.84	0.93	0.92	0.73	0.92	0.83	0.95	0.69	0.70	0.60	0.61	0.69
41	0.78	0.69	0.88	0.80	0.89	0.85	0.59	0.82	0.80	0.89	0.89	0.59	0.89	0.75	0.91	0.48	0.46	0.54	0.51	0.50
42	0.61	0.70	0.87	0.80	0.89	0.85	0.58	0.89	0.76	0.90	0.86	0.56	0.86	0.79	0.91	0.34	0.34	0.50	0.34	0.40
43	0.65	0.80	0.86	0.80	0.87	0.85	0.70	0.86	0.82	0.91	0.87	0.71	0.83	0.81	0.91	0.54	0.64	0.69	0.66	0.65
44	0.69	0.70	0.85	0.80	0.89	0.86	0.63	0.86	0.79	0.91	0.91	0.63	0.89	0.78	0.93	0.45	0.53	0.62	0.48	0.53
45	0.71	0.77	0.88	0.85	0.91	0.91	0.75	0.90	0.87	0.92	0.91	0.74	0.90	0.87	0.94	0.60	0.58	0.58	0.61	0.67
46	0.72	0.63	0.85	0.76	0.89	0.84	0.53	0.86	0.71	0.88	0.89	0.56	0.85	0.72	0.91	0.38	0.43	0.34	0.48	0.46
47	0.64	0.75	0.86	0.84	0.92	0.89	0.66	0.88	0.86	0.91	0.91	0.63	0.88	0.83	0.92	0.50	0.51	0.68	0.63	0.59
48	0.69	0.66	0.87	0.85	0.91	0.89	0.61	0.90	0.87	0.93	0.91	0.61	0.89	0.83	0.94	0.51	0.51	0.47	0.57	0.55
49	0.74	0.77	0.90	0.80	0.91	0.83	0.72	0.88	0.81	0.90	0.89	0.72	0.88	0.80	0.92	0.52	0.53	0.61	0.58	0.57
50	0.73	0.71	0.85	0.79	0.90	0.87	0.60	0.85	0.83	0.90	0.89	0.63	0.87	0.78	0.91	0.48	0.52	0.60	0.48	0.52
51	0.67	0.66	0.86	0.83	0.87	0.87	0.65	0.87	0.81	0.90	0.90	0.69	0.91	0.82	0.92	0.51	0.50	0.59	0.58	0.58
52	0.76	0.70	0.84	0.78	0.88	0.84	0.63	0.87	0.77	0.88	0.87	0.62	0.88	0.79	0.90	0.49	0.50	0.38	0.49	0.53
53	0.79	0.74	0.87	0.85	0.92	0.93	0.70	0.93	0.86	0.95	0.93	0.68	0.92	0.82	0.96	0.64	0.56	0.52	0.56	0.62
54	0.66	0.68	0.89	0.78	0.90	0.83	0.58	0.85	0.77	0.89	0.86	0.54	0.85	0.76	0.91	0.37	0.39	0.54	0.48	0.44
55	0.75	0.82	0.90	0.85	0.91	0.93	0.70	0.91	0.89	0.93	0.92	0.76	0.93	0.86	0.94	0.66	0.67	0.56	0.68	0.68
56	0.71	0.66	0.91	0.81	0.92	0.87	0.60	0.88	0.80	0.92	0.89	0.56	0.88	0.79	0.92	0.52	0.40	0.62	0.50	0.52
57	0.68	0.63	0.83	0.75	0.86	0.85	0.61	0.88	0.78	0.90	0.86	0.52	0.86	0.75	0.89	0.41	0.44	0.51	0.45	0.44
58	0.66	0.75	0.84	0.82	0.86	0.84	0.69	0.85	0.78	0.89	0.88	0.64	0.87	0.80	0.90	0.48	0.48	0.37	0.56	0.52
59	0.69	0.77	0.90	0.83	0.92	0.86	0.64	0.88	0.86	0.92	0.88	0.68	0.86	0.84	0.93	0.62	0.64	0.51	0.67	0.65
60	0.78	0.80	0.92	0.90	0.94	0.87	0.75	0.91	0.89	0.94	0.94	0.73	0.92	0.89	0.96	0.68	0.64	0.61	0.72	0.71
61	0.69	0.59	0.86	0.72	0.87	0.84	0.56	0.87	0.74	0.88	0.88	0.51	0.86	0.72	0.91	0.40	0.45	0.54	0.40	0.41
62	0.73	0.72	0.87	0.73	0.88	0.87	0.64	0.87	0.74	0.88	0.89	0.69	0.91	0.76	0.91	0.51	0.52	0.52	0.48	0.50
63	0.71	0.70	0.84	0.76	0.87	0.85	0.60	0.83	0.77	0.87	0.88	0.65	0.87	0.79	0.91	0.41	0.53	0.63	0.56	0.45
64	0.66	0.67	0.84	0.72	0.86	0.80	0.58	0.81	0.76	0.87	0.87	0.59	0.89	0.76	0.89	0.51	0.54	0.43	0.52	0.53
65	0.71	0.70	0.88	0.82	0.92	0.90	0.67	0.89	0.76	0.92	0.90	0.67	0.89	0.78	0.92	0.52	0.53	0.47	0.57	0.56
66	0.77	0.74	0.87	0.74	0.90	0.81	0.63	0.85	0.80	0.89	0.88	0.67	0.90	0.76	0.91	0.52	0.48	0.70	0.49	0.51
67	0.77	0.72	0.89	0.79	0.90	0.86	0.64	0.88	0.82	0.90	0.89	0.68	0.88	0.81	0.91	0.53	0.49	0.64	0.57	0.54
68	0.71	0.60	0.82	0.80	0.88	0.90	0.53	0.87	0.76	0.91	0.91	0.48	0.91	0.74	0.92	0.43	0.44	0.38	0.50	0.50
69	0.73	0.60	0.85	0.72	0.88	0.92	0.55	0.91	0.76	0.91	0.92	0.47	0.91	0.75	0.92	0.33	0.30	0.52	0.39	0.41
70	0.77	0.73	0.90	0.81	0.91	0.90	0.65	0.90	0.78	0.93	0.91	0.69	0.91	0.79	0.94	0.51	0.46	0.55	0.51	0.53
71	0.63	0.74	0.86	0.83	0.88	0.91	0.68	0.90	0.81	0.94	0.88	0.66	0.87	0.79	0.92	0.41	0.46	0.39	0.53	0.47
72	0.75	0.71	0.89	0.70	0.91	0.90	0.62	0.88	0.70	0.91	0.90	0.63	0.92	0.72	0.92	0.43	0.46	0.62	0.42	0.44
73	0.79	0.64	0.85	0.79	0.88	0.84	0.57	0.88	0.79	0.88	0.86	0.61	0.86	0.80	0.89	0.43	0.46	0.45	0.47	0.47
74	0.72	0.66	0.87	0.77	0.91	0.86	0.56	0.89	0.76	0.88	0.87	0.60	0.85	0.73	0.89	0.45	0.44	0.61	0.51	0.46
75	0.77	0.81	0.90	0.85	0.92	0.86	0.75	0.90	0.85	0.92	0.91	0.77	0.89	0.86	0.94	0.57	0.63	0.49	0.73	0.65
76	0.76	0.81	0.89	0.86	0.94	0.90	0.74	0.89	0.86	0.94	0.92	0.76	0.92	0.87	0.95	0.64	0.70	0.51	0.70	0.68
77	0.76	0.78	0.87	0.86	0.93	0.88	0.68	0.91	0.87	0.93	0.94	0.71	0.92	0.83	0.94	0.53	0.61	0.57	0.63	0.57
78	0.69	0.69	0.86	0.80	0.90	0.88	0.69	0.89	0.81	0.92	0.91	0.65	0.90	0.81	0.92	0.52	0.59	0.48	0.53	0.57
79	0.61	0.62	0.81	0.74	0.85	0.82	0.61	0.81	0.74	0.87	0.87	0.61	0.87	0.74	0.89	0.53	0.53	0.53	0.53	0.56
80	0.77	0.56	0.86	0.73	0.90	0.90	0.46	0.88	0.73	0.90	0.91	0.54	0.91	0.75	0.92	0.27	0.19	0.49	0.27	0.31
81	0.70	0.73	0.86	0.80	0.89	0.89	0.68	0.88	0.84	0.92	0.89	0.71	0.88	0.82	0.91	0.63	0.61	0.62	0.66	0.67
82	0.66	0.74	0.90	0.81	0.91	0.87	0.67	0.86	0.83	0.90	0.88	0.67	0.86	0.81	0.91	0.53	0.54	0.54	0.55	0.61
83	0.71	0.69	0.84	0.72	0.86	0.85	0.59	0.84	0.75	0.89	0.88	0.63	0.89	0.73	0.91	0.39	0.44	0.55	0.49	0.49
84	0.69	0.69	0.86	0.83	0.90	0.86	0.70	0.86	0.85	0.90	0.89	0.66	0.89	0.83	0.92	0.56	0.58	0.66	0.62	0.64
85	0.68	0.73	0.87	0.80	0.90	0.88	0.64	0.89	0.83	0.91	0.90	0.66	0.90	0.79	0.92	0.56	0.59	0.55	0.58	0.58
86	0.61	0.72	0.90	0.83	0.94	0.87	0.71	0.91	0.82	0.92	0.88	0.67	0.86	0.81	0.93	0.43	0.48	0.56	0.54	0.52
87	0.71	0.73	0.87	0.79	0.89	0.86	0.72	0.86	0.80	0.90	0.89	0.70	0.84	0.75	0.91	0.56	0.54	0.61	0.63	0.58
88	0.74	0.84	0.89	0.85	0.90	0.87	0.75	0.90	0.84	0.91	0.90	0.77	0.90	0.85	0.92	0.58	0.62	0.41	0.73	0.64
89	0.64	0.68	0.86	0.77	0.89	0.91	0.70	0.89	0.80	0.92	0.91	0.63	0.88	0.81	0.92	0.47	0.49	0.51	0.56	0.56
90	0.71	0.69	0.87	0.78	0.90	0.86	0.60	0.89	0.75	0.92	0.88	0.59	0.88	0.72	0.93	0.39	0.37	0.60	0.37	0.43
91	0.75	0.78	0.88	0.80	0.90	0.89	0.72	0.88	0.83	0.91	0.89	0.76	0.89	0.83	0.93	0.62	0.63	0.54	0.64	0.65
92	0.74	0.68	0.85	0.76	0.87	0.86	0.59	0.88	0.77	0.89	0.89	0.62	0.87	0.73	0.90	0.35	0.40	0.51	0.46	0.43
93	0.79	0.70	0.90	0.74	0.92	0.91	0.62	0.90	0.73	0.93	0.93	0.67	0.90	0.73	0.95	0.45	0.45	0.39	0.43	0.46
94	0.68	0.72	0.84	0.78	0.87	0.81	0.61	0.82	0.79	0.87	0.84	0.65	0.84	0.76	0.89	0.45	0.56	0.66	0.61	0.56
95	0.77	0.61	0.86	0.75	0.89	0.89	0.57	0.87	0.75	0.90	0.90	0.62	0.89	0.74	0.91	0.54	0.53	0.57	0.53	0.57
96	0.76	0.71	0.85	0.74	0.86	0.82	0.61	0.83	0.73	0.86	0.86	0.57	0.83	0.75	0.88	0.52	0.49	0.62	0.55	0.60
97	0.78	0.84	0.94	0.91	0.96	0.94	0.77	0.94	0.94	0.96	0.93	0.80	0.93	0.94	0.97	0.70	0.70	0.54	0.77	0.73
98	0.74	0.65	0.88	0.81	0.90	0.89	0.64	0.90	0.84	0.92	0.91	0.64	0.90	0.82	0.93	0.48	0.46	0.53	0.56	0.53
99	0.75	0.71	0.89	0.80	0.91	0.86	0.62	0.88	0.82	0.90	0.89	0.62	0.90	0.81	0.91	0.50	0.57	0.59	0.53	0.57
100	0.67	0.57	0.78	0.66	0.85	0.85	0.48	0.85	0.61	0.86	0.87	0.53	0.83	0.64	0.89	0.23	0.30	0.45	0.32	0.30

SNPs	10			52			100			500			2000			5000			10000			All		
cv	SVR_linear	SVR_poly	SVR_rbf	SVR_linear	SVR_poly	SVR_rbf	SVR_linear	SVR_poly	SVR_rbf	SVR_linear	SVR_poly	SVR_rbf	SVR_linear	SVR_poly	SVR_rbf	SVR_linear	SVR_poly	SVR_rbf	SVR_linear	SVR_poly	SVR_rbf	SVR_linear	SVR_poly	SVR_rbf
1	0.68	0.51	0.63	0.77	0.58	0.51	0.77	0.68	0.66	0.81	0.84	0.83	0.79	0.85	0.86	0.82	0.85	0.85	0.85	0.85	0.87	0.46	0.53	0.48
2	0.64	0.55	0.63	0.80	0.55	0.56	0.80	0.64	0.68	0.77	0.83	0.88	0.81	0.88	0.88	0.88	0.89	0.91	0.91	0.91	0.93	0.49	0.49	0.48
3	0.73	0.55	0.67	0.77	0.61	0.62	0.74	0.73	0.75	0.80	0.85	0.86	0.72	0.90	0.90	0.88	0.90	0.89	0.90	0.91	0.92	0.51	0.55	0.53
4	0.78	0.51	0.66	0.77	0.76	0.72	0.78	0.66	0.70	0.80	0.84	0.85	0.74	0.90	0.88	0.85	0.90	0.89	0.92	0.92	0.92	0.51	0.60	0.55

5	0.78	0.52	0.70	0.81	0.64	0.72	0.88	0.78	0.77	0.86	0.87	0.91	0.77	0.92	0.92	0.92	0.93	0.93	0.92	0.93	0.94	0.44	0.51	0.48
6	0.77	0.63	0.76	0.65	0.59	0.59	0.77	0.74	0.74	0.79	0.87	0.88	0.82	0.92	0.92	0.92	0.91	0.91	0.92	0.92	0.93	0.47	0.49	0.49
7	0.81	0.38	0.71	0.83	0.63	0.66	0.83	0.77	0.77	0.87	0.88	0.87	0.78	0.90	0.93	0.92	0.93	0.94	0.92	0.92	0.93	0.61	0.61	0.60
8	0.67	0.32	0.51	0.77	0.53	0.57	0.81	0.60	0.65	0.75	0.82	0.84	0.86	0.89	0.89	0.89	0.88	0.89	0.88	0.88	0.89	0.36	0.38	0.38
9	0.65	0.54	0.61	0.81	0.57	0.62	0.77	0.67	0.66	0.80	0.82	0.85	0.79	0.83	0.86	0.87	0.85	0.87	0.87	0.85	0.88	0.30	0.34	0.30
10	0.72	0.50	0.63	0.76	0.63	0.62	0.76	0.70	0.71	0.78	0.89	0.87	0.71	0.91	0.91	0.92	0.93	0.93	0.91	0.93	0.93	0.56	0.58	0.59
11	0.59	0.63	0.65	0.74	0.67	0.58	0.74	0.70	0.67	0.76	0.84	0.86	0.80	0.90	0.92	0.92	0.92	0.92	0.92	0.93	0.93	0.41	0.42	0.42
12	0.83	0.51	0.77	0.82	0.72	0.67	0.83	0.81	0.80	0.66	0.91	0.86	0.84	0.95	0.95	0.93	0.94	0.95	0.93	0.95	0.95	0.62	0.63	0.64
13	0.72	0.47	0.60	0.87	0.72	0.70	0.88	0.77	0.77	0.80	0.87	0.87	0.76	0.89	0.90	0.89	0.90	0.91	0.90	0.91	0.92	0.53	0.56	0.56
14	0.66	0.50	0.49	0.71	0.60	0.60	0.70	0.64	0.68	0.69	0.81	0.83	0.75	0.88	0.86	0.86	0.88	0.88	0.89	0.89	0.90	0.43	0.48	0.46
15	0.71	0.40	0.66	0.85	0.69	0.63	0.89	0.76	0.79	0.76	0.86	0.87	0.76	0.90	0.90	0.88	0.90	0.91	0.90	0.90	0.92	0.60	0.60	0.61
16	0.80	0.66	0.76	0.80	0.67	0.70	0.84	0.71	0.75	0.83	0.89	0.89	0.81	0.92	0.92	0.91	0.93	0.93	0.91	0.93	0.93	0.57	0.57	0.59
17	0.73	0.48	0.72	0.83	0.67	0.61	0.79	0.73	0.76	0.76	0.82	0.84	0.77	0.91	0.91	0.84	0.89	0.88	0.88	0.90	0.90	0.38	0.47	0.41
18	0.69	0.44	0.63	0.65	0.59	0.56	0.67	0.66	0.68	0.78	0.75	0.75	0.76	0.88	0.88	0.86	0.87	0.89	0.85	0.86	0.88	0.43	0.44	0.45
19	0.62	0.06	0.39	0.68	0.56	0.43	0.75	0.65	0.69	0.83	0.79	0.83	0.84	0.85	0.89	0.87	0.87	0.88	0.89	0.87	0.89	0.36	0.34	0.36
20	0.69	0.38	0.60	0.82	0.70	0.75	0.83	0.76	0.84	0.75	0.83	0.86	0.84	0.92	0.93	0.89	0.92	0.92	0.92	0.93	0.94	0.54	0.59	0.57
21	0.75	0.65	0.74	0.72	0.56	0.51	0.77	0.68	0.69	0.73	0.81	0.84	0.79	0.86	0.85	0.88	0.87	0.87	0.90	0.88	0.90	0.52	0.54	0.53
22	0.78	0.48	0.66	0.80	0.75	0.74	0.80	0.78	0.80	0.87	0.88	0.91	0.78	0.86	0.90	0.89	0.90	0.91	0.90	0.90	0.91	0.52	0.55	0.53
23	0.72	0.63	0.70	0.81	0.60	0.59	0.83	0.73	0.74	0.78	0.81	0.81	0.72	0.88	0.89	0.84	0.89	0.89	0.88	0.90	0.91	0.56	0.58	0.58
24	0.70	0.53	0.70	0.79	0.54	0.56	0.84	0.69	0.78	0.85	0.88	0.93	0.74	0.93	0.93	0.91	0.91	0.93	0.91	0.92	0.93	0.55	0.60	0.59
25	0.78	0.70	0.78	0.80	0.66	0.68	0.85	0.72	0.77	0.82	0.86	0.86	0.82	0.87	0.89	0.90	0.89	0.89	0.89	0.86	0.89	0.50	0.57	0.54
26	0.69	0.64	0.61	0.69	0.63	0.63	0.77	0.72	0.77	0.76	0.85	0.88	0.79	0.89	0.90	0.92	0.91	0.93	0.91	0.92	0.93	0.48	0.47	0.47
27	0.66	0.57	0.63	0.79	0.52	0.62	0.84	0.64	0.72	0.81	0.84	0.86	0.86	0.90	0.90	0.89	0.91	0.91	0.91	0.91	0.92	0.47	0.52	0.51
28	0.59	0.33	0.48	0.70	0.67	0.64	0.85	0.71	0.74	0.87	0.86	0.89	0.83	0.85	0.85	0.87	0.86	0.87	0.87	0.85	0.86	0.42	0.42	0.42
29	0.73	0.40	0.65	0.73	0.66	0.64	0.85	0.78	0.77	0.76	0.82	0.83	0.82	0.90	0.90	0.89	0.90	0.89	0.91	0.90	0.92	0.42	0.48	0.44
30	0.69	0.52	0.72	0.73	0.66	0.62	0.80	0.69	0.74	0.80	0.88	0.88	0.81	0.92	0.92	0.89	0.91	0.91	0.91	0.92	0.92	0.59	0.60	0.60
31	0.55	0.53	0.58	0.74	0.52	0.49	0.72	0.49	0.55	0.81	0.83	0.80	0.74	0.87	0.84	0.84	0.87	0.86	0.87	0.88	0.88	0.50	0.50	0.51
32	0.75	0.44	0.65	0.81	0.75	0.69	0.81	0.79	0.76	0.85	0.92	0.91	0.81	0.95	0.94	0.94	0.96	0.95	0.94	0.96	0.96	0.59	0.64	0.63
33	0.65	0.34	0.44	0.75	0.69	0.71	0.73	0.59	0.64	0.80	0.79	0.84	0.79	0.87	0.88	0.86	0.85	0.86	0.87	0.86	0.88	0.39	0.43	0.42
34	0.69	0.45	0.63	0.73	0.53	0.67	0.76	0.65	0.69	0.79	0.83	0.86	0.78	0.85	0.89	0.87	0.86	0.88	0.90	0.88	0.91	0.45	0.49	0.47
35	0.74	0.57	0.62	0.82	0.73	0.73	0.79	0.75	0.77	0.79	0.85	0.86	0.80	0.92	0.90	0.87	0.91	0.91	0.91	0.92	0.93	0.47	0.50	0.49
36	0.81	0.66	0.80	0.81	0.78	0.78	0.85	0.79	0.79	0.83	0.88	0.90	0.80	0.92	0.93	0.92	0.93	0.94	0.94	0.95	0.96	0.52	0.53	0.52
37	0.68	0.48	0.60	0.69	0.55	0.54	0.70	0.67	0.63	0.74	0.76	0.80	0.74	0.86	0.85	0.84	0.86	0.86	0.86	0.86	0.88	0.44	0.49	0.47
38	0.76	0.23	0.66	0.83	0.66	0.71	0.81	0.72	0.78	0.82	0.82	0.86	0.79	0.86	0.89	0.88	0.88	0.90	0.90	0.88	0.91	0.47	0.52	0.49
39	0.69	0.54	0.64	0.75	0.60	0.67	0.78	0.65	0.71	0.80	0.78	0.83	0.84	0.84	0.88	0.87	0.84	0.87	0.87	0.83	0.89	0.56	0.57	0.57
40	0.65	0.35	0.56	0.74	0.59	0.64	0.79	0.70	0.76	0.76	0.85	0.88	0.84	0.92	0.93	0.91	0.94	0.93	0.93	0.95	0.95	0.69	0.69	0.70
41	0.57	0.42	0.49	0.78	0.64	0.55	0.84	0.75	0.76	0.80	0.82	0.87	0.84	0.88	0.89	0.88	0.89	0.89	0.90	0.90	0.91	0.49	0.50	0.49
42	0.57	0.33	0.48	0.82	0.63	0.70	0.82	0.77	0.74	0.77	0.83	0.88	0.72	0.91	0.89	0.87	0.89	0.90	0.88	0.90	0.91	0.33	0.40	0.37
43	0.76	0.47	0.67	0.71	0.53	0.48	0.76	0.66	0.74	0.87	0.87	0.88	0.75	0.90	0.87	0.88	0.92	0.91	0.89	0.90	0.91	0.55	0.65	0.61
44	0.71	0.33	0.61	0.83	0.61	0.59	0.81	0.75	0.78	0.80	0.82	0.85	0.78	0.88	0.89	0.89	0.90	0.91	0.92	0.90	0.93	0.47	0.53	0.50
45	0.82	0.67	0.75	0.90	0.76	0.75	0.90	0.84	0.87	0.79	0.88	0.89	0.81	0.89	0.91	0.92	0.91	0.92	0.93	0.91	0.94	0.61	0.67	0.64
46	0.57	0.55	0.61	0.75	0.49	0.46	0.81	0.63	0.73	0.75	0.81	0.82	0.78	0.88	0.89	0.87	0.87	0.88	0.90	0.89	0.91	0.38	0.46	0.41
47	0.84	0.27	0.71	0.77	0.56	0.55	0.86	0.74	0.76	0.83	0.88	0.90	0.74	0.92	0.92	0.89	0.92	0.91	0.91	0.93	0.92	0.52	0.59	0.56
48	0.76	0.53	0.64	0.78	0.51	0.62	0.81	0.73	0.77	0.77	0.83	0.85	0.79	0.90	0.91	0.91	0.92	0.93	0.93	0.92	0.94	0.53	0.55	0.54
49	0.77	0.39	0.65	0.68	0.52	0.52	0.80	0.78	0.83	0.82	0.86	0.87	0.81	0.92	0.91	0.87	0.91	0.90	0.90	0.92	0.92	0.53	0.57	0.54
50	0.72	0.19	0.59	0.77	0.59	0.60	0.78	0.67	0.73	0.80	0.82	0.88	0.83	0.86	0.90	0.88	0.88	0.90	0.90	0.87	0.91	0.49	0.52	0.52
51	0.72	0.56	0.56	0.84	0.78	0.79	0.85	0.83	0.82	0.82	0.85	0.87	0.78	0.88	0.87	0.89	0.90	0.90	0.91	0.91	0.92	0.53	0.58	0.56
52	0.77	0.25	0.52	0.80	0.61	0.55	0.80	0.75	0.77	0.81	0.84	0.89	0.81	0.86	0.88	0.86	0.87	0.88	0.88	0.87	0.90	0.50	0.53	0.52
53	0.71	0.47	0.63	0.83	0.75	0.74	0.86	0.79	0.78	0.85	0.89	0.89	0.84	0.93	0.92	0.94	0.94	0.95	0.94	0.94	0.96	0.63	0.62	0.62
54	0.73	0.47	0.58	0.78	0.65	0.63	0.78	0.70	0.68	0.71	0.81	0.80	0.75	0.91	0.90	0.87	0.89	0.89	0.88	0.91	0.91	0.39	0.44	0.42
55	0.79	0.45	0.69	0.80	0.73	0.71	0.83	0.79	0.81	0.82	0.89	0.89	0.82	0.90	0.91	0.92	0.92	0.93	0.93	0.92	0.94	0.66	0.68	0.68
56	0.73	0.42	0.70	0.72	0.65	0.61	0.77	0.68	0.71	0.74	0.84	0.84	0.79	0.92	0.92	0.89	0.92	0.92	0.90	0.91	0.92	0.52	0.52	0.53
57	0.63	0.42	0.59	0.76	0.56	0.44	0.72	0.64	0.63	0.76	0.84	0.85	0.74	0.87	0.86	0.87	0.89	0.90	0.87	0.87	0.89	0.43	0.44	0.43
58	0.68	0.47	0.65	0.84	0.62	0.66	0.84	0.75	0.76	0.78	0.83	0.84	0.77	0.86	0.86	0.87	0.88	0.89	0.89	0.88	0.90	0.50	0.52	0.51
59	0.68	0.56	0.64	0.80	0.68	0.64	0.83	0.70	0.76	0.84	0.85	0.88	0.79	0.92	0.92	0.88	0.91	0.92	0.90	0.91	0.93	0.62	0.65	0.65
60	0.77	0.67	0.75	0.86	0.75	0.79	0.86	0.78	0.80	0.86	0.89	0.92	0.85	0.90	0.94	0.90	0.93	0.94	0.95	0.93	0.96	0.69	0.71	0.69
61	0.66	0.62	0.64	0.71	0.61	0.58	0.70	0.66	0.67	0.69	0.80	0.79	0.79	0.88	0.87	0.86	0.88	0.88	0.89	0.88	0.91	0.39	0.41	0.38
62	0.63	0.58	0.63	0.82	0.66	0.61	0.76	0.72	0.76	0.78	0.79	0.84	0.77	0.83	0.88	0.88	0.86	0.88	0.90	0.88	0.91	0.52	0.50	0.51
63	0.74	0.58	0.66	0.74	0.60	0.59	0.77	0.68	0.68	0.81	0.82	0.85	0.79	0.87	0.87	0.86	0.87	0.87	0.89	0.89	0.91	0.43	0.45	0.46
64	0.68	0.41	0.55	0.76	0.66	0.71	0.85	0.73	0.83	0.73	0.79	0.82	0.72	0.87	0.86	0.83	0.86	0.87	0.88	0.88	0.89	0.52	0.53	0.53
65	0.65	0.48	0.48	0.75	0.56	0.64	0.80	0.74	0.78	0.85	0.86	0.88	0.81	0.92	0.92	0.92	0.91	0.92	0.91	0.91	0.92	0.52	0.56	0.54
66	0.71	0.57	0.69	0.85	0.72	0.76	0.84	0.74	0.77	0.80	0.85	0.90	0.84	0.90	0.90	0.84	0.89	0.89	0.89	0.90	0.91	0.50	0.51	0.51
67	0.73	0.69	0.68	0.82	0.58	0.62	0.84	0.83	0.78	0.82	0.83	0.88	0.84	0.90	0.90	0.88	0.91	0.90	0.90	0.91	0.91	0.53	0.54	0.53
68	0.52	0.40	0.56	0.71	0.63	0.57	0.74	0.73	0.70	0.75	0.80	0.79	0.78	0.89	0.88	0.91	0.91	0.91	0.91	0.91	0.92	0.43	0.50	0.47
69	0.69	0.50	0.66	0.81	0.69	0.69	0.84	0.77	0.77	0.78	0.81	0.81	0.79	0.88	0.88	0.92	0.89	0.91	0.92	0.90	0.92	0.34	0.41	0.37
70	0.79	0.49	0.73	0.84	0.72	0.70	0.91	0.81	0.78	0.78	0.86	0.89	0.80	0.92	0.91	0.91	0.92	0.93	0.92	0.93	0.94	0.51	0.53	0.53
71	0.70	0.66	0.66	0.83	0.69	0.74	0.75	0.75	0.74	0.84	0.89	0.89	0.74	0.91	0.88	0.93	0.95	0.94	0.89	0.93	0.92	0.42	0.47	0.44
72	0.60	0.47	0.54	0.80	0.65	0.64	0.80	0.64	0.64	0.78	0.81	0.88	0.84	0.86	0.91	0.91	0.87	0.91	0.91	0.88	0.92	0.42	0.44	0.44
73	0.54	0.28	0.49	0.72	0.46	0.30	0.82	0.63	0.69	0.81	0.84	0.84	0.83	0.90	0.88	0.86	0.89	0.88	0.88	0.88	0.89	0.44	0.47	0.47
74	0.65	0.61	0.70	0.76	0.61	0.63	0.84	0.72	0.74	0.78	0.86	0.87	0.80	0.89	0.91	0.87	0.87	0.88	0.88	0.86	0.89	0.45	0.46	0.46
75	0.79	0.60	0.75	0.82	0.72	0.73	0.80	0.75	0.77	0.87	0.90	0.91	0.84	0.92	0.92	0.89	0.92	0.92	0.92	0.93	0.94	0.59	0.65	0.63
76	0.81	0.62	0.66	0.85	0.74	0.80	0.83	0.82	0.82	0.90	0.89	0.92	0.85	0.91	0.94	0.92	0.92	0.94	0.93	0.92	0.95	0.66	0.68	0.68
77	0.82	0.54	0.76	0.84	0.62	0.67	0.78	0.70	0.76	0.84	0.89	0.90	0.84	0.93	0.93	0.91	0.93	0.93	0.93	0.94	0.94	0.55	0.57	0.57
78	0.70	0.48	0.65	0.76	0.75	0.73	0.83	0.80	0.78	0.77	0.84	0.86	0.79	0.89	0.90	0.91	0.90	0.92	0.92	0.89	0.92	0.54	0.57	0.55
79	0.69	0.33	0.46	0.75	0.60	0.61	0.78	0.69	0.74	0.71	0.80	0.80	0.70	0.84	0.85	0.85	0.86	0.87	0.88	0.86	0.89	0.53	0.56	0.55
80	0.63	0.44	0.48	0.73	0.58	0.61	0.77	0.66	0.70	0.80	0.84	0.85	0.86	0.89	0.90	0.90	0.90	0.90	0.91	0.92	0.92	0.27	0.31	0.30
81	0.72	0.39	0.65	0.80	0.65	0.63	0.79	0.75	0.74	0.86	0.85	0.85	0.78	0.90	0.89	0.90	0.91	0.92	0.90	0.90	0.91	0.65	0.67	0.67
82	0.74	0.39	0.64	0.72	0.48	0.53	0.79	0.64	0.72	0.79	0.86	0.88	0.77	0.90	0.91	0.88	0.90	0.90	0.89	0.91	0.91	0.55	0.61	0.60
83	0.69	0.59	0.62	0.79	0.70	0.70	0.79	0.71	0.75	0.81	0.81	0.82	0.77	0.88	0.86	0.87	0.89	0.89	0.89	0.89	0.91	0.41	0.49	0.46
84	0.78	0.32	0.65	0.81	0.62	0.65	0.78	0.61	0.68	0.78	0.84	0.86	0.79	0.90	0.90	0.88	0.92	0.90	0.91	0.92	0.92	0.59	0.64	0.62
85	0.68	0.21	0.43	0.77	0.61	0.59	0.78	0.67	0.66	0.82	0.86	0.90	0.79	0.88	0.90	0.90	0.91	0.91	0.91	0.90	0.92	0.57	0.58	0.57
86	0.77	0.74	0.70	0.79	0.79	0.71	0.80	0.78	0.76	0.76	0.89	0.85	0.78	0.93	0.94	0.89	0.93	0.92	0.90	0.93	0.93	0.44	0.52	0.48
87	0.76	0.64	0.67	0.84	0.66	0.68	0.81	0.69	0.75	0.78	0.88	0.88	0.80	0.90	0.89	0.88	0.89	0.90	0.89	0.90	0.91	0.55	0.58	0.57
88	0.81	0.45	0.54	0.77	0.67	0.63	0.81	0.70	0.75	0.71	0.83	0.81	0.81	0.89	0.90	0.89	0.91	0.91	0.91	0.91	0.92	0.60	0.64	0.63
89	0.76	0.56	0.68	0.85	0.47	0.43	0.79	0.59	0.64	0.79	0.84	0.88	0.79	0.91	0.89	0.92	0.92	0.92	0.92	0.91	0.92	0.47	0.56	0.50
90	0.64	0.49	0.56	0.74	0.60	0.55	0.74	0.60	0.65	0.68	0.85	0.84	0.78	0.88	0.90	0.89	0.90	0.92	0.90	0.89	0.93	0.41	0.43	0.42
91	0.75	0.56	0.63	0.84	0.72	0.72	0.83	0.75	0.76	0.80	0.87	0.88	0.82	0.88	0.90	0.89	0.91	0.91	0.90	0.90	0.93	0.63	0.65	0.63
92	0.64	0.64	0.57	0.79	0.69	0.65	0.79	0.71	0.73	0.75	0.79	0.81	0.79	0.86	0.87	0.88	0.88	0.89	0.88	0.88	0.90	0.36	0.43	0.40
93	0.65	0.59	0.61	0.78	0.64	0.64	0.77	0.73	0.72	0.85	0.84	0.87	0.83	0.87	0.92	0.93	0.90	0.93	0.94	0.91	0.95	0.45	0.46	0.45
94	0.65	0.33	0.61	0.75	0.51	0.43	0.79	0.61	0.68	0.76	0.82	0.85	0.80	0.85	0.87	0.85	0.86	0.87	0.87	0.87	0.89	0.47	0.56	0.54
95	0.71	0.50	0.62	0.81	0.67	0.70	0.81	0.77	0.79	0.78	0.84	0.84	0.81	0.90	0.89	0.90	0.90	0.90	0.91	0.91	0.91	0.54	0.57	0.54
96	0.66	0.19	0.54	0.75	0.51	0.39	0.79	0.62	0.66	0.79	0.86	0.86	0.79	0.86	0.86	0.84	0.87	0.86	0.87	0.88	0.88	0.53	0.60	0.57
97	0.84	0.79	0.84	0.92	0.75	0.79	0.89	0.82	0.83	0.93	0.92	0.93	0.85	0.96	0.96	0.95	0.96	0.96	0.94	0.96	0.97	0.70	0.73	0.73
98	0.75	0.54	0.74	0.82	0.66	0.68	0.87	0.75	0.74	0.84	0.89	0.90	0.80	0.88	0.90	0.91	0.91	0.92	0.92	0.91	0.93	0.48	0.53	0.50
99	0.66	0.26	0.45	0.76	0.60	0.64	0.82	0.71	0.74	0.77	0.84	0.86	0.81	0.92	0.91	0.88	0.89	0.90	0.89	0.89	0.91	0.52	0.57	0.54
100	0.63	0.52	0.57	0.74	0.48	0.51	0.74	0.66	0.68	0.65	0.76	0.78	0.78	0.84	0.85	0.86	0.84	0.86	0.88	0.87	0.89	0.24	0.30	0.29
