# Supplementary material for: Deep learning empowers genomic selection of pest-resistant grapevine
Source: Hortic Res. 2025 May 7;12(8):uhaf128. doi: 10.1093/hr/uhaf128 (PMC12265469; doi:10.1093/hr/uhaf128)
Supplement: Web_Material_uhaf128 [file web_material_uhaf128.zip › Supplementary Fig 1-12.pdf]

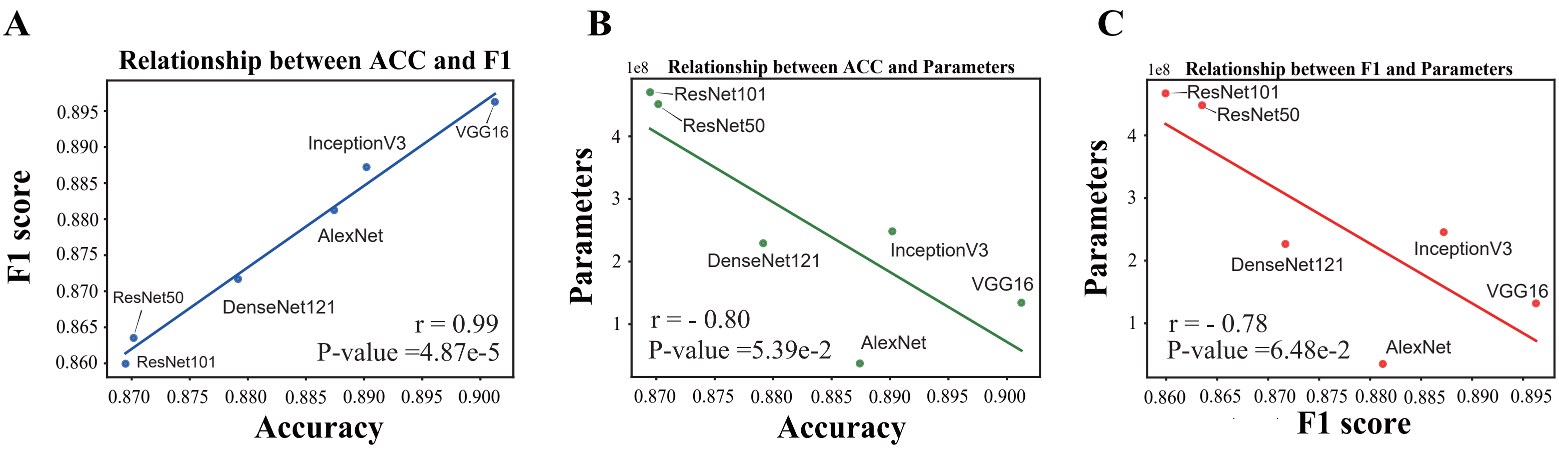

FIG. S. 1. **Analysis of the relationship between F1 score, accuracy and paramters.** (A) A scatter plot shows the average accuracy and F1 scores obtained from six different models in cross-validation, along with their correlations. (B) Correlations and a scatter plot illustrating the accuracy obtained from six models in relation to the number of training parameters. (C) Correlations and a scatter plot illustrating the F1 score obtained from six models in relation to the number of training parameters.

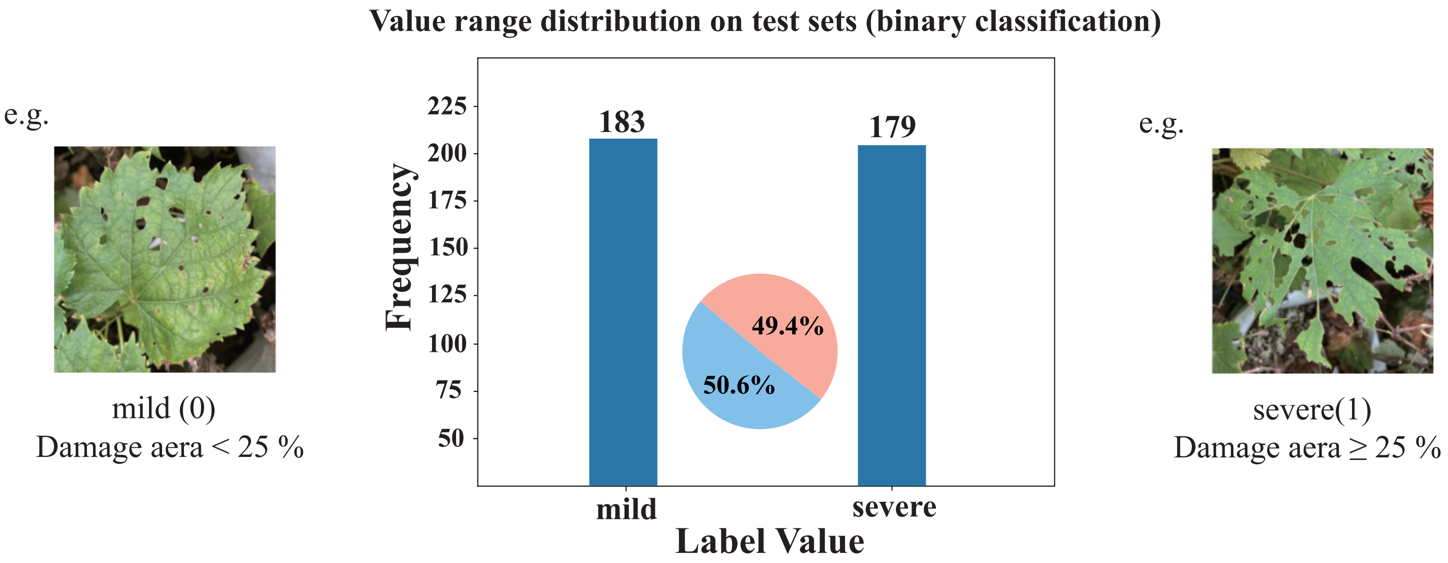

FIG. S. 2. **Numerical distribution of test datasets for classification models.** The numerical distribution of the test set used by VGG16, the examples of severe and mild pest damage on leaves are shown on the left and right, respectively. The bar chart and piechart depict the specific quantities and proportions of different categories, illustrating the severity levels of the pest infestation.

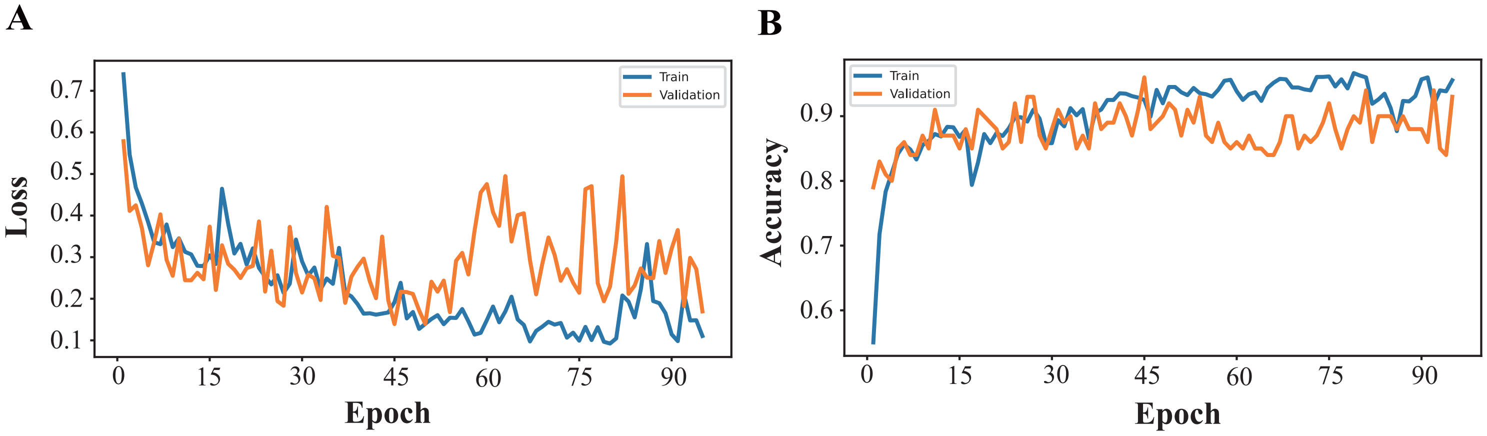

FIG. S.3. **Learning curve of VGG16.** (A) The flctuations in the loss function during VGG16 training. (B) The changes in accuracy during VGG16 training.

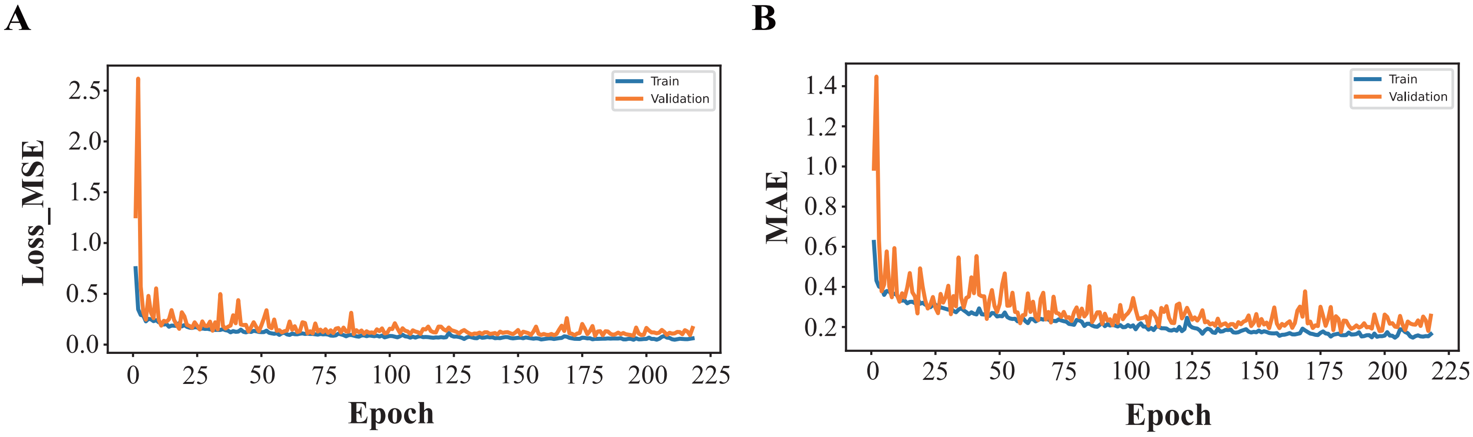

FIG. S.4. **Learning curve of DCNN-PDS.** (A) Changes in loss function (MSE) during VGG16 training. (B) Changes in MAE during DCNN-PDS training.

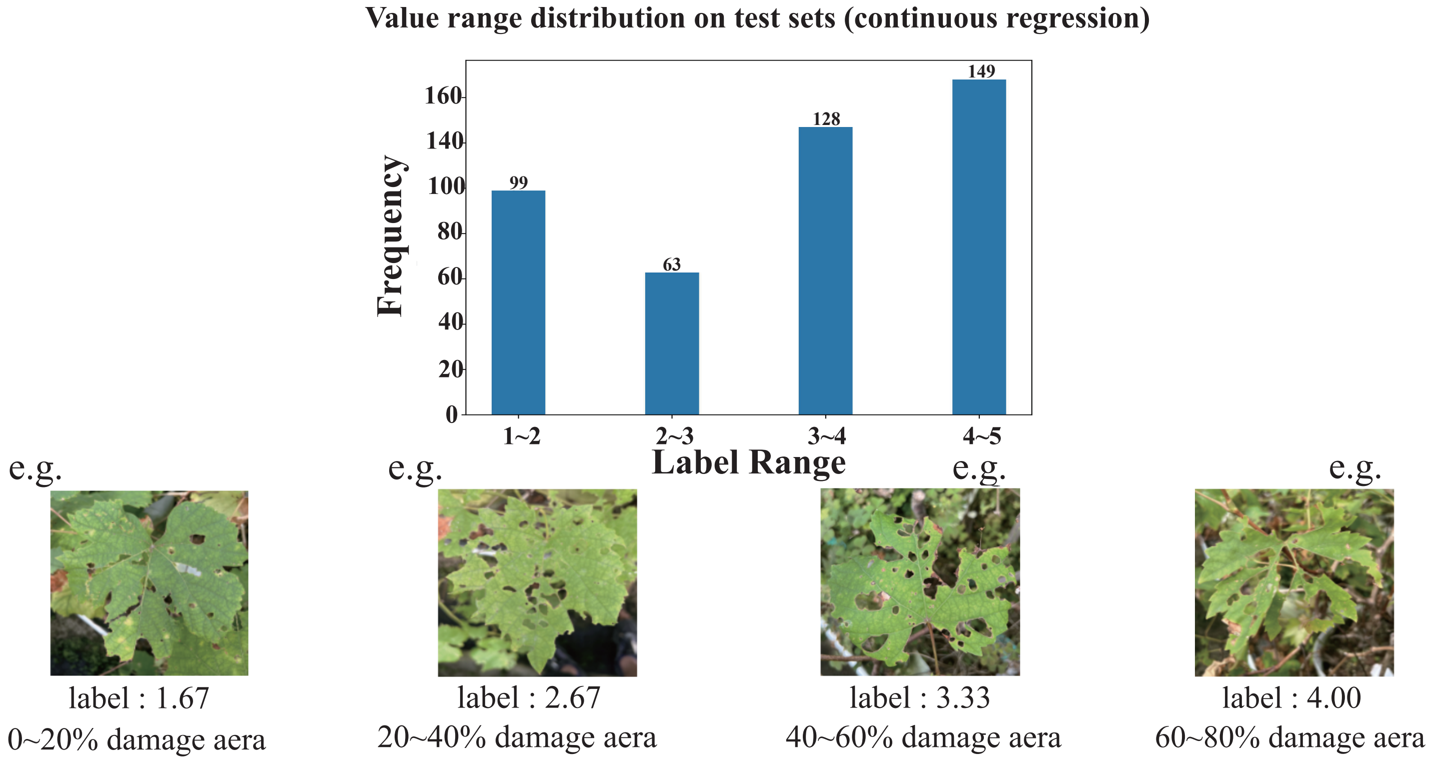

FIG .S.5. **The numerical distribution of the test set used by DCNN-PDS.** The bar chart illustrate the quantity of leaf images labeled for various pest severity levels by different raters, while the bottom section displays some sample examples

A

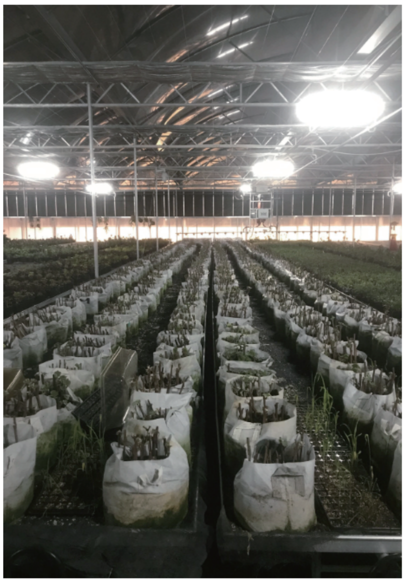

B

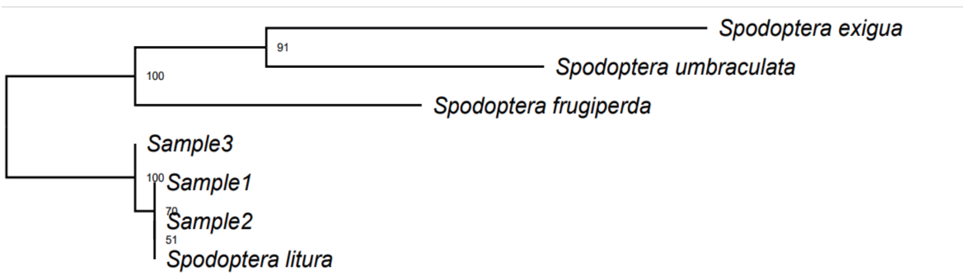

FIG. S.6. **Layout and enviromental conditions of greenhouse viticulture and experimental pest situation.** (A) Plant and cultivation of diferent grape varieties under greeenhouse conditions. (B) The evolutionary tree constucted for experimental pests is sitmated within the same branch as the *Spodoptera litura*.

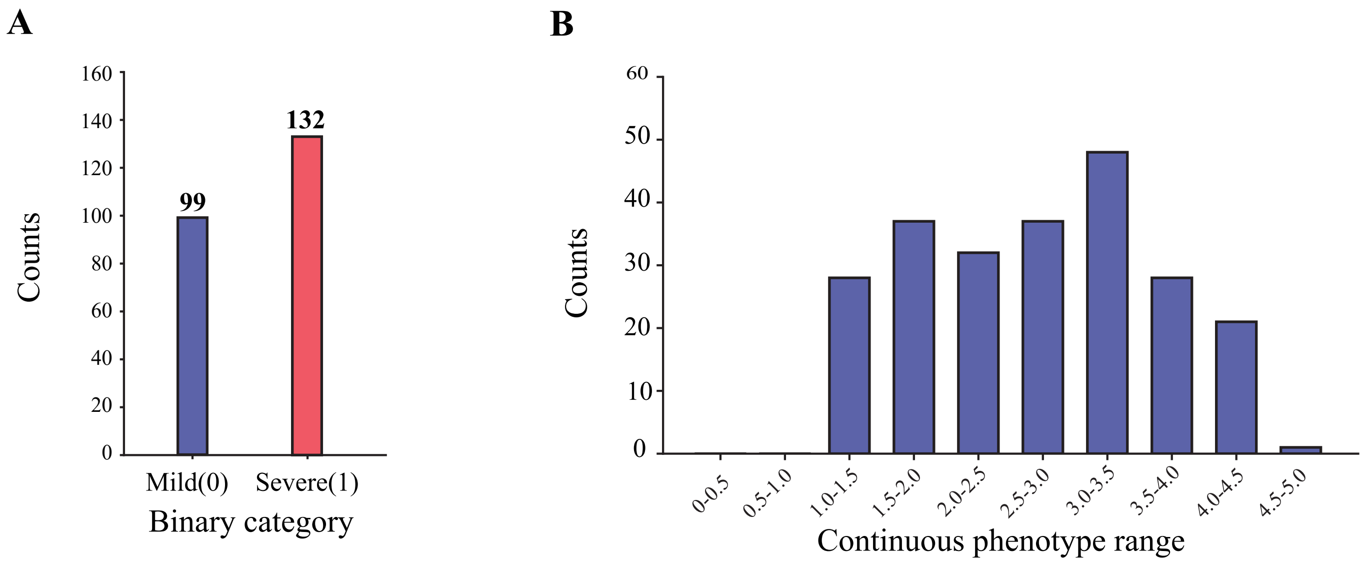

FIG. S.7. **The overall distribution of binary and contimuous phenotypes among 231 varieties.** (A) The biary phenotypic status of herbivore damage measured across all tested accessions. (B) The overall distribution of continuous phenotypes.

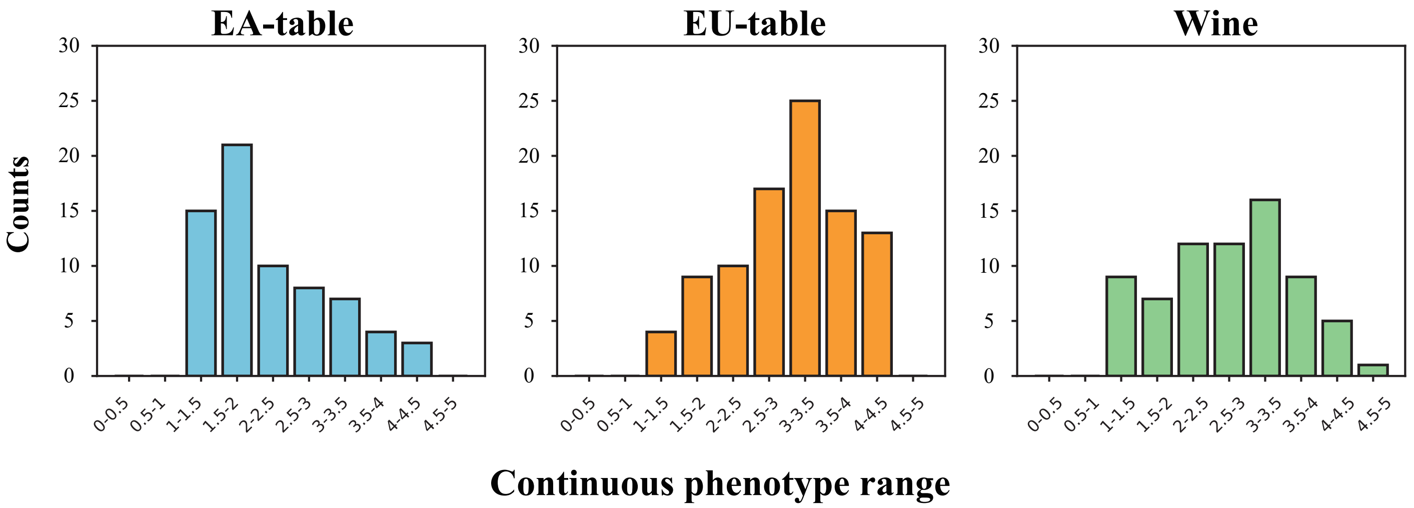

FIG. S.8. **The overal distribution of continuous phenotypes in three different categories of grapes.** From left toright, it represents the distribution of continuous phenotypes in three different categories of grapes: Euro-American table grapes.Euro-Asian table grapes, and Euro-Asian wine grapes.

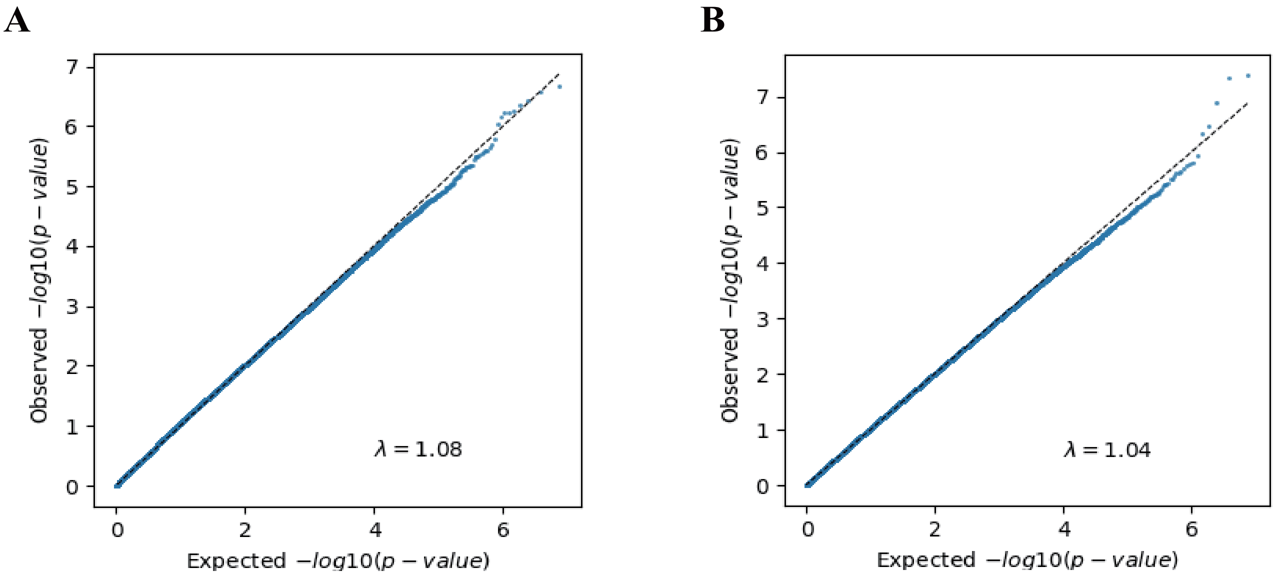

FIG. S.9. **QQ plot of GWAS results.** (A) Binary traits of GWAS result. (B) Continuous traits of GWAS result.

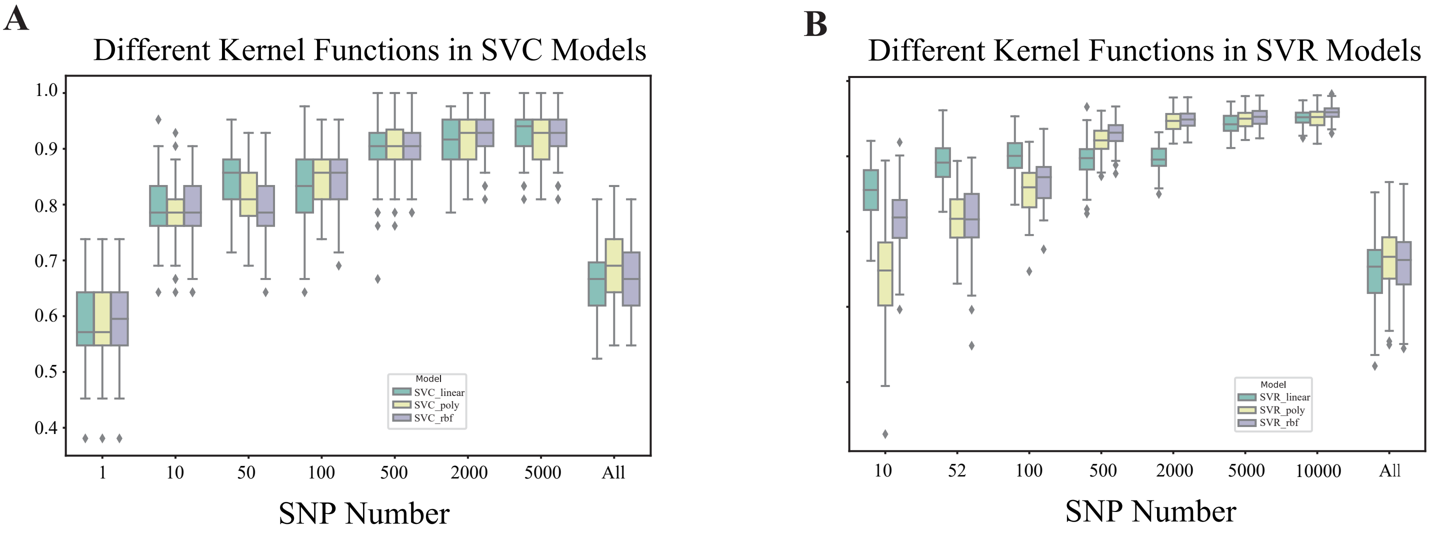

FIG. S.10. **Cross-validation of support vector machine models with different kernel functions.** (A) Performance of support vector classification (SVC) models with diferent kernel fumctions in predicting binary phenotypes. (B) Performance of support vector regression (SVR) models with different kermel fictions in predicting continuous phenotypes.

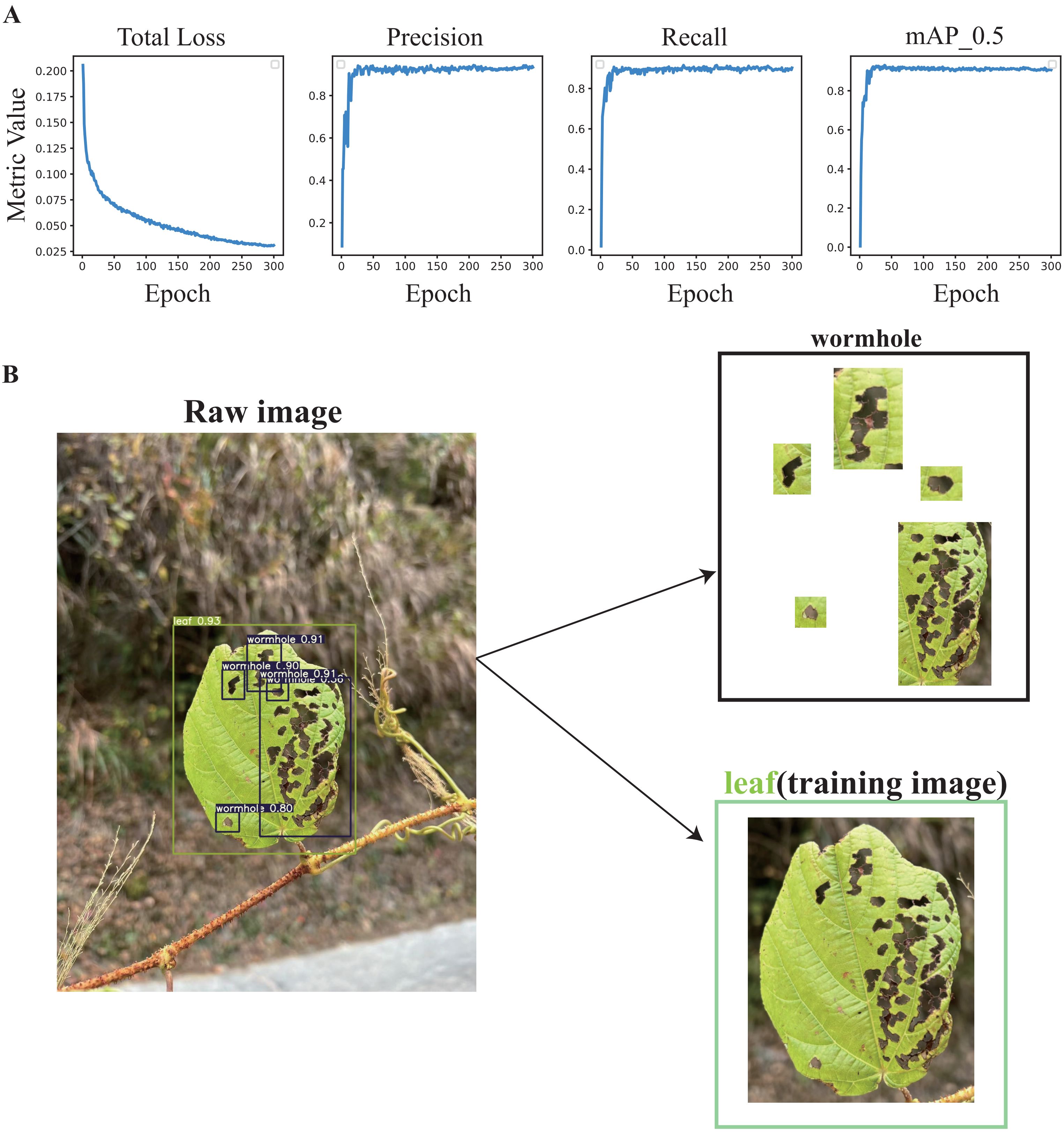

FIG. S.11. **YOLOv5 object detection training curves and image processing.** (A) the YOLO model shows the change curves for total loss, precision, recall, and mAP 0.5 in 300 epochs of trainimg. (B) After performing object detection on the original images, leaf target boxes are extracted as the final training images.

Sample	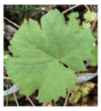	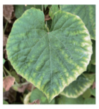	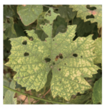	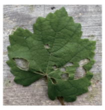	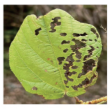	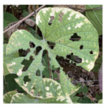	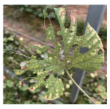	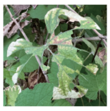	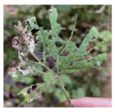	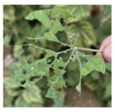
Rater1	0 1	0 1	0 1	0 1	1 2	1 2	1 3	1 4	1 4	1 5
Rater2	0 1	0 1	0 1	1 2	1 2	1 3	1 4	1 4	1 5	1 5
Rater3	0 1	0 1	0 2	0 2	0 3	1 3	1 4	1 5	1 5	1 5
Binary_label	0	0	0	0	1	1	1	1	1	1
PDS_label	1	1	1.33	1.67	2.33	2.67	3.67	4.33	4.67	5

FIG. S.12. **Binary and continuous labeling methods and examples.** Each image received two ratings from the raters a binary rating (left) and a continuous level rating (right). The binary label are determined by majortiy vote, where if two raters provide thesame rating, their ratings are adopted as the label values. The continuous label (PDS_label) are determined by the average of the scores from three raters.
